# Pre-vaccination inflammation and B-cell signalling predict age-related hyporesponse to hepatitis B vaccination

**DOI:** 10.1038/ncomms10369

**Published:** 2016-01-08

**Authors:** Slim Fourati, Razvan Cristescu, Andrey Loboda, Aarthi Talla, Ali Filali, Radha Railkar, Andrea K. Schaeffer, David Favre, Dominic Gagnon, Yoav Peretz, I-Ming Wang, Chan R. Beals, Danilo R. Casimiro, Leonidas N. Carayannopoulos, Rafick-Pierre Sékaly

**Affiliations:** 1 Vaccine and Gene Therapy Institute of Florida, Port St Lucie, Florida 34987, USA; 2 Department of Pathology, University Hospitals of Cleveland and Case Western Reserve University, 2103 Cornell Road, Cleveland, Ohio 44106, USA; 3 Department of Discovery Medicine, Merck Research Laboratories, Rahway, New Jersey 07065, USA; 4 Caprion/ImmuneCarta, Montréal, Québec, Canada H1W 4A4

## Abstract

Aging is associated with hyporesponse to vaccination, whose mechanisms remain unclear. In this study hepatitis B virus (HBV)-naive older adults received three vaccines, including one against HBV. Here we show, using transcriptional and cytometric profiling of whole blood collected before vaccination, that heightened expression of genes that augment B-cell responses and higher memory B-cell frequencies correlate with stronger responses to HBV vaccine. In contrast, higher levels of inflammatory response transcripts and increased frequencies of pro-inflammatory innate cells correlate with weaker responses to this vaccine. Increased numbers of erythrocytes and the haem-induced response also correlate with poor response to the HBV vaccine. A transcriptomics-based pre-vaccination predictor of response to HBV vaccine is built and validated in distinct sets of older adults. This moderately accurate (area under the curve≈65%) but robust signature is supported by flow cytometry and cytokine profiling. This study is the first that identifies baseline predictors and mechanisms of response to the HBV vaccine.

## Abstract (web summary)

Ageing is associated with poor responses to vaccines but the underlying mechanism remains unclear. Here the authors use a systems-based approach to define molecular signatures present before vaccination that correlate with non-responsiveness to hepatitis B vaccination in healthy, elderly adults.

## Graphical abstract

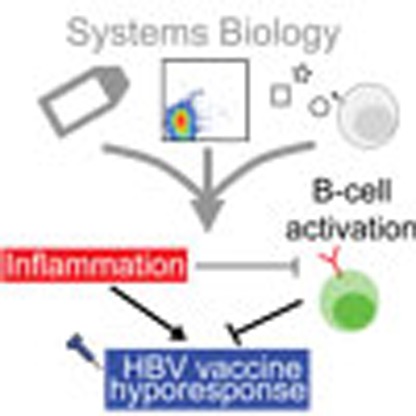

Aging confers elevated risk of illness and death from infection^1^. As the number and proportion of older individuals increase worldwide^2^, prevention of severe or poorly treatable infections among the elderly is ever more pressing. Though vaccination offers a proven approach to such prevention, well-described age-related vaccine hyporesponses (VHR) blunt its potential benefit^3^.

To date, studies of responses to vaccination have revealed associations with human leucocyte antigen polymorphisms^4^, innate and adaptive immune cell phenotypes^5^, suggesting that VHR results from the simultaneous interplay of many elements^6^. The impracticality of sampling human lymphoid organs coupled with limited translatability of animal models^7^ poses a formidable challenge to the discovery of these mechanisms as well as biomarkers of VHR using single-hypothesis approaches. To overcome this challenge, high dimensionality studies of cellular and molecular responses to vaccines have been proposed to speed convergence to actionable mechanistic and biomarker hypotheses; an approach termed ‘systems vaccinology'^8^. This approach has been used to discover early post-vaccination mRNA signatures of responses to yellow fever vaccine YF17D and to correlate strong immunogenicity with cross-lineage cellular activation^9^,^10^. Studies of influenza vaccination have yielded insights into new signalling pathways for B-cell regulation, the potential importance of early neutrophil responses to interferon exposure, and early predictors of post-vaccination immunity^11^,^12^. Systems vaccinology was applied to multi-vaccine comparisons^11^,^13^ as well as to identification of pre-vaccination transcriptomic^14^ and flow cytometric (FCM) correlates of response^15^. Nevertheless, biomarkers predictive of the response to vaccination have yet to be validated and more importantly mechanisms that control the response to vaccination remain to be defined.

Here we first identified a mRNA signature of aging that associated with the seroresponse to the hepatitis B virus (HBV) surface antigen (anti-HBsAg) in naive older adults. Then transcriptomics, polychromatic FCM and serum cytokine profiling were used to generate an integrated model to inform on potential involvement of specific cellular and molecular pathways in nascent anti-HBsAg responses in older adults.

## Results

### Clinical characteristics of the study cohort

**Figure 1:**
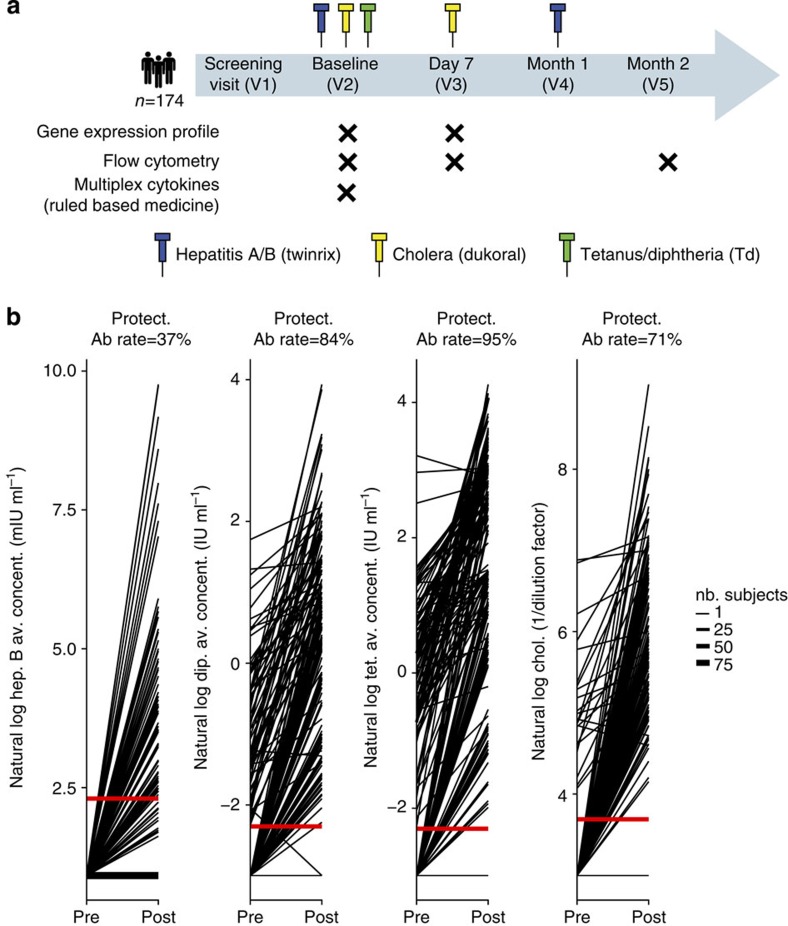
Study design and antibody titres for the three vaccines used in the EM131 study. (**a**) Schematic representation of the study design indicating blood collections and assays performed. All analysed participants were HBV-naive at the time of recruitment and received vaccines for hepatitis A/B, Cholera and Tetanus/Diphtheria. (**b**) Response plots showing antibody titres for HBsAg (hep. B), Diphtheria toxin (dip.), Tetanus toxin (tet.) and Cholera toxin (chol.) for the 174 study participants as a function of the vaccination status (*x*-axis: before and after vaccination). Red horizontal lines indicate standard titre thresholds and the percentage of participants above the protective thresholds are indicated above each plot (Protect. Ab.).

Socio-demographic and serological characteristics of this study's cohort (designated as the EM131 cohort) appear in Supplementary Table 1. One-hundred and seventy-four healthy adults received vaccines against Hepatitis A (HAV)/HBVs, Diphtheria/Tetanus toxoids and Cholera bacteria/toxin (Fig. 1a). The percentage of study participants that mounted protective Ab titres (anti-HBsAg: 10 mIU ml^−1^, anti-Tetanus toxoid: 0.1 IU ml^−1^, anti-Diphtheria toxoid: 0.1 IU ml^−1^ and anti-Cholera toxin: dilution 1:40)^16^,^17^,^18^ were, in decreasing order, 95% for Tetanus, 84% for Diphtheria, 71% for Cholera and 37% for HBV (Fig. 1b). Only the HBV vaccine involved an antigen to which all study participants were naive and presented the lowest response rate (37%, after two injections) even when we restricted the analysis to participants with undetectable Ab titres pre-vaccination (Supplementary Fig. 1a). We focused our analysis on the HBV vaccine response (primary immunization). Kernel density estimation of anti-HBsAg titres showed that a cutoff of 5 mIU ml^−1^ demarcated study participants as HBV vaccine responders (Ab titres≥5 mIU ml^−1^) and poor-responders (Ab titres below detection threshold) (Supplementary Fig. 1b). Logistic regression between demographic variables and HBV vaccine response revealed that age and gender were significantly associated with response to the HBV vaccine on the EM131 data set (Supplementary Table 2). Here we investigated the association of age-related transcriptomic changes with the response to HBV vaccination.

### Impact of age on the human immune transcriptome

**Figure 2:**
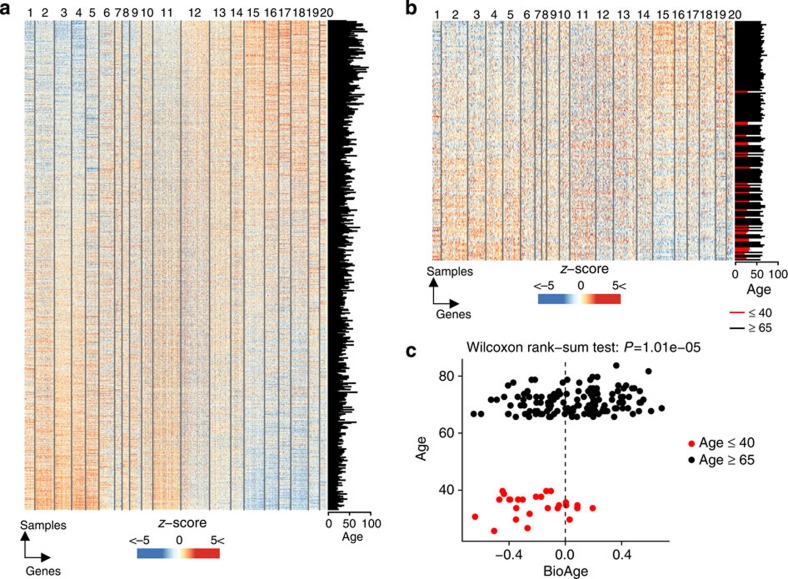
Development of the BioAge signature and application to the EM131 cohort. (**a**) Expression of the 2,285 age-related transcripts derived from SAFHS data set (*n*=1,240)^19^; all transcripts correlating with chronological age (moderated *t*-test: adjusted *P*≤0.05) were clustered in 20 modules using *k*-means clustering. In the heat map, transcripts (columns) were ordered by their membership to the 20 modules; the 1,240 samples/participants (row) were ordered by their BioAge score (signed average expression of the age-related transcripts). Transcript expression was transformed to *z*-score and is depicted in blue to white to red colour scale. The chronological age is given in the bar plot at the right. (**b**) Expression of the 2,285 transcripts of the BioAge signature in the EM131 data set (*n*=174). The chronological age of the 174 participants is given in the bar plot at the right. (**c**) Scatter plot showing the chronological age as a function of the BioAge. The vertical line indicates a BioAge score of 0. We observe that young participants (red circles) have significantly younger BioAge; and among elderly (black circles) about half of participants have a young BioAge (<0) while the other half have an old BioAge (oAge).

The impact of age on the transcriptome of peripheral blood mononuclear cells obtained from healthy participants was studied under controlled conditions (morning, after fasting) in the San Antonio Family Heart Study (SAFHS, *n*=1,240)^19^. Linear regression models were used to identify transcripts correlated to age (Methods formula 1). Using a cutoff of adjusted *P*≤0.05 (with the Benjamini–Hochberg method to correct for multiple testing), we identified 1,143 and 1,142 transcripts positively and negatively correlated to age, respectively, in the SAFHS data set (Fig. 2a and Supplementary Table 3a). The Gap statistic method estimated 20 as the optimal number of clusters that explained gene-expression variability (Supplementary Fig. 2a). *k*-means clustering was used to regroup the 2,285 transcripts in 20 modules (M1 to M20) based on their co-expression in the SAFHS data set (Supplementary Table 3a).

### Pro-inflammatory pathways prevail in elderly participants

To identify the biological pathways associated to older age, we assessed the overlap between each of these modules and Ingenuity canonical pathways (Fisher's exact test). Using a cutoff of *P*≤0.05, we identified 250 biological pathways enriched in the 20 modules (Supplementary Table 3b). On the one hand, pathways positively associated with older age included pathways that regulate cell motility (M12 and M14) and genes downstream of integrin signalling (M15). A type II interferon signalling module (M17) characterized by the induced expression of *TNF* and *IFNG* was also enriched in elderly participants. Inflammatory responses that include complement genes (M16) downstream of interferons, as well as T-cell/natural killer (NK)-cells-mediated cytolysis (M19) markers such as perforin and granzyme B, were also positively associated with older age. Module M19 was highly enriched in NK-cell markers such as the killer cell immunoglobin-like receptors (*KIR2DL2*, *KIR3DL1* and *KIR3DL3*). On the other hand, pathways enriched in younger participants included immunological pathways such as B-cell signalling (M1) comprising several B-cell markers (*CD79A*, *CD79B*, *CD19*, *CD20* and *CD22*), T-cell receptor signalling (M6) that included *ZAP70*, *TRAF1*, *TCF7* a marker of memory T cells^20^ and antiviral response (M8) that included the antiviral signalling gene *MAVS*. MYC signalling (M3), which triggers metabolic pathways such as amino acid metabolism (M2 and M4), fatty acid biosynthesis (M9) and lipid metabolic pathways (M11), all critical for the induction of T- and B-cell memory, was enriched in younger participants. These results indicate that each of the 20 modules reflect a specific biological process that is positively or negatively associated to aging. These results also confirm that elderly show constitutive upregulation of several pro-inflammatory pathways downstream of type I/II interferons which are suggested to favour immunosenescence^21^.

### An age-related signature is observed prior to vaccination

We developed an aggregate score that integrates transcriptomic changes associated with older age by subtracting the average expression of the 11 modules negatively associated with age from the average expression of the 9 modules positively associated with age (Methods formula 2). We calculated the BioAge score on the SAFHS cohort and as expected observed a significant correlation between the BioAge score and chronological age (Pearson's correlation: *r*=0.421, *P*<2.2e−16; Supplementary Fig. 2b). The same modules that discriminated elderly and young participants in the SAFHS cohort also distinguished the two age groups in the EM131 cohort (Fig. 2a,b). There was a statistically significant association between chronological age and BioAge on the EM131 cohort (Fig. 2c, Wilcoxon rank-sum test: *P*=1.01e−05), confirming the validity of this score to reflect the impact of age on gene expression in whole blood cells. While most young study participants had a low BioAge score, the BioAge predictor divided elderly patients (≥65, EM131 elderly cohort) into two groups designated as BioAge young (*n*=62, age∈[65, 78]) and BioAge old (*n*=76, age∈[65, 83]) (Fig. 2c).

### BioAge associated with HBV vaccine response pre-immunization

**Figure 3:**
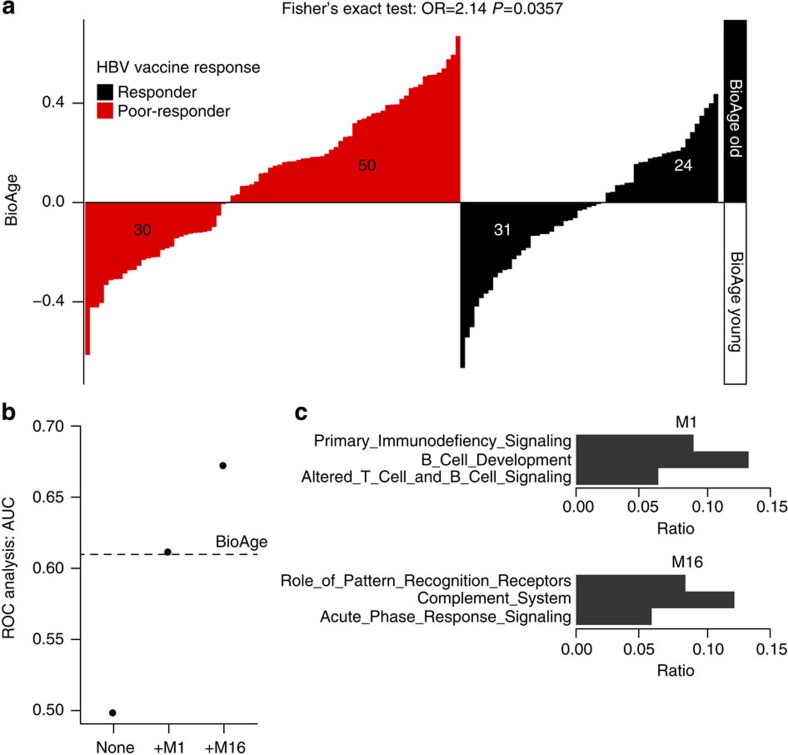
BioAge predicts HBV vaccine response. (**a**) BioAge classification of the 135 elderly patients based on gene expression prior to vaccination is significantly associated with the response to HBV vaccination. Each bar of the bar plot represents one of the elderly participants in the EM131 cohort. The height of the bar indicates the BioAge score of that donor. Bars were ordered by increasing level of the BioAge, separately for the HBV poor-responders (in red) and HBV responders (in black). Fisher's exact test *P* values are given on the plot. (**b**) Forward selection among the modules composing the BioAge signature resulted in the selection of modules M1 and M16 as optimal signatures, predicting HBV vaccine response in the EM131 elderly cohort (age≥65). (**c**) Pathway enrichment analysis on the genes included in modules M1 and M16 using the IPA canonical pathway database. Fisher exact test was performed to assess statistical enrichment and gene sets with FDR-corrected *P* value≤0.05 are presented.

We determined if different BioAge scores correlated with different responses to HBV vaccination. Both the BioAge score (Supplementary Fig. 3) and the two groups of elderly identified by the BioAge signature (Fisher's exact test: odds ratio (OR)=2.14, *P*=0.0357; Fig. 3a) were associated with the rate of response to the HBV vaccine. Importantly, in a multivariate analysis including available clinical parameters, the BioAge was both a better predictor of the HBV response than chronological age and independent of gender (Supplementary Table 2). Among the 20 gene modules defined in the BioAge signature, 2 modules M1 (lower expression in BioAge old) and M16 (high expression in BioAge old) allowed the most accurate prediction of the response to the HBV vaccine (Fig. 3b). Pathway enrichment analysis showed that module M1 was enriched in genes encoding two critical components of the B-cell receptor, that is, *CD79A* and *CD79B*, B-cell activation markers such as *CD19*, *CD22*, the transcription factor *POU2AF1* (a protein essential for the response of B cells to antigens and required for the formation of germinal centres)^22^ and *MZB-1*, a chaperone involved in assembly of IgM^23^ (Fig. 3c). Module M16 includes genes of the acute phase response, that is, the complement gene C1, the mediator of type II interferon response *MYD88* and the transcription factor *IRF7*. IRF7 target genes such as *LILRA5* (a marker expressed by TNF-producing macrophages), *S100* (a molecule associated to cellular senescence), *IL-15* (a pro-inflammatory cytokine that triggers CD8 T-cell and NK-cell expansion), *PILRA* (an inhibitory receptor that acts through SHP-1) and *PRELID-1* (a gene that negatively regulates the development of TH2 responses) were also present in module M16 (Fig. 3c). Overall, module M1 includes mostly genes involved in B-cell activation while module M16 includes genes linked to inhibition of the B-cell response.

To test the accuracy of this signature, we performed a receiver operating characteristic (ROC) analysis that indicated that the BioAge signature could predict the response to the HBV vaccine with an accuracy of 60.0% in the EM131 elderly cohort (Fig. 3b, permutation test: *P*=0.0163). Collectively these results show for the first time that transcriptional profiling allows the identification before vaccination, albeit with a moderate accuracy, of participants that will mount a poor response to the HBV vaccine. This signature highlights the interplay between the innate inflammatory pathways and B cells in the response to the HBV vaccine.

### A 15-gene signature predicts the HBV vaccine response

**Figure 4:**
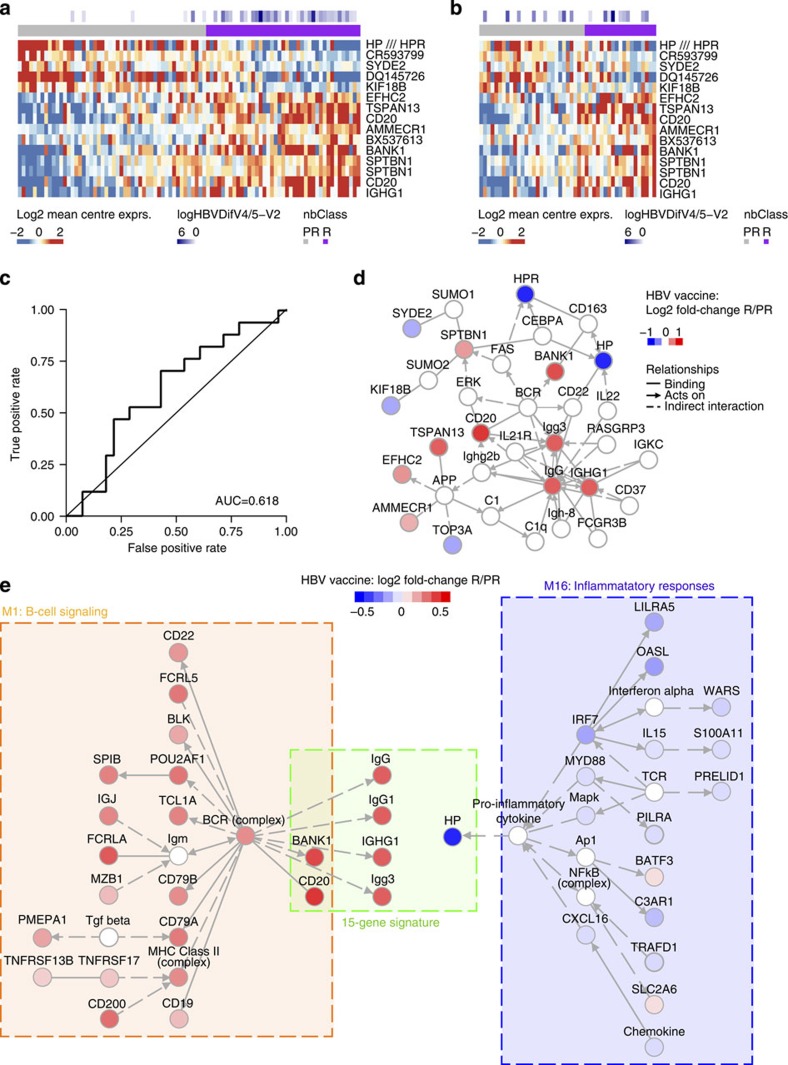
Identification of gene-expression signature predicting the HBV vaccine response. (**a**) Expression of 15 genes identified as predictors of the response to the HBV vaccine in the EM131 training set (*n*=95). The mean-centred gene expression is represented using a blue to white to red colour scale. Rows and columns correspond to the genes and the profiled samples, respectively. Samples were ordered by increasing levels of their predicted probability of responding to the vaccine (that is, posterior probability). Antibody response to the HBV vaccine (log(HepBDifV4/5-V2)) and the response group predicted by the 15-gene signature (nbClass) are presented in coloured squares above each sample. (**b**) Expression of the 15-gene signature on the EM131 test set (*n*=49). (**c**) ROC curve and area under the curve (AUC) for the prediction of the HBV vaccine response using the 15-gene signature on the EM131 test set (*n*=49). (**d**) Network inference based on the 15 markers identified as predictors of the response to the HBV vaccine. Red and blue nodes represent genes induced or repressed in HBV vaccine responders (R) compared with poor-responders (PR), respectively. (**e**) Networks were inferred for the BioAge module 1 combined to the 15-gene signature and the BioAge module 16 combined to the 15-gene signature, respectively. Nodes included in the BioAge or the 15-gene signature are coloured by their fold-change between R versus PR to the HBV vaccine in the EM131 training set.

We used an independent, supervised approach to identify other gene sets/pathways important for predicting the response to the HBV vaccine. The EM131 elderly cohort was randomly split into a training cohort (2/3 of the study participants) and a test cohort (1/3 of the participants). We were not able to identify a single transcript that was significantly differentially expressed between HBV vaccine responders and poor-responders of the EM131 training set (linear model for microarray (LIMMA): adjusted *P*≤0.05, Supplementary Table 4a). We then tested if a combination of genes (multivariate model) could distinguish the two classes of responders. To that end, a naive Bayes classifier based on the top 15 differentially expressed genes between responders and poor-responders to the HBV vaccines was built on the EM131 training set (Fig. 4a). The accuracy of the resulting 15-gene signature was assessed on the EM131 training set by 10-fold cross-validation and on the EM131 test set and found to be 62.6% and 62.2%, respectively (Fig. 4b,c, Supplementary Fig. 4 and Supplementary Table 4b). A permutation test was performed to assess the probability of building a signature of the same size (15 genes) with better or equal accuracy to the 15-gene signature on the EM131 test set. That probability was 0.0381, suggesting that building a better predictive model randomly is improbable. Network inference revealed that several members of this 15-gene signature included markers of B cells (for example, *CD20*, *IGHG1*), as well as downstream targets of B-cell receptor signalling (for example, *BANK1*) and molecules known to have functional interactions with IgG (for example, *C1*, *FCGR3B*; Fig. 4d). Network inference revealed that both the 15-gene signature and the BioAge signature shared members of the B-cell signalling (*CD20*, *BANK1*; Fisher's exact test: *P*=7.99e−07) and inflammation pathways (Fig. 4e). Both signatures showed upregulated expression of genes directly associated with inflammation. In the BioAge M16, *IRF7* (the master switch of type I interferon) and its downstream targets *IL-15* and *OAS1*, as well as *NFkB* and *C1* (hallmarks of inflammation) were associated with lower Ab titres. In the 15-gene signature, haptoglobin (*HP*), an acute phase protein and also a target gene of type I interferon signalling, was associated with low Ab titres. Pathway analysis performed on a list of genes derived with a less stringent statistical cutoff of nominal *P*≤0.05 confirmed the positive association of B-cell signalling and the response to HBV vaccine (Supplementary Table 4c). Collectively, two independent bioinformatic approaches showed that B cells and inflammation are important predictors of HBV vaccine response in the elderly.

### FCM and RBC counts reveal predictors of HBV vaccine response

**Figure 5:**
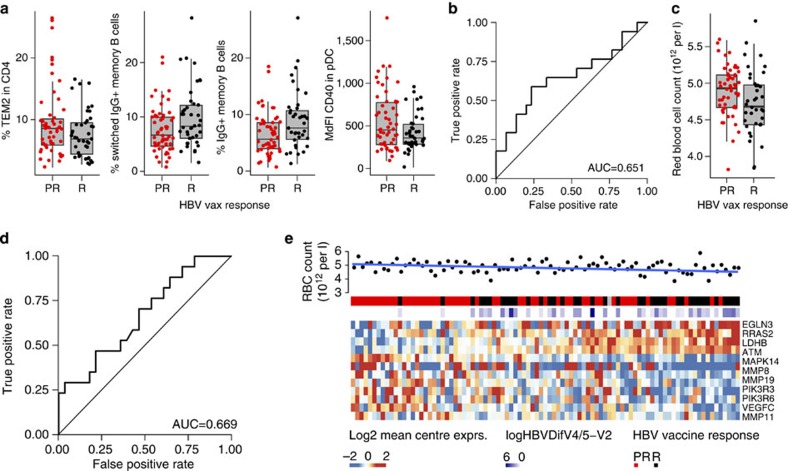
B-cell subset as well as innate immune cell subsets and RBC counts are predictive of the response to the HBV vaccine. Polychromatic FCM was used to identify cell surface markers of the response to the HBV vaccine. (**a**) Box plots presenting the frequencies of the four FCM markers selected in the forward selection model in responders and poor-responders to the HBV vaccine on the EM131 training set (*n*=95). (**b**) ROC curves for the prediction of the HBV vaccine response using the FCM data on the EM131 test set. (**c**) Box plots presenting the RBC counts (measured in a 28-day window prior to HBV vaccination) in responders and poor-responders to the HBV vaccine on the EM131 training set (*n*=95). (**d**) ROC curves illustrating the prediction of the HBV vaccine response using RBC counts on the EM131 test set. (**e**) Heat map representation of the genes differentially expressed between HBV vaccine R and PR overlapping the HIF-1a canonical pathway in the training set. The mean-centred gene expression is represented using a blue–red colour scale. Rows and columns correspond to the genes and the profiled samples, respectively. Samples were ordered by increasing level of their expression of the genes associated to the vaccine response (mean-rank ordering). Antibody response to the HBV vaccine (logHepBDifV4/5-V2) and HBV vaccine response group (HBV vaccine response) and red blood cells counts (RBC in 10^12^ per l) are presented in coloured squares above each sample. The *P* value of *t*-test between RBC and the ordering of the samples is 0.0462.

A set of 96 FCM markers was tested for their association with the HBV vaccine response (Supplementary Table 5a and Supplementary Fig. 5a). Logistic regression was used to determine the association of each FCM marker (frequencies of cells) to antibody titres to the HBV vaccine (Table 1). Two cell subsets defined by FCM, namely frequencies of switched IgG+ memory B cells (CD3−, CD19+, HLADR+, CD27+, IgG+) and of IgG+ memory B cells (CD3−, CD19+, HLADR+, CD27+, CD10−, CD20+, IgG+) were identified as significant predictors of the response to the HBV vaccine (Fig. 5a and Table 1). Frequencies of CD4 T-effector memory 2 (CD28−, marker of immunosenescence^24^) and CD40 on plasmacytoid dendritic cells (pDCs), another byproduct of chronic immune activation and inflammation^25^, correlated negatively with HBV vaccine response (Fig. 5a). A multivariate regression model combining four FCM markers (% switched IgG+ memory B cells, % IgG+ memory B cells, % CD4 T-effector memory 2 and the median fluorescence intensity of CD40 in pDCs; Table 1), trained on the EM131 training set, was able to predict the response to HBV vaccine in the test set with an accuracy of 63.3% (Fig. 5b, permutation test: *P*=0.0468).

We also screened a panel of 47 serum cytokines and chemokines for their association with the response to HBV vaccination. Three inflammation-associated proteins—IL-15, IL-1ra and TNFR2—were negatively associated with HBV vaccine response on the EM131 training set (Supplementary Table 5b,c and Supplementary Fig. 5b). This model was not significantly associated to the HBV vaccine response in the EM131 test set (Supplementary Fig. 5c, permutation test: *P*=0.250).

Similarly, a set of haematologic markers was screened for their association with the HBV vaccine response. Interestingly, higher red blood cell (RBC) counts were associated with low Ab titres in elderly (Table 1 and Fig. 5c,d). Collectively our data show for the first time that baseline measures of immune function as defined by elevated frequencies of effector cells of the response to vaccines (B cells and T-helper cells) and inflammation (downstream of infections or other metabolic pathways) are associated to the response to vaccination.

### B-cell and inflammation interplay shapes HBV vaccine response

**Figure 6:**
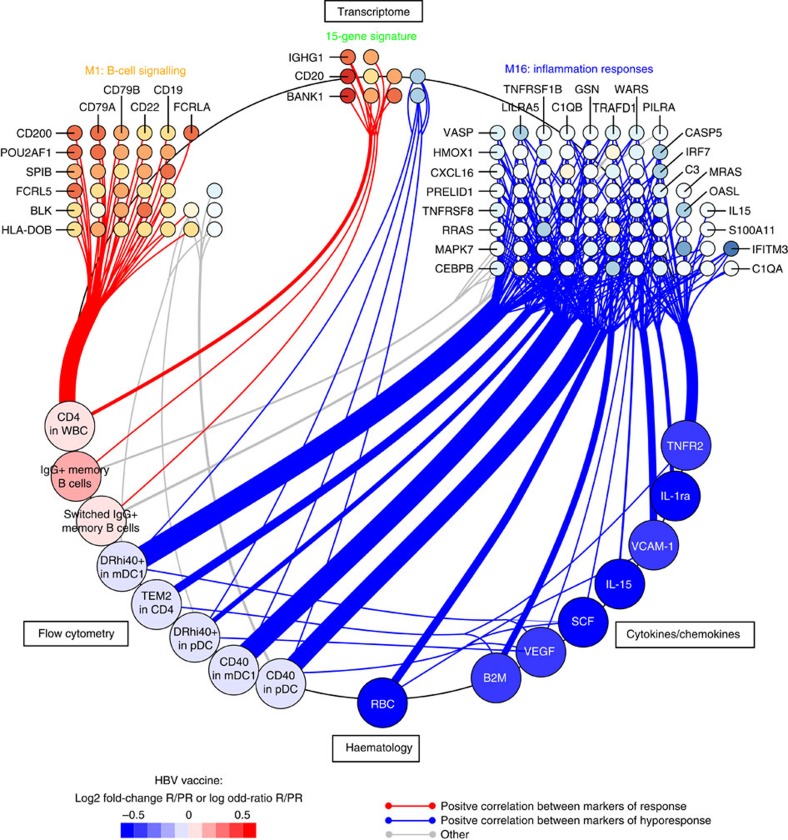
Integrative analysis reveals positive correlations between biomarkers of HBV vaccine response. A projection-based multivariate approach was adopted to assess the correlation between transcriptomic, FCM, haematologic and cytokine/chemokine expression data sets. Least-square regressions between pairs of data sets was performed using the R package ‘mixOmics'. The resulting regression coefficients were converted to Pearson's correlations between pairs of features of the different data sets (presented at each quadrant of the figure). Significant (*t*-test: *r*≥0.188, *P*≤0.05) positive correlations are presented as edges and the features as vertices. The vertices are coloured by fold-change between HBV vaccine responders versus poor-responders of the EM131 training set.

We used least-square regression models^26^ to demonstrate that the gene-expression signatures underlying the poor response to HBV vaccine in elderly participants were correlated to levels of effector cytokines, frequencies of specific subsets of CD4 cells and innate immune cells (Fig. 6). We included the transcriptomic, FCM, cytokines/chemokines and RBC counts data sets in the integrative analysis. Results of this analysis showed that the two independently derived B-cell gene-expression signatures (that is, the BioAge module M1 and the 15-gene signature) were correlated with frequencies of memory B cells identified by FCM and with increased CD4 counts. Conversely the gene-expression signature of inflammation (for example, *C1QB*, *C3*, *IRF7*) correlated positively with FCM markers of activated innate immune cells and immunosenescent CD4 T cells (TEM2 in CD4s, CD40 in mDC1s and CD40 in pDCs, respectively) (Fig. 6). This integrated analysis showed the correlation between heightened levels of pro-inflammatory cytokines (for example, SCF, TNFR2 and VEGF) and increased frequencies of activated innate immune cells (TEM2 in CD4s, CD40 in mDC1s and CD40 in pDCs, respectively). Overall, the gene-expression signatures that highlight the contrasting roles of B cells and inflammation on the development of the response to HBV vaccine were confirmed by the analysis of cellular subsets and of cytokine profiles.

## Discussion

In this study, we provide an integrated model and propose mechanisms that could explain the response to vaccines prior to primary vaccination. This study cohort included a training set of 95 elderly participants (Supplementary Table 1) that was used to identify biomarkers of the response to vaccines; a distinct set of 49 participants sharing similar demographic and clinical characteristics served as an independent test set. This study design allowed the assessment of the accuracy and robustness of biomarkers that can predict the response to HBV vaccine. Two types of signalling pathways, B-cell signalling and inflammation, were thus identified using two independent bioinformatic approaches as important regulators/predictors of the response to HBV vaccination. Both classifiers, the BioAge signature and the 15-gene signature, were confirmed in an independent test cohort and showed for the first time that it was possible to distinguish high responders from low responder to HBV vaccination with accuracies >60%. While this is lower than accuracies previously reported post vaccination^10^, it is well within the range of those reported for baseline predictors to cancer treatments (area under the curve=67.7% (50.0%, 80.4%))^27^. The opposing impacts of B-cell signalling and inflammation reflected in both classifiers were confirmed in independent set of participants and using three different experimental approaches: gene expression (Figs 3 and 4), FCM (Fig. 5a,b) and cytokine profiling (Supplementary Fig. 5b,c). The fact that all three approaches identified these two major pathways as being associated to a successful or a failed response to vaccination highlight the robustness of these signatures.

Ab titres are the established correlates of vaccine efficacy for the HBV vaccine, implying that the presence of the appropriate subset of B cells prior to vaccination could favour a strong response to the vaccine. Our results confirm this observation. First, FCM results show that the percentage of memory B-cell subsets (% switched IgG+ memory B cells and % IgG+ memory B cells) is a significant univariate predictor of HBV vaccine response (Fig. 5a) although absolute counts of total B cells were not a significant predictor of the response to HBV vaccine. Similar results were reported by Tsang *et al.*^15^ where a specific B-cell phenotype, namely percentage of CD38+CD27+ memory B cells, was the main feature of a model that predicted the Ab response to H1N1 vaccine in participants with undetectable Ab titres to H1N1 prior to vaccination. Transcriptomic profiling shows that several B-cell markers including components of the BCR complex (that is, *IGH*, *CD79A*, *CD79B* and *CD19*) were also increased in responders and were positive predictors of the HBV vaccine response in our training and test cohorts. Transcriptional profiles show that a strong response to HBV vaccine additionally requires that B cells express transcription factors such as POU2AF1 and MZB-1 that programme B cells for Ab production and trigger the formation of germinal centres^22^,^23^. Transcriptional profiling further highlighted the role of *TNFRSF13B*, the receptor for BAFF and APRIL cytokines involved in the maturation of the humoral response, and a positive correlates of strong responses to vaccination^13^. Our results show that increased frequencies of CD4+ T cells, which can provide help to B cells in white blood cells, are associated with higher anti-HBsAg titres (Fig. 5a)^28^.

Integration of FCM and cytokine profiles highlights the role of elements of the pro-inflammatory response, which is negatively correlated to antibody production. Increased frequencies of activated innate immune cells (DR+CD40+ mDC and CD40+ pDCs) were associated with transcriptional signatures and cytokines that predicted the poor Ab response (Fig. 5a). These cells produce type I interferons in response to inflammatory signals and this is confirmed by the induction of the transcription factors IRF7 and IRF9 in poor-responders to the HBV vaccine (Fig. 4 and Supplementary Table 4a). Activation of the interferon(s) transcriptional pathway was confirmed by the upregulation of several interferon-induced genes downstream of *IRF7* including *OASL*^29^,^30^, and *LILRA5* (ref. 29) in participants who responded poorly to the HBV vaccine. The induction of the pro-inflammatory cytokine IL-15, downstream of type I interferon^29^,^30^, was confirmed at the gene and protein levels in poor-responders to the HBV vaccine (Fig. 6). The pro-inflammatory complement complex (for example, *C3*, *C1QA*, *C1QB*) and VEGF, a positive regulator of angiogenesis that promotes the chronic inflammatory process^31^, are also negatively associated to antibody production (Fig. 6). Increased levels of *MYD88* and *TRAF*, downstream of TLR and IL1 signalling, are observed in poor-responders to HBV vaccination (Supplementary Table 4a). Both *MYD88* and *TRAF* are negatively correlated to the frequency of CD4 T cells, a marker of good response to HBV vaccination (Supplementary Fig. 6b, Pearson's correlation: *r*=−0.347 *P*<0.05 and *r*=−0.353 *P*<0.05, respectively). Moreover, *C3* and *WARS*, two target genes of type I interferons, were negatively associated to the frequencies of the two memory B-cell subsets. In contrast, anti-inflammatory protein-coding genes like *CD200* (ref. 32) and *BATF3* (ref. 33) were repressed in HBV vaccine hyporesponders (Fig. 6). Collectively our results highlight the importance of type I interferon and other pro-inflammatory pathways in the development of a poor response to HBV vaccine.

Poor-responders to HBV vaccine showed higher numbers of RBCs than responders. Interestingly genes and pathways that correlate positively with HBV Ab titres including genes within module M1 were negatively correlated with RBC counts. Conversely genes included in module M16 (Fig. 6) and pro-inflammatory genes included in pathways enriched in poor-responders (Fig. 5e) were positive correlates of RBC counts. The HIF-1 α pathway. which controls erythropoietin expression, was significantly upregulated in poor-responders (Fisher's exact test: *P*=0.0360, Supplementary Table 4c); target genes of HIF-1α were induced in poor-responders and their expression levels correlated with higher RBC counts (Pearson's *t*-test: *P*=0.0462, Fig. 5e). RBC counts were positively correlated to the *HMOX1* (haem oxidase catalysing the degradation of haem), *HP* (scavenger of free Hb) and *CD163* expressions (scavenger receptor of the Hb–HP complex), all of which are interferon-regulated genes and were induced in HBV vaccine poor-responders (Supplementary Table 4a). The haem-induced response pathway was significantly enriched among genes differentially expressed between HBV vaccine responders and poor-responders (Fisher's exact test: *P*=0.0461, Supplementary Table 4c). *EIF2AK1* (Haem-Regulated Inhibitor) and several of its downstream targets (for example, *EIF2, EIF3 and EIF4*) were negatively associated with Ab titres (Supplementary Fig. 6a). These results highlight a potential role for the haem-induced response and for hypoxia in the poor response to the HBV vaccine.

These results suggest a mechanism whereby this aging-associated inflammation leads to induction of the HIF-1α pathway (Supplementary Table 4c) and other pro-inflammatory effector molecules. This may upregulate erythropoietin, a known target of HIF-1α, leading to increased RBC counts, release of cell free haemoglobin as suggested from the heightened levels of *HP* mRNA^34^ and the downstream upregulation of HRI (Supplementary Fig. 6a), a trigger of type I interferon production by pDC and other innate immune cells (Supplementary Fig. 7). This will further enhance inflammatory pathways that downregulate B-cell activation and hence hyporesponse to *de novo* vaccination with a vaccine such as HBV (Supplementary Fig. 7). Alternatively, these perturbations in RBC homeostasis and B-cell response could be the mark of a global hyperimmune inflammation observed in elderly (‘inflammaging')^35^. The interplay between inflammation and B-cell signalling is supported by all the experimental approaches in this study (Supplementary Fig. 7).

Several strategies may help overcome the mechanisms impeding optimal response to vaccines. Subjects having a pro-inflammatory signature prior to vaccination could benefit from vaccine regimens conferring improved immunogenicity. For example, inflammatory bowel disease patients that did not respond to a first HBV vaccine course were more likely to mount an antibody response on receiving a second complete immunization schedule^36^. Strategies used to overcome desensitization of the innate immune response including the use of more potent adjuvants^37^ or anti-inflammatory drugs could also improve immune responses to HBV vaccination in the elderly. For example, the anti-inflammatory drug rapamycin improved Ab responses to influenza vaccination^38^ when used at low dose prior to vaccination. Our results show that different ‘inflammatory' pathways are at cross-purposes; these differential effects of inflammation will need to be further clarified to improve the response to vaccines. Accordingly, the predictive gene-expression signatures described herein might allow tailoring of vaccine regimens to older persons predisposed to VHR.

## Methods

### Study design and conduct

This was an observational open-label study (Fig. 1a), performed between July 2010 and November 2011. This study was registered on ClinicalTrials.gov (NCT01119703), was overseen by Institutional Review Board Services (Ontario, Canada) and was conducted according to cGCP and applicable laws.

One-hundred and seventy-four generally healthy, HBV -naive, adult residents of Québec were divided into two groups to participate in this study. One group was aged ≥65 (*n*=144); participants in the other group were aged between 25 and 40 years (*n*=30). A demographic summary appears in Supplementary Table 1. All participants provided written informed consent.

All participants were vaccinated with two doses of Twinrix (HBsAg and HAV virus—Glaxo Smith-Kline), and single doses of generic Tetanus-Diphtheria booster (Tetanus and Diphtheria—Sanofi Pasteur) and Dukoral (recombinant Cholera toxin B subunit and whole killed Vibrios—Sanofi Pasteur) according to the respective product labels. Intramuscular vaccines were administered to opposite arms and all doses of Twinrix were administered in the same arm. HBV titres were collected after the second injection based on published results, suggesting that all three injections would result in very high rates of seroresponse^39^,^40^. As we desired a wide spectrum of responses against which to regress biomarker results, titre responses were only recorded after two injections—leading to an unexpectedly large number of poor-responders. The observed range of titre responses sufficed for the required discovery effort.

### Antibody titres

Anti-Cholera toxin B subunit (CTB) IgG were quantified in serum samples according to a GM1-enzyme-linked immunosorbent assay (ELISA) method adapted from Svennerholm *et al.*^41^. GM1 is a membrane ganglioside which is found in cell membranes of the gastro-intestinal tract and that acts as a receptor for CTB. The GM1 ganglioside was used in the ELISA assay to increase binding of CTB to the plate wells. Briefly, high protein-binding microplates were first coated overnight with GM1 (1.5 μM in PBS). Plates were washed with PBS and were blocked with a 0.5% BSA solution for 1 h at room temperature. The plates were then coated with CTB (1 μg ml^−1^ in 0.5% BSA) for 1 h at room temperature and washed. Serum samples were serially diluted (six serial dilutions) with a 0.5% BSA+Tween solution. Diluted sera were added in duplicates to the blocked GM1/CTB-coated plate and incubated for 2 h at room temperature. A positive control serum was also included on each assay plate and was assayed at six serial dilutions, in duplicate. After successive washes to remove unbound antibodies, a goat anti-human IgG antibody conjugated to horseradish peroxidase was added and incubated for 1 h at room temperature. Unbound conjugate was removed by washing, and a tetramethylbenzidine (TMB) substrate solution was added. Following a 10-min incubation period the stopping solution was added and optical density (OD) was measured at 450/620 nm using a spectrophotometer. Titration curves were drawn by plotting averaged OD values versus the dilution factor and fitted using a five-parameter curve fit. The antibody titre was defined as the midpoint (or IC50) of the titration curve. Titres were normalized against the positive control serum titre.

Anti-hepatitis B surface antigen (anti-HBsAg) total antibodies were quantified in serum samples using the MONOLISA Anti-HBs PLUS kit (Bio-Rad catalogue number 72566). Briefly, serum samples were diluted at the recommended dilution factor in the dilution buffer provided by the kit. Diluted serum samples as well as calibration standards and QC high and QC low controls were added to HBsAg-coated plates, and incubated for 60 min at 37 °C. After a washing step, the conjugate was added to the plates and incubated for another 60 min at 37 °C. The plates were washed and the TMB substrate solution was added. Following a 30-min incubation period the stopping solution was added and OD was measured at 450/620 nm using a spectrophotometer. The determination of anti-HBs levels has been standardized by use of the WHO Anti-HBs reference preparation expressed in mIU ml^−1^. Though HBV titres were measured 1 month after the second dose, all patients were offered the third dose to conform to the approved regimen.

Anti-Diphtheria IgG were quantified in serum samples using a commercially available kit (Sekisui Virotech, catalogue number EC129.00). Briefly, serum samples were diluted at the recommended dilution factor in the dilution buffer provided by the kit. Diluted serum samples as well as calibration standards and IgG high and IgG low controls were added to the antigen-coated microplates and incubated for 30 min at 37 °C. After a washing step, the anti-IgG HRP conjugate was added to the plates and incubated for another 30 min at 37 °C. The plates were washed and the TMB substrate solution was added. Following a 30-min incubation period, the stopping solution was added and OD was measured at 450/620 nm using a spectrophotometer. The Anti-Diphtheria toxin IgG concentrations were expressed in IU ml^−1^ following the WHO Standard. The standard curve of the Diphtheria ELISA has been verified using the Diphtheria Antitoxin Human Serum (00/496) of the Institute for Biological Standards and Control, WHO International Laboratory for Biological Standards in Great Britain.

Anti-Tetanus IgG were quantified using a commercially available kit (Sekisui Virotech, catalogue number EC124.00). Briefly, serum samples were diluted at the recommended dilution factor in the dilution buffer provided by the kit. Diluted serum samples as well as calibration standards and IgG high and IgG low controls were added to the antigen-coated microplates and incubated for 30 min at 37 °C. After a washing step, the anti-IgG HRP conjugate was added to the plates and incubated for another 30 min at 37 °C. The plates were washed and the TMB substrate solution was added. Following a 30-min incubation period, the stopping solution was added and OD was measured at 450/620 nm using a spectrophotometer. The Anti-Tetanus toxin IgG concentrations were expressed in IU ml^−1^ following the WHO Standards. The standard curve of the Tetanus ELISA has been verified using the international standard of the WHO for human Tetanus immunoglobulin (TE-3).

### Development of the BioAge signature

The Gene Expression Omnibus and Arrays Express databases were searched for the largest publically available microarray data set studying blood samples and including age as clinical endpoint. The SAFHS comprising of 1,240 samples hybridized on Illumina Sentrix Human Whole Genome 6 BeadChips^19^ was used to train the BioAge signature. Raw files containing background-subtracted intensities, chip annotation and sample annotation were downloaded from ArrayExpress database with accession number E-TABM-305. Analysis of the raw data was conducted using R/Bioconductor software^42^. Missing values (corresponding to <1% of all the intensity values) were imputed using nearest neighbour averaging method^43^. Quantile normalization was performed followed by log2 transformation for variance stabilization. Background value of 0.1 was used for surrogate replacement prior to log2 transformation to prevent infinite intensities values.

The LIMMA package^44^ was used to fit a linear regression model (1) to each probe where the log2 expression of transcript (*x*) was used as an independent variable and chronological age as a dependent variable (age). Age was kept as a continuous variable ranging from 15 to 94 years.


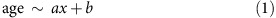


For each regression model, a (moderated) *t*-test was performed to test the significance of the association of expression of the transcript and the chronological age. The *P* values were adjusted for multiple comparisons using the Benjamini–Hochberg method^45^. A *P* value, corrected for false-positive rate, ≤0.05 was used as a cutoff to identify transcripts significantly associated with chronological age. About 2,285 transcripts passed this specified cutoff (Supplementary Table 3a). Once the genes correlated with age were identified (LIMMA: adjusted *P*≤0.05), we evaluate in how many sets of correlates genes (*i.e*. modules) they could be separated. To that end, the log2 expression was transformed to *z*-score (that is, for each transcript its average expression was subtracted and divided by its s.d. across samples). The optimal number of modules (*k*) was estimated via the gap statistic^46^ (Supplementary Fig. 2a). The functional annotation of each module in terms of representative pathways is given in Supplementary Table 3b.

We then defined the BioAge signature as two sets of modules (M): up arm with modules overexpressed in elderly (M12 to M20) and down arm with modules overexpressed in the non-elderly (M1 to M11). The BioAge score equation (2) is defined as the difference between the average gene expression (*μ*) of modules in the up arm minus the average gene expression of modules in the down arm.


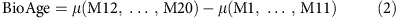


### Establishing gene-expression signature predictive of HBV vaccine response

RNA was isolated from PAXgene blood samples according to the manufacturer's instructions. Isolated total RNA samples were assayed for quality metrics (Agilent Bioanalyzer) prior to amplification. Three-hundred and thirty-five samples passing quality control were then amplified using the NuGEN amplification protocol and hybridized to Affymetrix HuRSTA-2a520709 chips (Affymetrix, Santa Clara, CA). The chips contain 60,607 probe sets representing 22,580 unique genes (GEO: GPL15048).

Analysis of the CEL files was conducted using R/Bioconductor software packages^42^. The expression data were normalized using the Robust Multi-array Average (RMA) method^47^. Five technical duplicates were included in the microarray experiments; each pair of technical duplicates was averaged after the microarrays normalization.

The elderly data set was separated randomly into a training set composed of 2/3 (91 samples) of the samples and a test set composed of 1/3 (47 samples) of the samples (Supplementary Table 1). Samples from subjects under 50 (*n*=55) years were excluded from the data set. The randomization of the remaining samples (*n*=280) was blocked by patients' age and gender (that is, controlling that the proportion of male/female and the age range are similar between training set and test set). To identify pre-vaccination biomarkers of vaccine response, the data set was restricted to pre-vaccination samples (138/280 samples). The resulting training set and test set contained 91 and 47 samples, respectively (Supplementary Table 1). Mathematical modelling of the anti-HBsAg titres performed using kernel density estimation as implemented in the function density of R package stats revealed a bimodal distribution of the titres. Participants who had anti-HBsAg titres 1 month after the second immunization below detection threshold (<5 mIU ml^−1^) were considered poor-responders to the HBV vaccine (*n*=52), while participants with anti-HBsAg titres ≥5 mIU ml^−1^ were considered responders to the HBV vaccine (*n*=38). The R package LIMMA was used to fit a linear model to each probe and to perform (moderated) *t*-tests on the comparison HBV vaccine responders versus poor-responders. The *P* values were adjusted for multiple comparisons using the Benjamini–Hochberg method^45^. A naive Bayes classifier was built on the training set based on the expression of top differentially expressed transcripts (ordered by LIMMA moderated *t*-test *P*-values). The number of features in the naive Bayes classifier was established by performing 10-fold cross-validation on the EM131 training set. The resulting classifier was based on the expression of the top 15 differentially expressed transcripts between HBV vaccine responders and poor-responders. ROC analysis and permutation test were performed on EM131 test set to evaluate the performance of the classifier. A 10,000 fold permutation test was performed to assess the probability of obtaining an equal or superior classifier performance.

### Pathway enrichment analysis

Fisher's exact test was used to assess significance of the intersect between the modules of genes composing the BioAge signature and *a priori* built gene sets (Ingenuity canonical pathways). Significantly enriched pathways were organized in sets of related pathways using the enrichment map strategy^48^. Overlap between significant gene sets was computed according to the Jaccard Index. A Jaccard index cutoff of 0.25 was used to generate the table presented in Supplementary Tables 3b and 4c.

### Statistical analysis

Clinical characteristics including age, gender, height, weight, body mass index and pre-vaccination cytomegalovirus antibody titres were tested as predictors of HBV vaccine response using univariate and multivariate logistic regression analysis. *Z*-tests were used to test whether the regression coefficient of each predictor was different from zero. Statistical tests were two-sided, adjusted for multiple testing using the Benjamini–Hochberg correction and considered significant when the adjusted *P* value was ≤0.05.

### Polychromatic FCM

Phenotypic analysis of T-cell and innate cell subsets was performed by staining whole blood collected in EDTA Vacutainer tubes. Blood for B-cell analysis was collected in Na-Heparin Vacutainer tubes and further processed by density gradient centrifugation through Ficoll to obtain peripheral blood mononuclear cells (PBMC) prior to performing the following steps. Processing and staining steps for cytometry were performed within 8 h of blood draw. Briefly, 50 μl of whole blood was added to TruCount tubes (Becton-Dickinson) for the phenotypic analysis and absolute enumeration of cellular subsets per microliter of blood. Following 30-min incubation at room temperature with surface staining antibodies, BD FACSLyze was added for 10 min to lyse RBCs. Cells were then washed and transferred to a 96-well plate for cytometric data acquisition. A similar procedure was implemented for the phenotypic analysis of innate cell subsets. However, given the lower frequencies of target populations, 100 μl of whole blood was processed and stained prior to analysing by FCM. An example of gating strategies for B-cell populations is presented in Supplementary Fig. 5a.

Three FCM panels (Supplementary Table 5) were respectively used to determine frequencies of T cells, B cells and innate immune cells obtained from the 174 pre-vaccination (Visit 2) samples of the study cohort. Logistic regression was used to assess the association between individual FCM markers and the response to HBV vaccine on the EM131 training set. A multivariate model combining the best markers (*z*-test: *P*≤0.1) was built using the forward selection approach. Briefly, the forward selection process starts with no variable in the model and tests the addition of each variable using the Akaike information criterion. Only those variables that provide a superior improvement of the model compared with the remaining variable are selected in the final model. The best model obtained included four FCM markers. ROC analysis was performed on EM131 test set to evaluate the performance of the multivariate model.

### Cytokine measurements

A panel of 45 cytokines/chemokines was measured on the 174 pre-vaccination (V2) samples of the EM131 cohort. Samples were assayed to determine the concentration of inflammatory proteins according to multianalyte profiling protocols established by Rules-Based Medicine (RBM) (Austin, TX). Serum samples were shipped to RBM for analysis using the multiplex ELISA protocol Human Inflammation multianalyte profiling (Supplementary Table 5b). Logistic regression was used to assess the association between individual cytokine markers and the response to the HBV vaccine and a multivariate model combining the best markers was built on the EM131 training set using a forward selection approach, as described above. The best model obtained included three cytokines. ROC analysis was performed on EM131 test set to evaluate the performance of the multivariate model.

### Haematologic measurements

A panel of 3 standard haematologic markers (complete blood count) was measured on the 174 pre-vaccination samples taken 28 days prior to vaccination (V1) of the EM131 cohort. Logistic regression was used to assess the association between individual haematologic markers and the response to HBV vaccine and a multivariate model combining the best markers was built on the EM131 training set and tested on the EM131 test set.

### Integrative analysis of all multi-dimensional data sets

For every pair of omics data, the one containing the most features was used as the independent variable and the other omics was used as dependent variable. A partial least-square regression as implemented by the function spls of the R package mixOmics was used to project the omics data in the same space^26^. Once the two omics are projected in the same space, a Pearson's correlation between features of the first and second omics are calculated. All absolute Pearson's correlations |*r*|≥0.188 corresponding to a *t*-test *P* value <0.05 were used to generate a correlation network between omics.

### Code availability

All the source codes are available at https://sites.google.com/a/case.edu/fouslim/publication/em131.

## Additional information

**Accession codes:** The microarray data have been submitted to the National Center for Biotechnology Information Gene Expression Omnibus (http://www.ncbi.nlm.nih.gov/geo) under accession number GEO: GSE65834.

**How to cite this article:** Fourati, S. *et al.* Pre-vaccination inflammation and B-cell signalling predict age-related hyporesponse to hepatitis B vaccination. *Nat. Commun.* 7:10369 doi: 10.1038/ncomms10369 (2016).

## Supplementary Material

Supplementary Information Supplementary Figures 1-7 and Supplementary Tables 1-5

## Figures and Tables

**Table 1:**
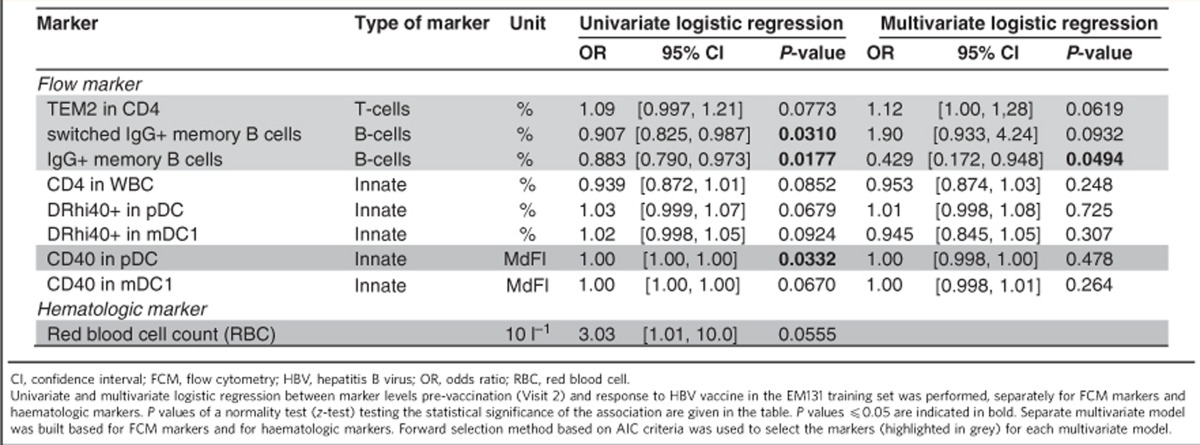
Identification of FCM markers and haematologic markers associated with response to HBV vaccine.
