# Supplementary material for: Pre-vaccination inflammation and B-cell signalling predict age-related hyporesponse to hepatitis B vaccination
Source: Nat Commun. 2016 Jan 8;7:10369. doi: 10.1038/ncomms10369 (PMC4729923; doi:10.1038/ncomms10369)
Supplement: Supplementary Information — Supplementary Figures 1-7 and Supplementary Tables 1-5 [file ncomms10369-s1.pdf]

a

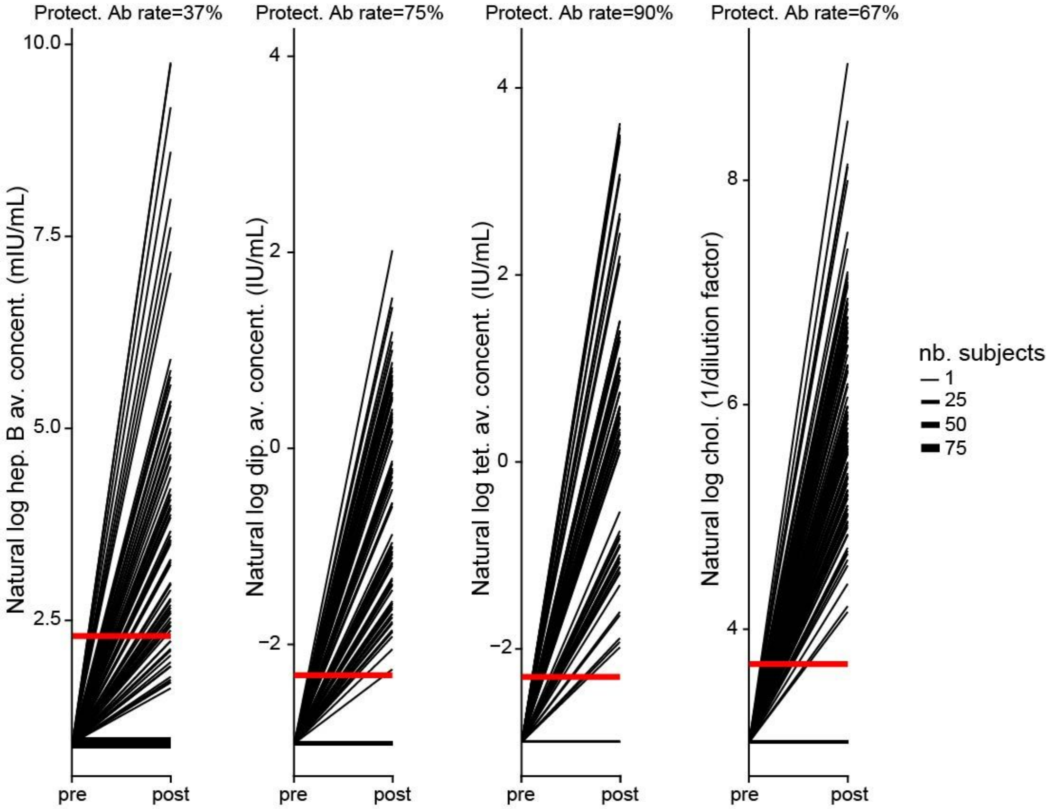

b

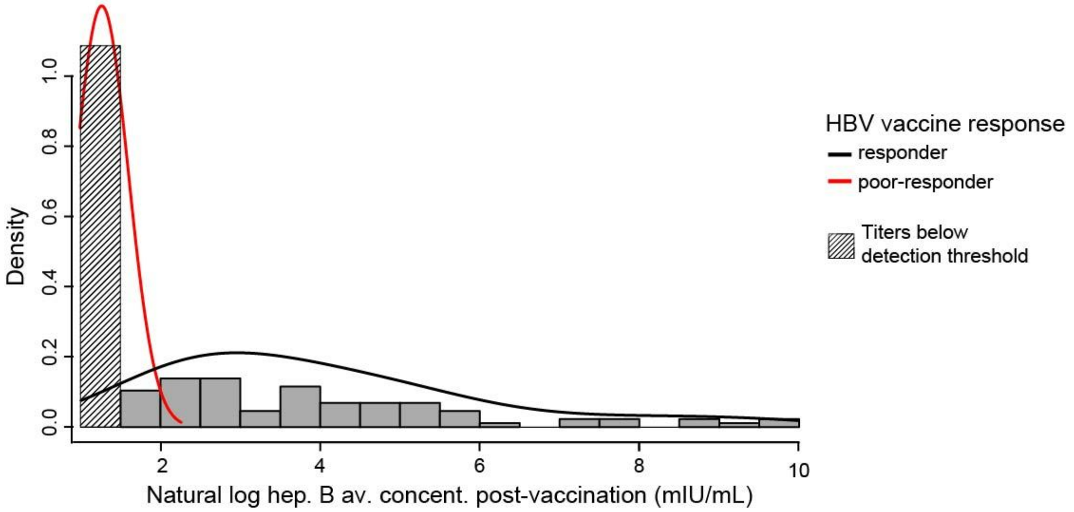

Supplementary Figure 1. Two groups of responders to the HBV vaccine can be identified based on the antibody response titers

(a) Response plots showing antibody titers as function of the vaccination status (x-axis: before and after receiving the vaccines). The analysis was restricted to subjects with undetectable titers pre-vaccination (hep. B: n= 170, dip: n=105, tet: n=67 and chol: n=151). The titers for HBsAg (hep. B), diphtheria toxin (dip.), tetanus toxin (tet.) and cholera toxin (chol.) are given on natural log scale. The unit of every titer is given in the y-axis label. Red horizontal lines indicated standard titer cutoffs used to define protective antibody levels. The percentage of patients having antibody titers above the protective thresholds (Protect. Ab) post-vaccination is indicated above each plot. (b) Density of the anti-HBsAg levels detected post-vaccination. Ab titers are given in mIU/mL in natural log scale space. 94 of the 174 subjects (54%) present Ab titers below the detection threshold of 5 mIU/mL while 79 subjects presented Ab titers ranging between 5.07 to 17,311.4 mIU/mL. Kernel density estimation of the Ab titers revealed two groups of responders based on Ab titers, HBV vaccine poor-responders (red line) with Ab titers below the detection threshold and HBV vaccine responders (black line) with Ab titers above 5 mIU/mL.

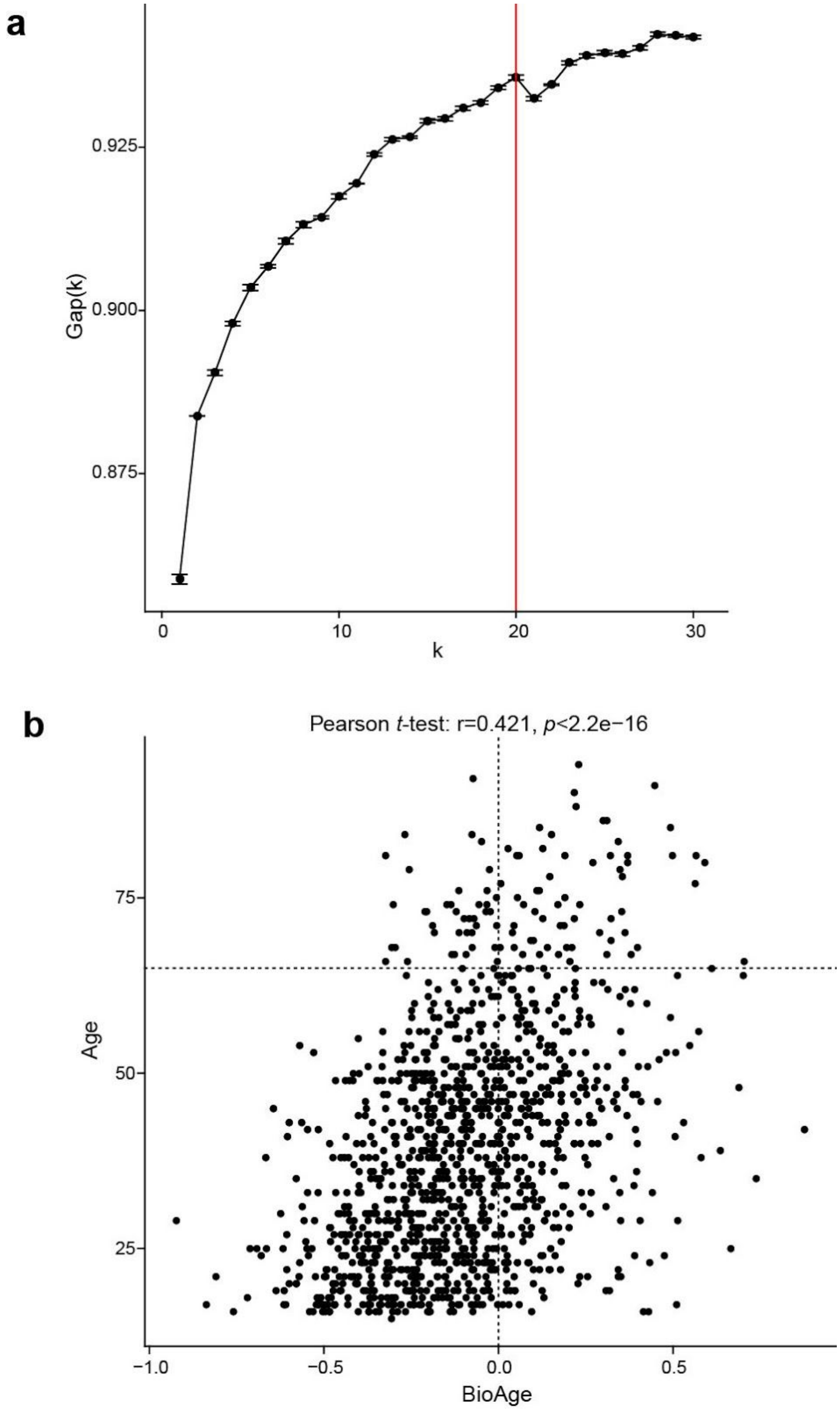

Supplementary Figure 2. Development of the BioAge signature on the SAFHS cohort

(a) Scatter plot showing the gap statistic estimation of the optimal number of cluster of genes among the 2285 genes correlated to age on the SAFHS cohort. The x-axis corresponds to the different number of clusters tested and the y-axis corresponds to the gap statistic (Gap) and its 95% confidence interval. The gap statistic corresponds to the between-clusters variance divided by the intra-cluster variance (the greater the gap statistic the better the fit). The optimal number of clusters was obtained following the rule described in Tibshirani *et al.* defined as the smallest k such that $Gap(k) \geq Gap(k+1) - sd(k+1)$. Consequently, twenty clusters were identified as the optimal number of clusters of genes. (b) Scatter plot showing the chronological age as a function of the BioAge on the SAFHS dataset. Among the 109 donors aged 65 or over (which is the population with the same age range as the elderly in EM131), 42 (39%) have a young BioAge (BioAge < 0) while 67 (61%) have an old BioAge (BioAge ≥ 0).

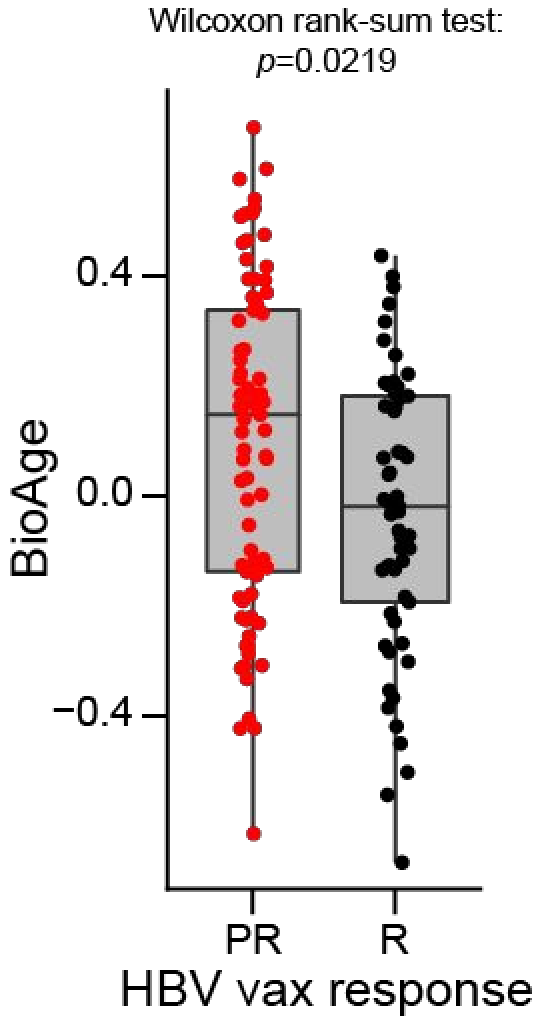

Supplementary Figure 3. Boxplot showing the BioAge score as a function of HBV vaccine response on the EM131 elderly cohort

A Wilcoxon rank-sum test was used to test for statistical difference in BioAge between HBV vaccine responders (R) and poor-responders (PR), the *p*-value is presented above the boxplot.

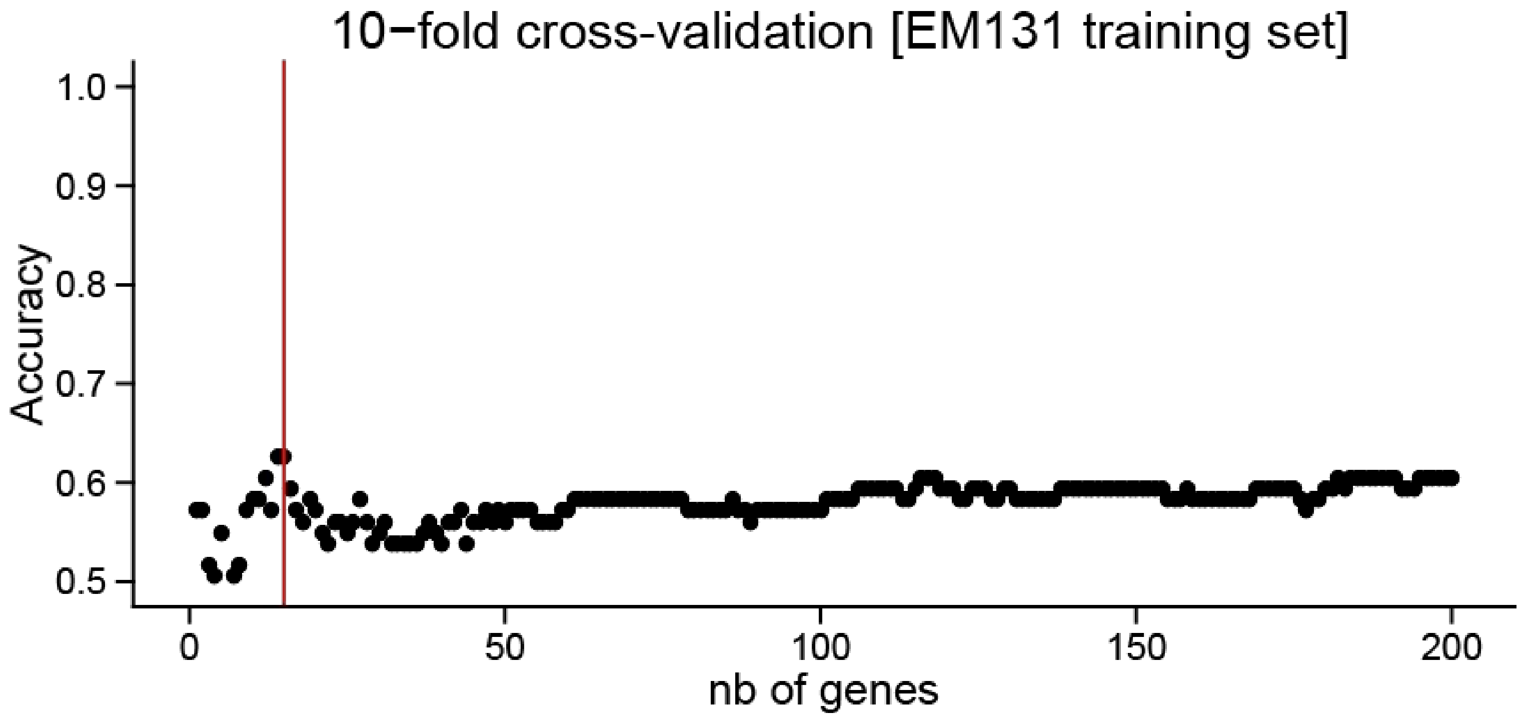

Supplementary Figure 4. Scatter plot showing the results of the 10-fold cross-validation on the EM131 training set.

The red line corresponds to naïve Bayes classifier of 15 genes, which gave an estimated accuracy of 62.6% accuracy.

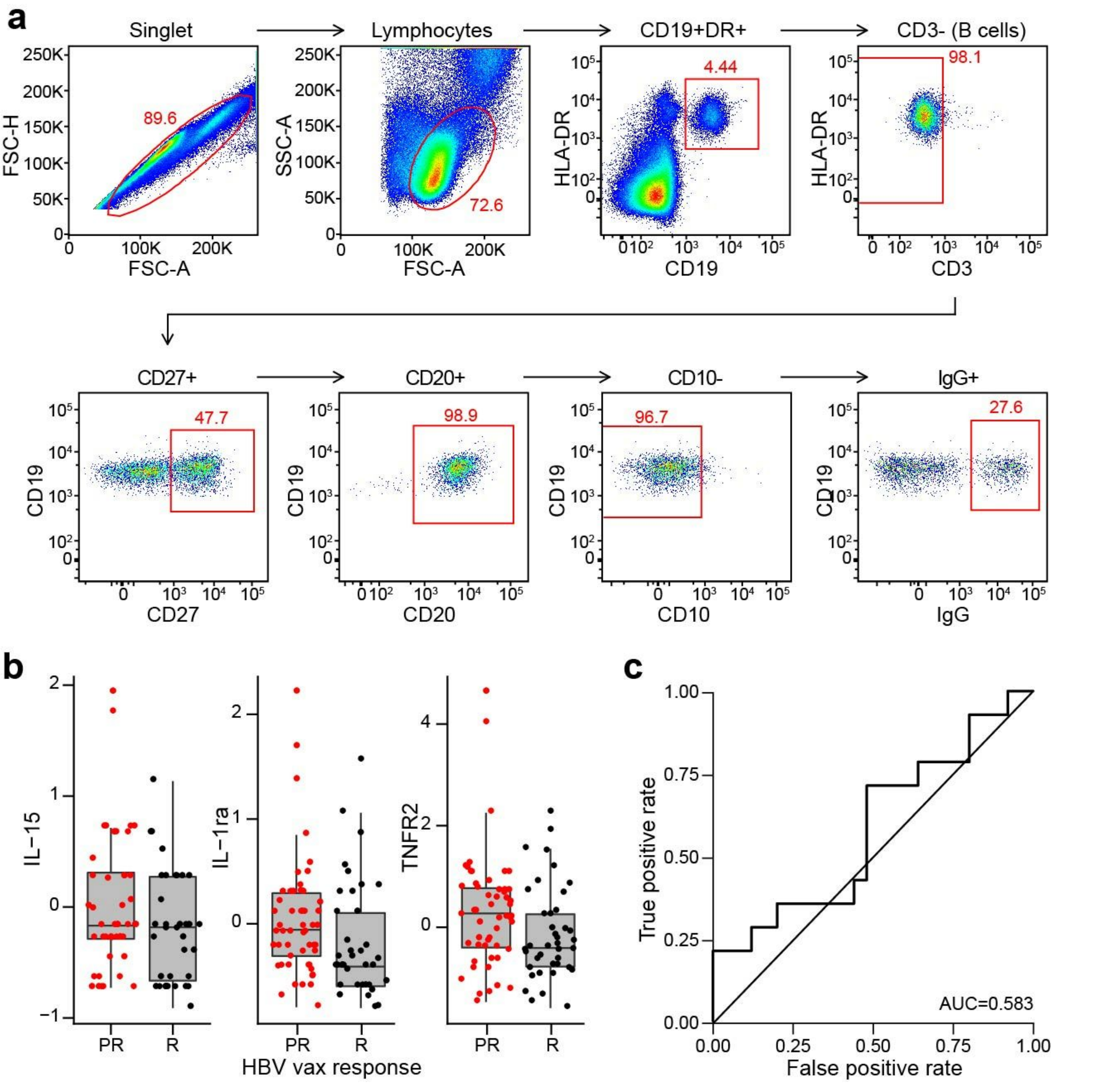

Supplementary Figure 5. FCM and cytokines associated with response to the HBV vaccine on the EM131 training set

(a) Examples of our gating procedure for obtaining the percentage of memory IgG+ B cell populations (CD3-CD19+HLA-DR+CD20+CD27+CD10-IgG+) among total B cells (CD3-CD19+HLA-DR+) for subject 2087 at visit 2 (value=12.6%). Please see **Supplementary Table 5a** for cell population annotations. (b-c) Cytokine-expression was analyzed in order to identify markers of response to the HBV vaccine. (b) Boxplots presenting the levels of three cytokines selected in the forward selection model between responders and poor-responders to the HBV vaccine on the EM131 training set. The three cytokines selected are implicated in the inflammatory mechanism. (c) ROC curves for the prediction of the HBV vaccine response using the cytokine data on the EM131 test set.

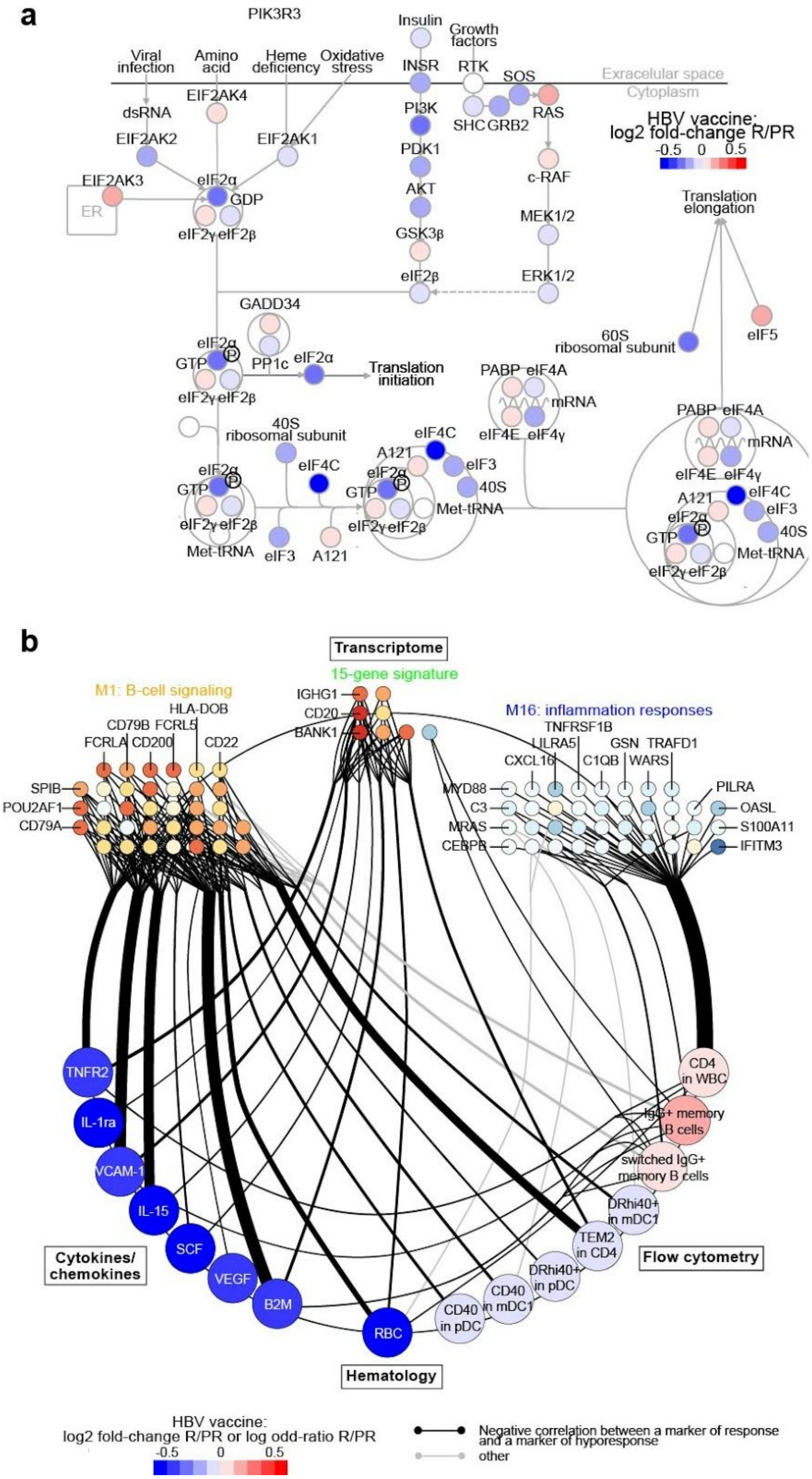

Supplementary Figure 6. Integrative analysis reveals negative correlations between biomarkers of response and hyporesponse to the HBV vaccine

(a) EIF2 signaling pathway is associated with hyporesponse to the HBV vaccine**.** Ingenuity EIF2 canonical pathway where nodes are colored by the fold-change difference between HBV vaccine responders (R) compared to poor-responders (PR) as calculated on the EM131 training set. (b) Least square regressions between pairs of “omics” was performed using the R package “mixOmics”. The results regression coefficients were converted to Pearson correlation between pairs of features of the different “omics”. Significant (*t*-test: $p \leq 0.05$) negatives correlations are presented as edges and the features as vertices. The vertices are colored by fold-change between HBV vaccine responders vs. poor-responders of the EM131 training set.

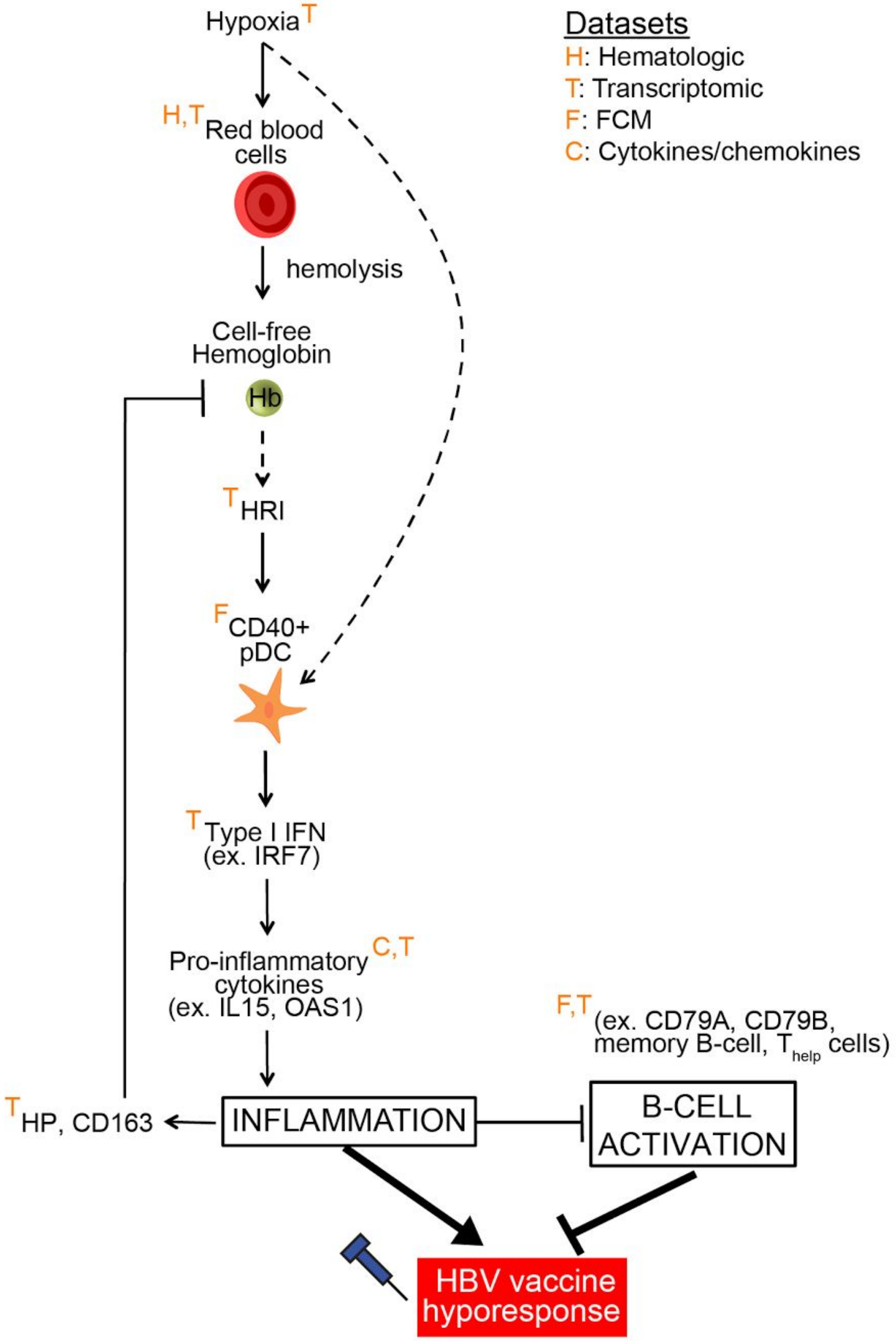

Supplementary Figure 7. Proposed mechanism leading to hyporesponse to HBV vaccine

Hypoxia (HIF-1α signaling pathway) leads to the increase in red blood cells. Turnover of red blood cells will release free-hemoglobin in the circulation. Hemoglobin, a source of heme-complex, will trigger the activation of the heme-regulated eIF2α kinase (HRI), In turn, HRI will stimulate the production of type I interferon by pDCs and promote the release of pro-inflammatory cytokines in the system. The resulting inflammatory response will hinder response to HBV vaccination. A negative feedback loop involving haptoglobin (HP) and CD163 will be activated to catalyze the degradation of cell-free hemoglobin. In parallel, the absence of B-cell activation or the low frequency of memory B-cell will result in hyporesponse to HBV vaccine. The datasets (transcriptomic, FCM, cytokines/chemokines and hematologic) supporting the elements of the proposed mechanism are indicated on the figure.

Supplementary Table 1 Clinical characteristics of the study cohort

	Complete data set	Training set	Test set	Fisher's exact test/ Wilcoxon test p-value
n	174	95	49	
Age				
range	[25, 83]	[65, 81]	[65, 83]	0.896
[25-40]	30	0	0	0.810
[65-75[	122	81	41	
[75-83]	22	14	8	
Gender				
male	79	44	23	1.00
female	95	51	26	
Race				
white	174	95	49	N/A
Height (cm)				
range	[139, 190]	[144, 182]	[139, 181]	0.666
Weight (kg)				
range	[40.3, 112.4]	[47.7, 102.5]	[40.3, 94.5]	0.634
BMI (kg/m2)				
range	[17.0, 34.9]	[21.5, 33.9]	[17.0, 33.5]	0.452
< 30	140	78	41	0.813
≥ 30	32	16	7	
N/A	2	1	1	
CMV at V2 (IU/mL)				
< 0.75	94	49	24	0.861
≥ 0.75	80	46	25	
HepB at V2 (mIU/mL)				
< 5	171	94	48	N/A
≥ 5	3	1	1	
HepB at V5 (mIU/mL)				
< 5	94	54	30	0.390
≥ 5	79	41	19	
NA	1	0	1	
Cholera at V2 (1/dilution factor)				
< 40	152	83	43	1.00
≥ 40	22	12	6	
Cholera at V4 (1/dilution factor)				
< 40	50	25	13	1.00
≥ 40	123	70	35	
N/A	1	0	1	
Dip at V2 (IU/mL)				
range	]0, 5.72]	]0, 5.72]	]0, 1.61]	0.401
Dip at V4 (IU/mL)				
range	]0, 51.1]	]0, 24.3]	]0, 51.1]	0.377
Tet at V2 (IU/mL)				
range	]0, 24.9]	]0, 19.4]	]0, 24.9]	0.628
Tet at V4 (IU/mL)				
range	]0, 71.3]	]0, 71.3]	]0, 41.6]	0.696

All participants were of European descent with BMI below 35 kg/m^2; ~55% were women; and none had histories of autoimmunity, immunomodulating medications, cancer, recent vaccination or recent infection. The Fisher's exact test (categorical variable) and the Wilcoxon Rank sum test (continuous variable) were used to assess difference between the EM131 training set and EM131 test set. No significant difference in term of clinical (BMI: body mass index) or serological (HepB: hepatitis B, Dip: diphtheria, Tet: tetanus) variables was observed between those two sets.

Supplementary Table 2 Logistic regression with poor-response to HBV vaccination

	Univarite			Multivariate		
	OR	95% CI	*p*-value	OR	95% CI	*p*-value
Age ([65, 83] *v* [25, 65[)	2.80	[1.24, 6.69]	**0.0158**	1.52	[0.593, 4.03]	0.385
Sex (male *v* female)	2.80	[1.50, 5.33]	**0.00142**	2.44	[1.26, 4.80]	**0.00847**
Height (per cm)	1.03	[0.994, 1.07]	0.105	---	---	---
Weight (per kg)	1.02	[0.998, 1.05]	0.0832	---	---	---
BMI (per kg/m2)	1.06	[0.959, 1.17]	0.272	---	---	---
CMV (≥0.75 *v* <0.75 IU/ml)	1.26	[0.688, 2.33]	0.453	---	---	---
BioAge (per unit)	5.62	[1.82, 18.6]	**0.00343**	4.14	[1.21, 15.2]	**0.0270**

Univariate and Multivariate logistic regression against response to HBV vaccine was performed using clinical variables and the BioAge signature. Only variables presenting a significant association with response to HBV vaccine at the univariate level (z-test: *p*-value ≤ 0.05) were included in the multivariate level. Age, gender and the BioAge are significantly associated with HBV vaccine response in the univariate analysis. The BioAge signature maintains significant association with HBV vaccine response even after correcting for clinical variable like age and gender.

Supplementary Table 3a. List of transcript in the BioAge signature

Probe ID	Gene Symbol	Module	Pearson Correlation	Regression Coefficient	t-statistic	nominal p-value	adjusted p-value
GI_4507398-S	TCF4	1	-0.310	-0.00829	-11.4	8.98E-29	3.03E-25
GI_24475968-S	MZB1	1	-0.285	-0.0142	-10.5	1.27E-24	2.15E-21
GI_31542536-S	CDCA7L	1	-0.248	-0.00941	-9.00	8.12E-19	6.51E-16
GI_13375806-S	BEND5	1	-0.232	-0.0160	-8.38	1.39E-16	7.72E-14
GI_32189367-S	IGJ	1	-0.218	-0.0138	-7.85	8.93E-15	3.80E-12
Hs.449609-S	---	1	-0.216	-0.0149	-7.80	1.28E-14	5.31E-12
GI_23238191-S	TNFRSF17	1	-0.216	-0.0194	-7.78	1.55E-14	6.20E-12
GI_38348195-S	DERL3	1	-0.207	-0.0146	-7.46	1.67E-13	5.59E-11
Hs.449584-S	---	1	-0.200	-0.0236	-7.19	1.15E-12	3.22E-10
GI_20336304-I	BCL11A	1	-0.196	-0.00621	-7.03	3.36E-12	8.40E-10
GI_41190507-S	---	1	-0.186	-0.0123	-6.64	4.58E-11	9.30E-09
GI_40317614-A	PMEPA1	1	-0.185	-0.0247	-6.63	4.99E-11	1.00E-08
GI_41190543-S	---	1	-0.184	-0.0169	-6.57	7.16E-11	1.37E-08
Hs.449602-S	IGLL5	1	-0.183	-0.0120	-6.57	7.58E-11	1.43E-08
GI_21361744-S	STRBP	1	-0.183	-0.00643	-6.54	9.24E-11	1.72E-08
GI_40804747-S	LIMS2	1	-0.180	-0.0113	-6.45	1.62E-10	2.93E-08
Hs.512137-S	---	1	-0.180	-0.0126	-6.43	1.84E-10	3.25E-08
GI_12669916-S	E2F5	1	-0.179	-0.00752	-6.40	2.19E-10	3.77E-08
Hs.205675-S	---	1	-0.176	-0.0106	-6.28	4.66E-10	7.42E-08
Hs.16515-S	---	1	-0.169	-0.00809	-6.01	2.40E-09	3.39E-07
hmm11123-S	---	1	-0.168	-0.0238	-6.01	2.50E-09	3.50E-07
Hs.512124-S	---	1	-0.165	-0.00928	-5.88	5.30E-09	6.79E-07
hmm10298-S	---	1	-0.165	-0.0129	-5.88	5.32E-09	6.80E-07
GI_40254976-S	MIR600HG	1	-0.160	-0.0102	-5.72	1.34E-08	1.61E-06
GI_21264573-S	TSPAN13	1	-0.159	-0.00968	-5.68	1.72E-08	1.99E-06
GI_21536441-S	BCL7A	1	-0.159	-0.0125	-5.66	1.87E-08	2.13E-06
GI_41350329-S	MOB3B	1	-0.159	-0.00854	-5.66	1.92E-08	2.17E-06
GI_37546969-S	---	1	-0.156	-0.0117	-5.54	3.59E-08	3.90E-06
GI_31542945-S	---	1	-0.156	-0.00399	-5.53	3.85E-08	4.16E-06
GI_7019342-S	CD24	1	-0.155	-0.0112	-5.53	3.95E-08	4.24E-06
GI_4507176-S	SPIB	1	-0.153	-0.00717	-5.45	6.00E-08	6.21E-06
GI_41281541-S	COBLL1	1	-0.151	-0.0154	-5.37	9.42E-08	9.37E-06
GI_42662613-S	---	1	-0.149	-0.00893	-5.31	1.27E-07	1.21E-05
GI_5453933-S	POU2AF1	1	-0.148	-0.00975	-5.25	1.76E-07	1.64E-05
GI_18641377-S	HLA-DOB	1	-0.144	-0.00797	-5.11	3.73E-07	3.19E-05
GI_7661757-S	KIAA0125	1	-0.143	-0.00580	-5.09	4.06E-07	3.43E-05
Hs.406489-S	---	1	-0.140	-0.0129	-4.99	6.82E-07	5.44E-05
GI_27500987-S	LINC00494	1	-0.137	-0.0198	-4.87	1.24E-06	9.09E-05
GI_21040361-A	BACE2	1	-0.137	-0.00735	-4.85	1.38E-06	0.000100
Hs.449605-S	---	1	-0.136	-0.00930	-4.85	1.42E-06	0.000103
GI_4502650-S	CD22	1	-0.136	-0.00678	-4.82	1.59E-06	0.000112
Hs.188173-S	---	1	-0.134	-0.00872	-4.76	2.12E-06	0.000145
GI_23110986-A	CD20	1	-0.134	-0.00731	-4.75	2.27E-06	0.000154
GI_32484980-S	BANK1	1	-0.132	-0.00595	-4.69	3.08E-06	0.000203
GI_6912271-S	STAP1	1	-0.128	-0.00897	-4.55	5.82E-06	0.000356
GI_7662295-S	FCHSD2	1	-0.126	-0.00326	-4.45	9.27E-06	0.000543
GI_11415027-S	TCL1A	1	-0.122	-0.00860	-4.34	1.53E-05	0.000837
GI_21264601-S	LAMA5	1	-0.120	-0.00598	-4.26	2.17E-05	0.00112
GI_20270264-A	FCRL2	1	-0.119	-0.0120	-4.23	2.47E-05	0.00124
Hs.444414-S	---	1	-0.118	-0.0105	-4.17	3.20E-05	0.00154
GI_19923628-I	FCRL2	1	-0.118	-0.00752	-4.17	3.21E-05	0.00154
GI_23238205-S	TNFRSF13B	1	-0.117	-0.00577	-4.16	3.41E-05	0.00162
Hs.469446-S	---	1	-0.116	-0.00779	-4.10	4.33E-05	0.00197
Hs.132571-S	---	1	-0.116	-0.0108	-4.10	4.33E-05	0.00197
GI_11038671-A	CD79A	1	-0.114	-0.00603	-4.02	6.15E-05	0.00266
GI_20302136-S	KIAA0125	1	-0.112	-0.0119	-3.98	7.22E-05	0.00302
GI_32698768-S	GNG7	1	-0.110	-0.00492	-3.90	0.000102	0.00402
GI_34147712-S	TLE1	1	-0.110	-0.00792	-3.89	0.000104	0.00409
GI_23110988-I	CD20	1	-0.109	-0.00597	-3.86	0.000117	0.00448
GI_31542206-S	FAM129C	1	-0.108	-0.00599	-3.83	0.000133	0.00498
GI_33469981-S	BLK	1	-0.108	-0.00491	-3.82	0.000137	0.00512
GI_42544162-S	FCRLA	1	-0.101	-0.00534	-3.58	0.000363	0.0113
GI_4826763-S	HS3ST1	1	-0.0991	-0.0158	-3.51	0.000472	0.0140
GI_7019566-S	VPREB3	1	-0.0982	-0.0120	-3.47	0.000529	0.0153
GI_15451903-S	CD200	1	-0.0981	-0.0104	-3.47	0.000537	0.0155
GI_32441282-I	RALGPS2	1	-0.0976	-0.0129	-3.45	0.000575	0.0164
Hs.136376-S	---	1	-0.0973	-0.0182	-3.44	0.000598	0.0169
GI_27777635-S	TCF3	1	-0.0973	-0.00263	-3.43	0.000619	0.0173
GI_13376686-S	KIAA0226L	1	-0.0963	-0.00455	-3.40	0.000686	0.0188
GI_32481214-S	CD19	1	-0.0949	-0.00502	-3.35	0.000826	0.0218
GI_14550413-S	FCRL5	1	-0.0934	-0.00789	-3.30	0.000992	0.0251
GI_9961251-A	ABCB4	1	-0.0925	-0.00750	-3.27	0.001103	0.0273
Hs.446193-S	---	1	-0.0917	-0.0134	-3.24	0.001212	0.0293
hmm3577-S	---	1	-0.0913	-0.00726	-3.23	0.001292	0.0307
GI_37546026-S	---	1	-0.0907	-0.00638	-3.21	0.001380	0.0323
hmm3574-S	---	1	-0.0879	-0.00606	-3.11	0.001933	0.0422
GI_8922306-I	---	1	-0.0863	-0.0152	-3.05	0.002349	0.0490
GI_11038675-A	CD79B	1	-0.0863	-0.00344	-3.04	0.002377	0.0494
GI_18375502-A	APEX1	2	-0.255	-0.00413	-9.18	1.80E-19	1.52E-16
GI_25777719-A	ALDH5A1	2	-0.207	-0.0133	-7.43	2.06E-13	6.73E-11
GI_5453602-S	CCT2	2	-0.195	-0.00413	-6.97	5.18E-12	1.25E-09
GI_16905527-A	DAP3	2	-0.189	-0.00237	-6.67	3.72E-11	7.69E-09
GI_34222198-S	LRRC8D	2	-0.172	-0.00383	-6.13	1.21E-09	1.77E-07
GI_17388802-S	PAICS	2	-0.168	-0.00707	-6.00	2.59E-09	3.57E-07

Legend
- Genes negatively correlated to age (blue)
- Genes positively correlated to age (red)

GI_4506792-S	ATXN1	2	0.168	0.00449	5.99	2.72E-09	3.73E-07
GI_38787975-S	TSPAN3	2	-0.167	-0.00675	-5.95	3.42E-09	4.57E-07
GI_37540654-S	ZNF518B	2	-0.162	-0.00616	-5.77	1.02E-08	1.23E-06
GI_22748692-S	ARL14EP	2	-0.157	-0.00463	-5.59	2.81E-08	3.11E-06
GI_4505454-S	NRIP1	2	-0.153	-0.00887	-5.44	6.31E-08	6.50E-06
GI_7657135-S	GPR160	2	-0.152	-0.00488	-5.39	8.55E-08	8.64E-06
GI_4506558-S	ABCE1	2	-0.151	-0.00462	-5.38	8.92E-08	8.99E-06
GI_27764872-S	CRLF3	2	-0.151	-0.00431	-5.36	1.00E-07	9.90E-06
GI_29570797-S	PPAT	2	-0.149	-0.00608	-5.31	1.27E-07	1.21E-05
GI_40254433-S	PDE3B	2	-0.148	-0.0245	-5.27	1.60E-07	1.49E-05
GI_10864048-S	FAM60A	2	-0.146	-0.00313	-5.18	2.53E-07	2.27E-05
GI_34222383-S	THNSL1	2	-0.144	-0.0244	-5.12	3.57E-07	3.06E-05
GI_7662509-S	LEPROTL1	2	-0.144	-0.00687	-5.11	3.82E-07	3.27E-05
GI_21359901-S	NSA2	2	-0.144	-0.00363	-5.09	4.12E-07	3.48E-05
GI_21265090-S	MRPL3	2	-0.140	-0.00450	-4.96	8.02E-07	6.27E-05
GI_24475984-S	TMEM14C	2	-0.139	-0.00425	-4.93	9.45E-07	7.23E-05
GI_16445423-S	WDR12	2	-0.139	-0.00422	-4.93	9.52E-07	7.27E-05
GI_19743810-S	TBK1	2	0.139	0.00255	4.89	1.15E-06	8.58E-05
GI_34147704-S	HMGN1	2	-0.138	-0.00469	-4.88	1.18E-06	8.75E-05
GI_40255150-S	---	2	-0.137	-0.00856	-4.87	1.27E-06	9.33E-05
GI_16950596-A	MRPS33	2	-0.136	-0.00314	-4.83	1.57E-06	0.000111
GI_7705432-S	EIF3L	2	-0.136	-0.00235	-4.79	1.89E-06	0.000131
GI_4502890-S	CLNS1A	2	-0.134	-0.00225	-4.72	2.58E-06	0.000173
GI_16306478-S	CEP41	2	-0.132	-0.0163	-4.69	3.00E-06	0.000198
GI_18482382-S	KLHL6	2	-0.132	-0.00601	-4.69	3.02E-06	0.000199
GI_37059742-S	NIFK	2	-0.133	-0.00315	-4.69	3.06E-06	0.000202
GI_42789728-S	LRMP	2	-0.131	-0.00220	-4.60	4.69E-06	0.000294
GI_40353732-S	NPM1	2	-0.129	-0.00536	-4.59	4.95E-06	0.000307
GI_4503520-S	EIF3E	2	-0.128	-0.00448	-4.53	6.58E-06	0.000399
GI_33286443-S	MAML2	2	-0.127	-0.00638	-4.52	6.73E-06	0.000406
GI_34335263-A	ZMYND8	2	-0.126	-0.00246	-4.45	9.48E-06	0.000554
GI_22748758-S	LRRC75A-AS1	2	-0.124	-0.00381	-4.40	1.19E-05	0.000677
GI_26190615-S	KLHDC1	2	-0.124	-0.00449	-4.39	1.23E-05	0.000699
GI_4506742-S	RPS8	2	-0.123	-0.00259	-4.34	1.53E-05	0.000837
GI_4506890-S	SET	2	-0.123	-0.00235	-4.34	1.56E-05	0.000854
GI_5730038-S	SETMAR	2	-0.122	-0.00347	-4.32	1.67E-05	0.000899
GI_14211872-S	EBPL	2	-0.120	-0.00320	-4.26	2.23E-05	0.00114
GI_37543779-S	AMN1	2	-0.120	-0.00519	-4.25	2.30E-05	0.00117
GI_10864022-S	ZBED5	2	-0.120	-0.00304	-4.24	2.38E-05	0.00121
GI_20127469-S	EEF1E1	2	-0.120	-0.00344	-4.23	2.56E-05	0.00128
GI_40789255-S	SRP72	2	-0.120	-0.00231	-4.21	2.72E-05	0.00134
GI_38455426-S	CCT4	2	-0.118	-0.00238	-4.14	3.64E-05	0.00171
GI_21359925-S	HMG20A	2	-0.117	-0.00376	-4.14	3.68E-05	0.00173
GI_13376622-S	THOC7	2	-0.117	-0.00215	-4.10	4.33E-05	0.00197
GI_8922630-S	EXD2	2	-0.116	-0.0198	-4.10	4.37E-05	0.00198
GI_20143934-I	RLN2	2	-0.114	-0.0198	-4.05	5.36E-05	0.00236
GI_7705727-S	CUTC	2	-0.115	-0.00224	-4.05	5.38E-05	0.00236
GI_22538484-S	PRPSAP2	2	-0.115	-0.00211	-4.04	5.61E-05	0.00245
GI_17402905-S	RPL22	2	-0.114	-0.00404	-4.03	5.82E-05	0.00253
GI_30315657-A	SPTBN1	2	-0.113	-0.0153	-4.01	6.30E-05	0.00272
GI_37694064-A	EBAG9	2	-0.114	-0.00212	-4.01	6.42E-05	0.00276
GI_17017971-S	RPL26L1	2	-0.114	-0.00209	-4.00	6.61E-05	0.00282
GI_15812219-S	RPL31	2	-0.114	-0.00167	-4.00	6.78E-05	0.00288
GI_13491165-S	PUM1	2	-0.113	-0.00214	-3.99	7.09E-05	0.00298
GI_24497598-A	PIGP	2	-0.112	-0.00295	-3.96	7.81E-05	0.00323
GI_11496890-S	ADH5	2	-0.112	-0.00377	-3.96	7.81E-05	0.00323
GI_40538800-S	TMEM200A	2	0.110	0.00698	3.89	0.000107	0.00418
GI_34147499-S	PPP1R2	2	-0.110	-0.00385	-3.88	0.000111	0.00429
GI_16418408-S	TMEM123	2	-0.110	-0.00463	-3.87	0.000112	0.00433
GI_13514830-S	DDX10	2	-0.110	-0.00226	-3.87	0.000113	0.00433
GI_8923351-S	MAP4K4	2	-0.109	-0.00376	-3.85	0.000123	0.00469
GI_16950602-S	MRPS35	2	-0.110	-0.00207	-3.85	0.000124	0.00470
GI_33519472-S	NDFIP1	2	-0.109	-0.00374	-3.84	0.000132	0.00494
GI_37538683-S	LINC-PINT	2	0.108	0.00558	3.81	0.000148	0.00545
Hs.508436-S	RABGAP1L	2	0.108	0.00473	3.80	0.000150	0.00551
GI_5453997-S	IPO7	2	-0.107	-0.00618	-3.80	0.000150	0.00552
GI_37550602-S	RPL22L1	2	-0.108	-0.00227	-3.79	0.000156	0.00567
GI_33239442-A	SYPL1	2	-0.107	-0.00366	-3.79	0.000161	0.00581
GI_40788009-S	CD55	2	-0.107	-0.00284	-3.77	0.000170	0.00609
GI_4504522-S	---	2	-0.106	-0.00383	-3.73	0.000201	0.00700
GI_7106298-S	ATXN10	2	-0.106	-0.00227	-3.72	0.000206	0.00712
GI_4758733-S	PMPCB	2	-0.106	-0.00188	-3.72	0.000207	0.00716
GI_23510357-A	RIOK1	2	-0.105	-0.00246	-3.71	0.000220	0.00753
GI_21314761-S	MCEE	2	-0.105	-0.00314	-3.71	0.000220	0.00753
GI_21389390-S	FOPNL	2	-0.105	-0.00171	-3.69	0.000232	0.00790
GI_33186904-S	OMA1	2	-0.104	-0.00345	-3.69	0.000237	0.00804
GI_21389336-S	RPIA	2	-0.104	-0.00206	-3.67	0.000256	0.00857
GI_24429565-I	PDE7A	2	-0.104	-0.0104	-3.67	0.000257	0.00858
GI_28416426-I	ZNF655	2	-0.104	-0.00207	-3.65	0.000273	0.00899
GI_38505204-S	PTEN	2	-0.103	-0.00211	-3.64	0.000286	0.00937
GI_42658173-S	SUN1	2	-0.102	-0.00256	-3.59	0.000340	0.0108
Hs.519802-S	NPM1	2	-0.102	-0.00434	-3.59	0.000340	0.0108
GI_21265077-S	MRPL15	2	-0.102	-0.00197	-3.59	0.000349	0.0110
GI_34147413-S	NOA1	2	-0.102	-0.00189	-3.58	0.000353	0.0111
GI_37059748-S	TBC1D15	2	-0.102	-0.00198	-3.58	0.000363	0.0113
GI_24429565-A	PDE7A	2	-0.101	-0.0141	-3.56	0.000385	0.0118
GI_31982952-I	POGLUT1	2	-0.100	-0.00337	-3.54	0.000413	0.0125

GI_8922941-S	AGPAT5	2	-0.100	-0.00387	-3.54	0.000415	0.0125
GI_16117786-A	RPL34	2	-0.100	-0.00313	-3.54	0.000418	0.0126
GI_38327549-S	LPAR6	2	0.100	0.00375	3.54	0.000423	0.0127
GI_4507130-S	SNRPF	2	-0.100	-0.00211	-3.53	0.000426	0.0128
GI_9506672-S	MIOS	2	-0.101	-0.00143	-3.53	0.000430	0.0129
GI_9966880-S	NUP107	2	-0.100	-0.00223	-3.53	0.000432	0.0129
GI_7662231-S	ZC3H11A	2	-0.100	-0.00224	-3.51	0.000462	0.0137
GI_14149729-S	SMARCAD1	2	-0.0987	-0.00528	-3.49	0.000500	0.0146
GI_8051634-S	XPO1	2	-0.0985	-0.00229	-3.47	0.000542	0.0156
GI_40255235-S	ZNF281	2	-0.0981	-0.00204	-3.45	0.000577	0.0165
GI_14042922-S	TMEM245	2	-0.0979	-0.00254	-3.45	0.000582	0.0166
GI_9951918-S	KATNA1	2	0.0975	0.00250	3.44	0.000610	0.0171
GI_29244580-S	ZDHHC17	2	-0.0971	-0.00244	-3.42	0.000642	0.0179
GI_31442407-S	SNRK	2	-0.0967	-0.00421	-3.42	0.000653	0.0182
GI_7662455-S	FOXJ3	2	-0.0974	-0.00162	-3.41	0.000659	0.0183
GI_17017970-S	RPL26	2	-0.0968	-0.00283	-3.41	0.000659	0.0183
GI_13375984-S	ZFAND1	2	-0.0968	-0.00308	-3.41	0.000659	0.0183
GI_4885386-S	HADH	2	-0.0970	-0.00221	-3.41	0.000662	0.0183
GI_7661795-S	GTPBP8	2	-0.0965	-0.00272	-3.40	0.000685	0.0188
GI_42476207-S	AMZ2	2	0.0961	0.00220	3.38	0.000738	0.0199
GI_16306499-A	FBXO21	2	-0.0963	-0.00162	-3.37	0.000765	0.0204
GI_29171760-S	PPA1	2	-0.0953	-0.00255	-3.36	0.000802	0.0213
GI_21237801-S	SMARCC1	2	-0.0962	-0.00140	-3.36	0.000804	0.0213
GI_18699723-S	PNISR	2	-0.0950	-0.00301	-3.35	0.000828	0.0218
GI_4505816-S	PIP5K1B	2	-0.0946	-0.0145	-3.34	0.000849	0.0222
GI_13376244-S	GGNBP2	2	-0.0953	-0.00164	-3.34	0.000856	0.0224
GI_27501445-S	DENR	2	-0.0950	-0.00185	-3.34	0.000877	0.0228
GI_12545407-S	RASA2	2	-0.0940	-0.00397	-3.32	0.000925	0.0237
GI_34101266-A	BCKDHB	2	-0.0939	-0.00444	-3.32	0.000930	0.0238
GI_30425377-S	DRAM2	2	-0.0935	-0.00272	-3.30	0.001006	0.0253
GI_29743852-S	PRMT3	2	-0.0929	-0.00309	-3.28	0.001074	0.0267
GI_7656970-S	N4BP2L2	2	-0.0930	-0.00232	-3.28	0.001081	0.0268
GI_20357534-S	ATP6V1G1	2	-0.0929	-0.00192	-3.26	0.001131	0.0278
GI_12597621-S	CLPX	2	-0.0925	-0.00215	-3.26	0.001162	0.0284
GI_14591917-S	SEH1L	2	-0.0919	-0.00431	-3.24	0.001206	0.0292
GI_41151340-S	HLA-F-AS1	2	-0.0919	-0.00223	-3.24	0.001242	0.0298
GI_5729936-S	MTX2	2	-0.0918	-0.00253	-3.23	0.001250	0.0300
GI_23270402-A	CRYZL1	2	-0.0915	-0.00244	-3.22	0.001299	0.0309
GI_4504450-S	RBMX	2	-0.0921	-0.00146	-3.22	0.001303	0.0309
GI_41054845-S	ARMCX5	2	-0.0907	-0.00803	-3.21	0.001375	0.0322
GI_31543090-S	MAK16	2	-0.0908	-0.00222	-3.20	0.001421	0.0331
GI_38158017-S	CNTRL	2	-0.0907	-0.00245	-3.20	0.001424	0.0332
GI_6912487-S	LSM5	2	-0.0906	-0.00274	-3.20	0.001430	0.0333
GI_34328903-A	PIGN	2	-0.0903	-0.00282	-3.18	0.001484	0.0343
GI_14110394-S	XBP1	2	-0.0905	-0.00190	-3.18	0.001513	0.0348
GI_40254881-S	C21orf91	2	0.0900	0.00342	3.18	0.001528	0.0351
GI_13384595-S	ZNF330	2	-0.0900	-0.00187	-3.16	0.001608	0.0366
GI_19923363-S	ARPP19	2	0.0889	0.00247	3.13	0.001778	0.0395
GI_32483368-A	ORC3	2	-0.0887	-0.00371	-3.13	0.001783	0.0396
GI_25777737-S	ALDH6A1	2	-0.0886	-0.00235	-3.12	0.001844	0.0406
GI_4505540-S	USO1	2	-0.0882	-0.00199	-3.10	0.001968	0.0428
GI_32306540-S	TRIT1	2	-0.0877	-0.00370	-3.10	0.002011	0.0435
GI_19311011-S	BRIX1	2	-0.0878	-0.00218	-3.09	0.002036	0.0440
GI_11863158-S	RYK	2	-0.0876	-0.00181	-3.08	0.002124	0.0454
GI_21389378-S	GTSF1	2	0.0871	0.00398	3.08	0.002141	0.0457
GI_39725682-S	NARS2	2	-0.0870	-0.00565	-3.07	0.002164	0.0460
GI_37059785-S	LRRN3	3	-0.529	-0.0431	-21.9	2.74E-90	1.29E-85
GI_4505674-S	PDE9A	3	-0.390	-0.0268	-14.9	2.56E-46	4.04E-42
GI_4503680-S	FCGBP	3	-0.357	-0.0179	-13.4	1.47E-38	1.16E-34
GI_4503746-S	FLNB	3	-0.319	-0.0101	-11.8	1.60E-30	6.86E-27
GI_30795213-S	CCR7	3	-0.317	-0.0168	-11.7	3.08E-30	1.21E-26
GI_4507052-S	SLC7A6	3	-0.289	-0.00832	-10.6	3.45E-25	6.79E-22
GI_31542834-S	GAL3ST4	3	-0.268	-0.0174	-9.80	6.64E-22	7.66E-19
GI_22749458-S	GPC2	3	-0.265	-0.0138	-9.68	2.03E-21	2.16E-18
GI_21361616-S	FAM134B	3	-0.265	-0.0146	-9.68	2.06E-21	2.16E-18
GI_34147607-S	NOSIP	3	-0.265	-0.00760	-9.63	3.31E-21	3.33E-18
Hs.146543-S	---	3	-0.264	-0.0140	-9.62	3.59E-21	3.53E-18
GI_19923451-S	LEF1	3	-0.261	-0.0153	-9.50	1.05E-20	1.01E-17
GI_42661554-S	ZNF667-AS1	3	-0.259	-0.0168	-9.44	1.78E-20	1.65E-17
GI_40354211-S	IPCEF1	3	-0.256	-0.00658	-9.28	7.29E-20	6.39E-17
GI_23510435-S	CD27	3	-0.244	-0.0121	-8.86	2.63E-18	1.97E-15
GI_37552665-S	SGK223	3	-0.244	-0.00961	-8.84	3.32E-18	2.38E-15
GI_37059752-S	SUSD3	3	-0.242	-0.00741	-8.77	5.73E-18	3.82E-15
GI_33356175-S	SATB1	3	-0.241	-0.0111	-8.74	7.49E-18	4.92E-15
GI_31341850-S	LINC00282	3	-0.239	-0.0180	-8.67	1.30E-17	8.40E-15
GI_34147626-S	ZSCAN18	3	-0.236	-0.0177	-8.53	4.16E-17	2.55E-14
GI_21361852-S	PLXDC1	3	-0.235	-0.0129	-8.50	5.39E-17	3.27E-14
GI_19923906-S	OXNAD1	3	-0.233	-0.0153	-8.44	8.63E-17	5.10E-14
GI_4505668-S	PDE6B	3	-0.233	-0.00668	-8.41	1.08E-16	6.31E-14
GI_21284386-A	ABLIM1	3	-0.231	-0.00952	-8.36	1.62E-16	8.92E-14
GI_42660348-S	---	3	-0.229	-0.0119	-8.29	2.98E-16	1.62E-13
GI_5453765-S	NELL2	3	-0.224	-0.0148	-8.09	1.37E-15	6.81E-13
GI_22748704-S	BTBD11	3	-0.223	-0.00965	-8.04	2.12E-15	1.04E-12
GI_33354248-S	DNAJA4	3	-0.220	-0.00499	-7.90	6.16E-15	2.75E-12
GI_37546957-S	TRABD2A	3	-0.214	-0.0128	-7.72	2.30E-14	8.71E-12
GI_42558263-S	FAM102A	3	-0.207	-0.00689	-7.42	2.12E-13	6.86E-11
GI_19923900-S	PCED1B	3	-0.200	-0.00700	-7.16	1.42E-12	3.83E-10

GI_4507610-S	TNK1	3	-0.199	-0.0270	-7.15	1.44E-12	3.85E-10
GI_4505466-S	NT5E	3	-0.197	-0.0216	-7.06	2.82E-12	7.17E-10
GI_22748948-S	IGF1R	3	-0.193	-0.0105	-6.91	7.82E-12	1.84E-09
GI_7706512-S	RAPGEF6	3	-0.191	-0.00560	-6.82	1.38E-11	3.09E-09
GI_22027529-S	LDLRAP1	3	-0.188	-0.00711	-6.74	2.42E-11	5.23E-09
GI_18426908-A	SIRPG	3	-0.188	-0.00887	-6.74	2.49E-11	5.32E-09
GI_37545997-S	C14orf64	3	-0.188	-0.0301	-6.74	2.50E-11	5.33E-09
GI_37546229-S	BEX4	3	-0.188	-0.00552	-6.73	2.53E-11	5.37E-09
GI_8393671-S	KLHL3	3	-0.187	-0.00638	-6.68	3.68E-11	7.63E-09
GI_42660596-S	IGF1R	3	-0.184	-0.0143	-6.60	5.91E-11	1.16E-08
GI_42660198-S	TBC1D4	3	-0.185	-0.00812	-6.60	6.00E-11	1.18E-08
GI_20143980-S	HSD17B8	3	-0.185	-0.00518	-6.60	6.06E-11	1.18E-08
GI_27262654-A	IL16	3	-0.182	-0.00451	-6.51	1.11E-10	2.05E-08
GI_22507386-S	CELA1	3	-0.179	-0.0160	-6.39	2.34E-10	3.98E-08
GI_13540489-S	BACH2	3	-0.177	-0.0121	-6.35	3.10E-10	5.16E-08
Hs.264076-S	RBM26-AS1	3	-0.176	-0.0243	-6.29	4.36E-10	7.02E-08
GI_34222221-S	EPHX2	3	-0.174	-0.0255	-6.24	6.03E-10	9.35E-08
GI_19923326-S	NET1	3	-0.175	-0.00500	-6.22	6.63E-10	1.02E-07
GI_21614516-A	GYPC	3	-0.169	-0.00528	-6.04	2.07E-09	2.96E-07
GI_34147517-S	FAIM3	3	-0.169	-0.00719	-6.02	2.29E-09	3.24E-07
GI_8393383-S	C6orf48	3	-0.166	-0.00475	-5.89	4.91E-09	6.41E-07
GI_42476192-S	TMEM66	3	-0.164	-0.00324	-5.81	8.01E-09	9.94E-07
GI_13376301-S	---	3	-0.161	-0.0154	-5.73	1.25E-08	1.50E-06
GI_11968026-S	AKTIP	3	-0.159	-0.00479	-5.66	1.84E-08	2.11E-06
GI_34222126-S	PIK3IP1	3	-0.158	-0.00741	-5.63	2.27E-08	2.55E-06
GI_32698973-S	LINC00663	3	-0.158	-0.00659	-5.62	2.42E-08	2.71E-06
GI_31543215-S	MYC	3	-0.155	-0.00682	-5.52	4.17E-08	4.45E-06
GI_37059747-S	ADPRM	3	-0.153	-0.00486	-5.43	6.88E-08	7.03E-06
GI_22035553-A	APBB1	3	-0.150	-0.00768	-5.33	1.16E-07	1.14E-05
GI_23110934-I	PSMA1	3	-0.146	-0.0127	-5.20	2.37E-07	2.14E-05
GI_41393585-A	DGKA	3	-0.145	-0.00559	-5.15	3.05E-07	2.68E-05
GI_4504706-S	INPP4B	3	0.143	0.00531	5.09	4.15E-07	3.49E-05
GI_31542293-S	CD2	3	0.141	0.00622	5.00	6.49E-07	5.25E-05
GI_22027626-A	TRAF5	3	-0.140	-0.00400	-4.98	7.27E-07	5.77E-05
GI_32699055-S	---	3	-0.138	-0.00691	-4.90	1.06E-06	8.02E-05
Hs.519321-S	---	3	-0.136	-0.0206	-4.84	1.49E-06	0.000107
GI_13375927-S	ANKRD55	3	-0.136	-0.0249	-4.83	1.52E-06	0.000108
hmm8270-S	FAM159A	3	-0.134	-0.00590	-4.75	2.27E-06	0.000154
GI_4507742-S	TXK	3	-0.133	-0.00975	-4.73	2.55E-06	0.000171
GI_33624782-I	SEPT6	3	-0.131	-0.00420	-4.64	3.84E-06	0.000248
GI_27477118-S	CAMK4	3	-0.131	-0.0134	-4.64	3.85E-06	0.000248
GI_32967308-S	EPHA1	3	-0.131	-0.0118	-4.64	3.89E-06	0.000251
GI_40255142-S	FAM184A	3	-0.129	-0.0190	-4.59	4.80E-06	0.000300
GI_34147571-S	ISG20	3	-0.129	-0.00358	-4.56	5.71E-06	0.000350
GI_34594658-S	MAPKAPK5-AS1	3	-0.128	-0.00264	-4.51	6.95E-06	0.000418
GI_28372504-S	CBX7	3	0.125	0.00328	4.43	1.05E-05	0.000607
GI_24497579-S	AKR1B1	3	-0.126	-0.00201	-4.42	1.06E-05	0.000613
Hs.497588-S	---	3	-0.123	-0.0239	-4.36	1.38E-05	0.000768
GI_44680134-A	BDH1	3	-0.123	-0.0104	-4.36	1.38E-05	0.000768
GI_18426907-I	SIRPG	3	-0.121	-0.0100	-4.28	2.00E-05	0.00105
GI_18598508-S	CDR2	3	-0.120	-0.00711	-4.27	2.12E-05	0.00110
GI_24475858-S	ZNF101	3	-0.119	-0.00429	-4.23	2.52E-05	0.00126
hmm18828-S	BEX2	3	-0.118	-0.0109	-4.19	3.00E-05	0.00146
GI_4758815-S	NMT2	3	-0.118	-0.00626	-4.18	3.08E-05	0.00149
GI_7657436-S	SESN1	3	-0.117	-0.00398	-4.14	3.76E-05	0.00175
hmm19321-S	PRKCQ-AS1	3	-0.117	-0.00727	-4.13	3.83E-05	0.00178
GI_38045937-S	RNF144A	3	-0.115	-0.00360	-4.07	5.06E-05	0.00225
GI_21362099-S	ELOVL4	3	-0.115	-0.0101	-4.06	5.17E-05	0.00228
GI_38158029-S	SRSF6	3	-0.113	-0.00214	-3.97	7.70E-05	0.00319
GI_37546926-S	---	3	-0.112	-0.00262	-3.95	8.39E-05	0.00341
GI_13569871-S	LBH	3	-0.111	-0.00454	-3.92	9.22E-05	0.00370
GI_7657101-S	PGAP2	3	-0.111	-0.00501	-3.92	9.51E-05	0.00379
GI_22062022-S	TMEM41B	3	-0.110	-0.00300	-3.90	0.000103	0.00403
GI_12232446-S	CRTC3	3	-0.110	-0.00290	-3.89	0.000105	0.00411
GI_31542322-S	CRIP2	3	0.110	0.00942	3.89	0.000105	0.00411
GI_16905512-S	RPLP2	3	-0.110	-0.00237	-3.89	0.000106	0.00415
GI_29735368-S	PP7080	3	-0.110	-0.00391	-3.89	0.000107	0.00418
GI_19923670-S	FOXP1	3	-0.110	-0.00367	-3.87	0.000114	0.00437
GI_40255167-S	TSHZ2	3	0.109	0.0169	3.85	0.000123	0.00469
GI_28144902-S	IL23A	3	-0.109	-0.00632	-3.84	0.000129	0.00486
GI_4503718-S	FHIT	3	-0.107	-0.00787	-3.80	0.000150	0.00552
GI_5453610-S	CD28	3	-0.107	-0.00510	-3.79	0.000155	0.00566
Hs.137587-S	LOC643733	3	0.107	0.00532	3.77	0.000169	0.00605
GI_4758719-S	MLLT3	3	-0.106	-0.00383	-3.75	0.000187	0.00659
GI_42659994-S	---	3	-0.105	-0.00342	-3.72	0.000211	0.00728
GI_28610150-S	IL7R	3	-0.105	-0.00596	-3.71	0.000215	0.00738
GI_7662075-S	KIAA0355	3	-0.104	-0.00234	-3.65	0.000271	0.00893
GI_37551029-S	FAM171A1	3	-0.103	-0.0111	-3.63	0.000296	0.00965
GI_6996015-A	LTB	3	-0.100	-0.00474	-3.54	0.000410	0.0125
GI_21361928-S	SLC38A1	3	-0.100	-0.00339	-3.54	0.000415	0.0125
GI_35038527-S	PASK	3	-0.100	-0.00666	-3.53	0.000424	0.0128
GI_9257221-S	FOXO1	3	-0.100	-0.00397	-3.53	0.000425	0.0128
GI_12408664-A	MAL	3	-0.0993	-0.00545	-3.51	0.000463	0.0137
GI_4753158-S	GTF3A	3	-0.100	-0.00207	-3.50	0.000481	0.0141
GI_18765693-S	DPP4	3	-0.0970	-0.00397	-3.43	0.000631	0.0176
GI_14110406-A	HNRNPDL	3	-0.0967	-0.00332	-3.41	0.000662	0.0183
GI_28827775-S	LY9	3	-0.0962	-0.00373	-3.40	0.000706	0.0193

Hs.439334-S	---	3	0.0954	0.00578	3.37	0.000767	0.0205
GI_7661651-S	ZBTB20	3	-0.0949	-0.00271	-3.35	0.000845	0.0222
Hs.510761-S	---	3	-0.0936	-0.00655	-3.31	0.000969	0.0246
GI_27500902-S	---	3	-0.0927	-0.00234	-3.27	0.001120	0.0276
GI_9966902-S	MAN1C1	3	-0.0914	-0.00618	-3.23	0.001272	0.0304
GI_24475729-S	CCDC65	3	0.0911	0.00699	3.22	0.001322	0.0312
GI_42661225-S	PRKCA	3	-0.0894	-0.00335	-3.15	0.001652	0.0374
GI_42658639-S	KIAA1147	3	-0.0888	-0.00289	-3.13	0.001781	0.0396
GI_20336210-I	GAMT	3	-0.0880	-0.00896	-3.11	0.001908	0.0417
Hs.349094-S	TENM1	3	-0.0871	-0.00525	-3.08	0.002150	0.0457
GI_21359840-S	TTC3	3	-0.0867	-0.00320	-3.06	0.002281	0.0480
GI_42657549-S	---	3	-0.0869	-0.00149	-3.04	0.002389	0.0495
GI_4557268-S	ADSL	4	-0.205	-0.00300	-7.29	5.44E-13	1.64E-10
GI_31543180-S	PCYOX1L	4	-0.203	-0.00380	-7.24	7.91E-13	2.30E-10
GI_13376239-S	ZNF671	4	-0.195	-0.00541	-6.98	4.67E-12	1.14E-09
GI_37546867-S	WDR43	4	-0.193	-0.00485	-6.90	8.41E-12	1.96E-09
GI_9558740-S	FAM216A	4	-0.191	-0.00461	-6.80	1.58E-11	3.49E-09
GI_22748624-S	ZNF439	4	-0.183	-0.00852	-6.55	8.34E-11	1.56E-08
GI_21314689-S	NGLY1	4	-0.169	-0.00307	-6.00	2.58E-09	3.57E-07
GI_5730016-S	RPP40	4	-0.165	-0.00641	-5.89	5.10E-09	6.60E-07
GI_39725714-S	ATIC	4	-0.165	-0.00331	-5.86	5.91E-09	7.49E-07
GI_8923268-S	TEX10	4	-0.161	-0.00304	-5.69	1.62E-08	1.88E-06
GI_22748614-S	C12orf23	4	-0.157	-0.00704	-5.58	2.93E-08	3.24E-06
GI_40786390-S	DNAJA3	4	-0.152	-0.00303	-5.37	9.44E-08	9.37E-06
GI_7770073-A	IARS	4	-0.145	-0.00331	-5.14	3.12E-07	2.73E-05
GI_27482477-S	---	4	-0.143	-0.00474	-5.07	4.62E-07	3.83E-05
GI_22325391-S	TIGD7	4	-0.138	-0.00754	-4.91	1.01E-06	7.72E-05
GI_24431963-S	HBS1L	4	-0.138	-0.00436	-4.88	1.22E-06	9.02E-05
GI_23308516-S	ZNF75A	4	-0.137	-0.00535	-4.84	1.43E-06	0.000103
GI_4757883-S	LDLRAD4	4	-0.135	-0.00530	-4.80	1.77E-06	0.000123
GI_12965196-S	GRAMD3	4	-0.131	-0.0190	-4.65	3.64E-06	0.000236
GI_8923943-S	NKRF	4	-0.130	-0.00360	-4.61	4.36E-06	0.000275
GI_31415881-S	EXOSC8	4	-0.130	-0.00246	-4.60	4.73E-06	0.000297
GI_32698917-S	NSUN6	4	-0.129	-0.00329	-4.57	5.33E-06	0.000328
GI_38679908-S	TEX2	4	-0.125	-0.00407	-4.42	1.09E-05	0.000625
GI_36287129-S	MRPS25	4	-0.124	-0.00574	-4.38	1.28E-05	0.000722
GI_20336200-A	ATM	4	-0.123	-0.00633	-4.36	1.43E-05	0.000790
GI_21362021-S	ZNF419	4	-0.123	-0.00286	-4.35	1.50E-05	0.000826
GI_21717806-S	TBC1D31	4	-0.122	-0.00324	-4.32	1.66E-05	0.000897
GI_34303962-A	POGLUT1	4	-0.121	-0.00228	-4.28	2.05E-05	0.00107
GI_20127480-S	EPM2A	4	-0.121	-0.00326	-4.27	2.06E-05	0.00108
GI_37594468-A	HIBCH	4	-0.121	-0.00367	-4.26	2.18E-05	0.00112
GI_21265083-S	MRPL1	4	-0.120	-0.00246	-4.24	2.35E-05	0.00119
GI_7669480-S	BNIP3	4	-0.120	-0.00421	-4.23	2.47E-05	0.00124
GI_23308510-S	ZFP3	4	-0.119	-0.00626	-4.20	2.87E-05	0.00140
GI_34147689-S	DENND2D	4	-0.119	-0.00361	-4.20	2.88E-05	0.00141
GI_42658518-S	ZNF789	4	-0.118	-0.00499	-4.19	2.93E-05	0.00143
GI_11024703-S	TRMT13	4	-0.118	-0.00355	-4.18	3.14E-05	0.00151
GI_31377626-S	NOC3L	4	-0.118	-0.00392	-4.16	3.43E-05	0.00163
GI_42794755-S	ACSL5	4	0.118	0.00234	4.15	3.49E-05	0.00165
GI_23503246-S	TCEAL8	4	-0.118	-0.00258	-4.15	3.52E-05	0.00166
GI_11034834-I	---	4	-0.117	-0.00851	-4.14	3.69E-05	0.00173
GI_45593129-S	GNL3	4	-0.117	-0.00398	-4.13	3.83E-05	0.00178
GI_31341800-S	C5orf51	4	-0.116	-0.00425	-4.12	4.07E-05	0.00186
GI_7706780-I	ZNF226	4	-0.114	-0.00486	-4.05	5.39E-05	0.00236
GI_4505430-S	NPAT	4	-0.113	-0.00456	-4.01	6.44E-05	0.00276
Hs.493585-S	---	4	-0.112	-0.0127	-3.99	7.13E-05	0.00299
GI_42656546-S	ANKAR	4	-0.112	-0.00499	-3.97	7.71E-05	0.00319
GI_19913370-S	TBL1XR1	4	-0.112	-0.00256	-3.93	8.83E-05	0.00356
GI_7661809-S	TIMM21	4	-0.111	-0.00241	-3.91	9.63E-05	0.00382
GI_39995081-S	NSUN2	4	-0.112	-0.00167	-3.91	9.79E-05	0.00388
GI_40255000-S	N4BP2L2	4	0.110	0.00269	3.88	0.000109	0.00424
GI_40789232-S	COQ9	4	-0.110	-0.00179	-3.88	0.000112	0.00432
GI_40789269-S	ZNF573	4	-0.109	-0.00372	-3.87	0.000116	0.00445
GI_38201711-A	DDX17	4	-0.109	-0.00237	-3.85	0.000124	0.00470
GI_33620772-S	KIAA0020	4	-0.108	-0.00276	-3.82	0.000138	0.00513
GI_37552523-S	---	4	-0.108	-0.0112	-3.81	0.000145	0.00537
GI_28827773-S	DYRK4	4	-0.108	-0.00269	-3.81	0.000147	0.00543
GI_41150782-S	OXLD1	4	-0.107	-0.00286	-3.79	0.000161	0.00581
GI_42741681-S	ZRANB2	4	-0.105	-0.00338	-3.72	0.000211	0.00728
GI_21327681-S	ACADM	4	-0.104	-0.00256	-3.66	0.000267	0.00885
GI_21735567-S	CEPT1	4	-0.104	-0.00227	-3.65	0.000274	0.00900
GI_33946328-S	RALA	4	-0.103	-0.00248	-3.62	0.000309	0.00997
GI_40254985-S	HSDL2	4	-0.102	-0.00208	-3.60	0.000328	0.0105
GI_38327028-S	UBE4A	4	-0.102	-0.00185	-3.60	0.000333	0.0106
GI_14110410-I	HNRNPDL	4	-0.101	-0.00290	-3.58	0.000361	0.0113
GI_16418033-S	N4BP2L1	4	-0.102	-0.00184	-3.57	0.000369	0.0114
GI_4758293-S	EPRS	4	-0.101	-0.00206	-3.56	0.000380	0.0117
GI_6005726-S	CCT8	4	-0.100	-0.00287	-3.54	0.000415	0.0125
GI_29826334-S	EIF2S2	4	-0.101	-0.00157	-3.53	0.000435	0.0130
GI_20149642-S	ZNHIT6	4	-0.100	-0.00283	-3.53	0.000435	0.0130
GI_14149614-S	RPAIN	4	-0.101	-0.00155	-3.52	0.000451	0.0134
GI_24308321-S	ZCCHC7	4	-0.100	-0.00343	-3.52	0.000454	0.0135
GI_32307149-A	OGT	4	-0.0990	-0.00288	-3.49	0.000498	0.0145
GI_26667176-S	MRPL46	4	-0.0992	-0.00206	-3.49	0.000500	0.0146
GI_23618925-S	ZNF559	4	-0.0983	-0.00313	-3.47	0.000540	0.0156
GI_37543284-S	ZNF594	4	-0.0976	-0.00964	-3.45	0.000574	0.0164

GI_6912325-S	FAM50B	4	-0.0975	-0.00596	-3.45	0.000587	0.0166
GI_16950599-S	MRPS31	4	-0.0977	-0.00274	-3.44	0.000591	0.0167
GI_17017972-S	RPL27	4	-0.0979	-0.00167	-3.43	0.000619	0.0173
GI_4504160-S	GRSF1	4	-0.0976	-0.00171	-3.42	0.000639	0.0178
GI_28372492-S	CUL4B	4	-0.0971	-0.00182	-3.41	0.000667	0.0184
GI_21361826-S	METTL3	4	-0.0959	-0.00235	-3.38	0.000751	0.0202
GI_5729771-S	CLN5	4	-0.0956	-0.00341	-3.38	0.000757	0.0203
GI_20336299-S	DHX32	4	-0.0957	-0.00245	-3.37	0.000772	0.0206
GI_38570159-S	XPO4	4	-0.0954	-0.00207	-3.35	0.000821	0.0217
GI_4826685-S	DDX1	4	-0.0946	-0.00209	-3.33	0.000896	0.0231
GI_37550558-S	U2SURP	4	-0.0942	-0.00218	-3.31	0.000943	0.0241
GI_45446742-S	DDX42	4	-0.0947	-0.00135	-3.31	0.000974	0.0247
GI_21626467-S	ZNF638	4	-0.0932	-0.00242	-3.28	0.001054	0.0263
Hs.13250-S	WDR33	4	-0.0928	-0.00362	-3.28	0.001078	0.0268
GI_20143932-A	RLN2	4	-0.0926	-0.00496	-3.27	0.001105	0.0274
GI_22035593-A	MRPL35	4	-0.0925	-0.00256	-3.26	0.001148	0.0282
GI_39930470-S	METTL25	4	-0.0924	-0.00246	-3.26	0.001164	0.0284
GI_21314666-S	CPSF3	4	-0.0920	-0.00176	-3.23	0.001274	0.0304
GI_22212923-I	NFX1	4	-0.0917	-0.00187	-3.22	0.001301	0.0309
GI_21361708-S	RPRD1A	4	-0.0914	-0.00246	-3.22	0.001311	0.0310
GI_32698693-S	DCUN1D4	4	-0.0914	-0.00228	-3.22	0.001318	0.0312
GI_24497452-S	NUP88	4	-0.0914	-0.00184	-3.21	0.001356	0.0319
GI_8922499-S	SHQ1	4	-0.0886	-0.00268	-3.12	0.001822	0.0402
GI_25914753-A	MKKS	4	-0.0876	-0.00265	-3.09	0.002069	0.0445
GI_38257145-S	ANKRD46	4	-0.0869	-0.00568	-3.07	0.002182	0.0463
GI_32129211-S	ZSCAN32	4	-0.0869	-0.00238	-3.06	0.002263	0.0477
GI_39725646-S	ALG8	4	-0.0872	-0.00149	-3.05	0.002302	0.0483
GI_8923000-S	ABHD10	4	-0.0866	-0.00266	-3.05	0.002319	0.0485
GI_15451891-S	EIF5B	4	-0.0866	-0.00214	-3.05	0.002349	0.0490
GI_24307912-S	ZNF234	4	-0.0863	-0.00363	-3.05	0.002362	0.0492
GI_4885496-S	MYB	5	-0.273	-0.0100	-9.97	1.46E-22	1.92E-19
GI_4826869-S	NUCB2	5	-0.236	-0.0111	-8.54	4.00E-17	2.49E-14
GI_7661743-S	BZW2	5	-0.218	-0.00631	-7.85	8.82E-15	3.79E-12
GI_41352701-S	DPH2	5	-0.218	-0.00380	-7.78	1.46E-14	5.91E-12
GI_33356127-I	NAA16	5	-0.195	-0.00842	-6.99	4.60E-12	1.13E-09
GI_7661669-S	SH3YL1	5	-0.185	-0.00692	-6.62	5.33E-11	1.07E-08
GI_37545625-S	TAF4B	5	-0.180	-0.00896	-6.44	1.67E-10	3.00E-08
GI_37595546-S	PDK1	5	-0.179	-0.00905	-6.40	2.26E-10	3.87E-08
GI_30425401-S	RPF2	5	-0.178	-0.00421	-6.34	3.24E-10	5.36E-08
GI_14110370-S	CDC16	5	-0.178	-0.00382	-6.32	3.69E-10	6.03E-08
GI_6806888-S	HSF2	5	-0.173	-0.00505	-6.18	8.55E-10	1.29E-07
GI_15812220-S	RPL32	5	-0.165	-0.00280	-5.83	7.18E-09	8.99E-07
GI_22726178-S	LDHB	5	-0.157	-0.00674	-5.58	3.01E-08	3.31E-06
GI_31542492-S	DBP	5	-0.151	-0.00645	-5.38	8.98E-08	9.03E-06
GI_19743797-A	ZNF274	5	-0.150	-0.00354	-5.32	1.22E-07	1.17E-05
GI_37539232-S	EEF1B2	5	-0.143	-0.00497	-5.09	4.21E-07	3.53E-05
GI_45120103-S	MGC70870	5	-0.143	-0.00835	-5.08	4.33E-07	3.61E-05
GI_16757969-I	FAM129A	5	0.142	0.00375	5.04	5.25E-07	4.31E-05
GI_16506300-S	EPB41L4A-AS1	5	-0.142	-0.00310	-5.01	6.29E-07	5.10E-05
GI_37546335-S	NOL11	5	-0.141	-0.00331	-5.00	6.61E-07	5.31E-05
GI_13518227-S	MCCC1	5	-0.141	-0.00334	-4.99	6.82E-07	5.44E-05
GI_29568100-S	ATP5L	5	-0.138	-0.00281	-4.88	1.23E-06	9.03E-05
GI_6631103-S	EI24	5	-0.136	-0.00239	-4.81	1.70E-06	0.000119
GI_33942063-S	SEMA4D	5	-0.130	-0.00296	-4.58	5.13E-06	0.000318
GI_16936532-A	CDK4	5	-0.129	-0.00226	-4.55	6.02E-06	0.000367
GI_4505286-S	IRF4	5	-0.128	-0.00623	-4.52	6.64E-06	0.000401
GI_5454101-S	TACC3	5	-0.126	-0.00347	-4.45	9.27E-06	0.000543
GI_29550827-S	ACBD3	5	-0.126	-0.00260	-4.45	9.32E-06	0.000546
GI_31542291-S	CCT3	5	-0.124	-0.00395	-4.38	1.27E-05	0.000717
GI_25306276-S	GFM1	5	-0.121	-0.00282	-4.26	2.22E-05	0.00114
GI_14602425-S	SEC62	5	-0.120	-0.00243	-4.23	2.49E-05	0.00125
GI_24308126-S	DNAJC10	5	-0.119	-0.00360	-4.20	2.81E-05	0.00138
GI_31542714-S	TUG1	5	-0.117	-0.00292	-4.14	3.68E-05	0.00173
GI_12232418-S	S100PBP	5	-0.116	-0.00303	-4.10	4.45E-05	0.00201
GI_42476016-S	ACPL2	5	-0.115	-0.00515	-4.09	4.63E-05	0.00209
GI_42656629-S	RPL22	5	-0.115	-0.00597	-4.09	4.67E-05	0.00210
GI_34304340-A	PDCD4	5	-0.115	-0.00267	-4.07	4.92E-05	0.00219
GI_8923721-S	UTP6	5	-0.115	-0.00231	-4.05	5.43E-05	0.00238
GI_42661295-S	---	5	-0.114	-0.00292	-4.02	6.27E-05	0.00271
Hs.402644-S	---	5	-0.113	-0.00602	-3.98	7.14E-05	0.00300
GI_34222329-S	NOP58	5	-0.113	-0.00288	-3.98	7.20E-05	0.00302
GI_44890067-S	ZMYM4	5	-0.112	-0.00260	-3.96	8.02E-05	0.00330
GI_4557580-S	FABP5	5	-0.110	-0.00552	-3.91	9.82E-05	0.00388
GI_23463298-S	ST13P4	5	-0.109	-0.00402	-3.85	0.000123	0.00469
GI_42659734-S	---	5	-0.109	-0.00425	-3.85	0.000127	0.00479
GI_29728071-S	TBC1D9	5	-0.108	-0.00340	-3.83	0.000133	0.00498
GI_31340578-S	IQCG	5	0.108	0.00514	3.83	0.000133	0.00499
GI_27886565-A	MALT1	5	-0.107	-0.00283	-3.78	0.000161	0.00582
GI_4507128-S	SNRPE	5	-0.107	-0.00352	-3.78	0.000166	0.00596
GI_8923935-S	USE1	5	-0.106	-0.00290	-3.73	0.000198	0.00693
GI_34304321-S	MRPL45	5	-0.105	-0.00346	-3.70	0.000228	0.00780
GI_42658709-S	RPL15	5	-0.105	-0.00322	-3.69	0.000234	0.00795
GI_24429563-I	---	5	-0.104	-0.0143	-3.68	0.000247	0.00832
GI_21237722-S	ST13	5	-0.103	-0.00461	-3.66	0.000264	0.00876
GI_23111044-S	SNX4	5	-0.102	-0.00250	-3.60	0.000328	0.0105
GI_33624820-A	SEPT6	5	-0.102	-0.00330	-3.60	0.000334	0.0106
GI_39930468-S	RPF2	5	-0.102	-0.00475	-3.59	0.000345	0.0109

GI_21362101-S	TMEM168	5	-0.102	-0.00315	-3.59	0.000349	0.0110
GI_23463288-S	---	5	-0.100	-0.00337	-3.53	0.000430	0.0129
GI_4505276-S	MTIF2	5	-0.100	-0.00247	-3.53	0.000435	0.0130
GI_23503310-S	TAPT1	5	-0.100	-0.0118	-3.52	0.000440	0.0131
GI_41146530-S	---	5	-0.0984	-0.00337	-3.47	0.000530	0.0154
GI_37556035-S	---	5	-0.0980	-0.00458	-3.46	0.000549	0.0158
GI_14042942-S	TATDN1	5	-0.0978	-0.00606	-3.46	0.000562	0.0161
GI_37545958-S	RBM25	5	-0.0978	-0.00237	-3.44	0.000591	0.0167
GI_15149469-S	SUZ12	5	-0.0977	-0.00221	-3.44	0.000600	0.0169
GI_12056464-S	FBL	5	-0.0972	-0.00331	-3.43	0.000625	0.0175
GI_33946314-I	NIN	5	-0.0962	-0.00319	-3.40	0.000707	0.0193
GI_21614500-A	DEGS1	5	-0.0963	-0.00201	-3.39	0.000733	0.0198
GI_16117795-A	RPL36	5	-0.0966	-0.00157	-3.38	0.000741	0.0200
GI_29337287-S	YEATS4	5	-0.0950	-0.00289	-3.35	0.000833	0.0219
GI_15011942-S	TRIM2	5	0.0944	0.0141	3.34	0.000865	0.0225
GI_8922910-S	RSAD1	5	-0.0949	-0.00216	-3.34	0.000868	0.0226
GI_37544033-S	RPL7	5	-0.0937	-0.00617	-3.31	0.000958	0.0244
GI_14210505-S	ZC3H8	5	-0.0938	-0.00262	-3.30	0.000977	0.0248
GI_7661579-I	WIPI2	5	-0.0942	-0.00170	-3.30	0.000983	0.0249
GI_7706680-S	REV1	5	-0.0929	-0.00348	-3.28	0.001067	0.0266
GI_37622889-A	PLAGL1	5	-0.0926	-0.0152	-3.27	0.001090	0.0270
GI_42656857-S	SET	5	-0.0928	-0.00236	-3.27	0.001116	0.0275
GI_18373333-S	TMEM14B	5	-0.0924	-0.00340	-3.26	0.001141	0.0280
GI_19923618-S	EDEM3	5	-0.0923	-0.00363	-3.26	0.001157	0.0283
GI_31542314-S	CNOT8	5	-0.0923	-0.00204	-3.25	0.001205	0.0292
GI_41197110-S	---	5	-0.0921	-0.00218	-3.24	0.001218	0.0294
GI_4758559-S	SNRNP40	5	-0.0920	-0.00205	-3.24	0.001245	0.0299
GI_17482322-S	RPS15A	5	-0.0917	-0.00279	-3.23	0.001249	0.0300
GI_13376668-S	IFT74	5	-0.0915	-0.00431	-3.23	0.001264	0.0303
GI_9966792-S	LZTFL1	5	-0.0910	-0.00303	-3.21	0.001369	0.0322
GI_13435382-S	TRMT61B	5	-0.0908	-0.00489	-3.21	0.001373	0.0322
GI_29735784-S	PPP1R3E	5	-0.0902	-0.00572	-3.19	0.001479	0.0342
GI_37541551-S	RPL10	5	-0.0902	-0.00304	-3.18	0.001495	0.0345
GI_8923576-S	PAK1IP1	5	-0.0892	-0.00303	-3.15	0.001688	0.0380
GI_39725648-S	PAGR1	5	-0.0890	-0.00220	-3.13	0.001769	0.0394
Hs.448642-S	RPS25	5	-0.0886	-0.00616	-3.13	0.001786	0.0396
GI_4507656-S	TPP2	5	-0.0885	-0.00543	-3.13	0.001810	0.0400
GI_15718688-A	RPS3A	5	-0.0890	-0.00151	-3.12	0.001876	0.0412
GI_13376287-S	SETD6	5	-0.0875	-0.00259	-3.08	0.002094	0.0449
GI_4504190-S	MSH6	5	-0.0871	-0.00260	-3.07	0.002202	0.0466
GI_37546978-S	UQCRH	5	-0.0870	-0.00266	-3.07	0.002204	0.0466
GI_39653322-I	PHF20L1	5	-0.0868	-0.00377	-3.07	0.002223	0.0470
GI_17454506-S	---	5	-0.0868	-0.00275	-3.06	0.002264	0.0477
hmm14190-S	RPLP1	5	-0.0867	-0.00392	-3.06	0.002268	0.0477
GI_21359821-S	DROSHA	5	-0.0869	-0.00169	-3.05	0.002328	0.0486
GI_13775598-S	SIRT1	5	-0.0862	-0.00281	-3.04	0.002411	0.0499
GI_42544152-S	LTK	6	-0.299	-0.0149	-11.0	5.15E-27	1.35E-23
GI_5803202-S	TNNT3	6	-0.292	-0.0120	-10.8	7.76E-26	1.67E-22
GI_37548529-S	OBSCN	6	-0.287	-0.0123	-10.5	7.44E-25	1.41E-21
GI_4755145-S	AEBP1	6	-0.275	-0.0164	-10.0	6.96E-23	9.98E-20
GI_40255313-S	OBSCN	6	-0.267	-0.0443	-9.74	1.17E-21	1.32E-18
GI_38569429-S	ATF7IP2	6	-0.245	-0.00708	-8.88	2.38E-18	1.84E-15
GI_42518079-S	TCF7	6	-0.232	-0.00843	-8.39	1.32E-16	7.51E-14
GI_37537721-S	AGMAT	6	-0.222	-0.0182	-8.03	2.30E-15	1.10E-12
Hs.150495-S	---	6	0.220	0.0215	7.95	4.13E-15	1.88E-12
GI_37552337-S	ARRDC5	6	-0.217	-0.00502	-7.80	1.30E-14	5.34E-12
GI_20127439-S	MFSD10	6	0.218	0.00354	7.80	1.34E-14	5.45E-12
GI_5174556-S	MFGE8	6	-0.216	-0.00813	-7.76	1.71E-14	6.63E-12
GI_30150520-S	C1orf228	6	-0.214	-0.00611	-7.67	3.38E-14	1.26E-11
GI_4502010-S	AK1	6	0.208	0.00823	7.49	1.32E-13	4.53E-11
GI_18497287-S	LTBP3	6	-0.204	-0.0103	-7.32	4.33E-13	1.33E-10
GI_14249535-S	CIRH1A	6	-0.199	-0.00313	-7.07	2.59E-12	6.62E-10
GI_5453909-S	PLCD1	6	0.196	0.00663	7.01	3.87E-12	9.59E-10
GI_16507966-S	ENO2	6	0.193	0.00729	6.91	7.81E-12	1.84E-09
GI_31542262-S	SLC17A9	6	-0.184	-0.00504	-6.55	8.15E-11	1.53E-08
GI_10835100-S	KAT2A	6	-0.181	-0.00543	-6.47	1.39E-10	2.53E-08
GI_37577150-S	SPEG	6	-0.177	-0.0140	-6.34	3.17E-10	5.26E-08
GI_4557514-S	DDB2	6	0.170	0.00954	6.08	1.56E-09	2.26E-07
Hs.483403-S	---	6	-0.169	-0.00896	-6.03	2.18E-09	3.10E-07
GI_7656870-S	ZNF544	6	-0.169	-0.00408	-6.00	2.58E-09	3.57E-07
GI_22507400-S	MDS2	6	-0.168	-0.00960	-5.99	2.74E-09	3.75E-07
GI_23238255-A	CPT1B	6	0.168	0.00816	5.98	2.91E-09	3.93E-07
GI_31341765-S	LINC01089	6	-0.166	-0.00568	-5.90	4.59E-09	6.05E-07
GI_19882236-S	RHPN1	6	-0.162	-0.0129	-5.77	1.00E-08	1.22E-06
GI_25914748-S	ABCC10	6	0.163	0.00283	5.75	1.12E-08	1.35E-06
GI_24234723-A	TRADD	6	0.161	0.00316	5.72	1.36E-08	1.62E-06
GI_22748640-S	CHMP7	6	-0.160	-0.00523	-5.70	1.48E-08	1.74E-06
GI_15619005-S	VIPR1	6	-0.155	-0.00482	-5.53	3.98E-08	4.25E-06
GI_31982913-S	WDR54	6	0.153	0.00327	5.42	6.97E-08	7.11E-06
GI_7657350-S	MYBBP1A	6	-0.151	-0.00326	-5.35	1.03E-07	1.02E-05
GI_34147678-S	HOOK1	6	-0.149	-0.00793	-5.32	1.25E-07	1.19E-05
GI_13376651-S	ATHL1	6	-0.148	-0.00718	-5.25	1.80E-07	1.67E-05
GI_23200030-A	TNFRSF25	6	-0.147	-0.00594	-5.22	2.11E-07	1.94E-05
GI_44890064-S	GCFC2	6	-0.147	-0.00322	-5.21	2.20E-07	2.01E-05
GI_37537686-S	ZNF444	6	-0.141	-0.00301	-5.00	6.59E-07	5.31E-05
GI_21361764-S	FOXRED1	6	-0.141	-0.00257	-4.96	7.98E-07	6.26E-05
GI_44680152-S	GOLGA2P7	6	-0.139	-0.00598	-4.95	8.34E-07	6.49E-05

GI_12383073-S	TUT1	6	-0.140	-0.00308	-4.94	8.78E-07	6.80E-05
GI_34330189-S	HKR1	6	-0.137	-0.00381	-4.86	1.35E-06	9.89E-05
GI_9910257-S	SDR39U1	6	-0.137	-0.00232	-4.82	1.65E-06	0.000116
GI_13375816-S	NEIL1	6	-0.134	-0.00562	-4.76	2.20E-06	0.000150
GI_13376184-S	RBFA	6	-0.131	-0.00359	-4.63	4.00E-06	0.000256
GI_22027610-S	TRAF1	6	0.131	0.00481	4.63	4.02E-06	0.000256
GI_4504776-S	ITGB7	6	0.130	0.00307	4.59	4.88E-06	0.000304
GI_8923379-S	OSGEP	6	-0.130	-0.00180	-4.57	5.36E-06	0.000330
GI_14249125-S	GTPBP3	6	-0.129	-0.00273	-4.56	5.54E-06	0.000339
GI_34577119-S	MTRF1	6	-0.127	-0.00259	-4.49	7.62E-06	0.000456
GI_19923288-S	PIK3CD	6	-0.127	-0.00226	-4.46	9.10E-06	0.000535
GI_42560245-I	POLR1C	6	-0.125	-0.00250	-4.42	1.07E-05	0.000621
GI_23503298-S	PRR22	6	-0.125	-0.00592	-4.42	1.08E-05	0.000625
GI_42659267-S	RP11-395P17.3	6	-0.124	-0.00441	-4.40	1.19E-05	0.000678
GI_5453923-S	POLD2	6	-0.124	-0.00263	-4.37	1.36E-05	0.000757
GI_33598945-A	PLCG1	6	-0.122	-0.00397	-4.33	1.59E-05	0.000867
GI_26787961-A	AGER	6	-0.123	-0.00301	-4.33	1.62E-05	0.000883
GI_32261299-S	CYHR1	6	-0.121	-0.00414	-4.28	2.06E-05	0.00107
GI_42660343-S	DICER1-AS1	6	-0.117	-0.00407	-4.15	3.61E-05	0.00170
GI_7662449-S	FAN1	6	-0.117	-0.00315	-4.13	3.83E-05	0.00178
GI_41327778-S	DDX55	6	-0.118	-0.00204	-4.13	3.87E-05	0.00179
GI_22779933-S	WDR19	6	-0.116	-0.00292	-4.10	4.40E-05	0.00200
GI_20336200-I	---	6	-0.115	-0.00575	-4.08	4.75E-05	0.00213
GI_34101281-S	SCNN1D	6	-0.115	-0.00443	-4.08	4.81E-05	0.00215
GI_38708010-S	---	6	-0.115	-0.00248	-4.07	5.09E-05	0.00225
GI_40255084-S	UBQLNL	6	-0.113	-0.00884	-4.01	6.30E-05	0.00272
GI_4507546-S	TM7SF2	6	-0.113	-0.00360	-4.01	6.43E-05	0.00276
GI_7706484-S	TRAP1	6	-0.113	-0.00307	-4.00	6.82E-05	0.00289
GI_34147388-S	PTGES3L-AARSD1	6	-0.113	-0.00215	-3.98	7.44E-05	0.00310
GI_24307946-S	HABP4	6	0.111	0.00339	3.92	9.52E-05	0.00379
GI_33859830-S	POLR3C	6	-0.111	-0.00162	-3.90	0.000101	0.00398
GI_4885552-S	---	6	-0.110	-0.00237	-3.89	0.000107	0.00416
GI_11968044-S	INF2	6	-0.110	-0.00391	-3.88	0.000109	0.00424
GI_34147648-S	TLE2	6	-0.109	-0.00446	-3.85	0.000126	0.00476
GI_13376499-S	---	6	-0.107	-0.00303	-3.79	0.000160	0.00578
GI_37563696-S	MEI1	6	0.107	0.00269	3.79	0.000160	0.00581
GI_21717802-S	NSUN5P1	6	-0.107	-0.00273	-3.78	0.000167	0.00600
GI_4557650-S	HSF4	6	0.107	0.00361	3.77	0.000173	0.00619
GI_5453556-S	ARIH2	6	0.107	0.00264	3.76	0.000178	0.00634
GI_34147343-S	GRAMD1A	6	-0.105	-0.00247	-3.68	0.000240	0.00813
GI_4505462-S	CNTNAP1	6	0.104	0.00568	3.68	0.000245	0.00828
GI_9506614-S	PRMT7	6	-0.104	-0.00229	-3.68	0.000248	0.00834
GI_41204979-S	PEAK1	6	-0.104	-0.00232	-3.66	0.000262	0.00869
GI_34222253-A	P4HTM	6	0.104	0.00271	3.66	0.000264	0.00876
GI_20149710-S	SP140L	6	-0.104	-0.00213	-3.65	0.000274	0.00901
GI_8922734-S	ELP2	6	-0.104	-0.00174	-3.64	0.000282	0.00925
GI_37540723-S	RNF214	6	0.103	0.00201	3.63	0.000299	0.00971
GI_23312366-S	TNFRSF4	6	0.102	0.00479	3.62	0.000312	0.0101
GI_12232402-S	C16orf58	6	-0.102	-0.00274	-3.60	0.000336	0.0107
GI_8922412-S	ANKZF1	6	-0.102	-0.00241	-3.59	0.000338	0.0107
GI_30181232-S	SAP25	6	-0.101	-0.00412	-3.58	0.000355	0.0111
GI_42476295-S	TPM2	6	-0.100	-0.00638	-3.55	0.000397	0.0121
GI_27734876-S	TYSND1	6	-0.101	-0.00204	-3.55	0.000399	0.0122
GI_18105029-A	COLQ	6	-0.0995	-0.00443	-3.52	0.000455	0.0135
GI_37594443-S	ZMYND10	6	0.0991	0.00711	3.50	0.000474	0.0140
GI_41281526-S	PAN2	6	-0.100	-0.00208	-3.50	0.000479	0.0141
GI_40807460-S	ZNF335	6	0.0965	0.00535	3.41	0.000668	0.0185
GI_14149994-S	TMEM191A	6	0.0961	0.00250	3.39	0.000726	0.0197
GI_34147521-S	ADCK3	6	-0.0961	-0.00159	-3.37	0.000787	0.0210
GI_32698941-S	SLC25A45	6	-0.0947	-0.00337	-3.34	0.000854	0.0223
GI_37622344-A	MZF1	6	-0.0945	-0.00210	-3.32	0.000916	0.0236
GI_31455610-S	ZAP70	6	0.0936	0.00329	3.30	0.000986	0.0250
GI_4758291-S	EPHB6	6	-0.0930	-0.00342	-3.28	0.001055	0.0263
GI_8923393-S	TMEM161A	6	-0.0932	-0.00163	-3.27	0.001108	0.0274
GI_37655174-S	ZNF26	6	-0.0924	-0.00279	-3.26	0.001147	0.0281
GI_23346638-S	NPRL2	6	0.0928	0.00162	3.25	0.001177	0.0287
GI_37547280-S	MEGF6	6	-0.0919	-0.00431	-3.25	0.001196	0.0290
GI_38524588-S	PNPLA7	6	-0.0918	-0.00404	-3.24	0.001224	0.0295
GI_13376422-S	TMEM156	6	-0.0917	-0.00312	-3.24	0.001243	0.0299
GI_15149471-S	PBX4	6	0.0908	0.00362	3.21	0.001381	0.0323
GI_27480910-S	C9orf142	6	0.0912	0.00169	3.20	0.001406	0.0328
GI_27485260-S	PHLDB3	6	-0.0897	-0.00410	-3.17	0.001577	0.0360
GI_37595557-I	BTN3A1	6	0.0894	0.00206	3.15	0.001694	0.0381
GI_7661859-S	LRRC14	6	0.0888	0.00261	3.13	0.001785	0.0396
GI_4885224-I	EWSR1	6	-0.0891	-0.00149	-3.12	0.001850	0.0407
GI_39812452-S	SURF6	6	-0.0876	-0.00187	-3.08	0.002116	0.0452
GI_45439340-S	PPOX	6	-0.0867	-0.00316	-3.06	0.002286	0.0480
GI_21536366-A	TAF1C	6	-0.0868	-0.00165	-3.04	0.002382	0.0494
GI_4758865-S	NREP	7	-0.275	-0.00723	-10.0	6.95E-23	9.98E-20
GI_20336245-S	PCSK5	7	-0.185	-0.00500	-6.61	5.54E-11	1.10E-08
GI_31543835-S	NME8	7	0.180	0.00925	6.43	1.85E-10	3.25E-08
GI_7662259-S	KBTBD11	7	-0.179	-0.00770	-6.41	2.02E-10	3.53E-08
GI_41222688-S	RPL31P11	7	-0.178	-0.0101	-6.36	2.86E-10	4.83E-08
GI_13375757-S	C1orf54	7	-0.147	-0.00557	-5.21	2.23E-07	2.02E-05
GI_27734688-S	SSPO	7	-0.143	-0.0171	-5.10	3.95E-07	3.36E-05
GI_4506760-S	S100A10	7	0.142	0.00568	5.04	5.47E-07	4.48E-05
GI_8923223-I	LRRFIP2	7	0.141	0.00292	4.97	7.69E-07	6.06E-05

GI_27500713-S	ZIK1	7	-0.137	-0.00709	-4.88	1.20E-06	8.89E-05
GI_9845515-A	S100A4	7	0.136	0.00481	4.84	1.48E-06	0.000106
GI_8923764-S	CACNA2D3	7	-0.130	-0.00794	-4.63	4.04E-06	0.000258
GI_16418454-S	RBP7	7	-0.126	-0.00563	-4.48	7.99E-06	0.000476
GI_11037064-S	---	7	-0.125	-0.00482	-4.42	1.09E-05	0.000630
GI_17158002-S	ANTXR2	7	0.124	0.00246	4.38	1.29E-05	0.000726
GI_31747574-S	CLEC5A	7	-0.121	-0.00849	-4.30	1.88E-05	0.000997
GI_40018619-S	TBC1D2B	7	0.121	0.00218	4.26	2.16E-05	0.00111
GI_6912575-S	PADI4	7	-0.120	-0.00455	-4.24	2.42E-05	0.00122
GI_7662375-S	FAM13A	7	0.117	0.00483	4.14	3.71E-05	0.00173
GI_22035639-S	MGST2	7	-0.113	-0.00300	-4.00	6.59E-05	0.00281
GI_33186919-S	---	7	-0.113	-0.00908	-4.00	6.79E-05	0.00288
GI_40255259-S	PID1	7	-0.113	-0.00645	-3.99	7.07E-05	0.00298
GI_4557574-S	FAAH	7	-0.112	-0.00544	-3.95	8.20E-05	0.00335
GI_21314670-S	TRPM4	7	-0.111	-0.00800	-3.95	8.36E-05	0.00340
GI_22095361-S	GLIPR2	7	0.110	0.00264	3.87	0.000113	0.00433
GI_33589822-S	PDK4	7	0.109	0.00672	3.87	0.000114	0.00437
GI_4758707-S	LY86	7	-0.108	-0.00274	-3.82	0.000139	0.00516
GI_27885012-S	C19orf59	7	0.108	0.00418	3.81	0.000147	0.00543
Hs.466809-S	---	7	-0.107	-0.00569	-3.77	0.000168	0.00604
hmm5973-S	MYO15B	7	-0.106	-0.00743	-3.74	0.000195	0.00685
GI_25072198-S	C11orf82	7	-0.105	-0.00557	-3.72	0.000208	0.00720
GI_31377572-S	DUSP18	7	0.100	0.00455	3.53	0.000432	0.0129
GI_30158043-S	PTPRJ	7	0.0993	0.00313	3.51	0.000472	0.0139
GI_19718745-A	OSBPL1A	7	-0.0991	-0.00383	-3.50	0.000480	0.0141
GI_19923275-S	---	7	-0.0981	-0.0148	-3.47	0.000541	0.0156
GI_21536270-I	MPZL2	7	-0.0972	-0.00522	-3.44	0.000607	0.0171
GI_37552472-S	CLN8	7	-0.0968	-0.00372	-3.42	0.000647	0.0180
GI_11038652-S	AQP9	7	0.0962	0.00506	3.40	0.000696	0.0190
GI_5713320-S	SELL	7	-0.0961	-0.00441	-3.39	0.000711	0.0194
GI_31317229-S	FCER1A	7	-0.0958	-0.00628	-3.39	0.000731	0.0198
GI_13375978-S	BAHCC1	7	-0.0949	-0.00839	-3.36	0.000817	0.0216
GI_31542726-S	CPED1	7	-0.0947	-0.00750	-3.35	0.000834	0.0220
GI_45243500-S	BCL2L13	7	-0.0952	-0.00156	-3.34	0.000876	0.0228
GI_22749220-S	SPRED1	7	0.0931	0.00659	3.29	0.001030	0.0258
GI_23308566-S	ASRGL1	7	-0.0931	-0.00731	-3.29	0.001031	0.0258
GI_30181235-S	CPNE2	7	-0.0930	-0.00314	-3.28	0.001058	0.0264
GI_37674209-S	SSH2	7	-0.0933	-0.00194	-3.28	0.001066	0.0266
GI_38348277-S	VSTM1	7	-0.0924	-0.0161	-3.27	0.001116	0.0275
GI_31543440-S	---	7	-0.0904	-0.00192	-3.18	0.001518	0.0349
GI_19747277-S	RHOU	7	0.0897	0.00251	3.16	0.001604	0.0365
Hs.525303-S	---	7	-0.0875	-0.00825	-3.09	0.002032	0.0439
GI_22749378-S	GAPT	7	-0.0871	-0.00362	-3.08	0.002150	0.0457
GI_19923329-S	CTDSP2	7	-0.0872	-0.00149	-3.05	0.002307	0.0484
GI_23397450-S	AMICA1	7	-0.0865	-0.00346	-3.05	0.002328	0.0486
Hs.233167-S	---	8	-0.244	-0.0179	-8.87	2.60E-18	1.97E-15
GI_30154358-S	AGPAT4	8	0.192	0.00534	6.86	1.08E-11	2.45E-09
Hs.185140-S	IPCEF1	8	-0.168	-0.00681	-6.00	2.55E-09	3.56E-07
Hs.370906-S	---	8	-0.162	-0.00943	-5.77	1.03E-08	1.25E-06
Hs.45184-S	---	8	-0.160	-0.00725	-5.70	1.45E-08	1.72E-06
Hs.397082-S	---	8	-0.153	-0.00779	-5.43	6.64E-08	6.81E-06
hmm15104-S	SLC18B1	8	-0.147	-0.00366	-5.21	2.15E-07	1.97E-05
Hs.443639-S	---	8	-0.146	-0.00731	-5.19	2.40E-07	2.16E-05
Hs.436457-S	---	8	0.145	0.0222	5.15	3.04E-07	2.67E-05
Hs.291277-S	---	8	-0.136	-0.00621	-4.83	1.52E-06	0.000108
GI_20475100-S	---	8	0.133	0.00652	4.71	2.69E-06	0.000180
Hs.119065-S	---	8	-0.132	-0.00606	-4.68	3.13E-06	0.000206
Hs.395295-S	---	8	-0.129	-0.00591	-4.59	4.85E-06	0.000303
Hs.437188-S	---	8	-0.127	-0.00804	-4.50	7.60E-06	0.000456
GI_22749246-S	CNST	8	-0.120	-0.00380	-4.24	2.42E-05	0.00122
hmm23672-S	---	8	-0.119	-0.00555	-4.22	2.65E-05	0.00132
Hs.12346-S	---	8	-0.117	-0.00359	-4.13	3.94E-05	0.00181
GI_42659368-S	PDCD11	8	-0.116	-0.00427	-4.10	4.33E-05	0.00197
Hs.348088-S	---	8	0.116	0.00680	4.10	4.50E-05	0.00203
GI_29738101-S	---	8	-0.115	-0.00520	-4.06	5.21E-05	0.00230
Hs.452445-S	---	8	-0.115	-0.00619	-4.06	5.23E-05	0.00230
Hs.465651-S	---	8	-0.113	-0.00528	-4.01	6.50E-05	0.00279
Hs.145444-S	---	8	-0.108	-0.00484	-3.83	0.000135	0.00504
Hs.171301-S	---	8	0.108	0.00645	3.83	0.000136	0.00509
hmm2974-S	MCF2L	8	-0.108	-0.00540	-3.81	0.000148	0.00545
GI_4507810-S	UGCG	8	-0.107	-0.00335	-3.80	0.000154	0.00562
hmm11379-S	RAB3GAP1	8	-0.107	-0.00307	-3.76	0.000176	0.00629
Hs.452714-S	---	8	-0.104	-0.00614	-3.69	0.000234	0.00794
Hs.472282-S	---	8	-0.104	-0.00441	-3.67	0.000248	0.00836
Hs.378028-S	---	8	-0.104	-0.00605	-3.67	0.000252	0.00846
GI_29747364-S	---	8	-0.103	-0.00460	-3.64	0.000288	0.00942
GI_14149666-S	SARM1	8	0.102	0.00485	3.62	0.000303	0.00981
GI_41114517-S	RPS16	8	-0.101	-0.00329	-3.56	0.000391	0.0120
GI_27485604-S	MAVS	8	-0.0994	-0.00367	-3.51	0.000465	0.0138
Hs.497573-S	DICER1-AS1	8	-0.0987	-0.00443	-3.49	0.000507	0.0148
GI_42662645-S	ZNF275	8	-0.0988	-0.00292	-3.49	0.000508	0.0148
Hs.348319-S	---	8	0.0970	0.00449	3.43	0.000627	0.0175
GI_12232372-S	RABGAP1	8	-0.0964	-0.00152	-3.38	0.000759	0.0203
Hs.368169-S	---	8	0.0950	0.00507	3.36	0.000808	0.0214
Hs.194417-S	---	8	-0.0947	-0.00378	-3.35	0.000845	0.0222
Hs.508413-S	---	8	-0.0940	-0.00680	-3.32	0.000921	0.0237
hmm19663-S	JMJD1C	8	-0.0932	-0.00312	-3.29	0.001036	0.0259

GI_7661625-S	SERBP1	8	-0.0934	-0.00152	-3.27	0.001105	0.0274
Hs.445048-S	---	8	-0.0921	-0.00285	-3.25	0.001200	0.0291
Hs.459570-S	ANKRD18A	8	0.0914	0.00441	3.23	0.001280	0.0305
Hs.473369-S	---	8	0.0914	0.00454	3.23	0.001282	0.0305
GI_27499805-S	LINC00641	8	-0.0909	-0.00354	-3.21	0.001368	0.0321
Hs.471449-S	---	8	-0.0903	-0.00711	-3.19	0.001455	0.0338
GI_5902159-S	ZNF22	8	-0.0904	-0.00283	-3.19	0.001470	0.0340
Hs.192103-S	---	8	-0.0901	-0.00309	-3.18	0.001513	0.0348
Hs.296713-S	---	8	-0.0897	-0.00433	-3.17	0.001567	0.0358
Hs.283500-S	---	8	0.0894	0.00277	3.15	0.001658	0.0375
GI_38502313-S	C22orf34	8	-0.0890	-0.00582	-3.14	0.001703	0.0383
Hs.12867-S	---	8	-0.0882	-0.00554	-3.12	0.001878	0.0412
Hs.311881-S	---	8	0.0872	0.00694	3.08	0.002097	0.0449
GI_13186319-A	METTL1	9	-0.273	-0.00628	-9.93	2.07E-22	2.65E-19
GI_6857817-S	NPM3	9	-0.257	-0.00850	-9.35	4.07E-20	3.69E-17
GI_38045911-A	NME1	9	-0.214	-0.00468	-7.65	3.97E-14	1.45E-11
GI_22748804-S	C1orf216	9	0.210	0.00803	7.54	8.91E-14	3.17E-11
GI_32171195-S	CENPV	9	-0.207	-0.00647	-7.44	1.83E-13	6.09E-11
GI_42476209-S	TMCO4	9	0.202	0.00348	7.21	9.61E-13	2.75E-10
GI_34147590-S	PUS1	9	-0.202	-0.00327	-7.20	1.06E-12	2.99E-10
GI_8923076-S	ZNF586	9	-0.193	-0.00441	-6.89	8.79E-12	2.04E-09
GI_34222270-S	SFXN2	9	-0.192	-0.00817	-6.89	9.10E-12	2.10E-09
GI_4504688-S	IMPDH2	9	-0.190	-0.00385	-6.76	2.09E-11	4.57E-09
GI_31657128-S	PFAS	9	-0.184	-0.00548	-6.57	7.23E-11	1.37E-08
GI_42542387-S	SFXN4	9	-0.182	-0.00480	-6.49	1.25E-10	2.31E-08
GI_11993934-S	CHI3L2	9	-0.176	-0.00741	-6.29	4.47E-10	7.15E-08
GI_41349477-S	PRDM7	9	-0.173	-0.00397	-6.14	1.11E-09	1.64E-07
GI_4885584-S	SAE1	9	-0.174	-0.00259	-6.13	1.18E-09	1.74E-07
GI_38348207-S	C1orf122	9	0.166	0.00280	5.86	5.85E-09	7.44E-07
GI_16554601-S	MRPS18B	9	-0.160	-0.00300	-5.67	1.82E-08	2.09E-06
GI_41352062-S	PFKP	9	0.158	0.00409	5.60	2.71E-08	3.02E-06
GI_4885080-S	ATP5G1	9	-0.157	-0.00246	-5.53	3.92E-08	4.23E-06
GI_21361347-S	UTP14A	9	-0.153	-0.00240	-5.40	7.90E-08	8.00E-06
GI_40317613-A	MRRF	9	-0.151	-0.00225	-5.32	1.21E-07	1.17E-05
GI_9558742-S	CCDC106	9	-0.150	-0.00506	-5.32	1.22E-07	1.17E-05
GI_4507208-S	SRM	9	-0.150	-0.00338	-5.31	1.33E-07	1.26E-05
GI_11968046-S	POLR1E	9	-0.150	-0.00308	-5.30	1.39E-07	1.31E-05
GI_22035587-A	MRPL24	9	-0.148	-0.00408	-5.24	1.90E-07	1.75E-05
GI_7706271-S	DPH5	9	-0.147	-0.00362	-5.22	2.05E-07	1.88E-05
GI_13435349-A	FDXR	9	0.146	0.00403	5.19	2.46E-07	2.21E-05
GI_14150140-S	PDCD2L	9	-0.146	-0.00452	-5.18	2.55E-07	2.28E-05
GI_31543400-S	PIM1	9	0.141	0.00418	5.01	6.22E-07	5.05E-05
GI_28559082-A	CTPS2	9	-0.139	-0.00423	-4.94	8.68E-07	6.74E-05
GI_20070219-S	PRMT5	9	-0.140	-0.00268	-4.93	9.32E-07	7.17E-05
GI_39725941-S	C10orf2	9	-0.136	-0.00807	-4.84	1.49E-06	0.000107
GI_27478147-S	PTRH1	9	0.135	0.00695	4.78	1.92E-06	0.000132
GI_24430136-S	LAGE3	9	-0.135	-0.00283	-4.77	2.09E-06	0.000143
GI_40353202-S	FAM219B	9	0.133	0.00203	4.68	3.14E-06	0.000206
GI_19593984-S	SURF4	9	0.133	0.00211	4.68	3.15E-06	0.000206
GI_20127554-S	---	9	-0.132	-0.00279	-4.66	3.45E-06	0.000225
GI_7657121-S	GEMIN4	9	-0.132	-0.00334	-4.66	3.44E-06	0.000225
GI_10835020-S	IGFBP4	9	-0.128	-0.00424	-4.55	6.03E-06	0.000367
GI_40254875-S	PDZD11	9	0.125	0.00219	4.40	1.18E-05	0.000671
GI_22035625-S	CDS2	9	0.125	0.00213	4.39	1.21E-05	0.000687
GI_22095396-S	CRELD1	9	-0.124	-0.00307	-4.38	1.31E-05	0.000737
GI_14249519-S	FAM136A	9	-0.123	-0.00261	-4.35	1.45E-05	0.000802
GI_17975596-S	SELM	9	0.122	0.00491	4.31	1.78E-05	0.000950
GI_21614498-S	EZR	9	0.121	0.00377	4.28	2.03E-05	0.00106
GI_7662339-S	MORC2	9	-0.121	-0.00309	-4.28	2.05E-05	0.00107
GI_11386190-S	EIF2D	9	-0.121	-0.00330	-4.26	2.18E-05	0.00112
GI_15011919-S	NHP2	9	-0.121	-0.00206	-4.26	2.23E-05	0.00114
GI_40217837-I	WDR4	9	-0.119	-0.00326	-4.22	2.66E-05	0.00132
GI_34577121-S	NFKB1	9	-0.119	-0.00214	-4.19	2.96E-05	0.00144
GI_27498545-S	CCDC167	9	0.117	0.00336	4.13	3.89E-05	0.00180
GI_15431296-A	RPL13	9	-0.116	-0.00309	-4.08	4.69E-05	0.00211
GI_7706199-S	THEM6	9	-0.114	-0.00338	-4.03	5.84E-05	0.00254
GI_23110931-A	PSMB9	9	0.114	0.00253	4.03	5.86E-05	0.00254
GI_23199997-A	NSUN5	9	-0.112	-0.00205	-3.95	8.30E-05	0.00338
GI_38327035-S	HAGH	9	0.111	0.00255	3.92	9.41E-05	0.00375
GI_38683842-S	DNPH1	9	-0.109	-0.00296	-3.84	0.000130	0.00488
GI_13904869-S	RPS5	9	-0.108	-0.00254	-3.82	0.000141	0.00524
GI_31543422-S	POLE3	9	-0.109	-0.00142	-3.81	0.000148	0.00545
GI_32698695-S	TMEM97	9	-0.108	-0.00319	-3.80	0.000150	0.00550
GI_10835092-S	PTTG2	9	0.107	0.00392	3.80	0.000154	0.00562
GI_4759187-S	STX8	9	0.108	0.00179	3.79	0.000158	0.00573
GI_33356546-S	MCM2	9	-0.107	-0.00313	-3.77	0.000170	0.00610
GI_39812361-S	PSMD8	9	0.107	0.00166	3.74	0.000192	0.00675
GI_37059745-S	PEX5	9	-0.106	-0.00148	-3.72	0.000208	0.00719
GI_38679979-A	ACACA	9	-0.105	-0.00204	-3.71	0.000217	0.00747
GI_4885062-S	ALDOC	9	-0.104	-0.00319	-3.67	0.000256	0.00855
GI_24308523-S	TMEM9	9	-0.104	-0.00184	-3.65	0.000269	0.00888
GI_30795226-S	DTD1	9	0.102	0.00300	3.58	0.000350	0.0110
GI_14917112-S	SRPRB	9	-0.100	-0.00195	-3.52	0.000446	0.0133
GI_12751492-S	PYCRL	9	-0.0991	-0.00383	-3.50	0.000481	0.0141
GI_22749340-S	FAM76A	9	0.0985	0.00245	3.47	0.000536	0.0155
GI_39725632-S	EXOSC7	9	-0.0987	-0.00166	-3.46	0.000558	0.0160
GI_37540621-S	NOP14	9	-0.0982	-0.00161	-3.44	0.000602	0.0169

GI_7657197-S	DIMT1	9	-0.0980	-0.00165	-3.43	0.000614	0.0172
GI_5032046-S	POP7	9	0.0977	0.00180	3.43	0.000623	0.0174
GI_34147613-S	OBFC1	9	0.0971	0.00172	3.41	0.000682	0.0187
GI_21361595-S	TCEAL4	9	-0.0960	-0.00190	-3.37	0.000765	0.0204
GI_41872630-S	FASN	9	-0.0937	-0.00303	-3.31	0.000969	0.0246
GI_41393591-S	ADCK2	9	0.0940	0.00179	3.30	0.000988	0.0250
GI_21361279-S	TSFM	9	-0.0940	-0.00160	-3.29	0.001018	0.0255
GI_24942125-S	SHMT2	9	-0.0935	-0.00163	-3.28	0.001069	0.0266
GI_17986251-I	SP110	9	-0.0929	-0.00148	-3.25	0.001181	0.0287
GI_38016923-S	PRKAB1	9	0.0917	0.00259	3.23	0.001266	0.0303
GI_41107699-S	RPL18A	9	-0.0904	-0.00197	-3.18	0.001524	0.0350
GI_29741265-S	ETV2	9	0.0883	0.00418	3.12	0.001860	0.0409
GI_42659182-S	SMU1	9	-0.0885	-0.00193	-3.11	0.001898	0.0416
GI_21735547-S	CEP250	9	0.0877	0.00245	3.09	0.002055	0.0443
GI_19923786-S	RER1	9	0.0882	0.00130	3.08	0.002105	0.0450
GI_31455613-S	DERL2	9	0.0870	0.00179	3.06	0.002289	0.0480
GI_14150188-S	HVCN1	9	-0.0866	-0.00288	-3.05	0.002318	0.0485
GI_31560867-S	ITM2C	10	-0.332	-0.0122	-12.3	4.01E-33	2.11E-29
GI_31982900-S	SCD	10	-0.275	-0.0103	-10.0	7.27E-23	1.01E-19
GI_22547194-S	SREBF1	10	-0.272	-0.00832	-9.93	2.14E-22	2.66E-19
GI_27552763-S	TMEM8B	10	-0.219	-0.00595	-7.89	6.47E-15	2.86E-12
GI_42544137-S	EXOSC5	10	-0.204	-0.00560	-7.30	5.28E-13	1.60E-10
GI_23238206-S	TNFRSF21	10	-0.202	-0.0363	-7.28	6.01E-13	1.80E-10
GI_12545379-S	SCT	10	-0.196	-0.0331	-7.04	3.25E-12	8.19E-10
GI_41199582-S	LRRC26	10	-0.194	-0.00900	-6.96	5.56E-12	1.34E-09
GI_24308228-S	PACSIN1	10	-0.192	-0.0275	-6.88	9.38E-12	2.15E-09
GI_40804463-S	LAMP5	10	-0.187	-0.00954	-6.70	3.25E-11	6.83E-09
GI_42794770-A	---	10	-0.185	-0.0107	-6.64	4.74E-11	9.57E-09
GI_18677769-S	C5orf20	10	-0.184	-0.0102	-6.60	6.13E-11	1.19E-08
GI_38454325-S	CD38	10	-0.177	-0.0112	-6.31	3.86E-10	6.27E-08
GI_39725933-S	SERPINF1	10	-0.176	-0.0181	-6.30	4.19E-10	6.79E-08
GI_6006016-S	LGALS3BP	10	-0.164	-0.00870	-5.85	6.25E-09	7.91E-07
GI_21914880-S	LGMN	10	-0.162	-0.0150	-5.79	9.04E-09	1.11E-06
Hs.149078-S	---	10	-0.159	-0.0170	-5.67	1.77E-08	2.04E-06
GI_32483415-S	NEFH	10	-0.158	-0.0142	-5.64	2.11E-08	2.39E-06
GI_14574565-I	AIF1	10	-0.155	-0.00907	-5.51	4.35E-08	4.64E-06
GI_42658673-S	LINC00996	10	-0.153	-0.0232	-5.46	5.81E-08	6.04E-06
GI_22748696-S	C12orf45	10	-0.150	-0.00498	-5.33	1.16E-07	1.14E-05
GI_45580689-S	CLEC4C	10	-0.150	-0.0235	-5.32	1.20E-07	1.16E-05
GI_5803120-S	PDIA5	10	-0.144	-0.00764	-5.12	3.53E-07	3.04E-05
GI_31652260-S	MYBL2	10	-0.142	-0.0184	-5.04	5.35E-07	4.39E-05
GI_30179901-S	SOX4	10	-0.141	-0.00500	-5.02	6.07E-07	4.94E-05
GI_37539476-S	PPM1J	10	-0.141	-0.0112	-5.00	6.52E-07	5.27E-05
GI_31542664-S	LRRC36	10	-0.141	-0.0238	-5.00	6.63E-07	5.32E-05
GI_5803026-S	IFI44L	10	-0.140	-0.0107	-4.97	7.52E-07	5.93E-05
GI_18490989-S	MX1	10	-0.139	-0.00639	-4.93	9.41E-07	7.21E-05
GI_4826697-S	DNASE1L3	10	-0.138	-0.0102	-4.90	1.08E-06	8.10E-05
GI_42661292-S	SCD	10	-0.138	-0.0221	-4.90	1.09E-06	8.18E-05
Hs.458273-S	MT1L	10	0.128	0.00845	4.52	6.64E-06	0.000401
GI_17149847-S	FKBP5	10	-0.127	-0.00427	-4.48	7.97E-06	0.000475
Hs.203697-S	---	10	-0.125	-0.00765	-4.45	9.49E-06	0.000554
GI_25014108-S	C11orf31	10	-0.126	-0.00245	-4.43	1.05E-05	0.000607
GI_39930600-S	ALKBH7	10	-0.124	-0.00220	-4.38	1.30E-05	0.000729
GI_32313609-S	USP18	10	-0.122	-0.0128	-4.34	1.52E-05	0.000836
GI_11496992-S	PARP3	10	0.121	0.00580	4.30	1.83E-05	0.000972
GI_22050996-S	PALD1	10	-0.121	-0.0174	-4.30	1.84E-05	0.000975
GI_34486095-S	RPL28	10	-0.121	-0.00324	-4.29	1.93E-05	0.00102
GI_11386142-S	SERPINF2	10	-0.119	-0.0168	-4.23	2.47E-05	0.00124
GI_20143965-A	KIF23	10	-0.118	-0.0106	-4.20	2.85E-05	0.00140
GI_6912245-S	CD3EAP	10	-0.118	-0.00747	-4.18	3.12E-05	0.00151
GI_20302170-A	TLR9	10	-0.118	-0.0112	-4.18	3.18E-05	0.00153
GI_29171688-S	LILRA4	10	-0.112	-0.0205	-3.98	7.26E-05	0.00303
GI_21914861-A	CECR5	10	-0.113	-0.00254	-3.97	7.52E-05	0.00312
Hs.125087-S	IFI44L	10	-0.111	-0.0189	-3.93	9.01E-05	0.00363
GI_4504584-S	IFIT1	10	-0.111	-0.0114	-3.93	9.08E-05	0.00365
GI_4505672-S	PDE6G	10	-0.110	-0.00936	-3.91	9.62E-05	0.00382
GI_24041025-S	NETO2	10	-0.110	-0.0163	-3.90	0.000100	0.00396
GI_9951914-S	AHCY	10	-0.110	-0.00227	-3.86	0.000120	0.00459
GI_22538815-S	CCL8	10	-0.108	-0.0184	-3.84	0.000129	0.00486
GI_4758893-S	PET112	10	-0.108	-0.00687	-3.81	0.000146	0.00538
GI_4506812-S	SCN9A	10	-0.106	-0.0171	-3.77	0.000170	0.00610
GI_31542717-S	ECHDC3	10	-0.106	-0.0152	-3.75	0.000182	0.00648
GI_20336295-S	DDX28	10	-0.105	-0.00275	-3.72	0.000209	0.00720
GI_14150184-S	ZBED3	10	-0.103	-0.00898	-3.66	0.000264	0.00876
GI_37541363-S	RSL1D1	10	-0.104	-0.00402	-3.66	0.000265	0.00879
GI_7706082-S	C1RL	10	0.103	0.00273	3.62	0.000306	0.00988
GI_21361309-S	IFI44	10	-0.101	-0.00549	-3.57	0.000374	0.0116
GI_4557436-S	CDC20	10	-0.100	-0.0127	-3.53	0.000425	0.0128
GI_16418460-S	TOP1MT	10	-0.100	-0.00656	-3.53	0.000431	0.0129
GI_34303916-S	FOXRED2	10	-0.0990	-0.00863	-3.50	0.000478	0.0141
GI_38570053-S	LYRM4	10	-0.0971	-0.0120	-3.43	0.000616	0.0173
GI_13162281-S	STS	10	0.0960	0.00453	3.39	0.000713	0.0194
GI_17921981-S	COX10	10	-0.0961	-0.00300	-3.39	0.000725	0.0197
GI_25952086-S	KCNA5	10	-0.0945	-0.0148	-3.34	0.000860	0.0225
GI_42734367-S	DPH6	10	-0.0943	-0.00704	-3.33	0.000880	0.0228
GI_34147519-S	STX6	10	0.0943	0.00417	3.33	0.000889	0.0230
GI_44890060-S	PPIB	10	-0.0946	-0.00193	-3.33	0.000905	0.0233

GI_37547044-S	C1orf228	10	-0.0933	-0.00753	-3.30	0.001006	0.0253
GI_27735126-S	SLC35F3	10	-0.0932	-0.0151	-3.30	0.001008	0.0254
GI_16950627-I	AP1S1	10	0.0919	0.00511	3.25	0.001204	0.0292
GI_17025229-S	KCNK17	10	-0.0917	-0.0159	-3.24	0.001216	0.0293
GI_26787980-A	IL15RA	10	0.0900	0.0119	3.18	0.001506	0.0347
GI_7669493-A	GCDH	10	-0.0898	-0.00502	-3.17	0.001542	0.0354
GI_31342325-S	NMNAT3	10	-0.0892	-0.00661	-3.15	0.001656	0.0375
GI_4557342-S	ALDH7A1	10	-0.0884	-0.0109	-3.12	0.001831	0.0404
GI_27881483-S	ZC3H7B	10	0.0883	0.0109	3.12	0.001844	0.0406
GI_21040270-S	NLRP7	10	-0.0879	-0.00992	-3.11	0.001938	0.0422
GI_4759305-S	LIN7A	10	0.0877	0.0126	3.10	0.001984	0.0431
GI_13775237-S	PLVAP	10	-0.0869	-0.00800	-3.07	0.002187	0.0464
GI_41352698-S	WARS2	10	-0.0866	-0.00442	-3.06	0.002267	0.0477
GI_16306577-A	FBXL6	10	0.0862	0.00427	3.04	0.002386	0.0495
GI_41281388-S	NRCAM	11	-0.381	-0.0687	-14.5	3.37E-44	3.98E-40
GI_38564321-S	ZNF154	11	-0.326	-0.0258	-12.2	3.37E-32	1.59E-28
GI_23308576-S	PHGDH	11	-0.291	-0.0241	-10.7	1.46E-25	3.00E-22
GI_4503664-S	FBLN2	11	-0.277	-0.0283	-10.2	2.23E-23	3.40E-20
GI_14589910-A	MMP28	11	-0.247	-0.0389	-8.96	1.16E-18	9.13E-16
GI_29747011-S	PLEKHG4	11	-0.242	-0.0450	-8.79	5.02E-18	3.44E-15
GI_22749362-S	FBXO15	11	-0.229	-0.0379	-8.26	3.57E-16	1.92E-13
GI_15011978-A	ARHGEF4	11	-0.225	-0.0398	-8.11	1.19E-15	5.98E-13
GI_21389428-S	TMIGD2	11	-0.217	-0.0114	-7.83	1.06E-14	4.49E-12
GI_37549771-S	TTC24	11	-0.217	-0.0317	-7.82	1.15E-14	4.83E-12
GI_14165275-S	C2orf40	11	-0.216	-0.0298	-7.77	1.61E-14	6.29E-12
GI_10863910-S	SPINK2	11	-0.212	-0.0357	-7.66	3.86E-14	1.43E-11
GI_28144896-A	AK5	11	-0.212	-0.0359	-7.65	4.02E-14	1.46E-11
GI_38788425-S	NOG	11	-0.204	-0.0335	-7.34	3.75E-13	1.17E-10
Hs.500643-S	RNF157-AS1	11	-0.202	-0.0230	-7.27	6.54E-13	1.94E-10
GI_31377628-S	CPA5	11	-0.202	-0.0338	-7.25	7.22E-13	2.12E-10
hmm3032-S	---	11	-0.201	-0.0312	-7.23	8.71E-13	2.51E-10
GI_7110714-S	SEC14L2	11	-0.200	-0.0318	-7.19	1.13E-12	3.18E-10
GI_19743835-S	TARBP1	11	-0.200	-0.00658	-7.17	1.29E-12	3.55E-10
GI_34304373-S	CDK5R1	11	-0.200	-0.00924	-7.17	1.32E-12	3.60E-10
GI_42658866-S	MTUS1	11	-0.199	-0.0316	-7.14	1.56E-12	4.12E-10
GI_37552330-S	ZFR2	11	-0.185	-0.0266	-6.61	5.68E-11	1.12E-08
GI_4502798-S	CHAD	11	-0.181	-0.0169	-6.48	1.29E-10	2.36E-08
GI_4505854-S	PLAG1	11	-0.181	-0.0186	-6.47	1.38E-10	2.52E-08
GI_8051576-A	ABCG1	11	-0.179	-0.00959	-6.39	2.31E-10	3.94E-08
GI_37540666-S	GPR125	11	-0.178	-0.0237	-6.35	2.99E-10	5.02E-08
GI_29789280-S	TSPYL5	11	-0.177	-0.0240	-6.33	3.42E-10	5.62E-08
GI_34147323-S	SERPINE2	11	-0.176	-0.0266	-6.29	4.44E-10	7.13E-08
GI_34419632-S	ZNF135	11	-0.171	-0.0274	-6.11	1.31E-09	1.91E-07
GI_34222164-S	ZNF502	11	-0.168	-0.0172	-5.98	2.87E-09	3.89E-07
GI_16933556-S	DCHS1	11	-0.167	-0.0267	-5.96	3.22E-09	4.34E-07
Hs.449572-S	---	11	-0.162	-0.0313	-5.78	9.29E-09	1.14E-06
GI_18593550-S	---	11	-0.161	-0.0135	-5.73	1.26E-08	1.51E-06
GI_17975754-I	MSL3	11	-0.160	-0.00310	-5.66	1.91E-08	2.17E-06
GI_32698741-S	CACHD1	11	-0.156	-0.0239	-5.54	3.60E-08	3.90E-06
hmm35424-S	---	11	-0.155	-0.0239	-5.51	4.43E-08	4.71E-06
GI_37540914-S	HYKK	11	-0.154	-0.0180	-5.47	5.36E-08	5.64E-06
GI_37540696-S	ROBO3	11	-0.153	-0.00941	-5.46	5.60E-08	5.86E-06
GI_14249635-S	TOX2	11	-0.153	-0.0269	-5.46	5.85E-08	6.07E-06
GI_30153083-S	---	11	-0.153	-0.0242	-5.45	6.14E-08	6.34E-06
GI_32528300-S	EPHB4	11	-0.152	-0.0121	-5.41	7.71E-08	7.82E-06
GI_21389548-S	PSMA8	11	-0.151	-0.0241	-5.38	9.08E-08	9.09E-06
GI_18597568-S	---	11	-0.150	-0.0233	-5.32	1.20E-07	1.16E-05
GI_39932584-S	STOX1	11	-0.144	-0.0235	-5.14	3.19E-07	2.78E-05
GI_30794499-S	KDM1A	11	-0.144	-0.00226	-5.06	4.72E-07	3.91E-05
GI_37574602-S	ZNF256	11	-0.140	-0.00953	-4.98	7.15E-07	5.69E-05
Hs.191828-S	---	11	-0.140	-0.0149	-4.96	7.93E-07	6.23E-05
Hs.293510-S	---	11	-0.137	-0.0258	-4.88	1.20E-06	8.89E-05
GI_11641230-S	EDAR	11	-0.137	-0.00845	-4.85	1.40E-06	0.000102
GI_38566701-S	ZNF285	11	-0.136	-0.0160	-4.83	1.52E-06	0.000108
Hs.289062-S	---	11	-0.136	-0.0159	-4.83	1.53E-06	0.000108
GI_13435360-A	DSC1	11	-0.135	-0.0228	-4.79	1.83E-06	0.000127
GI_27886606-A	PTK7	11	-0.134	-0.0198	-4.77	2.06E-06	0.000142
GI_31543612-S	SCML1	11	-0.134	-0.0120	-4.77	2.08E-06	0.000143
GI_40788000-I	SPRY1	11	-0.133	-0.0105	-4.71	2.71E-06	0.000180
GI_37546955-S	DNAH6	11	-0.132	-0.0110	-4.70	2.94E-06	0.000195
GI_19923369-S	KDM5B	11	-0.133	-0.00335	-4.70	2.96E-06	0.000196
GI_29171718-A	GPLD1	11	-0.131	-0.0203	-4.67	3.40E-06	0.000222
GI_31377838-S	ZNF606	11	-0.131	-0.0213	-4.64	3.84E-06	0.000248
Hs.158943-S	LOC100131289	11	-0.130	-0.0199	-4.62	4.26E-06	0.000270
GI_20558478-S	---	11	0.129	0.0184	4.59	4.96E-06	0.000308
GI_21284386-I	ABLIM1	11	-0.129	-0.0178	-4.58	5.23E-06	0.000323
GI_44889961-A	STMN1	11	-0.127	-0.00613	-4.52	6.90E-06	0.000416
GI_24307966-S	SRGAP3	11	-0.126	-0.0193	-4.47	8.72E-06	0.000516
hmm10289-S	---	11	-0.126	-0.0190	-4.46	8.78E-06	0.000519
GI_42661702-S	---	11	-0.125	-0.0229	-4.45	9.50E-06	0.000554
GI_27481523-S	FLJ37453	11	-0.124	-0.00402	-4.40	1.17E-05	0.000671
Hs.170303-S	---	11	-0.123	-0.0205	-4.37	1.37E-05	0.000763
Hs.450384-S	NPM1	11	-0.123	-0.0124	-4.36	1.42E-05	0.000787
GI_37546364-S	METTL1	11	-0.123	-0.00544	-4.35	1.45E-05	0.000803
Hs.5855-S	LOC100288152	11	-0.123	-0.0132	-4.35	1.46E-05	0.000804
hmm22840-S	FLYWCH1	11	-0.122	-0.0199	-4.34	1.54E-05	0.000843
Hs.302274-S	---	11	0.122	0.0111	4.33	1.64E-05	0.000888

GI_10092690-S	TJP3	11	0.122	0.00868	4.32	1.66E-05	0.000897
GI_27734858-S	RNF175	11	-0.122	-0.0209	-4.32	1.68E-05	0.000906
hmm10292-S	---	11	-0.122	-0.0202	-4.32	1.71E-05	0.000916
GI_14043043-A	CCR9	11	-0.122	-0.00607	-4.31	1.73E-05	0.000930
GI_7262383-A	KLRC1	11	-0.121	-0.0185	-4.31	1.78E-05	0.000950
hmm13194-S	MTHFD2L	11	-0.121	-0.0111	-4.28	1.97E-05	0.00104
GI_31542938-S	HPGD	11	-0.120	-0.0189	-4.27	2.15E-05	0.00111
GI_41327765-A	ARMCX2	11	-0.119	-0.00972	-4.22	2.58E-05	0.00129
GI_37542865-S	---	11	-0.119	-0.0169	-4.22	2.59E-05	0.00129
GI_22059218-S	KLHL14	11	-0.119	-0.0214	-4.22	2.66E-05	0.00132
GI_37545966-S	YLPM1	11	-0.120	-0.00196	-4.20	2.89E-05	0.00142
GI_24308244-S	RMI2	11	-0.118	-0.0126	-4.18	3.07E-05	0.00149
GI_32307162-A	AKT3	11	0.117	0.0192	4.16	3.33E-05	0.00159
GI_31652245-I	TNFRSF19	11	-0.117	-0.0167	-4.15	3.49E-05	0.00165
GI_42544176-S	CR2	11	-0.117	-0.0139	-4.14	3.67E-05	0.00172
Hs.443284-S	---	11	-0.117	-0.00675	-4.14	3.69E-05	0.00173
Hs.483404-S	---	11	-0.116	-0.0190	-4.13	3.93E-05	0.00180
GI_5031560-S	GPA33	11	-0.116	-0.0152	-4.12	3.98E-05	0.00183
GI_23065549-S	GSTM2	11	-0.116	-0.00538	-4.10	4.37E-05	0.00198
GI_37059768-S	LRRC34	11	-0.116	-0.0132	-4.10	4.41E-05	0.00200
hmm20898-S	---	11	0.115	0.0189	4.09	4.53E-05	0.00205
GI_31542705-S	PUS7	11	-0.115	-0.0181	-4.09	4.56E-05	0.00206
GI_26051274-S	KCNMB4	11	-0.115	-0.0203	-4.07	4.90E-05	0.00219
GI_21536348-A	ASIC1	11	-0.114	-0.0129	-4.05	5.35E-05	0.00235
GI_41406063-S	MYH10	11	-0.114	-0.0126	-4.03	5.93E-05	0.00257
Hs.27373-S	FAM174B	11	-0.113	-0.0165	-4.01	6.33E-05	0.00273
GI_37655176-S	ZNF415	11	-0.113	-0.0190	-4.00	6.70E-05	0.00285
GI_38570100-A	TBC1D8B	11	0.113	0.0161	3.99	7.10E-05	0.00299
Hs.483054-S	---	11	-0.112	-0.0127	-3.96	7.90E-05	0.00326
GI_20357538-A	ATP6V1G2	11	-0.111	-0.0189	-3.95	8.25E-05	0.00337
GI_29746868-S	---	11	-0.111	-0.0149	-3.94	8.76E-05	0.00354
GI_32967299-A	C1QTNF6	11	-0.111	-0.0186	-3.93	9.12E-05	0.00366
GI_7705754-S	PLLP	11	-0.111	-0.0107	-3.92	9.26E-05	0.00371
GI_42659932-S	---	11	-0.110	-0.00933	-3.90	0.000100	0.00394
GI_37620211-S	---	11	-0.110	-0.0188	-3.90	0.000102	0.00401
Hs.325015-S	---	11	-0.110	-0.0188	-3.88	0.000110	0.00427
GI_44680142-I	SLC23A1	11	-0.109	-0.0132	-3.87	0.000116	0.00444
GI_4505612-S	PAWR	11	-0.109	-0.0163	-3.86	0.000119	0.00455
Hs.462423-S	---	11	-0.108	-0.0112	-3.84	0.000129	0.00486
GI_7657464-S	PODXL2	11	-0.108	-0.0124	-3.84	0.000130	0.00489
GI_21071045-A	SMARCA1	11	-0.107	-0.0160	-3.80	0.000153	0.00559
GI_21396476-A	MEOX1	11	0.107	0.00913	3.79	0.000158	0.00573
GI_38455419-S	RCAN3	11	-0.107	-0.00670	-3.78	0.000166	0.00599
GI_42656307-S	EML6	11	-0.106	-0.0157	-3.76	0.000177	0.00631
Hs.506072-S	LOC100506548	11	-0.106	-0.00467	-3.76	0.000180	0.00641
GI_4502408-S	BICD1	11	-0.106	-0.00469	-3.75	0.000189	0.00665
GI_15451873-I	B3GALNT1	11	-0.105	-0.0158	-3.71	0.000218	0.00749
GI_45243513-A	ABCB9	11	-0.105	-0.00836	-3.71	0.000219	0.00752
GI_42544116-I	ENOSF1	11	-0.104	-0.00738	-3.70	0.000229	0.00780
GI_37546893-S	MTA3	11	-0.104	-0.00832	-3.68	0.000245	0.00828
GI_8923455-A	ANKMY1	11	0.104	0.0157	3.66	0.000258	0.00860
GI_5453622-S	CNKSR1	11	-0.103	-0.0117	-3.66	0.000260	0.00864
GI_21361365-S	IFRD2	11	-0.104	-0.00282	-3.66	0.000268	0.00886
GI_17437334-S	RPLP1	11	-0.103	-0.00346	-3.65	0.000268	0.00887
GI_7661883-S	HELZ	11	-0.104	-0.00202	-3.65	0.000271	0.00893
GI_31317254-S	NLGN2	11	-0.103	-0.0167	-3.65	0.000278	0.00914
hmm23789-S	---	11	-0.103	-0.0142	-3.63	0.000297	0.00967
GI_31542742-S	EFHC2	11	-0.102	-0.00957	-3.63	0.000300	0.00973
GI_14211906-S	ZNF347	11	-0.102	-0.0161	-3.63	0.000300	0.00974
GI_4757845-S	BCL9	11	-0.102	-0.0168	-3.61	0.000313	0.0101
Hs.135647-S	RPP40	11	-0.102	-0.0152	-3.61	0.000314	0.0101
GI_4759059-I	ZFYVE9	11	-0.102	-0.0154	-3.61	0.000320	0.0103
GI_33598967-S	LMO7	11	-0.102	-0.0157	-3.60	0.000327	0.0105
GI_34447228-S	STX16	11	-0.102	-0.00309	-3.58	0.000351	0.0110
hmm10299-S	---	11	-0.101	-0.0161	-3.58	0.000355	0.0111
GI_10938017-S	CCNB2	11	-0.101	-0.0126	-3.58	0.000360	0.0112
GI_18860901-S	PTPRK	11	-0.101	-0.0175	-3.57	0.000376	0.0116
GI_42660906-S	SYCE1L	11	-0.100	-0.00695	-3.55	0.000404	0.0123
GI_31341922-S	MB21D2	11	0.100	0.00865	3.55	0.000406	0.0123
GI_27262656-I	---	11	-0.100	-0.00544	-3.54	0.000415	0.0125
GI_34147389-S	TMEM121	11	-0.100	-0.0118	-3.53	0.000430	0.0129
Hs.445751-S	---	11	-0.0992	-0.0117	-3.51	0.000466	0.0138
Hs.205722-S	---	11	-0.0984	-0.00911	-3.48	0.000520	0.0151
GI_31317245-A	IL24	11	-0.0981	-0.0120	-3.47	0.000537	0.0155
GI_34916035-S	JAKMIP3	11	0.0980	0.0155	3.47	0.000544	0.0157
GI_23308524-S	MORC2-AS1	11	-0.0979	-0.0104	-3.46	0.000553	0.0159
GI_6042201-A	MME	11	-0.0978	-0.0149	-3.46	0.000558	0.0160
GI_24307904-S	SPON1	11	-0.0977	-0.0152	-3.46	0.000565	0.0162
GI_5730010-S	RFPL2	11	0.0974	0.0134	3.45	0.000589	0.0167
GI_21426832-S	RBM11	11	-0.0965	-0.0131	-3.41	0.000661	0.0183
GI_19913405-S	TOP2A	11	-0.0960	-0.0175	-3.40	0.000705	0.0193
Hs.146747-S	---	11	0.0959	0.0110	3.39	0.000715	0.0195
Hs.474412-S	---	11	-0.0958	-0.0138	-3.39	0.000726	0.0197
GI_34147386-S	EFHD1	11	-0.0958	-0.0145	-3.39	0.000729	0.0198
GI_4504012-S	GLDC	11	-0.0957	-0.0175	-3.39	0.000732	0.0198
GI_38455384-S	LAMP3	11	-0.0957	-0.0161	-3.38	0.000734	0.0198
GI_23397571-S	KLHL34	11	-0.0956	-0.0138	-3.38	0.000744	0.0200

GI_20514779-A	SPPL2B	11	-0.0955	-0.0145	-3.38	0.000757	0.0203
Hs.289587-S	USP3-AS1	11	-0.0951	-0.0149	-3.36	0.000791	0.0211
GI_13435144-S	CLUAP1	11	-0.0951	-0.00849	-3.36	0.000799	0.0213
GI_38604072-S	C5orf42	11	-0.0949	-0.0128	-3.36	0.000816	0.0216
GI_5729898-S	KLRAP1	11	0.0948	0.0111	3.35	0.000826	0.0218
GI_28373095-S	ST8SIA1	11	0.0946	0.0136	3.35	0.000841	0.0221
Hs.482647-S	---	11	-0.0943	-0.0159	-3.34	0.000878	0.0228
GI_4501912-S	ADAM23	11	0.0943	0.0114	3.33	0.000881	0.0228
GI_31341815-S	FLJ38379	11	-0.0943	-0.00627	-3.33	0.000887	0.0230
Hs.497310-S	---	11	-0.0941	-0.0110	-3.33	0.000904	0.0233
GI_34303922-S	TRMT10A	11	-0.0939	-0.0136	-3.32	0.000921	0.0237
GI_6677928-S	SH3BGR	11	-0.0939	-0.0143	-3.32	0.000925	0.0237
GI_21361284-S	RCL1	11	-0.0937	-0.00697	-3.31	0.000947	0.0242
Hs.423319-S	---	11	-0.0936	-0.0119	-3.31	0.000957	0.0244
GI_31377724-S	ZNF462	11	-0.0936	-0.0110	-3.31	0.000964	0.0246
GI_33624847-I	SYNE2	11	0.0934	0.0137	3.30	0.000983	0.0249
GI_24308068-S	ZNF521	11	-0.0934	-0.0105	-3.30	0.000989	0.0250
GI_27499706-S	FGF14-AS2	11	-0.0924	-0.0121	-3.27	0.001121	0.0276
Hs.483398-S	---	11	-0.0923	-0.00780	-3.26	0.001135	0.0279
GI_10518496-S	DLL1	11	-0.0920	-0.00799	-3.25	0.001178	0.0287
GI_31377649-S	TMEM25	11	-0.0919	-0.0140	-3.25	0.001183	0.0288
GI_42656192-S	---	11	-0.0918	-0.0172	-3.25	0.001198	0.0291
GI_5730105-S	CXCR6	11	-0.0912	-0.0118	-3.22	0.001300	0.0309
GI_16554580-S	COL5A2	11	-0.0910	-0.0137	-3.22	0.001322	0.0312
GI_9845497-S	LAMC1	11	-0.0909	-0.00979	-3.21	0.001340	0.0316
GI_20336252-I	---	11	-0.0908	-0.00644	-3.21	0.001360	0.0320
GI_15890083-S	COL4A4	11	-0.0905	-0.0129	-3.20	0.001415	0.0330
hmm3484-S	SNHG10	11	-0.0905	-0.00399	-3.19	0.001434	0.0334
GI_10346128-S	DDX43	11	-0.0902	-0.0156	-3.19	0.001468	0.0340
GI_24308377-S	TUBGCP5	11	-0.0900	-0.0142	-3.18	0.001505	0.0347
GI_19923649-S	ZNF607	11	-0.0898	-0.00510	-3.17	0.001543	0.0354
GI_44680155-S	ALG10	11	-0.0896	-0.0140	-3.17	0.001577	0.0360
GI_4503602-S	ESR1	11	-0.0895	-0.0131	-3.17	0.001586	0.0362
GI_42661099-S	TMEM220	11	-0.0894	-0.00879	-3.16	0.001610	0.0366
GI_22538425-A	TRAF3IP2	11	-0.0893	-0.00477	-3.15	0.001652	0.0374
Hs.495177-S	---	11	-0.0890	-0.00502	-3.14	0.001707	0.0383
GI_29540546-A	TRO	11	-0.0889	-0.0150	-3.14	0.001711	0.0384
GI_7657336-S	MLH3	11	-0.0887	-0.0125	-3.13	0.001760	0.0393
GI_14249703-S	RERG	11	-0.0886	-0.00786	-3.13	0.001772	0.0395
GI_42657343-S	---	11	-0.0886	-0.0113	-3.13	0.001773	0.0395
GI_37550176-S	ZNF608	11	-0.0886	-0.0139	-3.13	0.001785	0.0396
GI_18491007-S	CYP2J2	11	-0.0886	-0.0135	-3.13	0.001788	0.0397
GI_45238856-S	---	11	-0.0885	-0.0131	-3.13	0.001793	0.0397
GI_35493986-I	UBE2I	11	-0.0883	-0.00955	-3.12	0.001838	0.0406
GI_29294636-A	MEST	11	-0.0882	-0.0114	-3.12	0.001869	0.0411
hmm28640-S	PLEKHM3	11	-0.0882	-0.0137	-3.12	0.001871	0.0411
Hs.469464-S	---	11	-0.0881	-0.00690	-3.11	0.001889	0.0414
Hs.448493-S	---	11	-0.0881	-0.0140	-3.11	0.001895	0.0415
GI_4505896-S	PLS1	11	-0.0880	-0.0134	-3.11	0.001908	0.0417
GI_37547050-S	ZSWIM5	11	0.0878	0.0124	3.10	0.001969	0.0428
GI_37559987-S	---	11	-0.0877	-0.0140	-3.10	0.001986	0.0431
Hs.427578-S	---	11	-0.0877	-0.0128	-3.10	0.001988	0.0431
GI_4557694-S	KIT	11	-0.0869	-0.0132	-3.07	0.002176	0.0462
GI_38348259-S	KANK3	11	-0.0867	-0.00679	-3.06	0.002232	0.0471
Hs.444093-S	DBT	11	-0.0865	-0.0139	-3.06	0.002293	0.0481
GI_39725694-S	CRIP1	12	0.232	0.00997	8.39	1.33E-16	7.51E-14
GI_4504374-S	CFH	12	0.226	0.0298	8.18	6.78E-16	3.52E-13
GI_18375640-S	BATF	12	0.202	0.00682	7.24	7.87E-13	2.30E-10
GI_38016201-S	SATB2	12	0.199	0.0305	7.15	1.52E-12	4.04E-10
GI_16418432-S	ALPK2	12	0.196	0.0260	7.03	3.39E-12	8.44E-10
Hs.408284-S	ZBTB20-AS1	12	0.193	0.0132	6.93	6.53E-12	1.56E-09
GI_36054181-S	REG4	12	-0.184	-0.0157	-6.59	6.37E-11	1.23E-08
GI_4501882-S	ACTA2	12	0.180	0.00720	6.43	1.80E-10	3.20E-08
GI_37555873-S	---	12	-0.158	-0.00498	-5.63	2.23E-08	2.51E-06
GI_24497541-S	HOXC5	12	0.156	0.0212	5.56	3.30E-08	3.61E-06
GI_13376815-S	CXXC4	12	0.154	0.0167	5.50	4.63E-08	4.91E-06
GI_37595568-S	CLEC11A	12	-0.151	-0.00540	-5.37	9.27E-08	9.25E-06
GI_12621915-S	---	12	0.150	0.0309	5.32	1.20E-07	1.16E-05
GI_4504216-S	GUCY2D	12	-0.149	-0.0111	-5.31	1.28E-07	1.21E-05
GI_31542985-S	ITLN1	12	0.147	0.0118	5.22	2.13E-07	1.95E-05
GI_10092600-S	EIF4G3	12	0.146	0.00299	5.15	3.09E-07	2.71E-05
GI_11545796-A	FAM129A	12	0.144	0.00466	5.12	3.45E-07	2.99E-05
Hs.203743-S	---	12	-0.144	-0.0146	-5.10	3.83E-07	3.27E-05
GI_31341099-S	RNF207	12	0.141	0.00727	4.99	6.77E-07	5.43E-05
Hs.483737-S	---	12	0.140	0.0206	4.96	8.01E-07	6.27E-05
GI_4505948-S	POMC	12	-0.139	-0.00542	-4.93	9.26E-07	7.15E-05
GI_24119161-S	CD70	12	0.138	0.0176	4.91	1.02E-06	7.75E-05
GI_10835156-S	IGFBP2	12	0.138	0.0225	4.91	1.06E-06	7.99E-05
GI_21361999-S	TNIP3	12	0.136	0.00820	4.85	1.42E-06	0.000103
GI_27499434-S	PPFIBP2	12	-0.136	-0.00343	-4.83	1.55E-06	0.000109
GI_19923620-S	HSD3B7	12	0.130	0.0189	4.63	4.01E-06	0.000256
GI_37552339-S	KDM4B	12	-0.130	-0.00242	-4.59	4.79E-06	0.000299
GI_31543399-S	PIAS3	12	0.129	0.00287	4.55	5.91E-06	0.000361
GI_32967310-S	EPHA2	12	-0.128	-0.00632	-4.54	6.05E-06	0.000368
GI_40316916-S	PCSK4	12	-0.126	-0.0189	-4.47	8.45E-06	0.000502
GI_34222372-S	SPNS3	12	-0.125	-0.00506	-4.42	1.08E-05	0.000622
GI_27363487-S	C1QC	12	0.122	0.0203	4.34	1.51E-05	0.000829

hmm30425-S	---	12	-0.122	-0.0176	-4.31	1.76E-05	0.000942
GI_31377717-S	GREM2	12	0.120	0.0182	4.27	2.13E-05	0.00111
GI_35038563-S	KIAA0922	12	-0.121	-0.00249	-4.27	2.15E-05	0.00111
GI_29171761-S	GSC	12	0.120	0.0188	4.26	2.21E-05	0.00114
hmm18872-S	---	12	0.118	0.0107	4.19	3.01E-05	0.00146
GI_24430211-S	IL23R	12	-0.117	-0.0153	-4.16	3.33E-05	0.00159
Hs.473110-S	---	12	0.118	0.00809	4.16	3.33E-05	0.00159
GI_34147619-S	EHD4	12	0.118	0.00234	4.14	3.71E-05	0.00173
GI_38027945-S	COPS6	12	0.118	0.00186	4.13	3.83E-05	0.00178
GI_22208962-S	ASB2	12	0.116	0.00667	4.11	4.25E-05	0.00194
hmm26735-S	SOGA1	12	-0.116	-0.00880	-4.10	4.41E-05	0.00200
Hs.464549-S	---	12	0.115	0.0135	4.08	4.74E-05	0.00213
GI_12545403-S	ICAM5	12	0.115	0.00510	4.07	5.09E-05	0.00225
GI_5729809-S	EBP	12	0.113	0.00469	4.01	6.53E-05	0.00279
GI_30148676-S	FAR2P3	12	0.113	0.0163	4.00	6.77E-05	0.00288
GI_34303933-S	C19orf18	12	-0.113	-0.00793	-3.99	7.13E-05	0.00299
GI_10567815-I	GNAO1	12	0.112	0.0129	3.98	7.27E-05	0.00303
GI_37538413-S	MYO1G	12	0.112	0.00460	3.97	7.57E-05	0.00314
Hs.520721-S	---	12	0.112	0.0167	3.96	8.04E-05	0.00330
GI_4505042-S	LTF	12	0.111	0.0268	3.94	8.63E-05	0.00350
GI_19224662-S	TCEAL2	12	0.111	0.0131	3.93	8.98E-05	0.00362
GI_31341724-S	C1orf110	12	0.111	0.0166	3.93	9.04E-05	0.00364
GI_5016087-S	ACTA1	12	0.110	0.0133	3.90	0.000101	0.00398
GI_37546892-S	PKDCC	12	-0.109	-0.0102	-3.88	0.000111	0.00429
GI_45007001-S	CERS6	12	-0.109	-0.00406	-3.87	0.000114	0.00439
GI_44917611-S	KIF1C	12	0.109	0.00419	3.87	0.000117	0.00446
GI_42822890-S	MIEN1	12	0.110	0.00224	3.86	0.000120	0.00456
GI_4503752-I	FLT4	12	-0.109	-0.00287	-3.83	0.000135	0.00505
GI_24308353-S	C16orf45	12	0.108	0.00391	3.82	0.000140	0.00518
GI_4503954-S	BLOC1S1	12	0.108	0.00296	3.81	0.000144	0.00533
Hs.493281-S	---	12	-0.108	-0.00906	-3.81	0.000145	0.00538
hmm24546-S	PRSS57	12	0.107	0.0179	3.80	0.000153	0.00558
Hs.501419-S	---	12	-0.107	-0.0118	-3.79	0.000155	0.00566
GI_24497529-I	---	12	0.107	0.00440	3.78	0.000162	0.00584
GI_21704278-A	JAG2	12	0.106	0.0173	3.75	0.000184	0.00653
Hs.511682-S	---	12	0.106	0.0114	3.75	0.000185	0.00655
GI_40288196-S	GATA6	12	0.106	0.0151	3.74	0.000196	0.00687
GI_37540292-S	FAM221B	12	-0.105	-0.0114	-3.73	0.000197	0.00689
GI_4507686-S	TRPC3	12	0.105	0.00654	3.73	0.000199	0.00694
hmm21427-S	---	12	0.105	0.0140	3.70	0.000222	0.00760
GI_19743853-A	DCN	12	-0.104	-0.0133	-3.70	0.000229	0.00780
GI_8922406-S	FIGN	12	-0.104	-0.0148	-3.67	0.000251	0.00845
GI_4504542-S	HTR3A	12	-0.104	-0.0133	-3.67	0.000252	0.00847
hmm20063-S	---	12	0.104	0.0101	3.67	0.000254	0.00852
GI_31543182-S	---	12	-0.103	-0.0154	-3.64	0.000287	0.00937
GI_37537711-S	EPB41L4A	12	0.102	0.0128	3.61	0.000317	0.0102
GI_38093656-S	GPRIN3	12	0.102	0.00346	3.61	0.000322	0.0103
GI_29746633-S	SLC35E4	12	0.102	0.00633	3.60	0.000331	0.0106
hmm19284-S	---	12	-0.101	-0.0144	-3.59	0.000348	0.0110
Hs.463395-S	---	12	0.101	0.0137	3.58	0.000351	0.0110
Hs.368200-S	---	12	0.101	0.0133	3.57	0.000368	0.0114
GI_7662177-S	---	12	0.101	0.00595	3.57	0.000369	0.0114
Hs.276341-S	---	12	-0.101	-0.00838	-3.57	0.000375	0.0116
GI_38176293-S	ANKDD1A	12	0.101	0.00621	3.57	0.000376	0.0116
GI_27498471-S	ZSCAN23	12	0.101	0.00565	3.56	0.000388	0.0119
hmm34835-S	---	12	-0.100	-0.0126	-3.53	0.000426	0.0128
hmm4390-S	---	12	0.100	0.0146	3.52	0.000439	0.0131
GI_7662101-S	LRRTM2	12	-0.0994	-0.0150	-3.52	0.000451	0.0134
GI_27436900-S	MRPL12	12	-0.0995	-0.00209	-3.50	0.000483	0.0142
GI_27482674-S	PROSER2-AS1	12	0.0989	0.00774	3.50	0.000485	0.0142
Hs.519917-S	---	12	0.0990	0.00440	3.50	0.000487	0.0142
Hs.503601-S	---	12	0.0987	0.0174	3.49	0.000496	0.0145
GI_42476154-S	PTMS	12	0.0989	0.00369	3.49	0.000498	0.0145
hmm24798-S	---	12	0.0984	0.0150	3.48	0.000513	0.0149
GI_20535873-S	---	12	0.0984	0.0140	3.48	0.000519	0.0151
GI_42658224-S	VWDE	12	0.0978	0.0135	3.46	0.000559	0.0160
Hs.58509-S	LOC100506388	12	-0.0977	-0.0148	-3.46	0.000565	0.0162
GI_20536598-S	LOC100506405	12	-0.0975	-0.0147	-3.45	0.000584	0.0166
GI_31982944-S	SLC5A10	12	0.0973	0.0111	3.44	0.000597	0.0169
Hs.469893-S	---	12	0.0972	0.0129	3.44	0.000602	0.0169
GI_37563645-S	MIAT	12	0.0972	0.0106	3.44	0.000602	0.0169
GI_21327676-S	HLA-B	12	0.0975	0.00320	3.44	0.000602	0.0169
GI_21166383-A	HEPH	12	0.0972	0.00599	3.44	0.000612	0.0172
hmm19899-S	---	12	0.0979	0.00168	3.43	0.000616	0.0173
GI_7669552-S	VCP	12	0.0979	0.00143	3.42	0.000641	0.0179
GI_42476034-S	FAM20A	12	0.0966	0.0149	3.42	0.000657	0.0182
GI_42659240-S	SNX30	12	0.0965	0.0157	3.41	0.000662	0.0183
GI_42476062-S	PXDC1	12	0.0964	0.00877	3.41	0.000669	0.0185
Hs.374128-S	---	12	0.0964	0.00947	3.41	0.000674	0.0185
Hs.484809-S	---	12	-0.0963	-0.0118	-3.41	0.000680	0.0187
GI_21361986-A	TMTC1	12	0.0963	0.0146	3.41	0.000680	0.0187
GI_4505840-S	PKP1	12	0.0962	0.0158	3.40	0.000687	0.0188
GI_22749242-S	C1orf177	12	0.0962	0.0103	3.40	0.000689	0.0189
Hs.492837-S	---	12	0.0958	0.0113	3.39	0.000721	0.0196
GI_9845523-S	EGR2	12	0.0958	0.00953	3.39	0.000729	0.0198
GI_11119429-S	TSKS	12	-0.0957	-0.0144	-3.39	0.000730	0.0198
Hs.454935-S	---	12	0.0958	0.00498	3.39	0.000731	0.0198

Hs.444774-S	---	12	0.0956	0.00700	3.38	0.000743	0.0200
GI_37574713-S	CAMSAP3	12	-0.0950	-0.00853	-3.36	0.000801	0.0213
hmm34749-S	---	12	0.0950	0.00771	3.36	0.000805	0.0213
GI_21536459-S	MAP3K5	12	0.0949	0.00283	3.35	0.000838	0.0220
GI_42656660-S	---	12	-0.0948	-0.00452	-3.35	0.000839	0.0220
GI_38679925-S	CERCAM	12	0.0946	0.00979	3.35	0.000847	0.0222
GI_27894286-A	PHF7	12	0.0946	0.00487	3.34	0.000849	0.0222
GI_15451871-S	B3GALT2	12	-0.0944	-0.0130	-3.34	0.000865	0.0225
Hs.507256-S	---	12	0.0944	0.0147	3.34	0.000869	0.0226
GI_29788761-S	DNTT	12	-0.0942	-0.0125	-3.33	0.000887	0.0230
Hs.378091-S	---	12	0.0942	0.0151	3.33	0.000893	0.0231
GI_37552466-S	---	12	0.0942	0.0156	3.33	0.000894	0.0231
GI_12621916-S	---	12	0.0939	0.0169	3.32	0.000921	0.0237
GI_36054149-S	RIMS4	12	0.0939	0.0123	3.32	0.000924	0.0237
GI_23199981-S	RNF112	12	0.0938	0.00393	3.31	0.000955	0.0244
GI_19923712-A	BNIPL	12	-0.0934	-0.00477	-3.30	0.000991	0.0250
GI_40789088-S	NTAN1	12	0.0935	0.00306	3.30	0.001002	0.0253
Hs.498522-S	---	12	-0.0932	-0.0122	-3.30	0.001004	0.0253
Hs.482834-S	---	12	0.0932	0.0139	3.29	0.001013	0.0255
GI_37539105-S	MGC72080	12	-0.0937	-0.00196	-3.29	0.001015	0.0255
GI_18490994-A	MRVI1	12	0.0930	0.0117	3.29	0.001040	0.0260
Hs.463570-S	---	12	0.0930	0.0104	3.29	0.001042	0.0261
Hs.83346-S	---	12	-0.0929	-0.00971	-3.28	0.001050	0.0262
GI_5031882-S	LOXL1	12	0.0928	0.0124	3.28	0.001061	0.0264
GI_10518505-A	F8	12	0.0922	0.00691	3.26	0.001150	0.0282
GI_34916041-S	ESPNL	12	-0.0921	-0.0126	-3.26	0.001156	0.0283
GI_29568104-S	---	12	-0.0921	-0.00585	-3.25	0.001172	0.0286
GI_13569896-S	SHCBP1L	12	0.0919	0.0140	3.25	0.001192	0.0289
GI_27734902-S	ZNF584	12	-0.0917	-0.0143	-3.24	0.001220	0.0294
Hs.475556-S	---	12	-0.0917	-0.00715	-3.24	0.001224	0.0295
GI_28916690-A	MUC1	12	0.0916	0.0132	3.24	0.001228	0.0296
GI_23238209-A	ARPC2	12	0.0921	0.00177	3.23	0.001251	0.0300
Hs.445547-S	---	12	0.0915	0.00859	3.23	0.001252	0.0300
GI_39725713-S	ALG3	12	-0.0916	-0.00290	-3.23	0.001262	0.0302
hmm22123-S	---	12	-0.0914	-0.0111	-3.23	0.001270	0.0303
Hs.385594-S	---	12	-0.0913	-0.0142	-3.23	0.001273	0.0304
Hs.220971-S	---	12	0.0912	0.00613	3.22	0.001300	0.0309
hmm33438-S	---	12	-0.0911	-0.00901	-3.22	0.001309	0.0310
hmm30955-S	---	12	0.0908	0.00416	3.21	0.001373	0.0322
hmm36387-S	---	12	-0.0907	-0.00903	-3.21	0.001377	0.0323
GI_16306540-S	CDH9	12	0.0907	0.00719	3.21	0.001377	0.0323
Hs.490862-S	---	12	0.0905	0.00885	3.20	0.001418	0.0331
GI_15011976-S	ARHGEF16	12	0.0904	0.0124	3.20	0.001433	0.0333
GI_41208742-S	BLOC1S3	12	-0.0904	-0.0144	-3.19	0.001437	0.0334
Hs.514920-S	---	12	0.0903	0.00475	3.19	0.001457	0.0338
hmm20684-S	SLC22A20	12	0.0900	0.0108	3.18	0.001503	0.0347
GI_13376565-S	C3orf36	12	0.0900	0.0118	3.18	0.001506	0.0347
GI_42662756-S	---	12	0.0898	0.0136	3.17	0.001545	0.0354
Hs.459502-S	---	12	0.0897	0.0114	3.17	0.001553	0.0355
GI_23957699-S	ZNF114	12	-0.0896	-0.00842	-3.17	0.001576	0.0360
GI_4557632-S	GRID2	12	-0.0894	-0.0139	-3.16	0.001608	0.0366
GI_39777598-I	TGM2	12	0.0893	0.0106	3.16	0.001630	0.0370
Hs.152466-S	---	12	0.0893	0.00647	3.15	0.001649	0.0374
GI_37548655-S	OLFML2B	12	0.0893	0.00558	3.15	0.001652	0.0374
GI_23503296-S	PRSS35	12	-0.0892	-0.0104	-3.15	0.001656	0.0375
GI_42657574-S	C6orf52	12	-0.0893	-0.00371	-3.15	0.001663	0.0375
GI_27478827-S	---	12	0.0892	0.0133	3.15	0.001662	0.0375
GI_34147486-A	POFUT2	12	0.0895	0.00211	3.15	0.001680	0.0379
GI_41222692-S	---	12	0.0888	0.0140	3.14	0.001741	0.0390
Hs.525142-S	---	12	0.0888	0.0135	3.14	0.001743	0.0390
GI_9257200-S	CALY	12	0.0888	0.00790	3.14	0.001742	0.0390
GI_37546156-S	GLOD5	12	0.0888	0.0136	3.14	0.001745	0.0390
GI_34222229-S	FAM163A	12	-0.0887	-0.0129	-3.13	0.001761	0.0393
GI_27484966-S	---	12	0.0887	0.0136	3.13	0.001765	0.0393
GI_21314673-S	ERRFI1	12	0.0887	0.00359	3.13	0.001784	0.0396
GI_29550920-I	---	12	0.0886	0.00415	3.13	0.001792	0.0397
GI_29734567-S	---	12	0.0883	0.0121	3.12	0.001841	0.0406
GI_13376049-S	BMP8A	12	-0.0881	-0.0107	-3.11	0.001884	0.0414
GI_34222307-S	TPBG	12	0.0881	0.0135	3.11	0.001899	0.0416
Hs.127491-S	---	12	0.0879	0.0102	3.11	0.001935	0.0422
Hs.473220-S	---	12	0.0879	0.0100	3.11	0.001935	0.0422
GI_38327641-S	WDR55	12	0.0885	0.00167	3.11	0.001934	0.0422
GI_13129141-S	DHX58	12	-0.0882	-0.00194	-3.10	0.001962	0.0427
GI_21450823-S	DACT3	12	-0.0878	-0.00810	-3.10	0.001964	0.0427
GI_42734318-S	DCLRE1A	12	0.0878	0.00308	3.10	0.001993	0.0432
Hs.310697-S	---	12	0.0877	0.00679	3.10	0.001995	0.0432
GI_29743461-S	---	12	0.0875	0.00866	3.09	0.002031	0.0439
hmm19462-S	---	12	0.0874	0.00896	3.09	0.002057	0.0443
GI_5453709-S	LASP1	12	0.0877	0.00231	3.09	0.002068	0.0445
GI_40255082-S	SPTSSB	12	-0.0874	-0.00420	-3.09	0.002073	0.0446
Hs.81889-S	---	12	0.0873	0.0121	3.08	0.002088	0.0448
GI_40254477-S	SLC1A2	12	0.0873	0.00581	3.08	0.002095	0.0449
GI_29747632-S	---	12	0.0872	0.00865	3.08	0.002114	0.0452
hmm23603-S	---	12	-0.0871	-0.0110	-3.08	0.002122	0.0453
GI_17402913-A	WNT16	12	0.0872	0.00415	3.08	0.002126	0.0454
GI_27477711-S	LOC100505685	12	0.0871	0.00433	3.08	0.002145	0.0457
Hs.270801-S	---	12	0.0869	0.00899	3.07	0.002179	0.0463

hmm21666-S	---	12	-0.0869	-0.00823	-3.07	0.002180	0.0463
GI_15208653-S	DGCR6	12	0.0871	0.00273	3.07	0.002185	0.0463
Hs.500574-S	---	12	-0.0868	-0.00839	-3.07	0.002202	0.0466
Hs.396516-S	---	12	-0.0867	-0.0111	-3.07	0.002221	0.0469
hmm25481-S	---	12	-0.0868	-0.00482	-3.07	0.002223	0.0470
GI_18379345-S	SORCS3	12	0.0867	0.0126	3.06	0.002232	0.0471
GI_20482378-S	OR5B21	12	-0.0867	-0.0124	-3.06	0.002242	0.0473
Hs.373509-S	---	12	0.0866	0.0130	3.06	0.002253	0.0475
GI_18201908-A	VCAM1	12	0.0864	0.0139	3.05	0.002311	0.0484
Hs.490304-S	---	12	0.0863	0.0129	3.05	0.002334	0.0487
GI_31543061-S	TMEM45B	12	0.0863	0.00968	3.05	0.002354	0.0490
GI_11496969-S	ADH7	12	-0.0863	-0.00584	-3.05	0.002363	0.0492
Hs.127566-S	---	12	0.0862	0.0119	3.05	0.002369	0.0493
GI_20143915-I	TTN	12	-0.0862	-0.0126	-3.04	0.002379	0.0494
GI_9910361-S	SEMA3G	12	0.0862	0.0120	3.04	0.002378	0.0494
GI_19923110-S	IGFBP3	13	0.399	0.0370	15.3	9.89E-49	2.34E-44
GI_31377588-S	SYT11	13	0.361	0.0112	13.6	2.73E-39	2.58E-35
GI_5032234-S	RCAN2	13	0.351	0.0623	13.2	2.77E-37	1.87E-33
GI_21389504-S	JAKMIP1	13	0.311	0.0228	11.5	3.90E-29	1.42E-25
GI_5360215-I	IDS	13	0.298	0.00652	10.9	1.11E-26	2.77E-23
Hs.355618-S	MIAT	13	0.294	0.0143	10.8	3.81E-26	8.58E-23
GI_41149833-S	LINC00944	13	0.286	0.0141	10.5	9.21E-25	1.61E-21
GI_38348363-S	MXRA7	13	0.274	0.0148	10.0	9.26E-23	1.25E-19
GI_15718681-S	LAG3	13	0.271	0.0174	9.90	2.81E-22	3.41E-19
GI_31543092-S	GLB1L2	13	0.266	0.0150	9.72	1.41E-21	1.56E-18
GI_6996017-S	MSC	13	0.264	0.0205	9.63	3.25E-21	3.33E-18
GI_38569483-S	KIF21A	13	0.257	0.0104	9.34	4.14E-20	3.69E-17
GI_33356156-S	APOBEC3H	13	0.249	0.0409	9.04	6.13E-19	4.99E-16
GI_34147604-S	OPTN	13	0.245	0.00638	8.86	2.81E-18	2.07E-15
GI_22749032-S	MANEAL	13	0.244	0.0380	8.85	2.86E-18	2.08E-15
GI_4885266-S	GFI1	13	0.243	0.0124	8.80	4.34E-18	3.06E-15
GI_21450644-S	TTC16	13	0.242	0.0206	8.79	4.92E-18	3.42E-15
GI_7662169-S	NUAK1	13	0.233	0.0333	8.45	8.21E-17	4.91E-14
hmm2656-S	LINC00944	13	0.228	0.0405	8.24	4.18E-16	2.20E-13
GI_14670372-A	PCBP4	13	0.223	0.0116	8.06	1.83E-15	8.99E-13
GI_16905508-A	B3GAT1	13	0.222	0.0285	8.03	2.29E-15	1.10E-12
Hs.11594-S	---	13	0.221	0.0117	7.97	3.63E-15	1.67E-12
GI_5453735-S	MAF	13	0.220	0.00839	7.92	5.13E-15	2.31E-12
hmm30181-S	TNIP3	13	0.219	0.0326	7.88	6.89E-15	3.02E-12
GI_24308074-S	RAB11FIP5	13	0.218	0.0152	7.88	7.32E-15	3.18E-12
GI_27484243-S	PATL2	13	0.216	0.0314	7.77	1.60E-14	6.29E-12
GI_27597087-S	CYB561	13	0.215	0.0105	7.74	1.99E-14	7.66E-12
GI_4826845-S	MYO6	13	0.215	0.0389	7.74	2.10E-14	8.00E-12
GI_5174402-S	CACNA2D2	13	0.209	0.0127	7.52	1.06E-13	3.75E-11
GI_37550187-S	C5orf56	13	0.205	0.00473	7.33	4.25E-13	1.31E-10
GI_37550554-S	ZBTB38	13	0.198	0.0104	7.09	2.26E-12	5.87E-10
GI_22538813-S	CCL5	13	0.197	0.0111	7.08	2.49E-12	6.39E-10
GI_42659320-S	---	13	0.196	0.00723	7.04	3.19E-12	8.06E-10
Hs.293917-S	---	13	0.192	0.0171	6.90	8.03E-12	1.88E-09
GI_21070979-A	PAM	13	0.192	0.00454	6.85	1.17E-11	2.64E-09
GI_24308397-S	TMEM116	13	0.191	0.00629	6.84	1.22E-11	2.73E-09
GI_13569955-S	ARPC5L	13	0.191	0.00517	6.82	1.44E-11	3.19E-09
GI_42657283-S	C5orf56	13	0.189	0.00601	6.77	1.96E-11	4.30E-09
GI_4506520-S	RGS9	13	0.182	0.0227	6.52	1.01E-10	1.86E-08
GI_21361441-S	C18orf8	13	0.183	0.00256	6.45	1.61E-10	2.91E-08
GI_21361572-S	CHN1	13	0.179	0.0252	6.41	2.02E-10	3.53E-08
GI_5453624-S	C1orf61	13	0.179	0.0161	6.41	2.09E-10	3.64E-08
GI_22325374-A	EDARADD	13	0.178	0.0187	6.37	2.63E-10	4.45E-08
GI_24307908-S	RALGDS	13	0.178	0.00938	6.36	2.90E-10	4.87E-08
Hs.513794-S	---	13	0.177	0.0310	6.35	3.05E-10	5.10E-08
hmm19468-S	---	13	0.177	0.0107	6.34	3.28E-10	5.41E-08
Hs.243596-S	KATNAL1	13	0.175	0.00765	6.25	5.52E-10	8.71E-08
GI_33946273-A	PRDM1	13	0.175	0.00824	6.25	5.59E-10	8.75E-08
GI_4502686-S	CD84	13	0.174	0.00670	6.21	7.34E-10	1.12E-07
GI_4501848-S	ABCA3	13	0.173	0.0113	6.18	8.75E-10	1.31E-07
GI_37549797-S	FCRL6	13	0.172	0.0163	6.15	1.03E-09	1.53E-07
GI_31377630-S	GBP5	13	0.172	0.00670	6.15	1.05E-09	1.56E-07
GI_18860903-S	PTPRM	13	0.170	0.0324	6.08	1.61E-09	2.33E-07
GI_4506374-S	RAB7L1	13	0.169	0.00498	6.00	2.55E-09	3.56E-07
GI_18579257-S	---	13	0.165	0.0249	5.89	4.90E-09	6.41E-07
GI_41872576-S	RFTN1	13	0.166	0.00300	5.89	5.00E-09	6.52E-07
GI_13376859-S	SLC35G2	13	0.165	0.0250	5.89	5.10E-09	6.60E-07
GI_4503928-S	GATA3	13	0.165	0.00707	5.88	5.14E-09	6.62E-07
GI_22749528-S	C17orf66	13	0.165	0.0162	5.88	5.26E-09	6.77E-07
GI_21359902-S	HDGFRP3	13	0.163	0.0103	5.81	7.90E-09	9.86E-07
GI_37059743-S	PIP4K2C	13	0.164	0.00318	5.81	8.11E-09	1.00E-06
GI_34303927-S	ATP1A3	13	0.162	0.00962	5.79	8.92E-09	1.10E-06
GI_7706110-S	CD320	13	0.160	0.00605	5.71	1.42E-08	1.68E-06
GI_41406088-S	SOX13	13	0.160	0.0134	5.70	1.45E-08	1.72E-06
Hs.517064-S	---	13	0.160	0.00845	5.69	1.55E-08	1.82E-06
GI_13399303-S	APOBEC3G	13	0.160	0.00563	5.69	1.56E-08	1.82E-06
hmm15467-S	---	13	0.159	0.00849	5.66	1.84E-08	2.11E-06
GI_25121959-S	PTCH1	13	0.157	0.00826	5.60	2.64E-08	2.95E-06
GI_6466447-S	DSTN	13	0.158	0.00366	5.59	2.73E-08	3.03E-06
GI_22749050-S	---	13	0.157	0.00875	5.58	3.02E-08	3.32E-06
GI_28559030-A	IL5RA	13	0.150	0.0214	5.36	1.00E-07	9.90E-06
GI_42661316-S	---	13	0.150	0.00868	5.33	1.18E-07	1.14E-05

GI_28376647-S	---	13	0.150	0.0120	5.32	1.21E-07	1.16E-05
GI_37595540-A	TRIM69	13	0.149	0.00717	5.31	1.32E-07	1.26E-05
GI_22748764-S	CLDND2	13	0.148	0.0254	5.28	1.54E-07	1.44E-05
GI_37540072-S	MCOLN2	13	0.148	0.0139	5.27	1.59E-07	1.49E-05
GI_16418372-S	---	13	0.147	0.0143	5.22	2.10E-07	1.93E-05
GI_34335276-S	PPP1R16B	13	0.146	0.00380	5.18	2.60E-07	2.31E-05
GI_31341110-S	TIGIT	13	0.144	0.0211	5.12	3.49E-07	3.02E-05
GI_7662301-S	TBKBP1	13	0.143	0.0129	5.10	3.99E-07	3.38E-05
GI_21237760-S	CD81	13	0.144	0.00317	5.09	4.13E-07	3.48E-05
GI_31324542-S	CAMK2N1	13	0.142	0.0158	5.06	4.89E-07	4.03E-05
Hs.442319-S	---	13	0.142	0.0229	5.05	5.10E-07	4.19E-05
GI_21389384-S	RHEBL1	13	0.141	0.00670	5.02	5.94E-07	4.84E-05
GI_22749520-S	GK5	13	0.139	0.00814	4.94	8.75E-07	6.79E-05
GI_19923492-S	PLEKHA5	13	0.139	0.0107	4.93	9.28E-07	7.15E-05
GI_37543871-S	DNAH10	13	0.138	0.0227	4.90	1.11E-06	8.32E-05
GI_41146823-S	---	13	0.136	0.0197	4.84	1.49E-06	0.000106
GI_4557248-S	ADA	13	0.137	0.00271	4.84	1.49E-06	0.000106
GI_24497439-A	IL12RB1	13	0.135	0.0117	4.81	1.71E-06	0.000119
GI_37550189-S	SEPT8	13	0.134	0.0212	4.77	2.07E-06	0.000142
GI_27478592-S	---	13	0.133	0.00507	4.71	2.75E-06	0.000183
GI_33946273-I	PRDM1	13	0.132	0.00693	4.70	2.89E-06	0.000192
GI_40254903-S	CASZ1	13	0.132	0.00735	4.68	3.21E-06	0.000210
GI_8922178-S	STYK1	13	0.131	0.0179	4.65	3.62E-06	0.000235
GI_42716310-S	ARNTL	13	0.131	0.00288	4.63	4.02E-06	0.000256
GI_31377810-S	LSS	13	0.131	0.00558	4.63	4.06E-06	0.000259
GI_11386198-S	PVRL3	13	0.130	0.0224	4.61	4.50E-06	0.000283
GI_13518027-S	BFSP1	13	0.129	0.0141	4.58	5.16E-06	0.000319
GI_13540544-S	ZBP1	13	0.129	0.00466	4.57	5.27E-06	0.000325
Hs.374140-S	---	13	0.127	0.0153	4.51	7.06E-06	0.000424
GI_42544245-S	SEPW1	13	0.127	0.00251	4.46	8.84E-06	0.000521
GI_29731922-S	CEP78	13	0.125	0.00686	4.44	9.87E-06	0.000575
GI_22095346-S	CADM1	13	0.124	0.0164	4.40	1.17E-05	0.000671
GI_23957707-S	LPCAT4	13	0.123	0.00349	4.36	1.40E-05	0.000775
GI_7662357-S	ENPP4	13	0.123	0.00616	4.35	1.49E-05	0.000822
GI_33187364-S	EIF5A2	13	0.121	0.00511	4.28	1.98E-05	0.00104
GI_34222262-S	ITPRIPL1	13	0.120	0.00751	4.26	2.24E-05	0.00114
GI_38455386-S	ENPP5	13	0.119	0.0194	4.21	2.71E-05	0.00134
GI_8051633-S	RARRES3	13	0.119	0.00384	4.20	2.84E-05	0.00139
GI_22779931-A	SYNE1	13	0.118	0.00356	4.19	3.05E-05	0.00148
GI_37551767-S	TMEM181	13	0.118	0.00471	4.16	3.36E-05	0.00160
GI_34147575-S	IL32	13	0.117	0.00588	4.13	3.87E-05	0.00179
Hs.515625-S	---	13	0.116	0.0126	4.13	3.92E-05	0.00180
GI_19743812-I	ITGB1	13	0.115	0.00415	4.08	4.76E-05	0.00213
GI_8922070-S	ZNF821	13	0.115	0.00580	4.08	4.89E-05	0.00218
GI_12408645-S	CAPN2	13	0.115	0.00337	4.07	4.99E-05	0.00222
GI_18104977-S	PTPN1	13	0.115	0.00258	4.07	4.99E-05	0.00222
GI_13376227-S	HDAC11	13	0.115	0.0129	4.07	5.07E-05	0.00225
GI_25121986-S	NCAPH	13	0.113	0.0184	4.01	6.31E-05	0.00272
GI_22749146-S	SH3RF2	13	0.112	0.0174	3.96	7.96E-05	0.00328
GI_42661849-S	PRSS57	13	0.112	0.0164	3.95	8.11E-05	0.00332
GI_18105053-S	KIFAP3	13	0.112	0.00343	3.95	8.27E-05	0.00337
GI_21361404-S	RGS17	13	0.111	0.0186	3.94	8.62E-05	0.00350
GI_24797159-S	TAP1	13	0.111	0.00230	3.92	9.17E-05	0.00368
GI_44917605-S	NAPB	13	0.111	0.00323	3.91	9.75E-05	0.00386
GI_4504884-I	KLRC3	13	0.108	0.00780	3.84	0.000129	0.00487
GI_22748722-S	ACOT4	13	0.108	0.0168	3.81	0.000145	0.00537
GI_29736110-S	LINC00239	13	0.107	0.0145	3.80	0.000153	0.00558
GI_34335234-A	CORO2A	13	0.107	0.0104	3.80	0.000153	0.00558
GI_10835046-S	SGCE	13	0.107	0.0152	3.79	0.000157	0.00570
GI_13375645-S	LRFN3	13	0.107	0.0130	3.78	0.000164	0.00590
GI_4758913-S	LITAF	13	0.106	0.00486	3.76	0.000178	0.00633
GI_20270354-S	GALM	13	0.106	0.00607	3.75	0.000182	0.00647
GI_21464103-S	YWHAQ	13	0.106	0.00314	3.75	0.000185	0.00655
GI_32189374-S	SGCB	13	0.105	0.0127	3.72	0.000205	0.00711
GI_24371267-S	NAP1L5	13	0.105	0.00544	3.70	0.000222	0.00760
GI_31542523-S	VOPP1	13	0.105	0.00287	3.69	0.000231	0.00787
GI_21361293-S	KLRG1	13	0.104	0.00565	3.68	0.000242	0.00819
GI_4885170-S	MAMLD1	13	0.104	0.0125	3.68	0.000246	0.00830
GI_42661179-S	TBKBP1	13	0.104	0.00675	3.67	0.000254	0.00852
GI_22547143-A	FBXO32	13	0.102	0.00373	3.60	0.000332	0.0106
GI_31542788-S	EPHX4	13	0.102	0.00647	3.60	0.000335	0.0106
Hs.23853-S	ZBTB38	13	0.100	0.0138	3.54	0.000412	0.0125
GI_40538791-S	NIP7	13	0.0990	0.00441	3.50	0.000486	0.0142
GI_31377534-S	---	13	0.0989	0.00176	3.47	0.000536	0.0155
GI_41281572-S	BAHD1	13	0.0963	0.00235	3.39	0.000720	0.0196
GI_22054273-S	TMEM244	13	0.0947	0.0116	3.35	0.000831	0.0219
Hs.8882-S	GPR68	13	0.0938	0.00721	3.32	0.000934	0.0239
GI_42656068-S	---	13	0.0938	0.00416	3.31	0.000947	0.0242
GI_38327616-S	SLAMF6	13	0.0928	0.00365	3.28	0.001081	0.0268
GI_24586683-A	SYNM	13	0.0921	0.0123	3.26	0.001159	0.0283
GI_37059782-S	NINL	13	0.0920	0.00761	3.25	0.001175	0.0286
GI_29171703-A	MAGED2	13	0.0911	0.00143	3.19	0.001477	0.0342
GI_40805101-S	ZNF276	13	0.0897	0.00258	3.16	0.001606	0.0366
GI_34147355-S	NABP2	13	0.0894	0.00401	3.16	0.001631	0.0370
GI_10863920-S	DLG3	13	0.0888	0.0127	3.14	0.001726	0.0387
GI_4557328-S	FASLG	13	0.0885	0.0111	3.13	0.001802	0.0399
GI_33624872-A	SYNE2	13	0.0881	0.0139	3.11	0.001887	0.0414

GI_31563332-A	AKAP13	13	0.0890	0.00133	3.11	0.001910	0.0417
GI_25952146-A	TNFSF14	13	0.0879	0.00452	3.11	0.001943	0.0423
hmm31250-S	GRPEL2	13	0.0871	0.00396	3.07	0.002151	0.0458
GI_42658956-S	TOX	13	0.0860	0.0125	3.04	0.002409	0.0499
GI_13929470-S	B4GALT5	14	0.287	0.00645	10.5	8.67E-25	1.58E-21
GI_31377551-S	RAP2A	14	0.227	0.00769	8.18	7.17E-16	3.69E-13
GI_4502280-S	ATP1B3	14	0.189	0.00427	6.74	2.44E-11	5.24E-09
GI_23510430-A	FAS	14	0.187	0.00806	6.68	3.52E-11	7.34E-09
GI_42660339-S	---	14	0.181	0.00357	6.43	1.82E-10	3.23E-08
GI_22129777-S	SRXN1	14	0.176	0.00543	6.29	4.26E-10	6.87E-08
GI_19743892-I	TADA3	14	0.177	0.00283	6.25	5.58E-10	8.75E-08
GI_31881629-S	PTGER2	14	0.175	0.00665	6.25	5.67E-10	8.83E-08
GI_9845514-I	S100A4	14	0.170	0.00570	6.08	1.63E-09	2.34E-07
GI_6006015-S	LGALS1	14	0.168	0.00573	5.99	2.76E-09	3.76E-07
GI_6006040-S	SLC25A20	14	0.166	0.00507	5.90	4.70E-09	6.17E-07
GI_7705703-S	GSTK1	14	0.157	0.00260	5.53	3.97E-08	4.25E-06
GI_5729985-S	POP4	14	0.156	0.00220	5.47	5.44E-08	5.71E-06
GI_31742486-S	KIF3B	14	0.152	0.00227	5.35	1.06E-07	1.04E-05
GI_20986530-A	MAPK1	14	0.149	0.00328	5.28	1.52E-07	1.43E-05
GI_32455253-A	CERS2	14	0.148	0.00229	5.21	2.23E-07	2.02E-05
GI_21264579-S	TSPAN2	14	0.146	0.0229	5.20	2.35E-07	2.13E-05
GI_20986528-I	MAPK1	14	0.147	0.00244	5.19	2.48E-07	2.22E-05
GI_30023852-S	MTSS1	14	0.146	0.00527	5.17	2.68E-07	2.38E-05
GI_41406049-S	DOK2	14	0.145	0.00512	5.16	2.81E-07	2.48E-05
GI_19743895-A	TADA3	14	0.146	0.00328	5.16	2.89E-07	2.55E-05
GI_41222100-S	DPY19L1	14	0.145	0.00475	5.13	3.31E-07	2.88E-05
GI_19743818-A	ITGB1	14	0.143	0.00679	5.08	4.37E-07	3.65E-05
GI_4758013-S	CMKLR1	14	0.142	0.00876	5.03	5.51E-07	4.50E-05
GI_10835138-S	FCGR3B	14	0.140	0.00877	4.96	8.12E-07	6.33E-05
GI_5901975-S	PLA2G16	14	0.139	0.00507	4.93	9.41E-07	7.21E-05
GI_14589848-S	GNGT2	14	0.138	0.0130	4.91	1.05E-06	7.98E-05
GI_24475653-S	PROK2	14	0.137	0.00917	4.86	1.31E-06	9.62E-05
GI_10835237-S	IFITM2	14	0.136	0.00514	4.83	1.52E-06	0.000108
GI_21361584-S	PALLD	14	0.134	0.0130	4.76	2.20E-06	0.000150
GI_40254972-S	PRR5L	14	0.133	0.0115	4.74	2.38E-06	0.000160
GI_20986530-I	MAPK1	14	0.131	0.00254	4.62	4.33E-06	0.000274
GI_6005957-S	VSIG4	14	0.129	0.0128	4.56	5.54E-06	0.000339
GI_4557504-S	CYBA	14	0.124	0.00268	4.37	1.33E-05	0.000742
GI_5454165-S	VTI1B	14	0.122	0.00271	4.29	1.94E-05	0.00103
GI_15812191-S	FBXO6	14	0.121	0.00678	4.28	2.01E-05	0.00105
GI_5174428-S	ACAA2	14	0.121	0.00319	4.28	2.04E-05	0.00107
GI_21361911-S	DNAJC1	14	0.120	0.00335	4.23	2.51E-05	0.00126
GI_31982935-S	SGPL1	14	0.119	0.00281	4.22	2.66E-05	0.00132
GI_38679886-A	SRI	14	0.120	0.00264	4.22	2.67E-05	0.00132
GI_31542585-S	EIF4EBP2	14	0.119	0.00209	4.18	3.15E-05	0.00152
GI_22538439-I	CTSC	14	0.118	0.00340	4.16	3.41E-05	0.00162
GI_24797070-S	HLA-E	14	0.116	0.00298	4.09	4.54E-05	0.00205
GI_41055203-A	KLC1	14	0.115	0.00228	4.04	5.77E-05	0.00251
GI_27883865-S	RIC8A	14	0.114	0.00289	4.03	5.80E-05	0.00252
GI_38683837-S	CD47	14	0.113	0.00334	3.99	6.86E-05	0.00291
GI_4809272-S	ANXA4	14	0.112	0.00293	3.95	8.09E-05	0.00332
GI_20357549-S	TMEM50A	14	0.111	0.00302	3.92	9.31E-05	0.00372
Hs.273124-S	---	14	0.111	0.0172	3.91	9.55E-05	0.00380
GI_40254883-S	SSH1	14	0.108	0.00472	3.84	0.000131	0.00493
GI_38570088-S	RCBTB2	14	0.108	0.00460	3.83	0.000134	0.00500
GI_4557830-S	PCBD1	14	0.108	0.00329	3.83	0.000136	0.00506
GI_28872747-S	HIST3H2A	14	0.106	0.0137	3.74	0.000190	0.00669
GI_42660445-S	---	14	0.106	0.00325	3.73	0.000197	0.00689
GI_4503536-S	EIF4EBP3	14	-0.105	-0.00502	-3.71	0.000216	0.00742
GI_14141194-S	SDF2	14	0.105	0.00186	3.69	0.000237	0.00803
GI_31543395-S	PGAM1	14	0.102	0.00329	3.61	0.000321	0.0103
GI_29736248-S	GNG2	14	0.102	0.00492	3.60	0.000331	0.0106
GI_34222257-S	UFD1L	14	0.102	0.00193	3.58	0.000355	0.0111
GI_13236586-S	TMEM43	14	0.102	0.00193	3.57	0.000367	0.0114
GI_17978490-I	CD97	14	0.100	0.0119	3.56	0.000391	0.0120
GI_31377633-S	GBP4	14	0.101	0.00320	3.55	0.000396	0.0121
GI_20127456-S	GYG1	14	0.101	0.00196	3.55	0.000403	0.0123
GI_21687150-S	SLC25A43	14	0.100	0.00335	3.54	0.000414	0.0125
GI_22265328-S	CRADD	14	0.0991	0.00503	3.50	0.000475	0.0140
GI_4502676-S	CD58	14	0.0991	0.00400	3.50	0.000482	0.0141
GI_22538439-A	CTSC	14	0.0978	0.00336	3.45	0.000574	0.0164
GI_38524617-S	FMNL2	14	0.0973	0.0170	3.44	0.000598	0.0169
GI_4503020-S	CPT1A	14	0.0957	0.0138	3.38	0.000734	0.0198
GI_25777679-I	RASSF1	14	0.0957	0.0125	3.38	0.000738	0.0199
GI_13775601-A	SIRT2	14	0.0955	0.00616	3.38	0.000757	0.0203
GI_31543813-S	TMSB10	14	0.0960	0.00155	3.36	0.000800	0.0213
GI_4757755-S	ANXA2	14	0.0953	0.00222	3.35	0.000823	0.0218
GI_30089943-I	---	14	0.0945	0.00256	3.33	0.000892	0.0231
GI_37541953-S	---	14	0.0942	0.0146	3.33	0.000894	0.0231
GI_7705816-S	ZNF706	14	-0.0937	-0.00228	-3.30	0.000997	0.0251
GI_32967275-A	UBE2A	14	0.0938	0.00167	3.29	0.001034	0.0259
GI_7706157-S	PLAC8	14	-0.0930	-0.00351	-3.28	0.001056	0.0263
GI_17986259-A	MYL6	14	0.0933	0.00154	3.27	0.001114	0.0275
GI_34916047-S	TMTC1	14	0.0922	0.0115	3.26	0.001150	0.0282
GI_42518069-S	TJP2	14	0.0921	0.00460	3.25	0.001166	0.0285
GI_4507310-S	SUPT4H1	14	0.0925	0.00217	3.25	0.001167	0.0285
GI_30149745-S	SHISA4	14	0.0918	0.0107	3.24	0.001208	0.0292

GI_17572806-S	ROPN1L	14	0.0917	0.00883	3.24	0.001215	0.0293
GI_20336472-I	BCL7B	14	0.0911	0.00498	3.22	0.001316	0.0311
GI_19923135-S	CX3CR1	14	0.0904	0.00676	3.20	0.001426	0.0332
GI_13129033-S	AHNAK	14	0.0902	0.0114	3.19	0.001463	0.0339
GI_4502100-S	ANXA1	14	0.0899	0.00407	3.17	0.001538	0.0353
GI_4505670-S	PDE6D	14	0.0900	0.00193	3.17	0.001587	0.0362
GI_23110994-A	MS4A4A	14	0.0894	0.0142	3.16	0.001620	0.0368
GI_22538438-I	CTSC	14	0.0892	0.00287	3.15	0.001699	0.0382
hmm3151-S	FAM177A1	14	0.0885	0.00677	3.13	0.001807	0.0400
GI_20561649-S	ZNF319	14	0.0880	0.00336	3.10	0.001951	0.0425
GI_22538474-S	UBB	14	0.0880	0.00183	3.09	0.002040	0.0440
GI_4557885-S	ITGB2	14	0.0878	0.00216	3.09	0.002053	0.0443
GI_7662289-S	IQSEC1	14	0.0875	0.00322	3.09	0.002067	0.0445
GI_34222336-S	C3orf14	14	0.0874	0.00472	3.08	0.002082	0.0447
hmm36544-S	NHSL2	14	0.0865	0.0123	3.06	0.002285	0.0480
GI_31543149-S	MFSD5	14	0.0867	0.00257	3.05	0.002309	0.0484
GI_4885526-A	SH2D3C	14	0.0865	0.00254	3.05	0.002352	0.0490
GI_14249149-S	SSBP4	15	0.306	0.00591	11.2	5.81E-28	1.83E-24
GI_32454742-S	SESN2	15	0.222	0.00761	7.99	3.06E-15	1.42E-12
GI_4502848-S	TBCB	15	0.210	0.00284	7.44	1.88E-13	6.20E-11
GI_9910391-S	AGPAT4	15	0.206	0.00780	7.41	2.39E-13	7.65E-11
GI_7662347-S	MLXIP	15	-0.203	-0.00462	-7.26	6.67E-13	1.97E-10
GI_4503744-S	FLNA	15	0.199	0.00474	7.12	1.88E-12	4.91E-10
GI_4557252-S	ADAM8	15	0.198	0.00432	7.09	2.30E-12	5.93E-10
GI_5453861-S	PDE4A	15	0.187	0.00580	6.69	3.39E-11	7.09E-09
GI_45433498-S	ZDHHC18	15	0.187	0.00377	6.67	3.95E-11	8.11E-09
GI_10880123-A	CYTH2	15	0.187	0.00372	6.65	4.38E-11	8.96E-09
GI_30240931-S	EHD1	15	0.185	0.00385	6.59	6.37E-11	1.23E-08
GI_4504756-S	ITGAL	15	0.180	0.00546	6.43	1.79E-10	3.19E-08
GI_22779872-S	FAM89B	15	0.180	0.00444	6.40	2.15E-10	3.72E-08
GI_31543430-S	PPP1CA	15	0.176	0.00328	6.24	6.13E-10	9.47E-08
GI_13194196-S	KIF13B	15	0.176	0.00293	6.23	6.19E-10	9.53E-08
GI_22547178-S	TRPV2	15	0.173	0.00473	6.18	8.69E-10	1.30E-07
GI_42734309-S	FAM53B	15	0.171	0.00530	6.10	1.43E-09	2.08E-07
GI_24430213-S	IL10RA	15	0.169	0.00321	5.99	2.79E-09	3.79E-07
GI_4503554-S	ELF4	15	0.166	0.00341	5.88	5.13E-09	6.62E-07
GI_22547192-A	---	15	0.157	0.00342	5.56	3.38E-08	3.68E-06
GI_21312133-S	MED15	15	0.154	0.00365	5.46	5.74E-08	5.98E-06
GI_21735617-A	APOL1	15	0.153	0.00420	5.43	6.60E-08	6.79E-06
GI_38679906-S	TPRG1L	15	0.154	0.00270	5.43	6.73E-08	6.89E-06
GI_34147645-S	PLOD3	15	0.150	0.00354	5.33	1.16E-07	1.14E-05
GI_37552547-S	MAST3	15	0.148	0.00306	5.25	1.79E-07	1.66E-05
GI_34452697-S	ACTN4	15	0.148	0.00358	5.24	1.86E-07	1.71E-05
GI_40254469-S	SPN	15	0.146	0.00485	5.18	2.54E-07	2.27E-05
GI_7705876-S	HN1	15	0.144	0.00508	5.13	3.43E-07	2.98E-05
GI_37550526-S	HEG1	15	0.143	0.00606	5.07	4.60E-07	3.82E-05
GI_31543175-S	FAM134A	15	0.143	0.00306	5.07	4.63E-07	3.83E-05
GI_17978488-A	CD97	15	0.141	0.00571	4.99	6.80E-07	5.44E-05
GI_41281560-S	CLSTN1	15	0.141	0.00259	4.96	7.85E-07	6.18E-05
GI_38373674-A	RAPGEF1	15	0.140	0.00385	4.96	8.10E-07	6.32E-05
GI_38016920-S	RAB35	15	0.139	0.00216	4.89	1.12E-06	8.33E-05
GI_13929468-S	B4GALT3	15	0.138	0.00272	4.88	1.18E-06	8.76E-05
GI_5453959-S	RABAC1	15	0.138	0.00271	4.88	1.20E-06	8.89E-05
GI_31341881-S	C6orf1	15	0.139	0.00199	4.87	1.25E-06	9.19E-05
GI_4502564-S	CAPNS1	15	0.137	0.00430	4.85	1.39E-06	0.000102
GI_4753160-S	GTF3C1	15	0.137	0.00406	4.85	1.40E-06	0.000102
GI_22538458-A	NCOA1	15	0.137	0.00298	4.85	1.41E-06	0.000103
GI_30795206-S	PPP2R5B	15	0.135	0.00322	4.79	1.90E-06	0.000131
GI_34452731-S	PREX1	15	0.134	0.00273	4.72	2.67E-06	0.000178
GI_4885060-S	AKT1	15	0.131	0.00320	4.65	3.68E-06	0.000238
GI_28178831-S	IDH2	15	0.128	0.00254	4.53	6.50E-06	0.000395
GI_37551220-S	ZSWIM8	15	0.126	0.00243	4.45	9.27E-06	0.000543
GI_42734435-S	EFHD2	15	0.125	0.00483	4.43	1.01E-05	0.000586
GI_22547113-A	MRPL10	15	0.125	0.00350	4.41	1.11E-05	0.000640
GI_20302162-S	PIP4K2A	15	0.125	0.00325	4.41	1.12E-05	0.000642
GI_10947033-S	MXD4	15	0.125	0.00268	4.40	1.19E-05	0.000677
GI_20336261-S	GGA1	15	0.124	0.00305	4.39	1.23E-05	0.000695
GI_6006019-S	MAD2L2	15	0.124	0.00451	4.39	1.24E-05	0.000703
GI_5174578-S	FOXO4	15	0.122	0.00363	4.33	1.64E-05	0.000888
GI_19387853-S	NTNG2	15	0.122	0.00473	4.32	1.67E-05	0.000899
GI_22907038-S	APOBEC3C	15	0.122	0.00419	4.32	1.70E-05	0.000916
GI_34147566-S	GUK1	15	0.121	0.00246	4.27	2.13E-05	0.00111
GI_5031962-S	STUB1	15	0.121	0.00248	4.27	2.14E-05	0.00111
GI_7706468-S	KLF2	15	0.120	0.00468	4.25	2.25E-05	0.00115
GI_39777595-S	TGIF2	15	-0.120	-0.00237	-4.24	2.35E-05	0.00119
GI_31543978-S	ZBTB16	15	-0.119	-0.00613	-4.22	2.67E-05	0.00132
GI_7549806-S	FBXW2	15	0.119	0.00305	4.19	3.03E-05	0.00147
GI_34304384-S	YKT6	15	0.120	0.00154	4.18	3.13E-05	0.00151
GI_31742531-S	DIAPH1	15	0.118	0.00209	4.17	3.31E-05	0.00159
GI_4557468-S	AP2B1	15	0.118	0.00311	4.15	3.50E-05	0.00166
GI_32483393-A	CLTB	15	0.117	0.00266	4.13	3.89E-05	0.00180
GI_4826975-S	RCE1	15	0.116	0.00297	4.08	4.83E-05	0.00216
GI_27894367-S	NOTCH1	15	0.115	0.00281	4.07	4.99E-05	0.00222
GI_24308106-S	SZRD1	15	0.115	0.00290	4.06	5.14E-05	0.00227
GI_39995073-A	CDKN2D	15	0.114	0.00387	4.05	5.55E-05	0.00243
GI_21361093-S	IKBKG	15	0.115	0.00187	4.02	6.07E-05	0.00263
GI_31083143-A	AXIN1	15	0.114	0.00253	4.01	6.56E-05	0.00280

GI_8923476-S	TMEM160	15	0.113	0.00231	3.99	6.89E-05	0.00292
GI_4505904-S	PMM1	15	0.113	0.00341	3.99	7.05E-05	0.00297
GI_7706342-S	FAM96B	15	0.114	0.00166	3.98	7.39E-05	0.00308
GI_39725676-S	NUCB1	15	0.112	0.00316	3.96	8.01E-05	0.00330
GI_32171185-S	BCAP31	15	0.112	0.00197	3.95	8.43E-05	0.00342
GI_39725675-S	CDK2AP2	15	0.111	0.00336	3.94	8.64E-05	0.00350
GI_6552331-S	FLOT1	15	0.112	0.00183	3.93	9.11E-05	0.00366
GI_34147718-S	ZNF688	15	0.111	0.00315	3.91	9.64E-05	0.00382
GI_5454109-S	TESK1	15	0.111	0.00246	3.90	9.95E-05	0.00393
GI_4507840-S	UQCRC1	15	0.110	0.00201	3.88	0.000110	0.00425
GI_4502896-S	CLPTM1	15	0.110	0.00249	3.88	0.000112	0.00431
GI_19923916-S	SHKBP1	15	0.109	0.00349	3.86	0.000118	0.00451
GI_25286702-S	KIAA2013	15	0.110	0.00164	3.85	0.000125	0.00474
GI_21361780-S	MAP7D1	15	0.109	0.00203	3.84	0.000129	0.00487
GI_24475860-S	PHPT1	15	0.109	0.00199	3.84	0.000131	0.00492
GI_11321629-S	NR1H2	15	0.109	0.00214	3.82	0.000138	0.00512
GI_34147606-S	CNPPD1	15	0.108	0.00211	3.78	0.000162	0.00585
GI_21361508-S	---	15	0.107	0.00181	3.76	0.000181	0.00643
GI_9910203-S	---	15	0.106	0.00326	3.75	0.000184	0.00653
GI_4557552-S	EMD	15	0.106	0.00291	3.75	0.000189	0.00665
GI_34147601-S	ARHGDIA	15	0.106	0.00290	3.74	0.000192	0.00675
GI_7657045-S	UBE2S	15	0.106	0.00349	3.74	0.000193	0.00678
GI_21361484-S	TBC1D10B	15	0.106	0.00288	3.73	0.000198	0.00693
GI_7662409-S	SBNO2	15	0.105	0.00419	3.72	0.000205	0.00712
GI_38372922-A	BSG	15	0.105	0.00324	3.72	0.000205	0.00712
GI_10864068-S	TMEM8A	15	0.104	0.00343	3.67	0.000253	0.00848
GI_32481208-A	MAPKAPK2	15	0.104	0.00235	3.67	0.000254	0.00852
GI_40068460-S	TWF2	15	0.104	0.00297	3.67	0.000257	0.00858
GI_14149701-S	RNF167	15	0.104	0.00278	3.66	0.000258	0.00860
GI_37552347-S	GPR108	15	0.104	0.00196	3.65	0.000270	0.00893
GI_13259507-A	DCTN1	15	0.104	0.00230	3.65	0.000274	0.00901
GI_23397652-S	PIGT	15	0.104	0.00201	3.64	0.000283	0.00926
GI_31543199-S	TMUB1	15	0.103	0.00285	3.62	0.000301	0.00975
GI_40254968-S	NUDT18	15	0.102	0.00300	3.62	0.000312	0.0101
GI_23110949-S	CTSD	15	0.102	0.00345	3.61	0.000319	0.0102
GI_27544940-S	MYO1F	15	0.102	0.00345	3.59	0.000345	0.0109
GI_38455425-S	CITED4	15	-0.101	-0.00342	-3.58	0.000354	0.0111
GI_38016942-S	ORAI1	15	0.101	0.00376	3.58	0.000361	0.0113
GI_4758925-S	PITPNM1	15	0.101	0.00358	3.55	0.000398	0.0122
GI_38570148-S	WWC3	15	0.101	0.00205	3.55	0.000404	0.0123
GI_37537720-S	B3GALT6	15	-0.101	-0.00205	-3.55	0.000405	0.0123
GI_14149741-S	CALCOCO1	15	0.100	0.00276	3.54	0.000421	0.0127
GI_14917108-S	AP2M1	15	0.101	0.00199	3.54	0.000421	0.0127
GI_37546496-S	SSU72	15	0.0985	0.00217	3.47	0.000543	0.0157
GI_4557258-S	ADCY9	15	0.0977	0.00531	3.45	0.000575	0.0164
GI_41281455-S	RAB11FIP3	15	0.0971	0.00245	3.42	0.000641	0.0179
GI_8922347-S	TMEM39B	15	-0.0972	-0.00177	-3.41	0.000664	0.0184
GI_12408655-S	CAPN1	15	0.0964	0.00199	3.39	0.000724	0.0197
GI_33636765-S	LHPP	15	-0.0957	-0.00203	-3.37	0.000788	0.0210
GI_6005793-S	PRAF2	15	0.0949	0.00340	3.35	0.000833	0.0219
GI_34335250-A	DLGAP4	15	0.0949	0.00261	3.35	0.000847	0.0222
GI_4507102-S	SNAPC2	15	0.0947	0.00256	3.34	0.000865	0.0225
GI_42476331-S	SH3BGRL3	15	0.0944	0.00454	3.34	0.000873	0.0227
GI_23510449-S	MPST	15	0.0940	0.00343	3.32	0.000930	0.0238
GI_4507508-S	TIMP1	15	0.0939	0.00517	3.32	0.000936	0.0240
GI_33356147-S	FMNL1	15	0.0939	0.00265	3.31	0.000954	0.0244
GI_14249553-S	SPRYD3	15	0.0939	0.00257	3.31	0.000965	0.0246
GI_23397668-S	ARPC4	15	0.0940	0.00219	3.31	0.000968	0.0246
GI_4504196-S	GTF2F1	15	0.0933	0.00221	3.29	0.001046	0.0261
GI_21361267-S	TNIP1	15	0.0931	0.00190	3.27	0.001104	0.0273
GI_4506026-S	PPP4C	15	0.0928	0.00237	3.27	0.001112	0.0275
GI_16753213-S	PFN1	15	0.0930	0.00180	3.27	0.001115	0.0275
GI_24307876-S	POR	15	0.0925	0.00309	3.26	0.001130	0.0278
GI_18201904-S	GPI	15	0.0926	0.00200	3.26	0.001153	0.0283
GI_16357476-S	CDC34	15	0.0915	0.00287	3.23	0.001284	0.0306
GI_7669500-S	LAMP1	15	0.0919	0.00168	3.22	0.001297	0.0309
GI_6912247-S	DNPEP	15	0.0913	0.00261	3.22	0.001319	0.0312
GI_45333920-S	ASB8	15	0.0911	0.00264	3.21	0.001349	0.0318
GI_38146105-S	SLC52A2	15	0.0910	0.00300	3.21	0.001365	0.0321
GI_22095348-S	WDTC1	15	0.0909	0.00236	3.20	0.001398	0.0327
GI_40018625-S	MOB3A	15	0.0907	0.00212	3.19	0.001455	0.0338
GI_4504750-S	ITGA5	15	0.0905	0.00243	3.19	0.001462	0.0339
GI_20127505-S	PMVK	15	0.0905	0.00209	3.18	0.001491	0.0345
GI_31377621-S	DEDD2	15	0.0897	0.00195	3.15	0.001654	0.0374
GI_33859747-S	CENPBD1P1	15	0.0891	0.00519	3.15	0.001681	0.0379
GI_23943871-S	SMAP2	15	-0.0896	-0.00171	-3.14	0.001707	0.0383
GI_19718752-S	BAP1	15	0.0889	0.00270	3.14	0.001757	0.0392
GI_13569961-S	RAB1B	15	0.0880	0.00411	3.11	0.001931	0.0422
GI_24475655-S	FBXW4	15	0.0882	0.00173	3.10	0.001995	0.0432
GI_21703709-S	SHISA5	15	0.0881	0.00190	3.10	0.002002	0.0433
GI_17105397-I	WDR1	15	0.0875	0.00273	3.09	0.002072	0.0446
GI_4507204-S	SRF	15	0.0874	0.00239	3.08	0.002124	0.0454
GI_11345483-S	ZFAND3	15	0.0869	0.00210	3.06	0.002282	0.0480
GI_40538727-S	PLEKHM1	15	0.0865	0.00234	3.04	0.002377	0.0494
GI_32307157-S	AGAP3	15	0.0864	0.00237	3.04	0.002398	0.0497
GI_18375627-A	PRRC2A	15	0.0866	0.00180	3.04	0.002408	0.0499
GI_4507544-S	GPR137B	16	0.297	0.00669	10.9	1.90E-26	4.50E-23

GI_31543930-S	VAMP5	16	0.226	0.00557	8.13	1.05E-15	5.32E-13
GI_7705752-S	C1QA	16	0.214	0.0134	7.70	2.82E-14	1.06E-11
GI_34335231-S	CKB	16	0.210	0.0108	7.54	8.90E-14	3.17E-11
GI_34222243-S	RHOC	16	0.209	0.00744	7.51	1.11E-13	3.88E-11
GI_27735072-S	CYP4F22	16	0.208	0.0301	7.50	1.20E-13	4.16E-11
GI_42716282-S	USB1	16	0.206	0.00291	7.31	4.65E-13	1.42E-10
GI_4557440-S	CDKN1C	16	0.193	0.0118	6.92	7.03E-12	1.67E-09
GI_42476319-S	SUSD1	16	0.189	0.00418	6.75	2.24E-11	4.88E-09
GI_38016929-A	OASL	16	0.186	0.00793	6.65	4.39E-11	8.96E-09
GI_14165470-S	CDC42EP4	16	0.177	0.00614	6.31	3.83E-10	6.24E-08
GI_22202618-A	CTSL	16	0.175	0.0117	6.27	5.02E-10	7.97E-08
GI_31543214-S	MT2A	16	0.175	0.00560	6.26	5.35E-10	8.47E-08
GI_32698963-S	VMO1	16	0.174	0.0236	6.22	6.63E-10	1.02E-07
GI_11038661-S	C1QB	16	0.174	0.0152	6.21	7.11E-10	1.08E-07
GI_22049497-S	LYPD2	16	0.173	0.0113	6.18	8.60E-10	1.29E-07
GI_38679953-A	ABR	16	0.169	0.00310	6.00	2.64E-09	3.64E-07
GI_33620766-S	SCARB1	16	-0.167	-0.00540	-5.94	3.78E-09	5.02E-07
GI_30157733-S	---	16	0.166	0.00819	5.92	4.16E-09	5.51E-07
GI_8923562-S	TESC	16	0.160	0.00433	5.70	1.52E-08	1.79E-06
GI_13775223-S	APH1B	16	0.160	0.00327	5.68	1.68E-08	1.94E-06
GI_34328936-S	CD63	16	0.158	0.00480	5.61	2.54E-08	2.84E-06
GI_45387924-S	LYPD2	16	0.156	0.0124	5.57	3.05E-08	3.35E-06
GI_34485718-S	E2F2	16	0.156	0.00549	5.55	3.43E-08	3.73E-06
GI_4504436-S	HMOX1	16	0.154	0.00528	5.46	5.66E-08	5.91E-06
GI_18087856-S	UBE2F	16	0.153	0.00284	5.42	7.08E-08	7.20E-06
GI_31543384-S	PCYT1A	16	0.149	0.00440	5.28	1.54E-07	1.44E-05
GI_33457315-S	PLEKHO2	16	0.148	0.00514	5.27	1.62E-07	1.51E-05
GI_8924245-S	BATF3	16	0.146	0.00526	5.19	2.40E-07	2.16E-05
GI_12545400-A	ICAM4	16	0.145	0.00699	5.17	2.76E-07	2.44E-05
GI_42658645-S	---	16	0.143	0.00337	5.06	4.80E-07	3.96E-05
GI_42476329-S	SFTPD	16	0.141	0.0100	5.00	6.55E-07	5.29E-05
GI_26787979-A	IL15	16	0.138	0.00370	4.90	1.10E-06	8.29E-05
GI_32307143-S	PLOD1	16	0.136	0.00428	4.82	1.65E-06	0.000115
GI_7706275-A	TPPP3	16	0.134	0.00653	4.77	2.10E-06	0.000144
GI_19913411-A	MVP	16	0.133	0.00342	4.69	2.97E-06	0.000196
GI_40807488-S	EMR1	16	0.132	0.00448	4.66	3.48E-06	0.000227
GI_4757913-S	CASP5	16	0.131	0.00466	4.63	3.96E-06	0.000255
GI_21071009-S	TCN2	16	0.131	0.00536	4.63	3.98E-06	0.000256
GI_4557384-S	C3	16	0.127	0.00635	4.50	7.60E-06	0.000456
GI_20127497-S	RRAS	16	0.126	0.00563	4.48	8.00E-06	0.000476
GI_33636716-S	FAM214B	16	0.126	0.00481	4.46	8.79E-06	0.000519
GI_29337288-S	ABI3	16	0.126	0.00338	4.46	9.04E-06	0.000533
GI_9910341-S	SLAMF8	16	0.124	0.00651	4.38	1.27E-05	0.000717
GI_42490763-S	ELOVL1	16	0.124	0.00219	4.37	1.34E-05	0.000751
GI_23238227-S	CHST7	16	0.123	0.00383	4.35	1.50E-05	0.000826
GI_31541779-S	CYB561D2	16	0.123	0.00193	4.32	1.66E-05	0.000898
GI_23312365-S	TNFRSF1B	16	0.121	0.00271	4.27	2.11E-05	0.00110
GI_27754203-S	---	16	0.119	0.00350	4.21	2.72E-05	0.00134
GI_19923143-S	MYD88	16	0.119	0.00254	4.18	3.06E-05	0.00148
GI_8923465-S	TMEM127	16	0.118	0.00278	4.15	3.55E-05	0.00168
GI_31543450-S	PRELID1	16	0.118	0.00251	4.15	3.60E-05	0.00170
GI_20127596-S	HES4	16	0.116	0.00825	4.11	4.29E-05	0.00196
GI_19923781-S	LILRB1	16	0.114	0.00410	4.02	6.20E-05	0.00268
GI_31542755-S	PAQR4	16	0.113	0.00314	3.97	7.48E-05	0.00311
GI_34147574-S	HTATIP2	16	0.113	0.00215	3.96	7.93E-05	0.00327
GI_4758085-S	CSRP1	16	0.112	0.00211	3.96	8.06E-05	0.00331
GI_39725697-S	CALHM2	16	0.112	0.00366	3.95	8.22E-05	0.00336
GI_13236578-S	SLC27A3	16	0.111	0.00423	3.94	8.72E-05	0.00353
GI_38327597-I	RGS12	16	0.111	0.00489	3.93	9.09E-05	0.00365
GI_29648312-S	ASPHD2	16	0.111	0.00239	3.92	9.31E-05	0.00372
GI_23110999-S	MS4A7	16	0.109	0.00517	3.84	0.000129	0.00487
GI_7662125-S	SIPA1L1	16	0.108	0.00245	3.79	0.000159	0.00575
Hs.284257-S	---	16	0.105	0.0174	3.73	0.000202	0.00702
GI_7661727-S	LAMTOR2	16	0.106	0.00181	3.72	0.000207	0.00716
GI_19718758-A	MYOF	16	0.105	0.00372	3.69	0.000230	0.00783
GI_28872795-S	CEBPB	16	0.103	0.00585	3.63	0.000298	0.00969
GI_4502792-S	CEACAM3	16	0.102	0.0123	3.61	0.000314	0.0101
GI_24797075-S	HLA-DPB1	16	0.103	0.00200	3.61	0.000325	0.0104
GI_4505020-S	LRPAP1	16	0.102	0.00198	3.58	0.000356	0.0111
GI_21314629-S	C3AR1	16	0.101	0.00602	3.56	0.000382	0.0118
GI_11545764-S	CXCL16	16	0.101	0.00346	3.56	0.000391	0.0120
GI_32895366-I	LILRA5	16	0.0991	0.00525	3.50	0.000476	0.0140
GI_13376321-S	---	16	0.0991	0.00489	3.50	0.000480	0.0141
GI_23510436-A	TNFRSF8	16	0.0980	0.00396	3.46	0.000556	0.0160
GI_6031195-S	PLAGL2	16	0.0977	0.00331	3.45	0.000583	0.0166
GI_4758069-S	PTGDR2	16	0.0974	0.00555	3.44	0.000591	0.0167
GI_16579887-S	FBP1	16	0.0964	0.00354	3.40	0.000688	0.0188
GI_41393564-S	ITPK1	16	0.0962	0.00295	3.39	0.000708	0.0193
GI_7710155-S	WARS	16	0.0956	0.00429	3.38	0.000756	0.0203
GI_23110923-S	PSMB10	16	0.0964	0.00150	3.37	0.000761	0.0204
GI_20357564-S	CSTB	16	0.0954	0.00182	3.35	0.000833	0.0219
GI_34147669-S	SLC39A1	16	0.0948	0.00318	3.34	0.000849	0.0222
Hs.501087-S	---	16	0.0946	0.00838	3.34	0.000851	0.0223
GI_38372936-A	CHMP2A	16	0.0952	0.00136	3.32	0.000918	0.0236
GI_22907051-S	ARPC1A	16	0.0956	0.00116	3.32	0.000921	0.0237
GI_9938031-S	SLC2A6	16	0.0938	0.00283	3.31	0.000962	0.0245
GI_37549554-S	---	16	0.0933	0.0145	3.30	0.000994	0.0251

GI_39930526-S	TNNT1	16	0.0932	0.0126	3.30	0.001012	0.0254
GI_4809285-A	IRF7	16	-0.0934	-0.00317	-3.29	0.001016	0.0255
GI_21314780-S	NEURL1	16	0.0921	0.00826	3.26	0.001164	0.0284
GI_5729827-S	TRAFD1	16	0.0924	0.00183	3.25	0.001199	0.0291
GI_20986498-A	MAPK7	16	0.0921	0.00250	3.24	0.001209	0.0292
GI_32189356-S	MRAS	16	0.0917	0.00468	3.24	0.001232	0.0296
GI_27477265-S	FAM212B	16	0.0913	0.00334	3.22	0.001302	0.0309
GI_6912517-S	ACOT9	16	0.0909	0.00307	3.21	0.001379	0.0323
Hs.136355-S	---	16	0.0906	0.0118	3.20	0.001388	0.0325
GI_5032056-S	S100A11	16	0.0908	0.00307	3.20	0.001403	0.0328
GI_11995467-S	IFITM3	16	0.0901	0.00354	3.18	0.001505	0.0347
GI_4885234-S	FGR	16	0.0903	0.00209	3.18	0.001524	0.0350
GI_42822885-S	---	16	0.0893	0.00452	3.15	0.001653	0.0374
GI_30179906-A	PILRA	16	0.0894	0.00317	3.15	0.001657	0.0375
GI_4507868-S	VASP	16	0.0898	0.00170	3.15	0.001659	0.0375
GI_21389496-S	CCDC12	16	0.0900	0.00135	3.15	0.001695	0.0382
GI_38044287-A	GSN	16	0.0889	0.00289	3.13	0.001765	0.0393
GI_42542393-S	LPCAT3	16	0.0878	0.00173	3.08	0.002102	0.0450
GI_37540588-S	C4orf48	16	0.0869	0.00314	3.07	0.002214	0.0468
GI_10835170-S	IFNG	17	0.301	0.0167	11.1	2.55E-27	7.11E-24
GI_27436944-I	LMNA	17	0.205	0.0128	7.36	3.27E-13	1.02E-10
GI_13899218-S	GABARAPL1	17	0.201	0.00859	7.20	1.05E-12	2.99E-10
GI_5360207-A	IDS	17	0.200	0.00601	7.18	1.23E-12	3.39E-10
GI_27436944-A	LMNA	17	0.184	0.0132	6.58	6.73E-11	1.29E-08
GI_31880337-S	TUBA1C	17	0.181	0.00375	6.44	1.71E-10	3.06E-08
GI_15812223-A	TRERF1	17	0.180	0.00466	6.41	2.12E-10	3.67E-08
GI_12707565-S	DUSP5	17	0.169	0.00689	6.01	2.42E-09	3.41E-07
GI_5174516-S	SMAD7	17	0.166	0.0106	5.94	3.72E-09	4.95E-07
GI_34328908-A	DUSP4	17	0.163	0.0127	5.83	7.16E-09	8.99E-07
GI_4504720-S	IRF1	17	0.163	0.00639	5.80	8.17E-09	1.01E-06
GI_42657338-S	---	17	0.162	0.00627	5.77	9.86E-09	1.20E-06
GI_4503298-S	BHLHE40	17	0.154	0.00698	5.49	4.95E-08	5.23E-06
GI_4507728-S	TUBB2A	17	0.154	0.00945	5.49	4.98E-08	5.25E-06
GI_19743904-A	RORA	17	0.149	0.00629	5.28	1.50E-07	1.41E-05
GI_13699866-S	MAP1LC3B	17	0.145	0.00418	5.14	3.15E-07	2.75E-05
GI_17978496-A	CDKN1A	17	0.144	0.00671	5.13	3.34E-07	2.90E-05
GI_7019524-S	SERTAD1	17	0.143	0.00633	5.09	4.21E-07	3.53E-05
GI_31343528-S	CCDC71L	17	0.140	0.00435	4.98	7.19E-07	5.71E-05
GI_28416425-I	ZNF655	17	0.141	0.00239	4.98	7.44E-07	5.88E-05
GI_37542672-S	---	17	0.138	0.00409	4.89	1.12E-06	8.33E-05
GI_4758211-S	DUSP8	17	0.138	0.00573	4.89	1.13E-06	8.46E-05
GI_40254960-S	DERL1	17	0.135	0.00331	4.77	2.06E-06	0.000142
GI_22027510-A	CDCA4	17	0.131	0.00394	4.63	3.99E-06	0.000256
GI_37655156-S	KLF6	17	0.130	0.00598	4.60	4.63E-06	0.000291
GI_38201674-S	SDC4	17	0.125	0.0163	4.42	1.07E-05	0.000621
GI_14249657-S	MIR22HG	17	0.124	0.00494	4.40	1.15E-05	0.000659
GI_4885516-S	NFIL3	17	0.124	0.00553	4.40	1.19E-05	0.000677
GI_38327528-I	INSIG1	17	0.121	0.00547	4.29	1.88E-05	0.000999
GI_34577058-S	PLIN2	17	0.121	0.00468	4.29	1.93E-05	0.00102
GI_15718683-S	SERTAD3	17	0.121	0.00348	4.27	2.09E-05	0.00109
GI_4505878-S	PLEK	17	0.118	0.00496	4.19	2.98E-05	0.00145
GI_34147356-S	DDA1	17	0.118	0.00277	4.18	3.16E-05	0.00152
GI_22035666-S	TOB1	17	0.118	0.00317	4.17	3.32E-05	0.00159
GI_4501944-S	ADM	17	0.117	0.00912	4.15	3.55E-05	0.00168
GI_40018632-S	IDI1	17	0.113	0.00260	3.99	6.90E-05	0.00292
GI_18087854-S	DYNLL2	17	0.113	0.00409	3.99	6.97E-05	0.00295
GI_30026033-S	SLC35B2	17	0.113	0.00337	3.99	6.99E-05	0.00295
GI_17986282-S	TUBA1A	17	0.112	0.00450	3.95	8.11E-05	0.00332
GI_20149649-S	CHST11	17	0.111	0.00440	3.92	9.42E-05	0.00375
GI_15812192-S	FBXO7	17	0.111	0.00209	3.91	9.87E-05	0.00390
GI_4505460-S	ENC1	17	0.110	0.00390	3.89	0.000106	0.00413
GI_4504156-S	CXCL3	17	0.110	0.0102	3.89	0.000107	0.00416
GI_10835118-S	MYO5A	17	0.110	0.00280	3.88	0.000111	0.00428
GI_4503412-S	HBEGF	17	0.110	0.00719	3.88	0.000111	0.00429
GI_45439368-S	IER5	17	0.109	0.00455	3.85	0.000123	0.00469
GI_5729764-S	CGRRF1	17	0.106	0.00359	3.75	0.000185	0.00655
GI_31543823-S	TREM1	17	0.106	0.00522	3.74	0.000192	0.00676
GI_14574570-S	BCL2A1	17	0.106	0.00559	3.74	0.000193	0.00678
GI_38524593-S	TIPARP	17	0.105	0.00432	3.73	0.000200	0.00699
GI_34147657-S	DDIT3	17	0.106	0.00374	3.73	0.000201	0.00701
GI_34787408-S	RALGAPB	17	0.106	0.00276	3.72	0.000205	0.00712
GI_42656231-S	---	17	0.105	0.00315	3.72	0.000209	0.00720
GI_28872721-S	BTG3	17	0.105	0.00587	3.70	0.000223	0.00761
GI_30156248-S	---	17	0.104	0.00627	3.69	0.000236	0.00801
GI_29745613-S	TP53INP2	17	0.103	0.0177	3.65	0.000272	0.00896
GI_42658610-S	LINC-PINT	17	0.103	0.00362	3.64	0.000282	0.00924
GI_31317226-S	EGR1	17	0.103	0.00816	3.63	0.000291	0.00949
GI_4885332-S	FFAR2	17	0.102	0.00673	3.63	0.000300	0.00973
GI_31652256-S	MAFB	17	0.102	0.00616	3.62	0.000305	0.00988
GI_39725640-S	AKIRIN2	17	0.102	0.00260	3.61	0.000318	0.0102
GI_39930484-S	SLC25A25	17	0.102	0.00206	3.60	0.000333	0.0106
GI_25952110-S	TNF	17	0.101	0.0115	3.58	0.000354	0.0111
GI_28373098-A	ST8SIA4	17	0.101	0.00465	3.56	0.000381	0.0117
GI_27597060-S	UBR2	17	0.101	0.00207	3.55	0.000396	0.0121
GI_38327530-A	INSIG1	17	0.0992	0.00316	3.50	0.000480	0.0141
GI_5174460-S	HCAR3	17	0.0988	0.00844	3.49	0.000494	0.0144
GI_21362043-S	C9orf89	17	0.0985	0.00224	3.47	0.000546	0.0157

GI_32698821-S	ZFAND2A	17	0.0978	0.00291	3.45	0.000582	0.0166
GI_34147366-S	MAPKAP1	17	0.0974	0.00151	3.41	0.000670	0.0185
GI_31377612-S	MYADM	17	0.0966	0.00351	3.41	0.000671	0.0185
GI_34222319-S	JOSD1	17	0.0959	0.00283	3.38	0.000738	0.0199
GI_27735130-S	FAM117B	17	-0.0957	-0.00333	-3.38	0.000754	0.0203
GI_27485722-S	LOC284454	17	0.0951	0.00742	3.36	0.000799	0.0213
GI_21361192-S	CD44	17	0.0945	0.00285	3.33	0.000886	0.0229
GI_37539752-S	---	17	0.0937	0.00264	3.30	0.000985	0.0249
hmm23840-S	---	17	0.0915	0.00446	3.23	0.001257	0.0301
GI_20336474-A	BCL7B	17	0.0912	0.00256	3.22	0.001337	0.0315
GI_39930398-S	TP53INP2	17	0.0910	0.00647	3.21	0.001340	0.0316
GI_27886537-I	ATP2A2	17	0.0903	0.00246	3.18	0.001495	0.0345
GI_40807461-S	GZF1	17	0.0898	0.00652	3.17	0.001549	0.0355
GI_32171174-S	BUD31	17	0.0891	0.00227	3.14	0.001745	0.0390
GI_21040323-A	BCL6	17	0.0885	0.00394	3.13	0.001813	0.0401
GI_10835186-S	SOD2	17	0.0882	0.00366	3.11	0.001900	0.0416
GI_21071004-S	PHLDA2	17	0.0876	0.0171	3.10	0.001998	0.0433
GI_31542687-S	COQ10B	17	0.0880	0.00214	3.10	0.001999	0.0433
GI_22035597-S	MAP3K8	17	0.0875	0.00371	3.09	0.002046	0.0441
GI_40788016-S	TRIB1	17	0.0874	0.00410	3.09	0.002081	0.0447
GI_8923223-A	LRRFIP2	17	0.0870	0.00200	3.06	0.002253	0.0475
GI_31377584-S	SPATA2L	17	0.0865	0.00276	3.05	0.002355	0.0490
GI_34147050-S	GDPD5	18	0.302	0.00988	11.1	2.15E-27	6.36E-24
GI_4505036-S	LTBP4	18	0.281	0.00692	10.3	9.44E-24	1.49E-20
GI_18375661-A	PTPN7	18	0.253	0.00699	9.18	1.80E-19	1.52E-16
hmm525-S	SFXN3	18	0.209	0.00429	7.47	1.53E-13	5.21E-11
GI_24497439-I	IL12RB1	18	0.206	0.00561	7.39	2.72E-13	8.63E-11
GI_4758173-S	DMPK	18	0.195	0.00784	6.99	4.50E-12	1.11E-09
GI_31542746-S	COMTD1	18	0.171	0.00433	6.08	1.62E-09	2.34E-07
GI_14165459-A	NARF	18	0.170	0.00353	6.02	2.23E-09	3.17E-07
GI_30749197-A	FBXW5	18	0.168	0.00376	5.96	3.30E-09	4.43E-07
GI_45356148-S	TSPAN17	18	0.165	0.00473	5.87	5.56E-09	7.08E-07
GI_34335254-S	DCTN2	18	0.167	0.00193	5.83	6.99E-09	8.80E-07
GI_24497621-S	TRIM11	18	0.163	0.00449	5.81	7.99E-09	9.94E-07
GI_30794221-S	BTBD6	18	0.164	0.00283	5.79	8.78E-09	1.08E-06
GI_34147369-S	TNIP2	18	0.160	0.00357	5.68	1.64E-08	1.91E-06
GI_24476008-S	RHBDD2	18	0.159	0.00469	5.66	1.89E-08	2.16E-06
GI_31343485-S	RNF166	18	0.159	0.00418	5.65	1.95E-08	2.20E-06
GI_20070295-S	RNPEPL1	18	0.157	0.00363	5.57	3.13E-08	3.43E-06
GI_37552316-S	MIDN	18	0.151	0.00512	5.37	9.27E-08	9.25E-06
GI_13112053-S	FGFRL1	18	0.151	0.00550	5.36	1.00E-07	9.90E-06
GI_30151609-S	PHF19	18	0.146	0.00319	5.18	2.57E-07	2.29E-05
GI_20070291-S	CHST12	18	0.144	0.00511	5.12	3.45E-07	2.99E-05
GI_4757805-S	VPS9D1	18	0.144	0.00510	5.10	3.98E-07	3.38E-05
GI_27734692-S	C14orf80	18	0.143	0.00431	5.09	4.19E-07	3.52E-05
GI_42741656-A	---	18	0.139	0.00366	4.94	8.91E-07	6.88E-05
GI_4506138-S	CHMP1A	18	0.139	0.00302	4.91	1.03E-06	7.80E-05
GI_20544181-S	WDR13	18	0.136	0.00241	4.81	1.70E-06	0.000119
GI_34147672-S	ST6GALNAC6	18	0.135	0.00458	4.79	1.85E-06	0.000128
GI_38488711-S	TANGO2	18	0.135	0.00310	4.77	2.06E-06	0.000142
GI_38016939-S	MON1B	18	0.135	0.00272	4.75	2.27E-06	0.000154
GI_22091444-S	GFRA2	18	0.134	0.00586	4.75	2.29E-06	0.000155
GI_5803022-S	LMAN2	18	0.134	0.00299	4.74	2.39E-06	0.000161
GI_11321627-S	CTRC	18	0.133	0.00929	4.72	2.57E-06	0.000172
GI_34147603-S	STX4	18	0.132	0.00209	4.63	4.09E-06	0.000260
GI_4505492-S	OGDH	18	0.131	0.00324	4.63	4.13E-06	0.000262
GI_42661328-S	FAM195B	18	0.130	0.00462	4.62	4.14E-06	0.000262
GI_8922332-S	PLEKHJ1	18	0.131	0.00267	4.61	4.34E-06	0.000274
GI_32528285-A	ACOT7	18	0.130	0.00441	4.59	4.79E-06	0.000299
GI_19923461-S	PHF21A	18	0.131	0.00183	4.59	4.89E-06	0.000304
GI_39725688-S	TUBB4B	18	0.129	0.00426	4.57	5.34E-06	0.000329
GI_40353752-S	---	18	0.127	0.00296	4.49	7.61E-06	0.000456
GI_7019374-S	FHOD1	18	0.127	0.00263	4.47	8.53E-06	0.000505
GI_34594668-S	MOB2	18	0.123	0.00245	4.33	1.59E-05	0.000867
GI_10092596-S	MCOLN1	18	0.122	0.00343	4.31	1.77E-05	0.000945
GI_38679901-S	TMEM44	18	0.121	0.00477	4.28	1.99E-05	0.00105
GI_23199980-S	WIPF2	18	0.121	0.00247	4.26	2.15E-05	0.00111
GI_40353205-S	C6orf106	18	0.120	0.00335	4.26	2.22E-05	0.00114
GI_21686972-S	---	18	0.120	0.00445	4.25	2.26E-05	0.00115
GI_4505304-S	MYL5	18	-0.120	-0.00351	-4.24	2.39E-05	0.00121
GI_19924146-A	TCIRG1	18	0.120	0.00425	4.23	2.47E-05	0.00124
GI_40255030-S	GSDMD	18	0.120	0.00234	4.23	2.52E-05	0.00126
GI_29789109-S	TMCC3	18	0.119	0.00376	4.22	2.57E-05	0.00129
GI_41872473-S	ETV6	18	0.119	0.00394	4.22	2.61E-05	0.00130
GI_7662489-S	PPP6R1	18	0.119	0.00289	4.22	2.64E-05	0.00131
GI_40549452-S	ZBTB7A	18	0.119	0.00550	4.22	2.68E-05	0.00132
GI_42476122-S	RUSC1	18	0.119	0.00200	4.18	3.13E-05	0.00151
GI_5031816-S	KATNB1	18	0.118	0.00224	4.14	3.71E-05	0.00173
GI_4504222-S	GUSB	18	0.118	0.00205	4.13	3.83E-05	0.00178
GI_16753217-S	PURA	18	0.117	0.00250	4.13	3.89E-05	0.00180
GI_34147470-S	VPS26B	18	0.116	0.00243	4.09	4.68E-05	0.00210
GI_17978482-A	VPS16	18	0.115	0.00327	4.06	5.13E-05	0.00227
GI_20070341-S	TMEM62	18	0.115	0.00199	4.04	5.60E-05	0.00244
GI_34147460-S	TMBIM1	18	0.114	0.00236	4.01	6.53E-05	0.00279
GI_41872428-S	TRPC4AP	18	0.113	0.00198	3.98	7.21E-05	0.00302
GI_8051619-A	LIMK2	18	0.113	0.00296	3.98	7.38E-05	0.00308
GI_41350328-S	MFN2	18	0.112	0.00356	3.95	8.41E-05	0.00342

GI_37541410-S	KIAA0556	18	0.111	0.00242	3.92	9.40E-05	0.00375
GI_33469950-A	RABGGTA	18	0.111	0.00216	3.91	9.60E-05	0.00382
GI_16445440-S	TRIM26	18	0.111	0.00242	3.90	0.000101	0.00396
GI_42734322-S	ZSWIM8	18	0.110	0.00260	3.88	0.000109	0.00425
GI_42662295-S	---	18	0.107	0.00396	3.79	0.000157	0.00570
GI_5902089-S	SLC2A3	18	0.106	0.00445	3.76	0.000177	0.00631
GI_34147526-S	TP53I13	18	0.106	0.00391	3.75	0.000183	0.00648
GI_20336247-S	PCSK7	18	0.107	0.00228	3.75	0.000182	0.00648
GI_4505288-S	MVD	18	0.104	0.00569	3.67	0.000257	0.00858
GI_45269143-A	KEAP1	18	0.104	0.00205	3.65	0.000271	0.00894
GI_37655182-S	NDRG1	18	0.104	0.00187	3.64	0.000289	0.00944
GI_34996490-S	TMEM219	18	0.103	0.00236	3.63	0.000291	0.00948
GI_26787987-S	MICB	18	0.103	0.00202	3.63	0.000291	0.00949
GI_39995085-S	SLC8B1	18	-0.103	-0.00245	-3.62	0.000305	0.00986
GI_40254958-S	ATG9A	18	0.102	0.00341	3.62	0.000307	0.00993
GI_5174700-S	STK10	18	0.103	0.00221	3.62	0.000308	0.00995
GI_9951921-S	CIB1	18	0.102	0.00303	3.60	0.000329	0.0105
GI_24431972-S	CC2D1A	18	0.102	0.00286	3.60	0.000333	0.0106
GI_14211539-S	MOV10	18	0.102	0.00233	3.60	0.000336	0.0107
GI_31542324-S	CROT	18	0.102	0.00319	3.60	0.000336	0.0107
GI_45504354-S	MUSTN1	18	0.102	0.00339	3.59	0.000348	0.0110
GI_41350332-S	TBCD	18	0.101	0.00313	3.58	0.000362	0.0113
GI_39573729-S	---	18	0.101	0.00223	3.57	0.000369	0.0114
GI_31742506-A	UPP1	18	0.101	0.00335	3.57	0.000377	0.0116
GI_21361550-S	SLC4A2	18	0.101	0.00253	3.57	0.000377	0.0117
GI_37555948-S	SLC9A8	18	0.101	0.00216	3.56	0.000381	0.0117
GI_4504036-S	GNA11	18	-0.101	-0.00312	-3.56	0.000385	0.0118
GI_37541022-S	ATG2A	18	0.101	0.00331	3.55	0.000395	0.0121
GI_4755141-S	INPPL1	18	0.101	0.00263	3.55	0.000397	0.0121
GI_42544225-S	VPS18	18	0.101	0.00308	3.55	0.000402	0.0123
GI_11141876-S	SIGIRR	18	0.100	0.00312	3.54	0.000421	0.0127
GI_39930540-S	C9orf142	18	0.100	0.00299	3.52	0.000447	0.0133
GI_4507996-S	ZBTB17	18	0.100	0.00264	3.51	0.000462	0.0137
GI_5901987-S	WDR45	18	0.100	0.00193	3.50	0.000477	0.0141
GI_8670549-S	CYTH4	18	0.0993	0.00236	3.50	0.000484	0.0142
GI_29725606-I	UNC45A	18	0.0988	0.00218	3.48	0.000524	0.0152
GI_7019332-S	NRBP1	18	0.0983	0.00224	3.46	0.000556	0.0160
GI_33286441-S	BCORL1	18	0.0979	0.00322	3.45	0.000569	0.0163
GI_5921998-A	DYRK1B	18	0.0977	0.00294	3.45	0.000586	0.0166
GI_7382489-S	DGAT1	18	0.0979	0.00236	3.45	0.000587	0.0166
GI_20336278-A	SLC26A6	18	0.0968	0.00233	3.41	0.000673	0.0185
GI_31543298-S	PNPLA6	18	0.0965	0.00296	3.41	0.000682	0.0187
GI_29743268-S	TBC1D25	18	0.0965	0.00221	3.40	0.000704	0.0193
GI_4557478-A	CLK3	18	0.0961	0.00207	3.38	0.000744	0.0200
GI_4826947-S	PRKX	18	0.0962	0.00173	3.37	0.000763	0.0204
GI_34147725-A	SHC1	18	0.0969	0.00119	3.37	0.000778	0.0208
GI_14917110-S	AP4M1	18	0.0955	0.00227	3.36	0.000800	0.0213
GI_4885270-A	---	18	0.0947	0.00316	3.34	0.000859	0.0224
GI_45505128-S	TAOK2	18	0.0947	0.00272	3.34	0.000867	0.0226
GI_31543177-S	PRR14	18	0.0931	0.00174	3.27	0.001107	0.0274
GI_38327637-S	CREB3	18	0.0927	0.00214	3.26	0.001141	0.0280
GI_17017983-S	CDK9	18	0.0924	0.00200	3.25	0.001184	0.0288
GI_20149644-S	SARS2	18	-0.0918	-0.00216	-3.23	0.001269	0.0303
GI_31541963-S	ASL	18	0.0914	0.00252	3.22	0.001306	0.0310
GI_13899252-S	UCK1	18	0.0916	0.00173	3.22	0.001334	0.0315
GI_24432005-S	RHBDF2	18	0.0913	0.00196	3.21	0.001358	0.0319
GI_17865806-S	VPS4A	18	0.0906	0.00164	3.18	0.001515	0.0348
GI_20544143-A	CSNK1D	18	0.0904	0.00169	3.17	0.001555	0.0356
Hs.149165-S	---	18	0.0897	0.00287	3.16	0.001596	0.0364
GI_42476168-S	PAF1	18	0.0892	0.00259	3.15	0.001698	0.0382
GI_38146093-S	MED16	18	0.0891	0.00325	3.14	0.001714	0.0385
GI_13375689-S	CORO7	18	0.0893	0.00211	3.14	0.001727	0.0387
GI_4759179-A	STK19	18	0.0891	0.00187	3.13	0.001792	0.0397
GI_39930532-S	PPM1J	18	-0.0886	-0.00281	-3.12	0.001832	0.0404
GI_37547065-S	CC2D1B	18	0.0874	0.00294	3.08	0.002103	0.0450
GI_13994299-S	TMEM120A	18	0.0873	0.00235	3.08	0.002149	0.0457
GI_14141169-S	MTA2	18	0.0877	0.00162	3.08	0.002148	0.0457
GI_13129143-S	NDUFAF5	18	0.0870	0.00332	3.07	0.002198	0.0466
GI_38045930-S	RNF122	18	0.0866	0.00281	3.05	0.002316	0.0485
GI_33519471-S	NDUFB7	18	0.0872	0.00139	3.05	0.002328	0.0486
GI_31542869-S	GZMH	19	0.345	0.0223	12.9	6.49E-36	3.84E-32
GI_21687176-S	RASGEF1A	19	0.284	0.00877	10.4	2.08E-24	3.40E-21
GI_29729547-S	---	19	0.269	0.0107	9.80	6.57E-22	7.66E-19
GI_22538469-S	EOMES	19	0.260	0.0113	9.48	1.26E-20	1.19E-17
GI_40788019-S	C1orf21	19	0.252	0.00960	9.14	2.55E-19	2.11E-16
GI_29336036-S	PDZD4	19	0.242	0.0117	8.79	5.09E-18	3.44E-15
GI_30149753-S	LGR6	19	0.238	0.0102	8.63	1.87E-17	1.19E-14
GI_41327713-S	APMAP	19	0.239	0.00554	8.62	2.04E-17	1.28E-14
GI_41584199-S	GPR56	19	0.232	0.0129	8.40	1.18E-16	6.79E-14
GI_4758679-S	LLGL2	19	0.228	0.00932	8.24	4.18E-16	2.20E-13
GI_34147563-S	SLC1A7	19	0.221	0.0190	7.99	3.04E-15	1.42E-12
GI_33946290-S	LPCAT1	19	0.222	0.00543	7.99	3.01E-15	1.42E-12
GI_8923627-S	TTC38	19	0.216	0.00739	7.77	1.60E-14	6.29E-12
GI_20127481-S	NKG7	19	0.212	0.0106	7.65	4.15E-14	1.50E-11
GI_7108345-I	GNLY	19	0.208	0.0102	7.46	1.60E-13	5.40E-11
GI_24475870-S	GPR114	19	0.207	0.00616	7.44	1.94E-13	6.37E-11
GI_38788121-S	PRSS23	19	0.206	0.0152	7.41	2.35E-13	7.57E-11

GI_20302138-S	CST7	19	0.205	0.0110	7.38	2.90E-13	9.14E-11
GI_45446739-S	ABCA2	19	0.200	0.00927	7.18	1.20E-12	3.34E-10
GI_27484425-S	SYTL3	19	0.200	0.00642	7.16	1.41E-12	3.83E-10
GI_15451788-S	PDGFRB	19	0.199	0.0196	7.15	1.43E-12	3.85E-10
GI_31543022-S	FGFBP2	19	0.199	0.0113	7.14	1.59E-12	4.18E-10
GI_15718673-S	ADRB2	19	0.195	0.00763	6.98	4.79E-12	1.16E-09
GI_18079322-S	GAB3	19	0.192	0.00606	6.87	1.01E-11	2.32E-09
GI_39930402-S	LGR6	19	0.191	0.0104	6.86	1.11E-11	2.50E-09
GI_32698959-S	LINC00469	19	0.188	0.0119	6.74	2.39E-11	5.17E-09
GI_16506819-S	COLGALT2	19	0.187	0.00944	6.70	3.19E-11	6.73E-09
GI_32307116-A	PPP2R2B	19	0.184	0.0122	6.59	6.47E-11	1.24E-08
GI_4507470-S	TGFBR3	19	0.184	0.00887	6.57	7.48E-11	1.42E-08
GI_22035608-A	OSBPL5	19	0.175	0.00811	6.25	5.64E-10	8.80E-08
GI_42656914-S	DTHD1	19	0.173	0.00999	6.19	7.96E-10	1.21E-07
GI_24429585-S	---	19	0.173	0.00809	6.18	8.72E-10	1.30E-07
GI_7108343-A	GNLY	19	0.172	0.0102	6.13	1.19E-09	1.74E-07
GI_22748812-S	FCRLB	19	0.170	0.00538	6.05	1.96E-09	2.81E-07
GI_38372914-S	EVA1C	19	0.168	0.00709	6.00	2.57E-09	3.57E-07
GI_38505192-S	PTGDS	19	0.167	0.0108	5.95	3.40E-09	4.55E-07
GI_37542486-S	TSEN54	19	0.164	0.00470	5.84	6.50E-09	8.19E-07
GI_38016906-A	STOM	19	0.162	0.00564	5.76	1.09E-08	1.32E-06
GI_25777679-A	RASSF1	19	0.160	0.00467	5.69	1.55E-08	1.82E-06
GI_18141314-S	S1PR5	19	0.154	0.00881	5.47	5.36E-08	5.64E-06
GI_31543620-S	SH2D2A	19	0.151	0.00699	5.38	9.07E-08	9.09E-06
GI_37539470-S	DTHD1	19	0.151	0.00828	5.36	9.68E-08	9.59E-06
GI_18860909-A	SNTB2	19	0.150	0.00425	5.32	1.24E-07	1.19E-05
GI_38202210-S	GNPTAB	19	0.145	0.00322	5.12	3.54E-07	3.04E-05
GI_7108343-I	GNLY	19	0.143	0.00853	5.10	3.98E-07	3.38E-05
GI_15451920-A	PDGFD	19	0.140	0.00664	4.98	7.35E-07	5.82E-05
GI_6996012-S	GZMA	19	0.138	0.00739	4.91	1.03E-06	7.80E-05
GI_4757917-S	RUNX3	19	0.137	0.00579	4.84	1.43E-06	0.000103
GI_21955169-I	SSBP3	19	0.137	0.00352	4.84	1.43E-06	0.000103
GI_32483414-S	GZMB	19	0.136	0.00775	4.84	1.46E-06	0.000105
GI_19923571-S	SLAMF7	19	0.136	0.00528	4.83	1.51E-06	0.000108
GI_19913391-A	RGS3	19	0.136	0.00367	4.82	1.64E-06	0.000115
GI_7706528-S	CD244	19	0.135	0.00370	4.80	1.81E-06	0.000126
GI_7669497-I	KLRD1	19	0.134	0.00643	4.74	2.36E-06	0.000159
GI_14249307-S	PYROXD2	19	0.133	0.00428	4.73	2.51E-06	0.000169
GI_4759139-S	SLC9A3R1	19	0.129	0.00570	4.57	5.44E-06	0.000334
GI_4758955-S	BZRAP1	19	0.128	0.00479	4.53	6.59E-06	0.000399
GI_23110963-S	CTSW	19	0.126	0.00596	4.47	8.47E-06	0.000503
GI_21618332-S	STAT4	19	0.124	0.00351	4.39	1.25E-05	0.000706
GI_21450843-A	MATK	19	0.124	0.00466	4.38	1.30E-05	0.000731
GI_42734326-S	RAP1GAP2	19	0.122	0.00449	4.33	1.63E-05	0.000887
GI_31341948-S	PRSS30P	19	0.122	0.00673	4.32	1.67E-05	0.000899
Hs.147489-S	---	19	0.122	0.00699	4.31	1.78E-05	0.000951
GI_4504878-S	KLRB1	19	-0.117	-0.00649	-4.13	3.90E-05	0.00180
GI_40549458-S	YPEL1	19	0.115	0.00511	4.07	5.01E-05	0.00223
GI_17402874-I	COL6A2	19	0.114	0.00526	4.05	5.43E-05	0.00238
GI_32130537-S	CD7	19	-0.114	-0.00506	-4.05	5.46E-05	0.00239
GI_37551990-S	SIGLEC17P	19	0.113	0.00496	3.99	7.12E-05	0.00299
GI_40254807-S	PRF1	19	0.112	0.00646	3.96	7.87E-05	0.00325
GI_34147599-S	CD99	19	0.110	0.00297	3.87	0.000115	0.00442
GI_11968153-S	KIR2DL5A	19	0.105	0.00735	3.73	0.000202	0.00701
GI_31343330-S	RAB37	19	0.104	0.00256	3.68	0.000245	0.00828
GI_5902003-S	MMP23B	19	0.104	0.00410	3.66	0.000258	0.00860
GI_18604602-S	NCAM1	19	0.102	0.00462	3.59	0.000346	0.0109
GI_7019440-S	KIR3DL1	19	0.101	0.00635	3.56	0.000390	0.0120
GI_28466996-S	DLG5	19	0.0994	0.00535	3.51	0.000459	0.0136
GI_7657272-S	KIR2DL2	19	0.0979	0.00615	3.46	0.000553	0.0159
GI_29171679-S	CXCR1	19	0.0974	0.00977	3.45	0.000587	0.0166
GI_7705573-S	KLRF1	19	0.0965	0.00491	3.41	0.000671	0.0185
GI_21264586-A	HOPX	19	0.0936	0.00579	3.31	0.000965	0.0246
GI_38505191-S	PTGDR	19	0.0904	0.00585	3.19	0.001435	0.0334
GI_27477087-S	IL18RAP	19	-0.0879	-0.00456	-3.11	0.001941	0.0423
GI_23592225-S	KIR3DL3	19	0.0875	0.0174	3.09	0.002030	0.0439
GI_4506344-S	PXN	19	0.0875	0.00201	3.08	0.002139	0.0456
GI_6031164-S	F2R	20	0.209	0.0113	7.50	1.18E-13	4.12E-11
GI_19923320-S	KIFC3	20	0.185	0.0284	6.62	5.35E-11	1.07E-08
GI_17149840-A	FKBP1B	20	0.166	0.0148	5.92	4.23E-09	5.59E-07
GI_13376724-S	CCDC92	20	0.143	0.00445	5.08	4.41E-07	3.67E-05
GI_10938005-S	SLC10A3	20	0.138	0.00613	4.91	1.01E-06	7.72E-05
GI_21464102-S	YWHAH	20	0.133	0.00409	4.72	2.57E-06	0.000172
GI_34328914-A	CD151	20	0.127	0.00525	4.49	7.87E-06	0.000470
GI_13027798-S	MMP1	20	0.123	0.0203	4.36	1.40E-05	0.000775
GI_5031940-S	NFIB	20	0.121	0.00873	4.31	1.79E-05	0.000951
GI_45505152-S	NCKAP1	20	0.121	0.00630	4.29	1.89E-05	0.00100
GI_9955969-A	ABCC3	20	0.120	0.00767	4.26	2.24E-05	0.00114
GI_11761623-I	ST7	20	0.119	0.00394	4.20	2.85E-05	0.00140
GI_41152089-S	PDZK1IP1	20	0.117	0.0116	4.16	3.34E-05	0.00159
GI_41393552-S	MGLL	20	0.117	0.00602	4.14	3.76E-05	0.00175
GI_21237762-S	CD9	20	0.117	0.00625	4.13	3.81E-05	0.00177
GI_6552326-A	CYB5R3	20	0.116	0.00396	4.10	4.36E-05	0.00198
GI_15451935-I	CDC14B	20	0.114	0.00891	4.05	5.53E-05	0.00242
GI_12025669-S	ACTN1	20	-0.113	-0.00518	-4.01	6.51E-05	0.00279
GI_16753232-S	TLN1	20	0.113	0.00359	4.00	6.63E-05	0.00282
GI_20070329-S	ZFYVE21	20	0.112	0.00386	3.97	7.67E-05	0.00318

GI_41872613-S	CXCL5	20	0.112	0.00915	3.95	8.13E-05	0.00333
GI_4506116-S	PROS1	20	0.107	0.0103	3.80	0.000151	0.00552
GI_4759181-S	STX1A	20	0.108	0.00446	3.80	0.000151	0.00552
GI_31342330-S	TREML1	20	0.105	0.00606	3.73	0.000199	0.00694
GI_19923444-A	ATL1	20	0.105	0.00631	3.71	0.000213	0.00734
GI_6006009-S	ITGA2B	20	0.104	0.00807	3.69	0.000232	0.00790
GI_20336333-A	BCL2L1	20	0.104	0.00376	3.67	0.000254	0.00852
GI_31563525-S	SLC24A3	20	0.101	0.0158	3.58	0.000359	0.0112
GI_24430131-S	WBP2	20	0.101	0.00375	3.56	0.000379	0.0117
GI_38570041-S	CLDN5	20	0.101	0.00572	3.56	0.000391	0.0120
GI_21237780-S	WASF3	20	0.0979	0.00943	3.46	0.000552	0.0159
GI_29745993-S	ENDOD1	20	0.0976	0.00437	3.45	0.000581	0.0165
GI_45269134-S	GP1BA	20	0.0973	0.00912	3.44	0.000595	0.0168
GI_6031196-S	SELP	20	0.0968	0.00742	3.42	0.000643	0.0179
GI_4557676-S	ITGB3	20	0.0967	0.00771	3.42	0.000646	0.0180
GI_8922813-S	TMEM140	20	0.0953	0.00402	3.37	0.000785	0.0209
GI_42657580-S	CASC15	20	-0.0935	-0.0129	-3.31	0.000969	0.0246
GI_31543081-S	MARCH2	20	0.0929	0.00311	3.28	0.001080	0.0268
GI_34147679-S	TGFB1I1	20	0.0924	0.0152	3.27	0.001117	0.0275
GI_24432051-S	SPOCD1	20	0.0919	0.0154	3.25	0.001192	0.0289
GI_11184225-S	RGS10	20	-0.0917	-0.00236	-3.23	0.001278	0.0305
GI_4507356-S	TAGLN2	20	0.0912	0.00452	3.22	0.001310	0.0310
GI_31563523-A	MYL9	20	0.0888	0.0114	3.14	0.001734	0.0388
GI_11761623-A	ST7	20	0.0889	0.00362	3.14	0.001734	0.0388
GI_14456712-S	HBQ1	20	0.0881	0.00825	3.12	0.001881	0.0413
GI_13775195-S	TSPAN9	20	0.0863	0.00605	3.05	0.002355	0.0490

List of 2,285 transcript identified in the SAFHS dataset for which their expression is significantly correlated to chronological age (moderated t-test: adjusted $p \leq 0.05$). 1,143 transcripts were positively correlated to increase in age (red) and 1,142 transcripts negatively correlated to age (blue). For each transcript, its probeset identifier, its HUGO gene symbol and its association with age are given. The membership of the transcript in the twenty modules (M1 to M20) forming the BioAge is indicated in the column ‘Module’.

Supplementary Table 3b. Pathway enrichment analysis in the twenty BioAge modules

Legend
- Genes negatively correlated to age (blue)
- Genes positively correlated to age (red)

Module 1

Pathway	Ratio	nominal p-value	adjusted p-value	Intersect
B-cell Signaling				
B_CELL_DEVELOPMENT	0.125	4.37E-06	0.00162	HLA-DOB,CD79A,CD19,CD79B
ALTERED_T_CELL_AND_B_CELL_SIGNALING_IN_RHEUMATOID_ARTHRITIS	0.0595	9.77E-06	0.00182	TNFRSF17,HLA-DOB,TNFRSF13B,CD79A,CD79B
PRIMARY_IMMUNODEFICIENCY_SIGNALING	0.0851	2.09E-05	0.00259	IGLL5,TNFRSF13B,CD79A,CD19
PI3K_SIGNALING_IN_B_LYMPHOCYTES	0.0296	0.00126	0.117	CD79A,BLK,CD19,CD79B
B_CELL_RECEPTOR_SIGNALING	0.0274	0.00169	0.125	CD22,CD79A,CD19,CD79B
ROLE_OF_NFAT_IN_REGULATION_OF_THE_IMMUNE_RESPONSE	0.0209	0.00449	0.278	HLA-DOB,CD79A,GNG7,CD79B
APRIL_MEDIATED_SIGNALING	0.0500	0.00910	0.354	TNFRSF17,TNFRSF13B
P70S6K_SIGNALING	0.0244	0.00955	0.354	CD79A,CD19,CD79B
SYSTEMIC_LUPUS_ERYTHEMATOSUS_SIGNALING	0.0244	0.00955	0.354	CD22,CD79A,CD79B
B_CELL_ACTIVATING_FACTOR_SIGNALING	0.0476	0.0100	0.354	TNFRSF17,TNFRSF13B
FCGAMMARIIB_SIGNALING_IN_B_LYMPHOCYTES	0.0253	0.0330	0.683	CD79A,CD79B
COMMUNICATION_BETWEEN_INNATE_AND_ADAPTIVE_IMMUNE_CELLS	0.0235	0.0378	0.725	TNFRSF17,TNFRSF13B
Cell Signal Transduction by Trasncription factors				
THYROID_CANCER_SIGNALING	0.0465	0.0105	0.354	TCF4,TCF3
OVARIAN_CANCER_SIGNALING	0.0201	0.0160	0.497	TCF4,E2F5,TCF3
HUMAN_EMBRYONIC_STEM_CELL_PLURIPOTENCY	0.0184	0.0204	0.577	TCF4,GNG7,TCF3
WNT_BETA-CATENIN_SIGNALING	0.0180	0.0217	0.577	TCF4,TLE1,TCF3
BASAL_CELL_CARCINOMA_SIGNALING	0.0286	0.0264	0.655	TCF4,TCF3
ROLE_OF_WNT_GSK-3BETA__SIGNALING_IN_THE_PATHOGENESIS_OF_INFLUENZA	0.0267	0.0300	0.683	TCF4,TCF3
ACUTE_MYELOID_LEUKEMIA_SIGNALING	0.0253	0.0330	0.683	TCF4,TCF3
FACTORS_PROMOTING_CARDIOGENESIS_IN_VERTEBRATES	0.0227	0.0402	0.725	TCF4,TCF3
MOLECULAR_MECHANISMS_OF_CANCER	0.0109	0.0409	0.725	TCF4,E2F5,GNG7,TCF3

Module 2

Pathway	Ratio	nominal p-value	adjusted p-value	Intersect
Phosphoric Ester Hydrolase				
GRANZYME_A_SIGNALING	0.118	0.00693	0.860	APEX1,SET
AMINOSUGARS_METABOLISM	0.0441	0.0140	1.00	APEX1,PDE3B,PDE7A
RELAXIN_SIGNALING	0.0292	0.0186	1.00	APEX1,PDE3B,RLN2,PDE7A
Valine Catabolic Pathway				
VALINE_LEUCINE_AND_ISOLEUCINE_DEGRADATION	0.0667	0.000976	0.182	MCEE,HADH,BCKDHB,ALDH6A1
INOSITOL_METABOLISM	0.200	0.0369	1.00	ALDH6A1
Others				
PURINE_METABOLISM	0.0307	0.000581	0.182	PAICS,PPAT,PDE3B,PDE7A,KATNA1,BCKDHB,ATP6V1G1,CLPX
AMINOPHOSPHONATE_METABOLISM	0.0909	0.0115	1.00	BCKDHB,PIGN
GLUTAMATE_METABOLISM	0.0571	0.0279	1.00	ALDH5A1,PPAT

Module 3

Pathway	Ratio	nominal p-value	adjusted p-value	Intersect
MYC-LEF1 Signaling				
ROLE_OF_MACROPHAGES_FIBROBLASTS_AND_ENDOTHELIAL_CELLS_IN_RHEUMATOID_ARTHRITIS	0.0243	0.00273	0.974	LEF1,IL16,MYC,TRAF5,CAMK4,LTB,PRKCA
PROTEIN_KINASE_A_SIGNALING	0.0195	0.0158	0.974	PDE9A,FLNB,LEF1,PDE6B,CAMK4,PRKCA
GLIOBLASTOMA_MULTIFORME_SIGNALING	0.0268	0.0173	0.974	LEF1,IGF1R,MYC,FOXO1
SMALL_CELL_LUNG_CANCER_SIGNALING	0.0319	0.0250	0.974	MYC,TRAF5,FHIT
THYROID_CANCER_SIGNALING	0.0465	0.0337	0.974	LEF1,MYC
GLIOMA_SIGNALING	0.0275	0.0366	0.974	IGF1R,CAMK4,PRKCA
ENDOMETRIAL_CANCER_SIGNALING	0.0385	0.0477	0.974	LEF1,MYC
Others				
RHOA_SIGNALING	0.0370	0.00574	0.974	IGF1R,RAPGEF6,SEPT6,EPHA1
ALTERED_T_CELL_AND_B_CELL_SIGNALING_IN_RHEUMATOID_ARTHRITIS	0.0357	0.0186	0.974	IL23A,CD28,LTB
T_CELL_RECEPTOR_SIGNALING	0.0294	0.0309	0.974	TXK,CAMK4,CD28
CROSSTALK_BETWEEN_DENDRITIC_CELLS_AND_NATURAL_KILLER_CELLS	0.0286	0.0332	0.974	CCR7,CD28,LTB

Module 4

Pathway	Ratio	nominal p-value	adjusted p-value	Intersect
Cellular Metabolism				
BETA-ALANINE_METABOLISM	0.0435	0.00867	1.00	HIBCH,ACADM
PROPANOATE_METABOLISM	0.0357	0.0127	1.00	HIBCH,ACADM
VALINE_LEUCINE_AND_ISOLEUCINE_DEGRADATION	0.0333	0.0145	1.00	HIBCH,ACADM
FATTY_ACID_METABOLISM	0.0194	0.0397	1.00	ACSL5,ACADM
Cell Growth Regulation				
EIF2_SIGNALING	0.0238	0.0273	1.00	ATM,EIF2S2
VEGF_SIGNALING	0.0235	0.0279	1.00	ATM,EIF2S2
TR_RXR_ACTIVATION	0.0233	0.0285	1.00	ATM,TBL1XR1
P53_SIGNALING	0.0215	0.0329	1.00	ATM,GNL3
DNA_DOUBLE-STRAND_BREAK_REPAIR_BY_HOMOLOGOUS_RECOMBINATION	0.0667	0.0456	1.00	ATM
DNA_DOUBLE-STRAND_BREAK_REPAIR_BY_NON-HOMOLOGOUS_END_JOINING	0.0667	0.0456	1.00	ATM
Others				
CLEAVAGE_AND_POLYADENYLATION_OF_PRE-MRNA	0.167	0.000588	0.219	WDR33,CPSF3
AMINOACYL-TRNA_BIOSYNTHESIS	0.0571	0.00509	0.946	IARS,EPRS
VALINE_LEUCINE_AND_ISOLEUCINE_BIOSYNTHESIS	0.0909	0.0337	1.00	IARS

Module 5

Pathway	Ratio	nominal p-value	adjusted p-value	Intersect
P53_SIGNALING	0.0323	0.00322	1.00	CDK4,PLAGL1,SIRT1

Module 6

Pathway	Ratio	nominal p-value	adjusted p-value	Intersect
T-cell Receptor Signaling				
TWEAK_SIGNALING	0.0833	0.00103	0.203	TRADD,TNFRSF25,TRAF1
ROLE_OF_MACROPHAGES_FIBROBLASTS_AND_ENDOTHELIAL_CELLS_IN_RHEUMATOID_ARTHRITIS	0.0208	0.00486	0.465	TCF7,PLCD1,TRADD,TRAF1,PIK3CD,PLCG1
MACROPINOCYTOSIS_SIGNALING	0.0435	0.00669	0.465	ITGB7,PIK3CD,PLCG1
LEPTIN_SIGNALING_IN_OBESITY	0.0417	0.00753	0.465	PLCD1,PIK3CD,PLCG1
PROLACTIN_SIGNALING	0.0411	0.00783	0.465	TCF7,PIK3CD,PLCG1
HER-2_SIGNALING_IN_BREAST_CANCER	0.0395	0.00875	0.465	ITGB7,PIK3CD,PLCG1
VIRUS_ENTRY_VIA_ENDOCYTIC_PATHWAYS	0.0337	0.0134	0.536	ITGB7,PIK3CD,PLCG1
CTLA4_SIGNALING_IN_CYTOTOXIC_T_LYMPHOCYTES	0.0319	0.0156	0.536	PIK3CD,PLCG1,ZAP70
HMGB1_SIGNALING	0.0316	0.0160	0.536	KAT2A,PIK3CD,AGER
NEUROPATHIC_PAIN_SIGNALING_IN_DORSAL_HORN_NEURONS	0.0303	0.0179	0.536	PLCD1,PIK3CD,PLCG1
T_CELL_RECEPTOR_SIGNALING	0.0294	0.0193	0.536	PIK3CD,PLCG1,ZAP70
SPHINGOSINE-1-PHOSPHATE_SIGNALING	0.0280	0.0219	0.536	PLCD1,PIK3CD,PLCG1
FAK_SIGNALING	0.0275	0.0230	0.536	ITGB7,PIK3CD,PLCG1
ICOS-ICOSL_SIGNALING_IN_T_HELPER_CELLS	0.0270	0.0242	0.536	PIK3CD,PLCG1,ZAP70
NATURAL_KILLER_CELL_SIGNALING	0.0270	0.0242	0.536	PIK3CD,PLCG1,ZAP70
14-3-3-MEDIATED_SIGNALING	0.0263	0.0259	0.536	PLCD1,PIK3CD,PLCG1
EGF_SIGNALING	0.0435	0.0275	0.536	PIK3CD,PLCG1
P2Y_PURIGENIC_RECEPTOR_SIGNALING_PATHWAY	0.0252	0.0289	0.536	PLCD1,PIK3CD,PLCG1
CD28_SIGNALING_IN_T_HELPER_CELLS	0.0248	0.0302	0.536	PIK3CD,PLCG1,ZAP70
P70S6K_SIGNALING	0.0244	0.0315	0.536	PLCD1,PIK3CD,PLCG1
LYMPHOTOXIN_BETA_RECEPTOR_SIGNALING	0.0364	0.0383	0.536	TRAF1,PIK3CD
THROMBOPOIETIN_SIGNALING	0.0364	0.0383	0.536	PIK3CD,PLCG1
INOSITOL_PHOSPHATE_METABOLISM	0.0226	0.0384	0.536	PLCD1,PIK3CD,PLCG1
PI3K_SIGNALING_IN_B_LYMPHOCYTES	0.0222	0.0399	0.536	PLCD1,PIK3CD,PLCG1
DEATH_RECEPTOR_SIGNALING	0.0323	0.0476	0.536	TRADD,TNFRSF25
INDUCTION_OF_APOPTOSIS_BY_HIV1	0.0317	0.0490	0.536	TRADD,TRAF1
Others				
ASSEMBLY_OF_RNA_POLYMERASE_I_COMPLEX	0.222	0.00109	0.203	POLR1C,TAF1C
PYRIMIDINE_METABOLISM	0.0222	0.0399	0.536	POLR1C,POLD2,POLR3C

Module 7

Pathway	Ratio	nominal p-value	adjusted p-value	Intersect
Protein Tyrosine Phosphatase				
RIBOFLAVIN_METABOLISM	0.118	0.000403	0.0842	DUSP18,PTPRJ
FRUCTOSE_AND_MANNOSE_METABOLISM	0.0465	0.00261	0.323	DUSP18,PTPRJ
AMINOSUGARS_METABOLISM	0.0294	0.00642	0.597	DUSP18,PTPRJ
SPHINGOLIPID_METABOLISM	0.0256	0.00838	0.623	DUSP18,PTPRJ
NICOTINATE_AND_NICOTINAMIDE_METABOLISM	0.0208	0.0125	0.775	DUSP18,PTPRJ
Others				
CYANOAMINO_ACID_METABOLISM	0.111	0.000453	0.0842	FAAH,ASRGL1

Module 8

Pathway	Ratio	nominal p-value	adjusted p-value	Intersect
Antiviral Responses				
ROLE_OF_RIG1-LIKE_RECEPTORS_IN_ANTIVIRAL_INNATE_IMMUNITY	0.0227	0.0318	1.00	MAVS
ACTIVATION_OF_IRF_BY_CYTOSOLIC_PATTERN_RECOGNITION_RECEPTORS	0.0159	0.0453	1.00	MAVS

Module 9

Pathway	Ratio	nominal p-value	adjusted p-value	Intersect
Fatty Acid Biosynthesis				
FATTY_ACID_BIOSYNTHESIS	0.200	0.000954	0.141	ACACA,FASN
LXR_RXR_ACTIVATION	0.0441	0.00389	0.361	NFKB1,ACACA,FASN
AMPK_SIGNALING	0.0247	0.00664	0.458	PFKP,ACACA,FASN,PRKAB1
TR_RXR_ACTIVATION	0.0349	0.00750	0.458	PFKP,ACACA,FASN
PENTOSE_PHOSPHATE_PATHWAY	0.0667	0.00870	0.458	PFKP,ALDOC
FRUCTOSE_AND_MANNOSE_METABOLISM	0.0465	0.0174	0.719	PFKP,ALDOC
INOSITOL_METABOLISM	0.200	0.0235	0.875	ALDOC
PYRUVATE_METABOLISM	0.0328	0.0334	1.00	HAGH,ACACA
PPARALPHA_RXRALPHA_ACTIVATION	0.0189	0.0383	1.00	NFKB1,FASN,PRKAB1
Others				
PYRIMIDINE_METABOLISM	0.0370	0.000367	0.137	NME1,PUS1,POLR1E,CTPS2,POLE3
PURINE_METABOLISM	0.0230	0.00114	0.141	NME1,IMPDH2,PFAS,ATP5G1,POLR1E,POLE3
UREA_CYCLE_AND_METABOLISM_OF_AMINO_GROUPS	0.0625	0.00986	0.458	SRM,PYCRL
POLYAMINE_REGULATION_IN_COLON_CANCER	0.0333	0.0324	1.00	PSMB9,PSMD8
ARGININE_AND_PROLINE_METABOLISM	0.0282	0.0441	1.00	SRM,PYCRL

Module 10

Pathway	Ratio	nominal p-value	adjusted p-value	Intersect
Lysine-Tryptophan Metabolism				
TRYPTOPHAN_METABOLISM	0.0273	0.0180	1.00	GCDH,ALDH7A1,WARS2
LYSINE_DEGRADATION	0.0339	0.0361	1.00	GCDH,ALDH7A1
Others				
MITOTIC_ROLES_OF_POLO-LIKE_KINASE	0.0517	0.00306	1.00	PPM1J,KIF23,CDC20
INTERFERON_SIGNALING	0.0606	0.0121	1.00	MX1,IFIT1
LXR_RXR_ACTIVATION	0.0294	0.0467	1.00	SCD,SREBF1

Module 11

Pathway	Ratio	nominal p-value	adjusted p-value	Intersect
Glycophingolipid Biosynthesis				
GLYCOSPHINGOLIPID_BIOSYNTHESIS_-_GLOBOSERIES	0.0952	0.0178	1.00	B3GALNT1,ST8SIA1
GLYCOSPHINGOLIPID_BIOSYNTHESIS_-_GANGLIOSERIES	0.0833	0.0229	1.00	ST8SIA1,DBT
GLYCINE_SERINE_AND_THREONINE_METABOLISM	0.0448	0.0279	1.00	PHGDH,GLDC,DBT
Others				
PXR_RXR_ACTIVATION	0.0441	0.0290	1.00	AKT3,GSTM2,ABCB9

Module 12

Pathway	Ratio	nominal p-value	adjusted p-value	Intersect
Actin-Based Cell Motility				
RHOA_SIGNALING	0.0370	0.0124	1.00	ACTA2,ACTA1,ARPC2,TTN
CAVEOLAR-MEDIATED_ENDOCYTOSIS_SIGNALING	0.0417	0.0221	1.00	ACTA2,ACTA1,HLA-B
REGULATION_OF_ACTIN-BASED_MOTILITY_BY_RHO	0.0361	0.0320	1.00	ACTA2,ACTA1,ARPC2
VEGF_SIGNALING	0.0353	0.0340	1.00	ACTA2,ACTA1,FLT4
VIRUS_ENTRY_VIA_ENDOCYTIC_PATHWAYS	0.0337	0.0382	1.00	ACTA2,ACTA1,HLA-B
FCGAMMA_RECEPTOR-MEDIATED_PHAGOCYTOSIS_IN_MACROPHAGES_AND_MONOCYTES	0.0326	0.0415	1.00	ACTA2,ACTA1,ARPC2
MECHANISMS_OF_VIRAL_EXIT_FROM_HOST_CELLS	0.0500	0.0440	1.00	ACTA2,ACTA1

Others				
C21-STEROID_HORMONE_METABOLISM	0.105	0.0108	1.00	HSD3B7,EBP
COMPLEMENT_SYSTEM	0.0571	0.0345	1.00	CFH,C1QC
NITROGEN_METABOLISM	0.0571	0.0345	1.00	LOXL1,TGM2

Module 13

Pathway	Ratio	nominal p-value	adjusted p-value	Intersect
RHOA_SIGNALING	0.0278	0.0435	1.00	ARPC5L,PIP4K2C,SEPT8
PRIMARY_IMMUNODEFICIENCY_SIGNALING	0.0426	0.0458	1.00	ADA,TAP1

Module 14

Pathway	Ratio	nominal p-value	adjusted p-value	Intersect
Cell Motility				
REGULATION_OF_EIF4_AND_P70S6K_SIGNALING	0.0388	0.00316	0.637	MAPK1,ITGB1,EIF4EBP2,EIF4EBP3,ITGB2
CDC42_SIGNALING	0.0316	0.00747	0.637	MAPK1,ITGB1,HLA-E,MYL6,ITGB2
ACTIN_CYTOSKELETON_SIGNALING	0.0262	0.00836	0.637	MAPK1,ITGB1,SSH1,TMSB10,MYL6,ITGB2
PAK_SIGNALING	0.0364	0.0104	0.637	MAPK1,ITGB1,MYL6,ITGB2
ILK_SIGNALING	0.0276	0.0130	0.637	MAPK1,ITGB1,TMSB10,MYL6,ITGB2
PHOSPHOLIPASE_C_SIGNALING	0.0233	0.0145	0.637	MAPK1,ITGB1,GNG2,MYL6,AHNAK,ITGB2
LEUKOCYTE_EXTRAVASATION_SIGNALING	0.0267	0.0148	0.637	MAPK1,ITGB1,CYBA,MYL6,ITGB2
AGRIN_INTERACTIONS_AT_NEUROMUSCULAR_JUNCTION	0.0448	0.0153	0.637	MAPK1,ITGB1,ITGB2
EPHRIN_RECEPTOR_SIGNALING	0.0258	0.0171	0.637	MAPK1,ITGB1,GNG2,ITGB2,SH2D3C
INTEGRIN_SIGNALING	0.0258	0.0171	0.637	RAP2A,MAPK1,TSPAN2,ITGB1,ITGB2
NF-KAPPAB_ACTIVATION_BY_VIRUSES	0.0400	0.0206	0.697	MAPK1,ITGB1,ITGB2
INHIBITION_OF_ANGIOGENESIS_BY_TSP1	0.0606	0.0273	0.846	MAPK1,CD47

Module 15

Pathway	Ratio	nominal p-value	adjusted p-value	Intersect
Integrin Signaling				
INTEGRIN_SIGNALING	0.0464	0.000373	0.102	ITGAL,PPP1CA,ACTN4,RAPGEF1,CAPNS1,AKT1,CAPN1,ARPC4,ITGA5
VIRUS_ENTRY_VIA_ENDOCYTIC_PATHWAYS	0.0674	0.000549	0.102	FLNA,ITGAL,AP2B1,CLTB,AP2M1,ITGA5
REGULATION_OF_ACTIN-BASED_MOTILITY_BY_RHO	0.0602	0.00269	0.333	PPP1CA,PIP4K2A,ARHGDIA,ARPC4,PFN1
CTLA4_SIGNALING_IN_CYTOTOXIC_T_LYMPHOCYTES	0.0532	0.00460	0.361	PPP2R5B,AKT1,AP2B1,CLTB,AP2M1
ACTIN_CYTOSKELETON_SIGNALING	0.0349	0.00485	0.361	ITGAL,PPP1CA,ACTN4,PIP4K2A,DIAPH1,ARPC4,PFN1,ITGA5
FAK_SIGNALING	0.0459	0.00857	0.459	ITGAL,CAPNS1,AKT1,CAPN1,ITGA5
CAVEOLAR-MEDIATED_ENDOCYTOSIS_SIGNALING	0.0556	0.00965	0.459	FLNA,ITGAL,FLOT1,ITGA5
ERK_MAPK_SIGNALING	0.0335	0.0105	0.459	ITGAL,PPP1CA,ELF4,RAPGEF1,PPP2R5B,ITGA5,SRF
NF-KAPPAB_ACTIVATION_BY_VIRUSES	0.0533	0.0111	0.459	ITGAL,AKT1,IKBKG,ITGA5
HUNTINGTONS_DISEASE_SIGNALING	0.0324	0.0124	0.462	CAPNS1,AKT1,YKT6,UBE2S,DCTN1,CTSD,CAPN1
PTEN_SIGNALING	0.0382	0.0180	0.569	ITGAL,AKT1,FOXO4,IKBKG,ITGA5
AMYLOID_PROCESSING	0.0600	0.0202	0.579	CAPNS1,AKT1,CAPN1
PI3K_AKT_SIGNALING	0.0360	0.0226	0.600	ITGAL,PPP2R5B,AKT1,IKBKG,ITGA5
EPHRIN_RECEPTOR_SIGNALING	0.0309	0.0250	0.620	ITGAL,RAPGEF1,AKT1,AXIN1,ARPC4,ITGA5
CLATHRIN-MEDIATED_ENDOCYTOSIS_SIGNALING	0.0318	0.0357	0.831	AP2B1,CLTB,AP2M1,ARPC4,ITGA5
HYPOXIA_SIGNALING_IN_THE_CARDIOVASCULAR_SYSTEM	0.0462	0.0399	0.857	AKT1,UBE2S,CDC34
ANGIOPOIETIN_SIGNALING	0.0448	0.0430	0.857	AKT1,IKBKG,TNIP1
Others				
CDK5_SIGNALING	0.0460	0.0183	0.569	PPP1CA,PPP2R5B,ADCY9,CAPN1
RAR_ACTIVATION	0.0301	0.0438	0.857	NCOA1,AKT1,ZBTB16,MAPKAPK2,ADCY9

Module 16

Pathway	Ratio	nominal p-value	adjusted p-value	Intersect
Inflammatory Responses				
ACUTE_PHASE_RESPONSE_SIGNALING	0.0545	4.73E-06	0.00176	C1QA,C1QB,HMOX1,C3,RRAS,TNFRSF1B,MYD88,CEBPB,MRAS
ROLE_OF_PATTERN_RECOGNITION_RECEPTORS_IN_RECOGNITION_OF_BACTERIA_AND_VIRUSES	0.0789	2.72E-05	0.00507	C1QA,C1QB,C3,MYD88,C3AR1,IRF7
COMPLEMENT_SYSTEM	0.114	0.000159	0.0197	C1QA,C1QB,C3,C3AR1
ONCOSTATIN_M_SIGNALING	0.0882	0.00244	0.227	MT2A,RRAS,MRAS
IL-6_SIGNALING	0.0471	0.00458	0.316	RRAS,TNFRSF1B,CEBPB,MRAS
CHOLECYSTOKININ_GASTRIN-MEDIATED_SIGNALING	0.0444	0.00562	0.316	RHOC,RRAS,MAPK7,MRAS
FCGAMMA_RECEPTOR-MEDIATED_PHAGOCYTOSIS_IN_MACROPHAGES_AND_MONOCYTES	0.0435	0.00607	0.316	HMOX1,ARPC1A,FGR,VASP
HMGB1_SIGNALING	0.0421	0.00680	0.316	RHOC,RRAS,TNFRSF1B,MRAS
ENDOTHELIN-1_SIGNALING	0.0305	0.00959	0.390	HMOX1,CASP5,RRAS,MAPK7,MRAS
GLIOMA_INVASIVENESS_SIGNALING	0.0526	0.0105	0.390	RHOC,RRAS,MRAS
ERK5_SIGNALING	0.0492	0.0126	0.408	RRAS,MAPK7,MRAS
ROLE_OF_JAK1_AND_JAK3_IN_GAMMAC_CYTOKINE_SIGNALING	0.0484	0.0132	0.408	IL15,RRAS,MRAS
IL-15_SIGNALING	0.0469	0.0144	0.411	IL15,RRAS,MRAS
IL-8_SIGNALING	0.0270	0.0156	0.414	RHOC,HMOX1,RRAS,CSTB,MRAS
INTEGRIN_SIGNALING	0.0258	0.0188	0.441	RHOC,RRAS,ARPC1A,MRAS,VASP
PROLACTIN_SIGNALING	0.0411	0.0204	0.441	RRAS,CEBPB,MRAS
IL-17_SIGNALING	0.0405	0.0211	0.441	RRAS,CEBPB,MRAS
LPS_IL-1_MEDIATED_INHIBITION_OF_RXR_FUNCTION	0.0245	0.0228	0.441	SCARB1,CHST7,TNFRSF1B,MYD88,SLC27A3
CERAMIDE_SIGNALING	0.0375	0.0259	0.441	RRAS,TNFRSF1B,MRAS
NRF2-MEDIATED_OXIDATIVE_STRESS_RESPONSE	0.0237	0.0260	0.441	SCARB1,HMOX1,RRAS,MAPK7,MRAS
ROLE_OF_MACROPHAGES_FIBROBLASTS_AND_ENDOTHELIAL_CELLS_IN_RHEUMATOID_ARTHRITIS	0.0208	0.0263	0.441	IL15,RRAS,TNFRSF1B,MYD88,CEBPB,MRAS
MTOR_SIGNALING	0.0280	0.0270	0.441	RHOC,HMOX1,RRAS,MRAS
PPAR_SIGNALING	0.0366	0.0276	0.441	RRAS,TNFRSF1B,MRAS
REGULATION_OF_ACTIN-BASED_MOTILITY_BY_RHO	0.0361	0.0285	0.441	RHOC,ARPC1A,GSN
NON-SMALL_CELL_LUNG_CANCER_SIGNALING	0.0353	0.0303	0.441	E2F2,RRAS,MRAS
GLIOBLASTOMA_MULTIFORME_SIGNALING	0.0268	0.0308	0.441	RHOC,E2F2,RRAS,MRAS
GERM_CELL-SERTOLI_CELL_JUNCTION_SIGNALING	0.0255	0.0363	0.475	RHOC,RRAS,MRAS,GSN
NF-KAPPAB_SIGNALING	0.0255	0.0363	0.475	RRAS,TNFRSF1B,MYD88,MRAS
APOPTOSIS_SIGNALING	0.0326	0.0371	0.475	RRAS,TNFRSF1B,MRAS
CHRONIC_MYELOID_LEUKEMIA_SIGNALING	0.0313	0.0413	0.512	E2F2,RRAS,MRAS
MELANOMA_SIGNALING	0.0476	0.0444	0.512	RRAS,MRAS
PROSTATE_CANCER_SIGNALING	0.0303	0.0445	0.512	E2F2,RRAS,MRAS
THYROID_CANCER_SIGNALING	0.0465	0.0463	0.512	RRAS,MRAS
HIF1ALPHA_SIGNALING	0.0297	0.0468	0.512	RRAS,MAPK7,MRAS

Module 17

Pathway	Ratio	nominal p-value	adjusted p-value	Intersect
Type II Interferon Signaling				
AIRWAY_PATHOLOGY_IN_CHRONIC_OBSTRUCTIVE_PULMONARY_DISEASE	0.250	0.00109	0.365	CXCL3,TNF
ROLE_OF_PKR_IN_INTERFERON_INDUCTION_AND_ANTIVIRAL_RESPONSE	0.0698	0.00248	0.365	IFNG,IRF1,TNF
GERM_CELL-SERTOLI_CELL_JUNCTION_SIGNALING	0.0318	0.00295	0.365	TUBA1C,TUBB2A,TUBA1A,TNF,MAP3K8
IL-12_SIGNALING_AND_PRODUCTION_IN_MACROPHAGES	0.0364	0.00500	0.396	IFNG,IRF1,TNF,MAP3K8
14-3-3-MEDIATED_SIGNALING	0.0351	0.00568	0.396	TUBA1C,TUBB2A,TUBA1A,TNF
RETINOIC_ACID_MEDIATED_APOPTOSIS_SIGNALING	0.0500	0.00639	0.396	IFNG,IRF1,TIPARP
DIFFERENTIAL_REGULATION_OF_CYTOKINE_PRODUCTION_IN_INTESTINAL_EPITHELIAL_CELLS_BY_IL-17A_AND_IL-17F	0.0833	0.0100	0.488	IFNG,TNF
HEPATIC_FIBROSIS___HEPATIC_STELLATE_CELL_ACTIVATION	0.0286	0.0116	0.488	IFNG,SMAD7,CXCL3,TNF
T_HELPER_CELL_DIFFERENTIATION	0.0400	0.0118	0.488	IFNG,TNF,BCL6
ROLE_OF_HYPERCYTOKINEMIA_HYPERCHEMOKINEMIA_IN_THE_PATHOGENESIS_OF_INFLUENZA	0.0645	0.0164	0.518	IFNG,TNF
PRODUCTION_OF_NITRIC_OXIDE_AND_REACTIVE_OXYGEN_SPECIES_IN_MACROPHAGES	0.0256	0.0167	0.518	IFNG,IRF1,TNF,MAP3K8
INTERFERON_SIGNALING	0.0606	0.0185	0.530	IFNG,IRF1
APOPTOSIS_SIGNALING	0.0326	0.0204	0.542	LMNA,BCL2A1,TNF
AIRWAY_INFLAMMATION_IN_ASTHMA	0.250	0.0253	0.628	TNF
ROLE_OF_CYTOKINES_IN_MEDIATING_COMMUNICATION_BETWEEN_IMMUNE_CELLS	0.0465	0.0304	0.667	IFNG,TNF
MSP-RON_SIGNALING_PATHWAY	0.0455	0.0318	0.667	IFNG,TNF
BREAST_CANCER_REGULATION_BY_STATHMIN1	0.0206	0.0340	0.667	TUBA1C,TUBB2A,CDKN1A,TUBA1A
TYPE_I_DIABETES_MELLITUS_SIGNALING	0.0263	0.0355	0.667	IFNG,IRF1,TNF
GRAFT-VERSUS-HOST_DISEASE_SIGNALING	0.0426	0.0359	0.667	IFNG,TNF
TREM1_SIGNALING	0.0377	0.0447	0.791	TREM1,TNF
Others				
B_CELL_RECEPTOR_SIGNALING	0.0274	0.0134	0.498	BCL2A1,EGR1,BCL6,MAP3K8

Module 18

Pathway	Ratio	nominal p-value	adjusted p-value	Intersect
Tyrosine Kinases Activity				
INOSITOL_PHOSPHATE_METABOLISM	0.0376	0.00183	0.682	DMPK,LIMK2,INPPL1,PRKX,CSNK1D
NICOTINATE_AND_NICOTINAMIDE_METABOLISM	0.0417	0.00377	0.701	DMPK,LIMK2,PRKX,CSNK1D
CREB Signaling				
PAK_SIGNALING	0.0273	0.0374	1.00	MYL5,LIMK2,SHC1
EPHRIN_RECEPTOR_SIGNALING	0.0206	0.0406	1.00	LIMK2,GNA11,SHC1,CREB3
Others				
GLYCOSAMINOGLYCAN_DEGRADATION	0.0800	0.0121	1.00	FGFRL1,GUSB
CIRCADIAN_RHYTHM_SIGNALING	0.0645	0.0183	1.00	CREB3,CSNK1D
INSULIN_RECEPTOR_SIGNALING	0.0244	0.0494	1.00	STX4,INPPL1,SHC1

Module 19

Pathway	Ratio	nominal p-value	adjusted p-value	Intersect
T-cell and Natural Killer Cell Mediated Cytolysis				
GRANZYME_B_SIGNALING	0.125	0.00426	0.447	GZMB,PRF1
GRANZYME_A_SIGNALING	0.118	0.00480	0.447	GZMA,PRF1
TUMORICIDAL_FUNCTION_OF_HEPATIC_NATURAL_KILLER_CELLS	0.0833	0.00949	0.706	GZMB,PRF1
GRAFT-VERSUS-HOST_DISEASE_SIGNALING	0.0426	0.0340	1.00	GZMB,PRF1
AUTOIMMUNE_THYROID_DISEASE_SIGNALING	0.0377	0.0424	1.00	GZMB,PRF1
CYTOTOXIC_T_LYMPHOCYTE-MEDIATED_APOPTOSIS_OF_TARGET_CELLS	0.0345	0.0499	1.00	GZMB,PRF1
Platelet-Derived Growth Factor Signaling				
PPAR_SIGNALING	0.0366	0.0139	0.861	PDGFRB,PDGFD,IL18RAP
SPHINGOSINE-1-PHOSPHATE_SIGNALING	0.0280	0.0280	1.00	PDGFRB,S1PR5,PDGFD
PAK_SIGNALING	0.0273	0.0301	1.00	PDGFRB,PDGFD,PXN
Others				
NATURAL_KILLER_CELL_SIGNALING	0.0631	3.99E-06	0.00149	CD244,KLRD1,KLRB1,KIR2DL5A,KIR3DL1,KIR2DL2,KIR3DL3
CROSSTALK_BETWEEN_DENDRITIC_CELLS_AND_NATURAL_KILLER_CELLS	0.0571	3.75E-05	0.00698	KLRD1,PRF1,KIR2DL5A,KIR3DL1,KIR2DL2,KIR3DL3
EICOSANOID_SIGNALING	0.0351	0.0483	1.00	PTGDS,PTGDR

Module 20

Pathway	Ratio	nominal p-value	adjusted p-value	Intersect
Tissue Factor Signaling				
ACTIN_CYTOSKELETON_SIGNALING	0.0262	0.000213	0.0792	F2R,NCKAP1,ACTN1,ITGA2B,ITGB3,MYL9
PTEN_SIGNALING	0.0305	0.00164	0.253	YWHAH,ITGA2B,BCL2L1,ITGB3
PI3K_AKT_SIGNALING	0.0288	0.00204	0.253	YWHAH,ITGA2B,BCL2L1,ITGB3
ILK_SIGNALING	0.0221	0.00530	0.319	ACTN1,ITGB3,TGFB1I1,MYL9
INTEGRIN_SIGNALING	0.0206	0.00678	0.319	ACTN1,TLN1,ITGA2B,ITGB3
ROLE_OF_TISSUE_FACTOR_IN_CANCER	0.0283	0.00832	0.319	MMP1,BCL2L1,ITGB3
COAGULATION_SYSTEM	0.0571	0.00847	0.319	F2R,PROS1
ERK_MAPK_SIGNALING	0.0191	0.00879	0.319	YWHAH,TLN1,ITGA2B,ITGB3
FAK_SIGNALING	0.0275	0.00898	0.319	TLN1,ITGA2B,ITGB3
PAK_SIGNALING	0.0273	0.00921	0.319	ITGA2B,ITGB3,MYL9
IL-17A_SIGNALING_IN_FIBROBLASTS	0.0541	0.00943	0.319	MMP1,CXCL5
RAC_SIGNALING	0.0236	0.0136	0.422	NCKAP1,ITGA2B,ITGB3
ROLE_OF_IL-17A_IN_ARTHRITIS	0.0357	0.0208	0.572	MMP1,CXCL5
GLIOMA_INVASIVENESS_SIGNALING	0.0351	0.0215	0.572	F2R,ITGB3
CDC42_SIGNALING	0.0190	0.0243	0.602	ITGA2B,ITGB3,MYL9
AIRWAY_PATHOLOGY_IN_CHRONIC_OBSTRUCTIVE_PULMONARY_DISEASE	0.125	0.0317	0.728	MMP1
CAVEOLAR-MEDIATED_ENDOCYTOSIS_SIGNALING	0.0278	0.0333	0.728	ITGA2B,ITGB3
LEUKOCYTE_EXTRAVASATION_SIGNALING	0.0160	0.0375	0.774	MMP1,ACTN1,CLDN5
ATHEROSCLEROSIS_SIGNALING	0.0241	0.0431	0.838	MMP1,SELP
VEGF_SIGNALING	0.0235	0.0450	0.838	ACTN1,BCL2L1

List of 250 pathways from the Ingenuity canonical pathway database significantly enriched among genes correlated to age in the SAFHS dataset (Fisher's exact test: $p \leq 0.05$). For each module of the BioAge, the list of pathways and the list of genes overlapping with those pathways are given. Pathways of a same BioAge module that overlapped (Jaccard index ≥ 0.25) were regrouped and assigned a same biological function. Gene positively correlated to increases in age are indicated in red and gene negatively correlated to age are indicated in blue.

Supplementary Table 4a. List of genes differentially expressed between HBV vaccine responders and poor-responders

ProbeSet ID	Gene Symbol	Accession Number	log2 Fold-Change	t-statistic	nominal p-value	adjusted p-value
merck-DB350188_a_at	IGHG1	BC019046	0.466	4.33	3.83E-05	1.00
merck-NM_005143_s_at	HP HPR	NM_005143 NM_001126102 NM_0209	-0.689	-4.22	5.77E-05	1.00
merck-CR593799_s_at	---	CR593799	-0.264	-4.08	9.52E-05	1.00
merck-ENST00000341460_at	SYDE2	NM_032184	-0.244	-4.06	0.000103	1.00
merck-HSS00061246_at	---	---	-0.244	-4.01	0.000124	1.00
merck-CR617832_a_at	CD20	NM_152866 NM_021950	0.593	3.99	0.000133	1.00
merck-ENST00000378777_x_at	---	DQ145726	-0.820	-3.92	0.000169	1.00
merck-AK023762_at	SPTBN1	NM_003128	0.254	3.89	0.000187	1.00
merck-ENST00000335534_s_at	KIF18B	BC048263	-0.259	-3.89	0.000187	1.00
merck2-ENST00000378777_at	---	---	-0.831	-3.89	0.000192	1.00
merck2-CD517359_a_at	SPTBN1	NM_003128	0.284	3.83	0.000230	1.00
merck2-AK093024_at	BANK1	NM_017935 NM_001083907 NM_0011	0.515	3.76	0.000299	1.00
merck-BX537613_at	---	BX537613	0.289	3.76	0.000299	1.00
merck-ENST00000372057_s_at	AMMECR1	NM_015365 NM_001025580 NM_0011	0.222	3.73	0.000332	1.00
merck2-NM_152867_a_at	CD20	NM_152866 NM_021950	0.472	3.71	0.000351	1.00
merck-NM_014399_at	TSPAN13	NM_014399	0.461	3.71	0.000358	1.00
merck2-BC031039_at	EFHC2	NM_025184	0.302	3.69	0.000374	1.00
merck-BC027917_s_at	DEFA1 DEFA1B DEFA3	NM_004084 NM_001042500 NM_0052	-0.565	-3.69	0.000382	1.00
merck2-NM_001042500_s_at	---	---	-0.595	-3.67	0.000400	1.00
merck-XM_497642_at	TARM1	NM_001135686	-0.253	-3.62	0.000475	1.00
merck2-AK225546_at	---	---	0.323	3.61	0.000501	1.00
merck2-ENST00000376463_at	---	CR612105	0.325	3.57	0.000562	1.00
merck2-AK225575_at	LRRK1	NM_024652	-0.215	-3.57	0.000566	1.00
merck-NM_175870_at	IGHV5-78	BC002792	0.402	3.56	0.000595	1.00
merck2-BX647990_at	RASGRP3	NM_001139488 NM_170672 NM_0153	0.345	3.55	0.000605	1.00
merck-CB410700_at	---	AC136424	0.259	3.55	0.000614	1.00
merck-NM_025184_s_at	EFHC2	NM_025184	0.322	3.54	0.000637	1.00
merck-CA447901_at	---	---	0.481	3.53	0.000644	1.00
merck2-NM_032738_at	FCRLA	NM_001184866 NM_032738 NM_0011	0.480	3.50	0.000727	1.00
merck-hCT1764595_x_at	---	NG_027989	-0.230	-3.49	0.000738	1.00
merck-NM_001813_at	CENPE	NM_001813	-0.306	-3.49	0.000740	1.00
merck-ENST00000316124_at	LOC439914	AK094364	-0.263	-3.49	0.000740	1.00
merck-LIT1834_at	---	AY166681	-0.276	-3.48	0.000772	1.00
merck-NM_030764_s_at	FCRL2	NM_030764	0.393	3.47	0.000795	1.00
merck-AK094902_at	---	AK094902	-0.235	-3.46	0.000818	1.00
merck2-AM180330_a_at	BLNK	NM_013314 NM_001114094	0.285	3.46	0.000821	1.00
merck2-BX503020_at	PDE12	NM_177966	-0.247	-3.45	0.000840	1.00
merck2-BC048131_at	LOC550643	BC048131	0.389	3.45	0.000858	1.00
merck-NM_001039567_at	RPS4Y2	NM_001039567	-0.363	-3.44	0.000882	1.00
merck-NM_017784_s_at	OSBPL10	NM_017784 NM_001174060	0.557	3.43	0.000890	1.00
merck-NM_032738_at	FCRLA	NM_001184866 NM_032738 NM_0011	0.458	3.43	0.000916	1.00
merck-ENST00000380813_at	SNAPC3	BC036031	0.235	3.42	0.000939	1.00
merck-AK097083_at	LOC283663	NR_024433	0.426	3.42	0.000940	1.00
merck-NM_021963_at	NAP1L2	NM_021963	0.405	3.41	0.000953	1.00
merck-NM_006235_at	POU2AF1	NM_006235	0.395	3.39	0.00102	1.00
merck-NM_032539_at	SLITRK2	NM_032539 NM_001144003 NM_00114	0.679	3.38	0.00106	1.00
merck-AK096733_at	LOC729603	NR_003288	-0.244	-3.38	0.00106	1.00
merck-AK094141_s_at	ZMAT1	NM_001011657 NR_036431	0.332	3.37	0.00109	1.00
merck-NM_001925_at	DEFA4	NM_001925	-1.10	-3.37	0.00110	1.00
merck2-NM_021950_at	CD20	NM_152866 NM_021950	0.596	3.37	0.00110	1.00
merck-NM_001911_at	CTSG	NM_001911	-0.740	-3.37	0.00111	1.00
merck2-AI382268_at	BACH2	NM_001170794	0.247	3.36	0.00113	1.00
merck-NM_014888_at	FAM3C	NM_014888 NM_001040020	0.426	3.35	0.00118	1.00
merck2-AK025965_at	BOD1L	NM_148894	-0.205	-3.34	0.00123	1.00
merck-NM_002777_at	PRTN3	NM_002777	-0.282	-3.33	0.00124	1.00
merck-AK025613_at	STRBP	NM_018387 NM_001171137 NR_03323	0.283	3.33	0.00125	1.00
merck-ENST00000373502_at	CHIC1	NM_001039840	0.246	3.33	0.00125	1.00
merck-BC035211_at	---	BC035211	0.210	3.31	0.00134	1.00
merck2-BX641093_at	---	---	-0.211	-3.31	0.00134	1.00
merck-NM_001025580_at	AMMECR1	NM_015365 NM_001025580 NM_0011	0.182	3.30	0.00139	1.00
merck-XM_067085_a_at	---	---	-0.297	-3.30	0.00139	1.00
merck2-M92449_at	NAAA	NM_001042402	-0.243	-3.29	0.00140	1.00
merck2-AW613529_at	TPCN2	NM_139075	-0.215	-3.28	0.00145	1.00
merck2-N48311_at	SSPN	NM_005086 NM_001135823	0.305	3.28	0.00147	1.00
merck-NM_005346_s_at	HSPA1B	NM_005346	-0.309	-3.28	0.00147	1.00
merck-NM_020993_at	BCL7A	NM_020993 NM_001024808	0.295	3.27	0.00149	1.00
merck-NM_000475_at	NR0B1	NM_000475	-0.216	-3.27	0.00150	1.00
merck-NM_032226_s_at	ZCCHC7	NM_032226	0.220	3.27	0.00151	1.00
merck-L17326_at	---	L17326	-0.299	-3.27	0.00153	1.00
merck-AK128305_a_at	LOC100132741 FLJ46446	AK126424	0.396	3.26	0.00155	1.00
merck2-AF343666_at	---	---	0.264	3.26	0.00157	1.00
merck-BC071811_s_at	LOC390940	NM_001193621 NM_001193622	-0.257	-3.26	0.00158	1.00
merck-NM_017570_s_at	OPLAH	NM_017570	-0.257	-3.26	0.00158	1.00
merck-AK090917_a_at	TMEM47	NM_031442	0.368	3.25	0.00159	1.00
merck-NM_018159_at	NUDT11	NM_018159	0.431	3.25	0.00160	1.00
merck2-AY358278_at	ANGPTL1	NM_004673	0.256	3.24	0.00165	1.00
merck2-BQ574789_at	PRICKLE1	NM_153026 NM_001144881 NM_00114	0.359	3.24	0.00168	1.00
merck-XM_941496_at	CCDC42B	NM_001144872	-0.200	-3.23	0.00170	1.00
merck-CR607463_at	---	XR_109139 XR_109140 XR_111345 XR	-0.216	-3.22	0.00175	1.00
merck-AK057582_a_at	INO80	NM_017553	-0.219	-3.22	0.00179	1.00
merck-ENST00000329990_at	---	AF090928	-0.237	-3.21	0.00183	1.00
merck2-AK092411_at	---	AK092411	-0.201	-3.21	0.00184	1.00
merck2-NM_021034_x_at	IFITM3	NM_021034	-0.560	-3.20	0.00188	1.00
merck-AW138288_at	---	---	-0.210	-3.19	0.00195	1.00
merck-NM_000939_at	POMC	NM_001035256 NM_000939	-0.266	-3.18	0.00199	1.00
merck-NM_001040084_s_at	ANXA8L1 ANXA8L2 ANXA8	NM_001098845 NM_001630 NM_0010	-0.294	-3.18	0.00199	1.00
merck2-AI125130_at	ZC3H13	NM_015070	-0.238	-3.18	0.00202	1.00
merck-NM_032452_at	JPH4	NM_032452 NM_001146028	-0.208	-3.18	0.00202	1.00
merck-NM_021722_at	ADAM22	NM_021723 NM_021722 NM_016351	0.224	3.17	0.00206	1.00
merck-NM_004847_at	AIF1	NM_004847	-0.328	-3.17	0.00209	1.00
merck-NM_000601_a_at	HGF	NM_000601 NM_001010932	-0.324	-3.16	0.00210	1.00
merck-AI125255_s_at	LOC404266	NR_033205 NR_033202 NR_033203	-0.138	-3.16	0.00211	1.00
merck-AK002111_a_at	KPNA6	NM_012316	-0.231	-3.16	0.00212	1.00
merck2-DQ893507_a_at	CD20	NM_152866 NM_021950	0.427	3.15	0.00218	1.00
merck-AA761555_at	---	---	0.208	3.15	0.00219	1.00
merck-BX648376_s_at	CD300E	NM_181449	-0.226	-3.15	0.00220	1.00
merck-AK123284_a_at	DMXL2	NM_001174116 NM_015263 NM_00117	-0.253	-3.15	0.00220	1.00
merck-ENST00000377058_at	CXorf24	BC025179	0.295	3.15	0.00222	1.00
merck-BM724616_at	---	---	0.253	3.15	0.00222	1.00
merck-NM_000017_at	ACADS	NM_000017	-0.184	-3.15	0.00223	1.00
merck-NM_004673_at	ANGPTL1	NM_004673	0.285	3.14	0.00224	1.00
merck-NM_000109_at	DMD	NM_004022 NM_004014 NM_004013 N	0.544	3.14	0.00228	1.00
merck-NM_058186_s_at	FAM3B	NM_058186 NM_206964	-0.471	-3.14	0.00228	1.00
merck-AF143331_at	---	AF143331	-0.181	-3.13	0.00236	1.00
merck-AL833646_at	LOC285084	AL833646	-0.165	-3.12	0.00240	1.00
merck-AF086013_at	---	AF086013	0.153	3.12	0.00240	1.00
merck-XM_944199_at	---	AC007244	-0.315	-3.12	0.00242	1.00
merck-NM_002483_at	CEACAM6	NM_002483	-0.685	-3.12	0.00242	1.00
merck2-BX641138_at	RAB30	BX641138	0.276	3.12	0.00243	1.00
merck-NM_198519_at	---	XM_002343118 XM_003119510 XM_00	0.294	3.11	0.00246	1.00
merck-BI765156_at	---	---	-0.201	-3.11	0.00247	1.00
merck-BC033758_a_at	ADAP2	NM_018404	-0.231	-3.10	0.00254	1.00
merck-X56197_s_at	XIST	NR_001564	1.97	3.10	0.00254	1.00
merck-NM_170672_s_at	RASGRP3	NM_001139488 NM_170672 NM_0153	0.392	3.10	0.00254	1.00
merck2-AK092049_at	CLCN5	NM_001127899 NM_001127898 NM_00	0.259	3.10	0.00259	1.00
merck-U54711_a_at	BIRC3	NM_001165	0.232	3.09	0.00261	1.00
merck-NM_001005287_x_at	OR2A1 OR2A42	NM_001005287 NM_001001802	0.282	3.09	0.00263	1.00
merck-ENST00000379910_x_at	---	AF487336	0.641	3.09	0.00265	1.00
merck2-BF804746_a_at	SUPT6H	NM_003170	-0.237	-3.09	0.00268	1.00
merck2-AB011420_a_at	STK17A	NM_004760	0.447	3.08	0.00269	1.00
merck-NM_175080_at	P2RX5	NM_002561 NM_175080 NM_0012045	0.360	3.08	0.00270	1.00
merck-BC046476_at	LOC100507360	XR_109953 XR_112345 XR_115626	0.239	3.08	0.00272	1.00
merck2-BC033178_x_at	---	---	0.413	3.08	0.00274	1.00
merck-AF379636_x_at	NBPF10 NBPF14 NBPF15 NBPF16 NB	NM_001039703 NM_015383 NM_00117	-0.193	-3.08	0.00275	1.00
merck-ENST00000338882_at	---	AK127146	-0.128	-3.08	0.00276	1.00
merck-AW975021_s_at	---	NG_012559	0.264	3.07	0.00281	1.00
merck-BE670974_at	---	AY528561	0.248	3.06	0.00292	1.00

Legend
- 15-gene signature
- Genes repressed in HBV vaccine responders compared to poor-responders
- Genes induced in HBV vaccine responders compared to poor-responders

merck-AK025659_at	---	AK025659	0.296	3.06	0.00294	1.00
merck-AA758573_at	---	AC231981	-0.201	-3.06	0.00294	1.00
merck2-BC046187_at	CXorf23	NM_198279	0.182	3.05	0.00296	1.00
merck-NM_020995_at	HPR	NM_020995	-0.156	-3.05	0.00298	1.00
merck2-AK129777_x_at	---	---	0.654	3.05	0.00300	1.00
merck-AK026467_at	CD22	AK026467	0.229	3.05	0.00302	1.00
merck-NM_194251_at	GPR151	NM_194251	-0.182	-3.04	0.00304	1.00
merck2-BF435813_at	---	---	0.288	3.04	0.00310	1.00
merck-AK054860_at	XIST	NR_001564	2.24	3.03	0.00313	1.00
merck-AL079282_at	---	AL079282	0.593	3.03	0.00314	1.00
merck2-NM_139013_at	MAPK14	NM_001315 NM_139012 NM_139013 N	-0.203	-3.03	0.00318	1.00
merck-AK095945_s_at	---	AK095945	0.182	3.03	0.00319	1.00
merck-NM_032458_at	PHF6	NM_001015877 NM_032458	0.266	3.03	0.00320	1.00
merck2-AY172957_x_at	---	---	0.645	3.02	0.00328	1.00
merck-NM_033446_s_at	FAM125B	NM_033446	-0.283	-3.02	0.00328	1.00
merck2-AK096690_at	FCRL1	NM_052938 NM_001159397 NM_00115	0.540	3.02	0.00328	1.00
merck-NM_001816_at	CEACAM8	NM_001816	-0.803	-3.01	0.00336	1.00
merck2-NM_001040077_x_at	---	---	0.630	3.01	0.00337	1.00
merck2-BC041570_at	TRPM2	NM_003307	-0.239	-3.01	0.00337	1.00
merck2-AA604317_at	---	AC004111	-0.308	-3.01	0.00340	1.00
merck-NM_021034_x_at	IFITM3	NM_021034	-0.567	-3.01	0.00341	1.00
merck-AF379633_x_at	NBPF10 NBPF14 NBPF15 NBPF16 NBI	NM_001039703 NM_015383 NM_00117	-0.189	-3.00	0.00344	1.00
merck2-BM983732_at	XIST	NR_001564	2.29	3.00	0.00344	1.00
merck-hCT2255763_at	---	---	0.284	3.00	0.00346	1.00
merck-NM_002526_at	NT5E	NM_002526	0.508	3.00	0.00351	1.00
merck-BM547816_a_at	LIX1	NM_153234	0.266	2.99	0.00357	1.00
merck-NM_006885_at	ZFHX3	NM_006885 NM_001164766	-0.168	-2.99	0.00358	1.00
merck2-BC117447_at	SMARCA1	NM_003069 NM_139035	0.285	2.98	0.00364	1.00
merck-AF119866_at	---	AF119866	0.196	2.98	0.00366	1.00
merck2-NM_001816_at	CEACAM8	NM_001816	-0.809	-2.98	0.00367	1.00
merck2-AF035036_x_at	---	U07539	0.336	2.98	0.00372	1.00
merck-NM_175886_at	PRPS1L1	NM_175886	-0.159	-2.97	0.00374	1.00
merck2-X56198_at	XIST	NR_001564	1.37	2.97	0.00379	1.00
merck-BX649112_s_at	COBLL1	BX649112	0.209	2.97	0.00381	1.00
merck-NM_018125_at	ARHGEF10L	NM_018125 NM_001011722	-0.216	-2.97	0.00384	1.00
merck-BQ423246_s_at	---	AL355520	0.245	2.96	0.00390	1.00
merck-BG193833_s_at	RPS4Y1 RPS4Y2	NM_001008 NM_001039567	-1.95	-2.96	0.00390	1.00
merck-XM_938841_at	---	NG_024791	-0.436	-2.96	0.00393	1.00
merck-NM_015884_a_at	MBTPS2	NM_015884	0.168	2.96	0.00396	1.00
merck-NM_014750_at	DLGAP5	NM_014750 NM_001146015	-0.249	-2.95	0.00397	1.00
merck-BX505106_a_at	BBS7	NM_176824 NM_018190	-0.176	-2.95	0.00398	1.00
merck-AV658731_at	---	M97168	2.12	2.95	0.00403	1.00
merck-NM_012471_at	TRPC5	NM_012471	0.177	2.95	0.00405	1.00
merck-NM_032591_a_at	SLC9A7	NM_032591	0.227	2.94	0.00415	1.00
merck-CR749383_at	XIST	NR_001564	2.34	2.94	0.00418	1.00
merck-XM_942050_at	---	NG_008685	-0.122	-2.94	0.00418	1.00
merck-NM_004469_at	FIGF PIR-FIGF	NM_004469 NR_037859	0.301	2.94	0.00418	1.00
merck-NM_006777_at	ZBTB33	NM_001184742 NM_006777	0.165	2.93	0.00423	1.00
merck2-AF000985_at	DDX3Y	NM_001122665 NM_004660	-0.998	-2.93	0.00425	1.00
merck-NM_001972_at	ELANE	NM_001972	-0.628	-2.93	0.00425	1.00
merck-AA322245_s_at	---	AC010969	-0.299	-2.93	0.00426	1.00
merck-NM_001947_s_at	DUSP7	NM_001947	-0.186	-2.93	0.00426	1.00
merck-NM_014562_at	OTX1	NM_014562 NM_001199770	-0.191	-2.93	0.00427	1.00
merck-BX641058_s_at	---	BX641058	-0.398	-2.92	0.00433	1.00
merck2-NM_001831_at	CLU	NM_001831 NM_203339 NM_00117113	0.156	2.92	0.00436	1.00
merck2-BX640620_x_at	---	---	0.566	2.92	0.00437	1.00
merck-BI461644_a_at	MEX3C	NM_016626	-0.144	-2.92	0.00439	1.00
merck-AK092185_s_at	---	BC032890	0.307	2.92	0.00445	1.00
merck2-EL947810_at	DHRS4L2	AY920361	-0.292	-2.91	0.00449	1.00
merck2-NM_001007_x_at	---	---	0.0973	2.91	0.00450	1.00
merck-NM_000071_s_at	CBS	NM_000071 NM_001178008	-0.239	-2.91	0.00454	1.00
merck2-NM_032451_at	SPIRE2	NM_032451	-0.160	-2.90	0.00461	1.00
merck-NM_152633_at	FANCB	NM_001018113 NM_152633	0.337	2.90	0.00461	1.00
merck-NM_005401_at	PTPN14	NM_005401	-0.184	-2.90	0.00469	1.00
merck-ENST00000371256_at	GRIA3	NM_007325 NM_000828	0.326	2.90	0.00471	1.00
merck-BG054624_at	---	---	-0.181	-2.89	0.00474	1.00
merck-NM_017666_at	ZNF280C	NM_017666	0.246	2.89	0.00477	1.00
merck-BX116938_at	ZFY-AS1	XR_109785 XR_115519	-0.321	-2.89	0.00479	1.00
merck-BC033940_at	---	---	-0.264	-2.89	0.00481	1.00
merck-BX464612_s_at	ASNS	NM_133436 NM_001673 NM_183356 N	0.210	2.89	0.00486	1.00
merck2-AK027592_at	ZNF382	NM_032825	0.274	2.88	0.00492	1.00
merck-NM_052938_at	FCRL1	NM_052938 NM_001159397 NM_00115	0.541	2.88	0.00497	1.00
merck-ENST00000368180_a_at	FCRL2	NM_030764	0.321	2.88	0.00499	1.00
merck-BF755867_at	---	AL590065	0.215	2.88	0.00500	1.00
merck2-AK093149_x_at	MYO5B	NM_001080467	0.148	2.87	0.00503	1.00
merck-AK095793_at	PRKXP1	AK095793	0.434	2.87	0.00503	1.00
merck-AK095439_at	---	AK095439	0.375	2.87	0.00503	1.00
merck2-BC036314_at	---	---	-0.566	-2.87	0.00516	1.00
merck2-BX092881_at	---	AC091821	0.257	2.86	0.00520	1.00
merck-NM_152339_at	SPATA2L	NM_152339	-0.211	-2.86	0.00520	1.00
merck-NM_181332_at	NLGN4X	NM_020742 NM_181332	0.470	2.86	0.00521	1.00
merck-BC005248_a_at	EIF1AY	NM_004681	-1.94	-2.86	0.00525	1.00
merck-AL713776_at	DKFZp434L192	NR_026929	-0.167	-2.86	0.00525	1.00
merck-NM_023068_s_at	SIGLEC1	NM_023068	-0.709	-2.86	0.00525	1.00
merck-NM_004653_at	KDM5D	NM_001146705 NM_004653 NM_00114	-1.50	-2.86	0.00527	1.00
merck2-BC016381_x_at	---	---	0.575	2.86	0.00528	1.00
merck2-DA322541_at	DMXL2	NM_001174116 NM_015263 NM_00117	-0.260	-2.86	0.00528	1.00
merck-BF979936_s_at	HEATR8 HEATR8-TTC4	NM_001039464 NR_026782 NR_03763	-0.229	-2.85	0.00532	1.00
merck2-NM_016529_at	ATP8A2	NM_016529	-0.169	-2.85	0.00534	1.00
merck2-DA885371_a_at	EPB41L4A	NM_022140	-0.240	-2.85	0.00536	1.00
merck2-NM_001008_at	RPS4Y1	NM_001008	-2.01	-2.85	0.00537	1.00
merck2-CF454950_at	GPM6B	NM_001001996 NM_005278 NM_0010(	0.378	2.85	0.00545	1.00
merck2-Z26649_at	PLCB3	NM_000932 NM_001184883	-0.186	-2.85	0.00545	1.00
merck-NM_000693_at	ALDH1A3	NM_000693	0.209	2.85	0.00546	1.00
merck2-BE382874_a_at	MRPS12	NM_033362	-0.182	-2.84	0.00549	1.00
merck2-AF068846_a_at	---	---	0.0843	2.84	0.00552	1.00
merck2-AL699751_at	---	CR616022	0.182	2.84	0.00553	1.00
merck-Y09443_a_at	AGPS	NM_003659	-0.167	-2.84	0.00554	1.00
merck2-AI658826_a_at	EIF1AY	NM_004681	-1.84	-2.84	0.00554	1.00
merck-ENST00000375217_a_at	UBR4	NM_020765	-0.242	-2.84	0.00554	1.00
merck-AK125225_at	ATP8B3	BC033179	-0.217	-2.84	0.00556	1.00
merck-XM_926024_x_at	---	NG_022676	0.132	2.84	0.00556	1.00
merck-NM_002167_s_at	ID3	NM_002167	0.291	2.84	0.00558	1.00
merck-hCT1640487.2_x_at	---	NG_009885	-0.143	-2.84	0.00558	1.00
merck-ENST00000380600_at	TCEANC	BC020095	0.230	2.84	0.00561	1.00
merck2-NM_058171_s_at	INGX	NR_002226	0.371	2.83	0.00577	1.00
merck-AA757671_at	---	AC003014	0.493	2.83	0.00578	1.00
merck-NM_003069_at	SMARCA1	NM_003069 NM_139035	0.279	2.82	0.00579	1.00
merck-NM_001008537_a_at	KIAA2022	NM_001008537	0.382	2.82	0.00582	1.00
merck-NM_001908_s_at	CTSB	NM_001908 NM_147780 NM_147781 N	-0.197	-2.82	0.00586	1.00
merck-NM_021983_x_at	HLA-DRB4 LOC100509582	NM_021983 XM_003120275 XM_00312	-1.77	-2.82	0.00590	1.00
merck-NM_001003698_a_at	RREB1	NM_001003699 NM_001168344 NM_0(	-0.147	-2.82	0.00590	1.00
merck-AF147347_at	---	AF147347	-0.283	-2.82	0.00591	1.00
merck-AF069138_at	---	---	0.164	2.82	0.00592	1.00
merck-AK074226_at	---	AK074226	-0.198	-2.82	0.00595	1.00
merck2-AI864170_at	SENP5	NM_152699	-0.218	-2.81	0.00597	1.00
merck-NM_000408_s_at	GPD2	NM_001083112 NM_000408	-0.169	-2.81	0.00598	1.00
merck2-BC040939_at	LOC386758	NR_037159 NR_037160 NR_037161	0.179	2.81	0.00602	1.00
merck-NM_001725_at	BPI	NM_001725 XM_002345420	-0.703	-2.81	0.00602	1.00
merck2-NM_138450_at	ARL11	NM_138450	-0.294	-2.81	0.00604	1.00
merck-NM_030639_at	BHLHB9	NM_001142524 NM_030639 NM_00114	0.419	2.81	0.00605	1.00
merck-NM_002547_at	OPHN1	NM_002547	0.322	2.81	0.00606	1.00
merck-NM_022003_at	FXYD6	NM_022003 NM_001164831 NM_00116	-0.345	-2.81	0.00610	1.00
merck2-DB485269_at	---	---	0.169	2.81	0.00610	1.00
merck-NM_004619_at	TRAF5	NM_004619 NM_145759 NM_0010339	0.276	2.80	0.00614	1.00
merck-ENST00000311191_s_at	LRCH1	NM_001164213	0.138	2.80	0.00621	1.00
merck-AK097140_at	BCORP1	NR_033732 NR_002923	-0.317	-2.80	0.00626	1.00
merck-ENST00000355088_a_at	KAT6B	NM_012330	0.143	2.80	0.00629	1.00
merck-CR593697_a_at	PLSCR1	NM_021105	-0.322	-2.79	0.00633	1.00

merck-R08351_s_at	---	NG_023798	-0.166	-2.79	0.00635	1.00
merck-NM_152761_at	CXorf58	NM_152761 NM_001169574	0.341	2.79	0.00637	1.00
merck-AL833181_at	---	AL833181	0.219	2.79	0.00638	1.00
merck-NM_001412_at	EIF1AX	NM_001412	0.405	2.79	0.00639	1.00
merck-G36912_at	---	AF266850	-0.201	-2.79	0.00639	1.00
merck2-BC089400_at	MAP7D2	NM_001168465 NM_152780 NM_00116	0.296	2.79	0.00639	1.00
merck2-NM_015187_at	SEL1L3	NM_015187	0.287	2.79	0.00640	1.00
merck2-ENST00000368925_at	---	AL050350	-0.232	-2.79	0.00640	1.00
merck2-NM_018387_at	STRBP	NM_018387 NM_001171137 NR_03323	0.249	2.79	0.00641	1.00
merck2-AM393028_a_at	STRBP	NM_018387 NM_001171137 NR_03323	0.294	2.79	0.00645	1.00
merck2-CR621041_at	FRA10AC1	NM_145246	0.168	2.78	0.00653	1.00
merck-NM_004660_at	DDX3Y	NM_001122665 NM_004660	-1.82	-2.78	0.00653	1.00
merck2-AK126502_at	TRAF5	NM_004619 NM_145759 NM_0010339	0.280	2.78	0.00654	1.00
merck-AK123757_a_at	EBF1	NM_024007	0.350	2.78	0.00657	1.00
merck-D16877_at	---	NG_011791	0.185	2.78	0.00657	1.00
merck-NM_001040077_x_at	---	---	0.504	2.78	0.00663	1.00
merck-AK057794_x_at	LOC143666	NR_026967	-0.171	-2.77	0.00674	1.00
merck-NM_013432_at	TONSL	NM_013432	-0.174	-2.77	0.00678	1.00
merck-NM_002575_at	SERPINB2	NM_001143818 NM_002575	-0.321	-2.77	0.00681	1.00
merck-BC017958_at	CCDC160	NM_001101357	0.667	2.77	0.00682	1.00
merck-BC069446_at	RNF185	BC017302	0.155	2.77	0.00685	1.00
merck2-NM_017863_s_at	---	---	0.533	2.76	0.00690	1.00
merck-NM_001165_at	BIRC3	NM_001165 NM_182962	0.293	2.76	0.00692	1.00
merck-NM_004654_at	USP9Y	NM_004654	-1.47	-2.76	0.00693	1.00
merck-BF115658_a_at	ITGB3	NM_000212	-0.256	-2.76	0.00693	1.00
merck-NM_145802_at	SEPT6	NM_145800 NM_145802	0.278	2.76	0.00694	1.00
merck-NM_152732_at	RSPH9	NM_152732 NM_001193341	-0.336	-2.76	0.00695	1.00
merck-NM_031438_at	NUDT12	NM_031438	-0.283	-2.76	0.00700	1.00
merck-AK056310_a_at	ZAK	NM_133646	-0.167	-2.76	0.00702	1.00
merck2-NM_007071_x_at	HHLA3	NM_001031693 NM_001036646 NR_02	0.180	2.76	0.00704	1.00
merck-NM_032024_a_at	C10orf11	NM_032024	-0.218	-2.76	0.00704	1.00
merck2-AK024285_at	STRBP	NM_018387 NM_001171137 NR_03323	0.260	2.76	0.00704	1.00
merck2-BX647065_x_at	PGBD2	BX647065	-0.180	-2.76	0.00705	1.00
merck2-AV694034_at	---	X06096	-0.132	-2.76	0.00705	1.00
merck-NM_004297_a_at	GNA14	NM_004297	-0.103	-2.76	0.00706	1.00
merck-NM_006084_at	IRF9	NM_006084	-0.209	-2.76	0.00706	1.00
merck-NM_016539_s_at	SIRT6	NM_016539 NM_001193285	-0.195	-2.75	0.00708	1.00
merck-NM_014332_at	SMPX	NM_014332	0.290	2.75	0.00708	1.00
merck-CF123899_at	---	---	-0.141	-2.75	0.00709	1.00
merck-NM_138399_at	TMEM44	NM_138399 NM_001011655 NM_00116	-0.224	-2.75	0.00717	1.00
merck-AI686166_s_at	NCRNA00185	NR_001544	-0.513	-2.75	0.00717	1.00
merck-NM_005449_at	FAIM3	NM_005449 NM_001142473 NM_00119	0.243	2.75	0.00719	1.00
merck-BX470592_at	SRD5A1P1	NR_028597	0.160	2.75	0.00721	1.00
merck-NM_007281_at	SCRG1	NM_007281	-0.276	-2.75	0.00725	1.00
merck-AA747306_at	BCL11A	NM_022893	0.236	2.74	0.00727	1.00
merck2-NM_152909_at	ZNF548	NM_001172773 NM_152909	0.207	2.74	0.00730	1.00
merck-NM_173512_s_at	SLC38A11	NM_001199148 NM_173512	0.276	2.74	0.00732	1.00
merck-NM_001039933_s_at	CD79B	NM_000626 NM_021602 NM_0010399	0.325	2.74	0.00735	1.00
merck-NM_174951_at	FAM9A	NM_001171186 NM_174951	0.282	2.74	0.00739	1.00
merck2-BC005008_at	CEACAM6	NM_002483	-0.653	-2.74	0.00739	1.00
merck2-NM_001015001_s_at	---	---	-0.277	-2.74	0.00740	1.00
merck-NM_032576_s_at	TXLNG2P	NM_032576	-1.20	-2.74	0.00741	1.00
merck-BX115310_at	---	AC109927	-0.177	-2.74	0.00742	1.00
merck-NM_020142_s_at	NDUFA4L2	NM_020142	-0.182	-2.74	0.00744	1.00
merck-AK095618_a_at	---	AK095618	-0.208	-2.73	0.00748	1.00
merck-AK095064_at	---	AK095064	-0.129	-2.73	0.00751	1.00
merck2-NM_182660_at	UTY	NM_182660 NM_182659 NM_007125	-0.895	-2.73	0.00751	1.00
merck-BI491675_at	---	AF324931	0.377	2.73	0.00753	1.00
merck-AK022993_at	---	XR_112926 XR_113938	0.164	2.73	0.00753	1.00
merck-ENST00000354256_a_at	TTTY10	NR_001542	-0.669	-2.73	0.00756	1.00
merck-AF085872_at	---	AF085872	0.183	2.73	0.00758	1.00
merck-NM_006922_at	SCN3A	NM_006922 NM_001081676 NM_0010	0.402	2.73	0.00762	1.00
merck-hCT1841255.1_at	---	---	-0.172	-2.73	0.00763	1.00
merck2-AA622265_at	LOC100507718 LOC100509457	XM_003119326 XM_003120269 XM_00	-2.22	-2.73	0.00764	1.00
merck2-NM_030824_at	ZNF442	NM_030824	0.196	2.73	0.00765	1.00
merck2-NM_182660_x_at	UTY	NM_182660 NM_182659 NM_007125	-0.530	-2.73	0.00768	1.00
merck2-NM_199334_at	THRA	NM_199334	-0.245	-2.72	0.00769	1.00
merck-AL049449_at	GAB1	NM_207123 NM_002039	-0.196	-2.72	0.00770	1.00
merck-AK054601_at	---	AK054601	-0.149	-2.72	0.00772	1.00
merck-AB209628_at	HLA-DQA1	AB209628	1.02	2.72	0.00780	1.00
merck-NM_006418_s_at	OLFM4	NM_006418	-0.923	-2.72	0.00784	1.00
merck-NM_133369_at	UNC5A	NM_133369	-0.174	-2.71	0.00792	1.00
merck-NM_015896_at	ZMYND10	NM_015896	-0.197	-2.71	0.00794	1.00
merck2-AF085412_at	SLC6A5	NM_004211	-0.165	-2.71	0.00795	1.00
merck-AK093002_at	---	AK093002	-0.133	-2.71	0.00800	1.00
merck-ENST00000381046_at	LOC554174	BC009388	0.194	2.71	0.00804	1.00
merck-AW975799_at	---	NG_016249	0.266	2.71	0.00807	1.00
merck-NM_002564_at	P2RY2	NM_176072 NM_002564 NM_176071	-0.203	-2.71	0.00810	1.00
merck-CD643907_at	---	---	0.159	2.71	0.00812	1.00
merck-NM_006754_at	SYPL1	NM_006754 NM_182715	0.195	2.70	0.00813	1.00
merck2-BX648887_at	---	BX648887	-0.196	-2.70	0.00814	1.00
merck-CD359455_at	ANK3	NM_020987 NM_001149 NM_0012044	0.232	2.70	0.00816	1.00
merck-BC021710_a_at	DDI1	NM_001001711	-0.181	-2.70	0.00817	1.00
merck-AK097122_at	---	AK097122	0.206	2.70	0.00823	1.00
merck2-R36888_at	---	---	-0.188	-2.70	0.00826	1.00
merck2-BF026299_at	---	---	-0.149	-2.70	0.00830	1.00
merck2-BG291982_at	---	---	0.164	2.70	0.00833	1.00
merck2-BC014958_at	STAP1	NM_012108	0.332	2.69	0.00840	1.00
merck-NM_025040_at	ZNF614	NM_025040	0.189	2.69	0.00847	1.00
merck-NM_015507_at	EGFL6	NM_015507 NM_001167890	0.348	2.69	0.00852	1.00
merck-BF369128_at	---	---	-0.159	-2.69	0.00853	1.00
merck2-CB147469_at	CDC42SE2	NM_020240 NM_001038702	0.140	2.69	0.00853	1.00
merck-NR_002914_x_at	SNORD62A SNORD62B	NR_002914 NR_003050	-0.131	-2.69	0.00859	1.00
merck-NM_015210_a_at	CCDC165	NM_015210	-0.147	-2.68	0.00862	1.00
merck-BU681761_at	KDM5C	EF613277	0.482	2.68	0.00871	1.00
merck-NM_001700_at	AZU1	NM_001700	-0.374	-2.68	0.00875	1.00
merck-BX097821_at	---	AC241513	0.199	2.68	0.00876	1.00
merck-ENST00000216255_a_at	---	---	-0.157	-2.68	0.00878	1.00
merck2-AW575038_at	FAIM3	NM_005449 NM_001142473 NM_00119	0.228	2.68	0.00883	1.00
merck2-CR749861_x_at	---	---	0.367	2.68	0.00883	1.00
merck-NM_152693_a_at	LINC00246A LINC00246B	NR_026595 NR_026594	0.233	2.67	0.00888	1.00
merck-NM_003611_at	OFD1	NM_003611	0.255	2.67	0.00889	1.00
merck-ENST00000288838_at	---	NG_024785	-0.741	-2.67	0.00891	1.00
merck2-BU149471_x_at	ANKRD11	NM_013275	-0.172	-2.67	0.00892	1.00
merck-AK055261_at	CTTNBP2NL	NM_018704	-0.189	-2.67	0.00893	1.00
merck2-BQ440490_at	HAUS2	NM_018097 NM_001130447	-0.179	-2.67	0.00898	1.00
merck-BC000212_a_at	GTF3C2	NM_001521 NM_001035521	-0.160	-2.67	0.00902	1.00
merck2-BC068526_at	FAM3C	NM_014888 NM_001040020	0.387	2.67	0.00905	1.00
merck-ENST00000374922_at	LOC550643	BX537532	0.361	2.67	0.00906	1.00
merck2-CA776414_at	---	AC008783	0.209	2.67	0.00907	1.00
merck-NM_017449_at	EPHB2	NM_017449 NM_004442	-0.240	-2.66	0.00913	1.00
merck-NM_006679_at	---	---	-0.143	-2.66	0.00914	1.00
merck-AF019382_s_at	MBL1P	NR_002724	-0.156	-2.66	0.00915	1.00
merck-NM_138737_s_at	HEPH	NM_138737 NM_014799 NM_0011308	0.200	2.66	0.00916	1.00
merck-CR749861_x_at	IGHG2	BX640623	0.337	2.66	0.00918	1.00
merck2-BQ428957_at	---	---	0.154	2.66	0.00920	1.00
merck-BC043399_a_at	ETV6	NM_001987	-0.198	-2.66	0.00922	1.00
merck-NM_002758_at	MAP2K6	NM_002758	-0.177	-2.66	0.00925	1.00
merck-M34181_a_at	PRKACB	NM_182948 NM_002731	0.181	2.66	0.00926	1.00
merck-NM_172107_a_at	KCNQ2	NM_172107 NM_172106 NM_004518 N	-0.163	-2.66	0.00930	1.00
merck-NM_017633_s_at	FAM46A	NM_017633	-0.163	-2.66	0.00931	1.00
merck2-DA409317_s_at	APLP2	NM_001642 NM_001142276 NM_00114	-0.312	-2.66	0.00931	1.00
merck-ENST00000265384_s_at	TJP2	NM_001170630	-0.211	-2.66	0.00932	1.00
merck2-BQ880108_at	---	---	-0.175	-2.66	0.00933	1.00
merck2-BC033623_a_at	UNC93B1	NM_030930	-0.170	-2.65	0.00935	1.00
merck2-NM_001013706_at	PLIN5	NM_001013706	-0.220	-2.65	0.00936	1.00
merck2-AA826176_at	---	AL109753	0.608	2.65	0.00937	1.00
merck-AI074020_at	---	AC004056	0.225	2.65	0.00942	1.00
merck-BU684868_at	---	EF445041	0.153	2.65	0.00942	1.00

merck-AF087961_at	---	AF087961	-0.236	-2.65	0.00943	1.00
merck-NM_020805_at	KLHL14	NM_020805	0.415	2.65	0.00943	1.00
merck-NM_001747_at	CAPG	NM_001747	-0.231	-2.65	0.00944	1.00
merck-NM_017935_at	BANK1	NM_017935 NM_001083907 NM_00112	0.534	2.65	0.00945	1.00
merck-BM998631_at	---	NG_008408	-0.180	-2.65	0.00954	1.00
merck2-DB333082_at	---	---	0.375	2.65	0.00957	1.00
merck-NM_003629_at	PIK3R3	NM_003629 NM_001114172	-0.260	-2.64	0.00961	1.00
merck-L06875_x_at	---	---	0.159	2.64	0.00962	1.00
merck2-CN389991_at	METTL8	NM_024770	0.214	2.64	0.00963	1.00
merck2-AA148029_at	PODXL	NM_001018111 NM_005397	0.178	2.64	0.00966	1.00
merck-BX571745_a_at	NPHP1	NM_000272 NM_207181 NM_00112817	0.198	2.64	0.00967	1.00
merck2-NM_001725_at	BPI	NM_001725 XM_002345420	-0.650	-2.64	0.00970	1.00
merck2-H00590_at	RRAS2	NM_012250 NM_001102669 NM_00117	0.209	2.64	0.00970	1.00
merck2-BC128082_s_at	EDARADD	NM_145861 NM_080738	0.186	2.64	0.00977	1.00
merck2-BC022823_x_at	---	AF047230	0.535	2.64	0.00978	1.00
merck-XM_942068_x_at	UNC93B1	NM_030930	-0.141	-2.64	0.00979	1.00
merck-BC010154_s_at	NOTCH2 NOTCH2NL	NM_024408 NM_001200001 NM_20345	-0.202	-2.64	0.00979	1.00
merck-NM_006108_at	SPON1	NM_006108	0.239	2.64	0.00981	1.00
merck-NM_015284_at	SZT2	NM_015284	-0.151	-2.63	0.00987	1.00
merck-AK122755_a_at	ZNF518B	NM_053042	0.167	2.63	0.00988	1.00
merck2-BC012419_a_at	CD40	NM_001250 NM_152854	0.269	2.63	0.00989	1.00
merck-BX118195_at	---	AL033376	-0.232	-2.63	0.00990	1.00
merck-NM_003436_at	ZNF135	NM_007134 NM_003436 NM_00116452	0.252	2.63	0.00991	1.00
merck-ENST00000382751_at	URB1	NM_014825	-0.145	-2.63	0.00991	1.00
merck-NM_006559_at	KHDRBS1	NM_006559	0.125	2.63	0.00995	1.00
merck2-EF553520_at	CD109	NM_133493 NM_001159587 NM_00115	-0.207	-2.63	0.00996	1.00
merck-NM_173217_s_at	ST6GAL1	NM_173216 NM_003032 NM_173217	0.154	2.63	0.00997	1.00
merck-NM_133325_a_at	PHF10	NM_018288 NM_133325	0.161	2.63	0.00999	1.00
merck-BX648323_a_at	XIST	NR_001564	0.575	2.63	0.0100	1.00
merck-NM_006847_at	LILRB4	BC026309	-0.207	-2.63	0.0101	1.00
merck2-NM_004258_at	CD101	NM_004258	-0.264	-2.63	0.0101	1.00
merck-NM_004202_at	TMSB4Y	NM_004202	-0.687	-2.62	0.0101	1.00
merck-NM_001002916_at	H2BFWT	NM_001002916	-0.137	-2.62	0.0102	1.00
merck-NM_004473_at	FOXE1	NM_004473	0.173	2.62	0.0102	1.00
merck-NM_013314_at	BLNK	NM_013314 NM_001114094	0.286	2.62	0.0102	1.00
merck-NM_000111_at	SLC26A3	NM_000111	-0.196	-2.62	0.0102	1.00
merck2-NM_001098672_at	HEPHL1	NM_001098672	0.316	2.62	0.0102	1.00
merck-DA991164_a_at	FAM114A1	NM_138389 NR_033290	-0.255	-2.62	0.0103	1.00
merck-AF086183_at	---	BX648817	-0.0992	-2.62	0.0103	1.00
merck-AK022249_at	---	AK022249	0.402	2.62	0.0104	1.00
merck2-AL833915_at	TDRD9	NM_153046	-0.293	-2.62	0.0104	1.00
merck-AK123878_s_at	MEG3	AK123878	-0.153	-2.62	0.0104	1.00
merck-BC032026_at	---	BC032026	0.299	2.61	0.0105	1.00
merck-AF527832_a_at	TTTY20	NR_001546	-0.226	-2.61	0.0105	1.00
merck-NM_005564_at	LCN2	NM_005564	-0.700	-2.61	0.0106	1.00
merck-hCT6747_at	---	NG_010722	-0.144	-2.61	0.0106	1.00
merck-AK094711_at	---	AK094711	0.167	2.61	0.0106	1.00
merck2-AK022858_at	WDR3	NM_006784	0.143	2.61	0.0106	1.00
merck2-BG939621_a_at	C20orf30	NM_001009923 NM_001009924 NM_01	0.138	2.61	0.0107	1.00
merck2-BX648896_at	C4orf40	NM_214711	-0.138	-2.60	0.0107	1.00
merck2-ENST00000355899_at	PLS3	NM_005032 NM_001136025 NM_00117	0.247	2.60	0.0107	1.00
merck-NM_052820_a_at	CORO2A	NM_003389 NM_052820	-0.211	-2.60	0.0108	1.00
merck-NM_001040021_at	CD14	NM_000591 NM_001040021 NM_00117	-0.175	-2.60	0.0108	1.00
merck-NM_006785_at	MALT1	NM_006785 NM_173844	0.186	2.60	0.0108	1.00
merck-ENST00000331888_a_at	MAD2L1	NM_002358	-0.231	-2.60	0.0109	1.00
merck2-VZV_OKA_ORF7_at	---	NC_001348	-0.114	-2.60	0.0109	1.00
merck-AL833947_a_at	STRBP	NM_018387 NM_001171137 NR_03323	0.266	2.60	0.0109	1.00
merck-ENST00000308604_s_at	LOC541471 LINC00152	NR_015395 NR_024373 NR_024204 NI	-0.152	-2.60	0.0109	1.00
merck-NM_006790_at	MYOT	NM_006790 NM_001135940	0.225	2.60	0.0109	1.00
merck-BE048858_at	---	---	-0.220	-2.60	0.0109	1.00
merck-NM_014246_at	CELSR1	NM_014246	0.229	2.60	0.0109	1.00
merck2-ENST00000390299_x_at	---	AF043589	0.284	2.59	0.0110	1.00
merck-NM_000443_at	ABCB4	NM_000443 NM_018849 NM_018850	0.254	2.59	0.0110	1.00
merck2-BC002710_at	KLK10	NM_002776 NM_145888 NM_0010775(	-0.125	-2.59	0.0111	1.00
merck-NM_032714_at	INF2	NM_032714	-0.196	-2.59	0.0111	1.00
merck-BC041882_at	---	CR626222	0.232	2.59	0.0112	1.00
merck-NM_002285_at	AFF3	NM_002285 NM_001025108	0.375	2.59	0.0112	1.00
merck-CA843851_a_at	NEURL	NM_004210	-0.187	-2.59	0.0112	1.00
merck-AW813711_at	---	---	0.141	2.59	0.0113	1.00
merck-AW835608_at	---	---	-0.140	-2.58	0.0113	1.00
merck-NM_021783_at	EDA2R	NM_001199688 NM_001199689 NM_00	0.364	2.58	0.0114	1.00
merck2-NM_175710_s_at	---	---	-0.249	-2.58	0.0114	1.00
merck-CB067148_a_at	PKIG	NM_181805 NM_007066 NM_181804	0.226	2.58	0.0114	1.00
merck-VZV_OKA_ORF64_s_at	---	---	-0.139	-2.58	0.0114	1.00
merck-AW962377_s_at	TXLNG2P	AL832583	-0.928	-2.58	0.0115	1.00
merck-AK094554_at	---	AF454829	-0.162	-2.58	0.0115	1.00
merck-NM_031409_at	CCR6	NM_004367 NM_031409	0.248	2.58	0.0116	1.00
merck-T85133_at	---	---	-0.153	-2.57	0.0116	1.00
merck2-BF174540_x_at	IGHM	AK090464	0.164	2.57	0.0116	1.00
merck-NM_000367_at	TPMT	NM_000367	-0.156	-2.57	0.0117	1.00
merck2-CB957115_s_at	---	---	-0.131	-2.57	0.0117	1.00
merck-BC015092_a_at	PTER	NM_001001484 NM_030664	0.187	2.57	0.0117	1.00
merck-NM_005146_at	SART1	NM_005146	-0.220	-2.57	0.0117	1.00
merck-NM_022872_at	IFI6	NM_002038 NM_022872 NM_022873	-0.431	-2.57	0.0118	1.00
merck2-NM_001005852_at	TXLNG2P	NM_001005852	-1.30	-2.57	0.0118	1.00
merck-BE933112_x_at	PRKY	Y15801	-0.216	-2.57	0.0118	1.00
merck-NM_006781_a_at	C6orf10	NM_006781	-0.122	-2.57	0.0119	1.00
merck2-CK821126_at	MXRA5	NM_015419	0.213	2.57	0.0119	1.00
merck-CD624674_at	BEAN1	NM_001197225 NM_001197224	-0.188	-2.57	0.0119	1.00
merck-NM_033543_s_at	CEACAM21	NM_001098506 NM_033543	-0.238	-2.57	0.0119	1.00
merck-DA926692_at	---	---	-0.127	-2.56	0.0120	1.00
merck2-CMV_UL54_at	---	---	-0.119	-2.56	0.0120	1.00
merck-NM_032551_s_at	KISS1R	NM_032551	-0.260	-2.56	0.0121	1.00
merck-NM_002343_s_at	LTF	NM_002343 NM_001199149	-0.723	-2.56	0.0121	1.00
merck-NM_013943_at	CLIC4	NM_013943	0.170	2.56	0.0121	1.00
merck-NM_004345_at	CAMP	NM_004345	-0.423	-2.56	0.0122	1.00
merck2-NM_004681_at	EIF1AY	NM_004681	-1.16	-2.56	0.0122	1.00
merck-NM_006573_s_at	TNFSF13B	NM_006573 NM_001145645	-0.175	-2.56	0.0122	1.00
merck2-BC130370_at	SERPINB11	NM_080475	0.131	2.56	0.0122	1.00
merck-BE819416_at	---	---	0.149	2.56	0.0122	1.00
merck-NM_182922_s_at	HEATR3	NM_182922	-0.167	-2.56	0.0122	1.00
merck-NM_014689_at	DOCK10	NM_014689	0.148	2.55	0.0122	1.00
merck2-BX538322_at	FGD6	NM_018351	-0.209	-2.55	0.0123	1.00
merck2-BC150285_a_at	MYO10	NM_012334	-0.171	-2.55	0.0123	1.00
merck-ENST00000282333_s_at	ZNF837	NM_001129730 NM_138466	0.165	2.55	0.0123	1.00
merck-AK128852_a_at	---	AK128852	-0.118	-2.55	0.0123	1.00
merck-BQ314506_at	---	---	-0.197	-2.55	0.0123	1.00
merck-XM_939250_at	C5orf65	NM_001161546	-0.141	-2.55	0.0124	1.00
merck-NM_005590_at	MRE11A	NM_005591 NM_005590	-0.136	-2.55	0.0124	1.00
merck2-NM_138999_at	NETO1	NM_138999	-0.109	-2.55	0.0124	1.00
merck-BF952253_at	---	---	-0.159	-2.55	0.0124	1.00
merck-XM_067050_at	---	---	-0.212	-2.55	0.0124	1.00
merck-NM_032536_a_at	NTNG2	NM_032536	-0.207	-2.55	0.0124	1.00
merck-NM_016541_at	GNG13	NM_016541	0.116	2.55	0.0125	1.00
merck2-NM_025152_a_at	NUBPL	NM_025152 NM_001201573 NM_0012(	0.175	2.55	0.0125	1.00
merck2-AK001584_a_at	SPON1	NM_006108	0.221	2.55	0.0125	1.00
merck2-L34717_at	---	L34717	0.354	2.55	0.0125	1.00
merck-BF511462_a_at	LOC286071	AK091759	0.213	2.55	0.0125	1.00
merck-AW665698_at	---	---	-0.173	-2.55	0.0125	1.00
merck-NM_005429_at	VEGFC	NM_005429	-0.181	-2.55	0.0125	1.00
merck-NM_032117_at	MND1	NM_032117	-0.232	-2.55	0.0126	1.00
merck2-CF135398_at	PIK3AP1	NM_152309	-0.220	-2.55	0.0126	1.00
merck-BG756002_at	---	---	-0.125	-2.55	0.0126	1.00
merck-NM_052847_s_at	GNG7	NM_052847	0.170	2.54	0.0126	1.00
merck-BX107845_at	---	AL031319	0.256	2.54	0.0126	1.00
merck2-F01509_at	LIX1	NM_153234	0.170	2.54	0.0126	1.00
merck-NM_198480_at	ZNF615	NM_001199324 NM_198480	0.170	2.54	0.0126	1.00
merck2-AI752658_at	---	---	-0.217	-2.54	0.0126	1.00
merck2-AF097709_at	HTRA1	NM_002775	-0.191	-2.54	0.0127	1.00

merck-HSS00295480_at	---	---	-0.168	-2.54	0.0127	1.00
merck-ENST00000294993_at	---	AK023372	-0.148	-2.54	0.0127	1.00
merck-NM_002123_x_at	LOC100293977	XM_003120273 XM_001714067 XM_00	-0.810	-2.54	0.0127	1.00
merck-AI671847_s_at	---	AC007845	0.216	2.54	0.0128	1.00
merck-AK130566_at	---	AK130566	-0.249	-2.54	0.0128	1.00
merck-AI952248_a_at	VHL	NM_000551 NM_198156	-0.165	-2.54	0.0128	1.00
merck2-AI813659_at	---	AC093873	-0.133	-2.54	0.0128	1.00
merck-NM_001011658_at	TRAPPC2	NM_001011658 NM_014563 NM_00112	0.188	2.54	0.0128	1.00
merck-NM_001029884_at	PLEKHG1	NM_001029884	0.288	2.54	0.0128	1.00
merck-ENST00000328474_at	EIF4B	AF119854	0.147	2.54	0.0129	1.00
merck-CB164481_at	---	AC131182	-0.322	-2.53	0.0129	1.00
merck-BC067869_at	LOC441493	XR_113304	0.235	2.53	0.0129	1.00
merck-ENST00000334516_a_at	KDM6A	NM_021140	0.297	2.53	0.0129	1.00
merck2-R70164_at	---	---	-0.232	-2.53	0.0130	1.00
merck2-NM_001098825_at	TMEM91	NM_001042595 NM_001098825	-0.143	-2.53	0.0130	1.00
merck-BF891877_at	---	---	-0.140	-2.53	0.0130	1.00
merck-XM_212532_at	---	---	-0.176	-2.53	0.0131	1.00
merck-NM_001002247_at	ANAPC11	NM_001002244 NM_016476 NM_0010(	-0.181	-2.53	0.0131	1.00
merck2-BE220053_at	---	AF057280	-0.203	-2.53	0.0131	1.00
merck-NM_003919_at	SGCE	NM_001099401 NM_003919 NM_0010(	0.262	2.53	0.0132	1.00
merck-NM_203458_at	NOTCH2NL	NM_203458	-0.204	-2.53	0.0132	1.00
merck-BF109132_at	---	AL355796	-0.153	-2.53	0.0132	1.00
merck2-BX396168_a_at	PLSCR1	NM_021105	-0.284	-2.53	0.0133	1.00
merck-BF594635_s_at	PAQR5	NM_001104554 NM_017705	-0.135	-2.52	0.0133	1.00
merck-NM_001025463_at	LOC284751	NR_034124	-0.191	-2.52	0.0133	1.00
merck2-NM_006061_at	CRISP3	NM_006061 NM_001190986	-0.607	-2.52	0.0133	1.00
merck-BC034402_a_at	RBM47	NM_001098634 NM_019027	-0.295	-2.52	0.0134	1.00
merck-BC022823_x_at	---	---	0.412	2.52	0.0134	1.00
merck-XM_927287_s_at	---	AC008783	0.249	2.52	0.0134	1.00
merck-XM_930134_x_at	---	---	-0.132	-2.52	0.0134	1.00
merck-NM_144622_at	---	---	-0.135	-2.52	0.0134	1.00
merck-BM979088_s_at	ATRX	NM_000489 NM_138270	0.160	2.52	0.0134	1.00
merck-ENST00000326985_s_at	RBMY1A1 RBMY1B RBMY1D RBMY1E	NM_005058 NM_001006121 NM_0010(	-0.149	-2.52	0.0134	1.00
merck-NM_004258_at	CD101	NM_004258	-0.215	-2.52	0.0134	1.00
merck-AK125162_at	---	AK125162	0.130	2.52	0.0135	1.00
merck-NM_001039900_at	---	AK130368	0.479	2.52	0.0135	1.00
merck2-BG202125_at	---	---	-0.231	-2.52	0.0136	1.00
merck2-NM_001040070_x_at	---	BC031259	0.357	2.52	0.0136	1.00
merck-NM_022831_a_at	AIDA	NM_022831	0.160	2.51	0.0137	1.00
merck2-NM_001024074_at	HNMT	NM_001024074	-0.310	-2.51	0.0137	1.00
merck-NM_175726_at	IL5RA	NM_000564 NM_175726	-0.159	-2.51	0.0137	1.00
merck2-NM_018538_a_at	ERMAP	NM_001017922 NM_018538	-0.243	-2.51	0.0137	1.00
merck2-NM_024005_at	---	---	0.236	2.51	0.0137	1.00
merck-CX782565_s_at	NAAA	NM_014435 NM_001042402	-0.229	-2.51	0.0138	1.00
merck-NM_018081_s_at	WRAP53	NM_018081 NM_001143990 NM_00114	-0.226	-2.51	0.0138	1.00
merck-U92981_at	---	U92981	0.299	2.51	0.0138	1.00
merck-AK090788_s_at	DNM1L	NM_012062 NM_012063 NM_005690	0.129	2.51	0.0138	1.00
merck2-CB241297_at	---	---	0.286	2.51	0.0138	1.00
merck2-BQ068166_s_at	---	---	-0.208	-2.51	0.0139	1.00
merck2-BC026213_at	FBXW11	NM_033645 NM_033644 NM_012300	0.101	2.51	0.0139	1.00
merck-ENST00000299550_at	TRIM66	NM_014818	-0.303	-2.51	0.0139	1.00
merck-AF332230_a_at	TTTY3 TTTY3B	NR_001524 NR_002176	-0.165	-2.51	0.0139	1.00
merck2-AL833067_at	---	AL833067	0.154	2.51	0.0140	1.00
merck2-NM_032042_at	FAM172A	NM_032042 NM_001163417 NM_00116	0.109	2.50	0.0140	1.00
merck2-NM_001037501_s_at	---	---	-0.112	-2.50	0.0140	1.00
merck-AK000215_a_at	PGPEP1	NM_017712	-0.197	-2.50	0.0140	1.00
merck-AF196865_at	BPESC1	NR_026783	-0.124	-2.50	0.0141	1.00
merck-BE567163_at	---	---	-0.124	-2.50	0.0141	1.00
merck-AY367054_a_at	FGD4	NM_139241	-0.227	-2.50	0.0141	1.00
merck-NM_138441_s_at	MB21D1	NM_138441	-0.211	-2.50	0.0141	1.00
merck-ENST00000382880_x_at	---	EF589510	0.415	2.50	0.0141	1.00
merck-AF442151_a_at	RSAD2	NM_080657	-0.658	-2.50	0.0142	1.00
merck-ENST00000327008_x_at	SSX2	BC103863	0.392	2.50	0.0142	1.00
merck-AJ133123_a_at	ADCY9	NM_001116	-0.179	-2.50	0.0142	1.00
merck-AA447325_at	---	---	0.186	2.50	0.0142	1.00
merck-hCT1648089.3_at	---	NG_005626	-0.128	-2.50	0.0142	1.00
merck-NM_002578_at	PAK3	NM_001128166 NM_002578 NM_00112	0.636	2.50	0.0143	1.00
merck2-AK093852_at	LTF	NM_002343 NM_001199149	-0.725	-2.50	0.0143	1.00
merck-AK128024_a_at	---	AY598347	-1.17	-2.50	0.0143	1.00
merck-NM_152909_at	ZNF548	NM_001172773 NM_152909	0.181	2.50	0.0143	1.00
merck-NM_001001686_at	---	AK125858	0.263	2.50	0.0144	1.00
merck2-BF681579_at	---	---	-0.116	-2.49	0.0144	1.00
merck-NM_004655_at	AXIN2	NM_004655	0.255	2.49	0.0144	1.00
merck2-NM_004881_at	TP53I3	NM_004881 NM_147184	-0.274	-2.49	0.0144	1.00
merck-XM_926634_at	RABL3	NM_173825	0.198	2.49	0.0144	1.00
merck-NM_032178_at	SLC7A6OS	NM_032178	0.220	2.49	0.0145	1.00
merck-NM_013378_at	VPREB3	NM_013378	0.407	2.49	0.0145	1.00
merck-T68445_a_at	---	AL356358	0.269	2.49	0.0145	1.00
merck-NM_144580_at	C1orf85	NM_144580	-0.286	-2.49	0.0145	1.00
merck-NM_004240_at	TRIP10	NM_004240	-0.194	-2.49	0.0145	1.00
merck-ENST00000296491_at	---	---	-0.125	-2.49	0.0145	1.00
merck2-AA448172_at	---	AC127512	-0.223	-2.49	0.0146	1.00
merck-BC057761_a_at	CCDC149	NM_173463 NM_001130726	-0.381	-2.49	0.0146	1.00
merck-ENST00000378271_a_at	DCLRE1C	NM_001033855 NM_022487 NM_0010(	-0.116	-2.49	0.0146	1.00
merck-BI256526_x_at	RPS4X	NM_001007	0.0996	2.49	0.0146	1.00
merck-NM_000044_a_at	AR	NM_000044 NM_001011645	0.306	2.49	0.0146	1.00
merck-NM_152497_at	STMN1	NM_001145454	-0.221	-2.49	0.0147	1.00
merck2-AK128466_at	---	AK128466	0.157	2.49	0.0147	1.00
merck-AK026286_at	---	AK026286	-0.228	-2.49	0.0147	1.00
merck-BX500576_at	---	---	-0.183	-2.48	0.0148	1.00
merck-ENST00000340515_at	FAM160A1	NM_001109977	0.154	2.48	0.0148	1.00
merck-BE672660_at	---	---	-0.213	-2.48	0.0148	1.00
merck-AK129777_x_at	---	---	0.529	2.48	0.0148	1.00
merck-NM_198472_at	C10orf125	NM_001098483 NM_198472	-0.193	-2.48	0.0149	1.00
merck-BX420480_a_at	PTN	NM_002825	-0.173	-2.48	0.0149	1.00
merck-NR_003029_x_at	SNORD9	NR_003029	-0.195	-2.48	0.0149	1.00
merck2-NM_012334_at	MYO10	NM_012334	-0.140	-2.48	0.0149	1.00
merck2-BM974460_at	LOC100507493	XR_110882 XR_113132 XR_114149	0.228	2.48	0.0150	1.00
merck-ENST00000313276_at	---	AF399356	-0.180	-2.48	0.0150	1.00
merck-ENST00000229824_at	---	---	-0.208	-2.48	0.0150	1.00
merck-AJ519332_x_at	---	---	-0.125	-2.48	0.0150	1.00
merck-NM_182659_a_at	UTY	NM_182659 NM_007125	-0.612	-2.48	0.0150	1.00
merck-NM_003282_at	TNNI2	NM_003282 NM_001145829 NM_00114	-0.279	-2.48	0.0150	1.00
merck-NM_001567_s_at	INPPL1	NM_001567	-0.192	-2.48	0.0151	1.00
merck2-CMV_UL35_at	---	---	-0.132	-2.48	0.0151	1.00
merck2-AK057023_at	LOC100290566	XR_109384 XR_111701 XR_115104	-0.152	-2.48	0.0151	1.00
merck-AK027209_at	---	AK027209	-0.200	-2.47	0.0151	1.00
merck-AK054695_at	LAS1L	AK074087	-0.215	-2.47	0.0152	1.00
merck-NM_144718_at	SPICE1	NM_144718	0.152	2.47	0.0152	1.00
merck-AB209258_at	TRPC5	AB209258	0.540	2.47	0.0152	1.00
merck2-BC011819_at	DDX3X	NM_001356 NM_001193416 NM_0011‌9	0.166	2.47	0.0152	1.00
merck-XM_377230_at	---	AC203597	-0.202	-2.47	0.0152	1.00
merck-BC015129_at	---	BC015129	-0.162	-2.47	0.0153	1.00
merck-NM_001835_at	CLTCL1	NM_007098 NM_001835	-0.287	-2.47	0.0153	1.00
merck-NM_182538_at	SPNS3	NM_182538	-0.292	-2.47	0.0153	1.00
merck2-DQ892828_a_at	SAP30	NM_003864	-0.171	-2.47	0.0153	1.00
merck2-DN919376_a_at	ZC3H13	NM_015070	-0.194	-2.47	0.0153	1.00
merck-ENST00000206681_at	---	---	0.340	2.47	0.0153	1.00
merck-ENST00000375169_at	WNK3	NM_020922 NM_001002838	0.475	2.47	0.0153	1.00
merck-NM_003121_at	SPIB	NM_003121	0.357	2.47	0.0153	1.00
merck-BC020226_a_at	HLA-DOB	NM_002120	0.217	2.47	0.0154	1.00
merck-BC033178_x_at	---	AY869727	0.539	2.47	0.0154	1.00
merck2-BP196448_at	---	---	-0.189	-2.47	0.0154	1.00
merck-AK074440_at	---	AK074440	0.221	2.47	0.0154	1.00
merck2-NM_133439_at	TADA2A	NM_001488 NM_133439 NM_0011661(	-0.135	-2.47	0.0155	1.00
merck2-BC062574_at	NPHP1	NM_000272 NM_207181 NM_00112817	0.185	2.47	0.0155	1.00
merck-BQ361926_at	---	---	-0.108	-2.47	0.0155	1.00
merck2-ENST00000302686_at	BPNT1	NM_006085	0.141	2.47	0.0155	1.00
merck-DR432803_a_at	FAM40B	NM_020704 NM_001134336	-0.136	-2.47	0.0155	1.00

merck-AF086222_at	---	AF086222	-0.145	-2.47	0.0155	1.00
merck2-AL833454_at	SFXN5	AL833454	-0.174	-2.47	0.0155	1.00
merck-AW138771_a_at	TRIM66	NM_014818	-0.124	-2.46	0.0156	1.00
merck2-AF400595_at	CLEC7A	NM_197947 NM_197948	-0.217	-2.46	0.0156	1.00
merck-NM_000256_at	MYBPC3	NM_000256	-0.198	-2.46	0.0156	1.00
merck2-CA438250_at	PARM1	NM_015393	0.206	2.46	0.0156	1.00
merck-BE669821_at	USP27X	NM_001145073	0.245	2.46	0.0157	1.00
merck-NM_014048_at	MKL2	NM_014048	0.209	2.46	0.0157	1.00
merck-AF070581_a_at	---	AF070581	0.309	2.46	0.0157	1.00
merck2-BE698643_x_at	USP24	NM_015306	-0.226	-2.46	0.0157	1.00
merck2-VZV_OKA_O67_up_at	---	---	-0.127	-2.46	0.0157	1.00
merck-NM_207468_at	FAM177B	NM_207468	0.326	2.46	0.0157	1.00
merck-AJ420434_at	NRARP	NM_001004354	-0.181	-2.46	0.0158	1.00
merck2-NM_001010982_x_at	AFMID	NM_001145526 NM_001010982	-0.160	-2.46	0.0158	1.00
merck-NM_004951_at	GPR183	NM_004951	0.260	2.46	0.0159	1.00
merck-NM_005362_s_at	MAGEA3 MAGEA6	NM_005362 NM_005363 NM_175868	0.244	2.46	0.0159	1.00
merck-NM_175617_at	MT1E	NM_175617	-0.158	-2.46	0.0159	1.00
merck-AK025887_at	---	AK025887	-0.182	-2.46	0.0159	1.00
merck-XM_496062_x_at	LOC653075	NR_033933	0.209	2.46	0.0159	1.00
merck2-Rota_G3_s10_s_at	---	---	-0.103	-2.46	0.0160	1.00
merck-NM_021821_at	MRPS35	NM_021821 NM_001190864	0.161	2.45	0.0160	1.00
merck-Contig6529_RC_at	---	---	-0.144	-2.45	0.0160	1.00
merck2-AW814840_x_at	---	---	0.103	2.45	0.0161	1.00
merck2-NM_017940_s_at	---	---	-0.111	-2.45	0.0161	1.00
merck-ENST00000378670_a_at	UQCRQ	NM_014402	-0.207	-2.45	0.0161	1.00
merck-BC047330_at	---	---	0.152	2.45	0.0161	1.00
merck2-BE464047_at	ALDOB	NM_000035	0.203	2.45	0.0162	1.00
merck-NM_001400_at	S1PR1	NM_001400	0.203	2.45	0.0162	1.00
merck-AW973537_at	---	NG_015661	0.393	2.45	0.0163	1.00
merck-AF086064_at	---	---	0.212	2.45	0.0163	1.00
merck2-AK123319_at	NETO1	NM_138966	0.179	2.45	0.0163	1.00
merck-hCT1643393.2_at	---	---	-0.110	-2.45	0.0163	1.00
merck-NM_203287_x_at	PSG11	NM_002785 NM_203287 NM_00111341	0.130	2.45	0.0163	1.00
merck2-NM_001931_at	DLAT	NM_001931	0.247	2.45	0.0163	1.00
merck-X03068_x_at	HLA-DQB1	NM_002123 XM_003119997 XM_00312	0.938	2.45	0.0164	1.00
merck-BC016381_x_at	IGHG1	Y17957	0.488	2.45	0.0164	1.00
merck-ENST00000378925_s_at	WWC2	NM_024949	-0.167	-2.45	0.0164	1.00
merck-NM_014806_at	RUSC2	NM_001135999 NM_014806	-0.142	-2.45	0.0164	1.00
merck-BG495163_at	---	---	0.129	2.44	0.0164	1.00
merck2-NM_017817_at	RAB20	NM_017817	-0.298	-2.44	0.0164	1.00
merck2-AF379636_s_at	---	---	-0.109	-2.44	0.0164	1.00
merck-AK074192_at	---	AK074192	-0.204	-2.44	0.0164	1.00
merck-AK130615_at	---	AK130615	0.171	2.44	0.0164	1.00
merck-ENST00000326237_at	LOC442075	XR_110030 XR_112457 XR_113459	0.141	2.44	0.0164	1.00
merck-BC043572_at	LOC339874	BC043572	-0.251	-2.44	0.0165	1.00
merck-NM_001821_at	CHML	NM_001821	0.273	2.44	0.0166	1.00
merck-NM_001572_at	IRF7	NM_001572 NM_004029 NM_004031	-0.321	-2.44	0.0166	1.00
merck-NM_138439_at	FLYWCH2	NM_138439 NM_001142499 NM_00114	-0.138	-2.44	0.0167	1.00
merck-CB962149_at	---	AL132795	0.212	2.44	0.0167	1.00
merck-hCT1651059.3_at	---	NG_001122	-0.122	-2.44	0.0168	1.00
merck-NM_001017923_at	C14orf28	NM_001017923	0.182	2.44	0.0168	1.00
merck-AL544754_a_at	LGALS3BP	NM_005567	-0.180	-2.44	0.0168	1.00
merck-NM_002852_at	PTX3	NM_002852	-0.202	-2.44	0.0168	1.00
merck2-BX281223_at	SCN3A	NM_006922 NM_001081676 NM_00108	0.120	2.44	0.0168	1.00
merck-ENST00000382764_at	---	AK026367	-0.126	-2.44	0.0168	1.00
merck-AI733562_at	---	U10116	-0.239	-2.43	0.0168	1.00
merck-NM_024506_at	GLB1L	NM_024506	-0.362	-2.43	0.0169	1.00
merck-XM_291763_s_at	EIF3L	NM_016091	0.126	2.43	0.0169	1.00
merck2-AI659225_at	---	AL627402	0.190	2.43	0.0169	1.00
merck-NM_032880_at	IGSF21	NM_032880	-0.139	-2.43	0.0169	1.00
merck2-L37307_x_at	---	U07546	0.343	2.43	0.0170	1.00
merck-NM_003770_at	KRT37	NM_003770	-0.119	-2.43	0.0171	1.00
merck-BX091412_at	---	AL772392	0.430	2.43	0.0171	1.00
merck2-BG959880_at	---	---	-0.160	-2.43	0.0171	1.00
merck-AF024541_a_at	AFF1	NM_001166693 NM_005935	-0.178	-2.43	0.0171	1.00
merck-ENST00000329684_a_at	TTTY8 TTTY8B	NR_001533 NR_003591	-0.389	-2.43	0.0171	1.00
merck-NM_004065_at	CDR1	NM_004065	0.532	2.43	0.0171	1.00
merck-NM_201437_at	TCEA1	NM_006756 NM_201437	0.149	2.43	0.0171	1.00
merck-NM_005180_at	BMI1 COMMD3-BMI1	NM_005180 NM_001204062	0.223	2.43	0.0171	1.00
merck-NM_005296_at	LPAR4	NM_005296	0.654	2.43	0.0171	1.00
merck-CR936684_a_at	UTY	NM_007125	-0.738	-2.43	0.0171	1.00
merck-NM_024122_at	APOO	NM_024122 NR_026545	0.275	2.43	0.0171	1.00
merck-AK127655_at	LOC100131231	AK127655	-0.191	-2.43	0.0172	1.00
merck2-NM_002483_at	CEACAM6	NM_002483	-0.556	-2.43	0.0172	1.00
merck2-BX473738_x_at	TNPO1	NM_002270 NM_153188	-0.0988	-2.43	0.0172	1.00
merck-NM_152785_at	GCET2	NM_152785 NM_001190259 NM_00119	0.212	2.43	0.0172	1.00
merck-NM_006061_at	CRISP3	NM_006061 NM_001190986	-0.579	-2.42	0.0173	1.00
merck2-BC013329_a_at	DPP4	NM_001935	0.192	2.42	0.0173	1.00
merck-BC022384_at	---	BC022384	-0.216	-2.42	0.0173	1.00
merck-NM_002760_at	PRKY	NR_028062	-0.546	-2.42	0.0173	1.00
merck-DA818672_a_at	BANK1	NM_017935 NM_001083907 NM_00112	0.310	2.42	0.0174	1.00
merck-NM_001804_at	CDX1	NM_001804	-0.154	-2.42	0.0174	1.00
merck2-BU147541_at	BUB3	NM_004725	0.160	2.42	0.0174	1.00
merck-NM_001013722_at	TNRC18	AY129022	-0.177	-2.42	0.0174	1.00
merck-NM_001989_at	EVX1	NM_001989	-0.153	-2.42	0.0175	1.00
merck-NM_203504_s_at	G3BP2	NM_203505 NM_012297 NM_203504	0.149	2.42	0.0175	1.00
merck2-BM740515_at	PRR11	NM_018304	-0.164	-2.42	0.0175	1.00
merck2-AI278965_at	---	---	-0.241	-2.42	0.0176	1.00
merck-hsa-mir-507_x_at	MIR507	NR_030234	0.182	2.42	0.0176	1.00
merck-NM_001715_at	BLK	NM_001715	0.244	2.42	0.0176	1.00
merck2-NM_000051_at	ATM	NM_000051	0.175	2.42	0.0176	1.00
merck-BQ347127_at	---	AL035417	-0.150	-2.42	0.0176	1.00
merck-AF119870_at	---	AF119870	-0.190	-2.42	0.0176	1.00
merck2-AB033049_at	ANKRD50	NM_020337 NM_001167882	-0.214	-2.42	0.0176	1.00
merck2-BQ185231_at	USP9X	NM_001039590 NM_001039591	0.214	2.42	0.0176	1.00
merck2-AF379635_s_at	---	---	-0.105	-2.42	0.0177	1.00
merck-NM_015257_at	TMEM194A	NM_001130963 NM_015257	0.181	2.42	0.0177	1.00
merck-NM_032505_at	KBTBD8	NM_032505	0.245	2.41	0.0177	1.00
merck2-DA829519_a_at	ZC3H13	NM_015070	-0.191	-2.41	0.0178	1.00
merck-NM_007362_at	NCBP2	NM_007362 NM_001042540	0.175	2.41	0.0178	1.00
merck2-NM_139290_at	ANGPT1	BC029406	-0.147	-2.41	0.0178	1.00
merck-ENST00000361688_at	---	---	-0.132	-2.41	0.0179	1.00
merck-ENST00000357972_at	SYTL5	NM_138780 NM_001163335 NM_00116	0.253	2.41	0.0179	1.00
merck-AK098425_s_at	---	AK098425	0.169	2.41	0.0179	1.00
merck2-NM_018555_at	ZNF331	NM_018555 NM_001079906 NM_00107	0.171	2.41	0.0179	1.00
merck2-BG566231_at	---	---	-0.118	-2.41	0.0179	1.00
merck-AK055048_at	---	AK055048	0.271	2.41	0.0179	1.00
merck-NM_002002_at	FCER2	NM_002002	0.296	2.41	0.0179	1.00
merck-NM_019555_at	ARHGEF3	NM_001128615 NM_001128616 NM_01	0.174	2.41	0.0180	1.00
merck-BC062745_at	---	BC062745	0.447	2.41	0.0180	1.00
merck-AY358864_at	GPN2	AY358864	-0.135	-2.41	0.0180	1.00
merck-NM_001014979_at	C16orf93	NM_001014979	-0.197	-2.41	0.0180	1.00
merck-AW611782_at	---	---	0.300	2.41	0.0180	1.00
merck-Z22965_s_at	---	M90482	0.232	2.41	0.0180	1.00
merck-NM_032918_at	RERG	NM_032918 NM_001190726	0.105	2.41	0.0180	1.00
merck-NM_007148_at	RNF112	NM_007148	-0.146	-2.41	0.0180	1.00
merck2-AK054757_at	TBCD	AK129619	-0.172	-2.41	0.0180	1.00
merck-BM805635_at	---	---	0.112	2.41	0.0180	1.00
merck-NC_001526_ORF_1178_at	---	---	-0.0964	-2.41	0.0180	1.00
merck-NM_002003_at	FCN1	NM_002003	-0.151	-2.41	0.0180	1.00
merck-NM_006669_s_at	LILRB1	NM_006669 NM_001081637 NM_00108	-0.186	-2.41	0.0181	1.00
merck-NM_001882_at	CRHBP	NM_001882	-0.170	-2.41	0.0181	1.00
merck-AA773342_x_at	---	---	-0.138	-2.41	0.0181	1.00
merck-NM_000216_at	KAL1	NM_000216	-0.275	-2.40	0.0182	1.00
merck-BC016940_s_at	---	---	0.300	2.40	0.0182	1.00
merck2-CA748516_at	FGD4	NM_139241	-0.185	-2.40	0.0182	1.00
merck-NM_001037232_a_at	ZNF829	NM_001171979 NM_001037232	-0.100	-2.40	0.0183	1.00
merck-AF527833_a_at	TTTY21 TTTY21B	NR_001535 NR_003588	-0.161	-2.40	0.0183	1.00
merck-AK123814_s_at	POM121 POM121C	NM_172020 NM_001099415	0.143	2.40	0.0183	1.00

merck-NM_024820_a_at	DENND1A	NM_020946 NM_024820	-0.173	-2.40	0.0183	1.00
merck2-BX349325_at	PRR11	NM_018304	-0.181	-2.40	0.0184	1.00
merck2-KSHV_ORF4_at	---	---	-0.117	-2.40	0.0184	1.00
merck2-BU174390_a_at	SCD	NM_005063	-0.140	-2.40	0.0184	1.00
merck-ENST00000254908_s_at	PCBD2	NM_032151	-0.161	-2.40	0.0184	1.00
merck-AK127697_at	---	AK127697	0.353	2.40	0.0184	1.00
merck-NM_182980_at	OSGIN1	NM_013370 NM_182980 NM_182981	-0.160	-2.40	0.0184	1.00
merck-AI863735_at	---	AY534685	-0.158	-2.40	0.0184	1.00
merck-NM_001017418_s_at	SPRR2B	NM_001017418	0.0899	2.40	0.0184	1.00
merck2-NM_178125_at	TRIM50	NM_178125	-0.133	-2.40	0.0184	1.00
merck-NM_004701_at	CCNB2	NM_004701	-0.312	-2.40	0.0185	1.00
merck-AK055765_at	ODZ1	NM_001163278 NM_001163279 NM_01	0.182	2.40	0.0185	1.00
merck-NM_001033081_s_at	MYCL1	NM_001033081 NM_001033082	-0.182	-2.40	0.0186	1.00
merck-AK057810_at	---	AK057810	-0.165	-2.40	0.0186	1.00
merck2-BP431382_at	MPO	NM_000250	-0.441	-2.40	0.0186	1.00
merck-NM_003446_at	---	---	0.198	2.39	0.0186	1.00
merck-VZV_OKA_ORF9_s_at	---	---	-0.133	-2.39	0.0186	1.00
merck-AK024877_at	---	AK024877	-0.229	-2.39	0.0186	1.00
merck2-BX641171_at	---	BX641171	0.344	2.39	0.0187	1.00
merck2-BI199293_a_at	KIAA0226	NM_001145642 NM_014687	-0.123	-2.39	0.0187	1.00
merck2-AK225719_at	MAP2K6	AK225719	-0.157	-2.39	0.0188	1.00
merck-NM_080669_a_at	SLC46A1	NM_080669	-0.155	-2.39	0.0188	1.00
merck-NC_001357_ORF_1185_at	---	---	-0.104	-2.39	0.0188	1.00
merck-NM_001040034_at	CD63	NM_001780 NM_001040034	-0.113	-2.39	0.0188	1.00
merck-NM_174902_a_at	LDLRAD3	NM_174902	-0.147	-2.39	0.0189	1.00
merck-NM_133478_a_at	SLC4A5	NM_021196 NM_133478	0.156	2.39	0.0189	1.00
merck2-BC005395_at	HPX	NM_000613	-0.147	-2.39	0.0190	1.00
merck-AL833666_at	---	AL833666	-0.296	-2.39	0.0190	1.00
merck-XM_939116_a_at	CXorf48	NM_001031705 NM_017863	0.336	2.39	0.0191	1.00
merck-XM_944971_at	---	AC068642	-0.127	-2.38	0.0192	1.00
merck2-D26070_at	ITPR1	NM_001099952 NM_002222 NM_0011€	0.178	2.38	0.0192	1.00
merck-AK095619_s_at	---	---	-0.262	-2.38	0.0192	1.00
merck2-BG035799_at	CAD	NM_004341	-0.168	-2.38	0.0192	1.00
merck-AK098043_s_at	---	AK098043	-0.204	-2.38	0.0192	1.00
merck2-AK091896_at	MFSD4	NM_181644	0.168	2.38	0.0192	1.00
merck2-NM_174901_at	FAM9C	NM_174901	0.312	2.38	0.0193	1.00
merck-NM_006227_at	PLTP	NM_006227 NM_182676	-0.203	-2.38	0.0193	1.00
merck-AF086376_at	---	AF086376	0.188	2.38	0.0193	1.00
merck2-BC072452_a_at	FAM107B	NM_031453	0.176	2.38	0.0193	1.00
merck-AV655754_at	---	DQ314888	-0.172	-2.38	0.0194	1.00
merck-NM_017856_at	GEMIN8	NM_001042480 NM_001042479 NM_0'	0.202	2.38	0.0194	1.00
merck-NM_181780_at	BTLA	NM_181780 NM_001085357	0.266	2.38	0.0194	1.00
merck-NM_001468_at	GAGE1	NM_001468 NM_001040663	0.215	2.38	0.0194	1.00
merck-BM973820_s_at	ODF3B	NM_001014440	-0.245	-2.38	0.0194	1.00
merck-NM_001004686_at	OR2L2	NM_001004686	0.123	2.38	0.0194	1.00
merck-DB317658_at	---	---	0.398	2.38	0.0194	1.00
merck-BX422512_a_at	BRD7	NM_001173984 NM_013263	-0.168	-2.38	0.0195	1.00
merck2-CU013091_s_at	---	---	-0.297	-2.38	0.0196	1.00
merck2-U69611_at	ADAM17	NM_003183	-0.207	-2.38	0.0196	1.00
merck-BQ325657_at	---	AF017104	-0.209	-2.37	0.0196	1.00
merck-XM_939413_x_at	---	AL049641	0.600	2.37	0.0196	1.00
merck2-BE855602_at	OR2A9P	NR_002157	0.294	2.37	0.0197	1.00
merck-NM_017848_a_at	FAM120C	NM_017848	0.217	2.37	0.0197	1.00
merck-AI345952_at	---	---	0.208	2.37	0.0197	1.00
merck-AK092710_a_at	EPT1	NM_033505	0.169	2.37	0.0198	1.00
merck-BC050368_s_at	MMP19	NM_002429	-0.176	-2.37	0.0198	1.00
merck-ENST00000334139_at	---	NG_005176	-0.149	-2.37	0.0198	1.00
merck2-AI283400_at	SPTBN1	NM_178313	0.138	2.37	0.0199	1.00
merck-NM_052873_at	IFT43	NM_052873	0.155	2.37	0.0199	1.00
merck-BC028005_at	GRASPOS	BC028005	-0.179	-2.37	0.0199	1.00
merck-AF086096_x_at	FAM102A	AL049365	0.170	2.37	0.0199	1.00
merck-XM_069912_at	---	AC005482	-0.116	-2.37	0.0199	1.00
merck-BG755321_x_at	HLA-DRB1 HLA-DRB3	NM_002124 NM_022555 XM_00312051	0.414	2.37	0.0199	1.00
merck2-NM_152547_at	BTNL9	NM_152547	0.243	2.37	0.0199	1.00
merck-XM_928469_s_at	---	AC006476	-0.162	-2.37	0.0199	1.00
merck-NM_181802_at	UBE2C	NM_007019 NM_181799 NM_181800 N	-0.249	-2.37	0.0199	1.00
merck-NM_032838_s_at	ZNF566	NM_001145345 NM_001145344 NM_03	0.214	2.37	0.0200	1.00
merck-XM_940379_at	---	---	-0.161	-2.37	0.0200	1.00
merck-NM_001783_at	CD79A	NM_001783 NM_021601	0.380	2.37	0.0200	1.00
merck-ENST00000330288_a_at	CXorf69	NM_001163438	0.513	2.37	0.0200	1.00
merck2-BX647088_at	SRRM2	NM_016333	-0.250	-2.37	0.0201	1.00
merck-U16861_a_at	KCNJ2	NM_000891	-0.271	-2.37	0.0201	1.00
merck-ENST00000381635_at	---	---	-0.281	-2.36	0.0202	1.00
merck2-AI342698_at	---	AL109811	-0.171	-2.36	0.0203	1.00
merck-BG028539_at	---	---	-0.143	-2.36	0.0203	1.00
merck-NM_007125_at	UTY	NM_007125	-1.09	-2.36	0.0203	1.00
merck-ENST00000296564_at	KIAA0947	NM_015325	0.152	2.36	0.0203	1.00
merck2-AF262405_at	CASK	NM_003688 NM_001126054 NM_00112	0.235	2.36	0.0204	1.00
merck-BC071813_s_at	FLJ46906	NR_033896	-0.209	-2.36	0.0204	1.00
merck-CB851948_a_at	WDR52	NM_001164496 NM_018338	0.219	2.36	0.0204	1.00
merck2-BT019764_at	LDHB	NM_002300 NM_001174097	0.145	2.36	0.0204	1.00
merck-NM_014265_at	ADAM28	NM_014265	0.383	2.36	0.0204	1.00
merck-NM_014694_at	ADAMTSL2	NM_014694 NM_001145320	-0.187	-2.36	0.0205	1.00
merck2-DB444113_at	---	---	-0.151	-2.36	0.0205	1.00
merck-NM_012415_at	RAD54B	NM_012415	0.279	2.36	0.0205	1.00
merck2-AB208968_at	CDC42BPA	NM_014826 NM_003607	-0.137	-2.36	0.0205	1.00
merck2-VZV_OKA_ORF8_at	---	NC_001348	-0.113	-2.36	0.0205	1.00
merck2-AL515981_at	NCLN	NM_020170	-0.200	-2.36	0.0205	1.00
merck-hCT13097.2_at	---	NG_010976	-0.138	-2.36	0.0206	1.00
merck2-BX112120_at	USP51	NM_201286	0.180	2.36	0.0206	1.00
merck-NM_001039725_a_at	PNPLA1	NM_173676 NM_001145716 NM_00114	-0.184	-2.36	0.0206	1.00
merck-NM_205849_s_at	FAM9B	NM_205849	0.384	2.36	0.0206	1.00
merck-NM_080817_at	GPR82	NM_080817	0.218	2.35	0.0206	1.00
merck2-R50740_at	---	---	0.109	2.35	0.0207	1.00
merck2-NM_012307_at	EPB41L3	NM_012307	-0.168	-2.35	0.0207	1.00
merck-NM_173695_at	CXorf59	NM_173695	0.341	2.35	0.0207	1.00
merck-NM_022351_at	NECAB1	NM_022351	-0.188	-2.35	0.0207	1.00
merck-DR001278_at	---	---	-0.391	-2.35	0.0207	1.00
merck2-NM_004504_at	AGFG1	NM_001135187 NM_004504 NM_00113	-0.182	-2.35	0.0207	1.00
merck2-VZV_OKA_O17_up_s_at	---	---	0.111	2.35	0.0207	1.00
merck2-CB529222_at	---	CR603455	0.256	2.35	0.0207	1.00
merck-BX104821_at	---	---	-0.125	-2.35	0.0207	1.00
merck-NM_001040075_s_at	---	---	0.325	2.35	0.0207	1.00
merck-ENST00000317577_s_at	PPAPDC1B	NM_001102559	0.368	2.35	0.0208	1.00
merck2-AK058103_at	ADRBK2	NM_005160	-0.199	-2.35	0.0208	1.00
merck-NM_032317_at	DNAJC30	NM_032317	0.242	2.35	0.0209	1.00
merck2-AA262548_at	PAWR	NM_002583	0.240	2.35	0.0209	1.00
merck-NM_012113_at	CA14	NM_012113	-0.115	-2.35	0.0209	1.00
merck-NM_078483_at	SLC36A1	NM_078483	-0.248	-2.35	0.0210	1.00
merck-BC092466_a_at	TTC3	NM_003316 NM_001001894	0.126	2.35	0.0210	1.00
merck-XM_371781_x_at	---	---	0.0683	2.35	0.0210	1.00
merck2-NM_182827_at	FKBP9 FKBP9L	NM_007270 NR_027339 NR_027340 N	-0.213	-2.35	0.0210	1.00
merck-NM_145284_at	FAM122B	NM_145284 NM_001166599 NM_0011€	0.252	2.35	0.0211	1.00
merck2-BM919836_at	LEF1	NM_016269 NM_001130713 NM_00113	0.158	2.35	0.0211	1.00
merck-XM_065755_at	---	---	-0.117	-2.35	0.0211	1.00
merck2-CN343947_at	ZNF711	NM_021998	0.284	2.35	0.0211	1.00
merck-NM_024330_at	SLC27A3	NM_024330	-0.213	-2.35	0.0211	1.00
merck2-NM_138389_at	FAM114A1	NM_138389 NR_033290	-0.141	-2.35	0.0211	1.00
merck-NM_173463_at	CCDC149	AL834257	-0.162	-2.35	0.0212	1.00
merck-NM_004397_at	DDX6	NM_004397	-0.191	-2.34	0.0212	1.00
merck2-DQ892007_s_at	---	---	0.314	2.34	0.0212	1.00
merck-NM_012108_at	STAP1	NM_012108	0.375	2.34	0.0212	1.00
merck-DA736753_a_at	PRRC2C	NM_015172	-0.260	-2.34	0.0213	1.00
merck-XM_928495_at	---	CR610555	-0.101	-2.34	0.0213	1.00
merck-NM_001004342_at	TRIM67	NM_001004342	0.156	2.34	0.0213	1.00
merck-R07431_s_at	---	---	-0.231	-2.34	0.0214	1.00
merck2-CF121504_a_at	C9orf86	NM_024718 NM_001173988 NM_00117	-0.186	-2.34	0.0214	1.00
merck-AK024080_a_at	TOP2A	NM_001067	-0.251	-2.34	0.0214	1.00
merck-NM_003813_at	ADAM21	NM_003813	0.240	2.34	0.0214	1.00

merck-hCT1970811_at	---	AC114284	-0.122	-2.34	0.0214	1.00
merck-NM_005859_at	PURA	NM_005859	0.178	2.34	0.0214	1.00
merck-BC035518_s_at	---	AL049423	0.117	2.34	0.0214	1.00
merck2-AK056270_at	ST8SIA5	NM_013305	-0.146	-2.34	0.0214	1.00
merck-NM_033101_s_at	LGALS12	NM_001142535 NM_033101 NM_00114	-0.303	-2.34	0.0215	1.00
merck2-H12280_at	---	NG_005636	0.208	2.34	0.0215	1.00
merck2-AA776845_at	---	---	-0.144	-2.34	0.0215	1.00
merck-NM_016139_x_at	CHCHD2	NM_016139	-0.102	-2.34	0.0215	1.00
merck-NM_012225_at	NUBP2	NM_012225	-0.167	-2.34	0.0216	1.00
merck-U23849_s_at	APP	NM_000484 NM_001204303 NM_2014	-0.157	-2.34	0.0216	1.00
merck-AK094945_at	LOC285500	AK094945	0.146	2.34	0.0216	1.00
merck2-DT217746_at	ASRGL1	NM_001083926 NM_025080	-0.233	-2.34	0.0216	1.00
merck-NM_000950_at	PRRG1	NM_000950 NM_001142395 NM_00117	0.326	2.34	0.0216	1.00
merck2-ENST00000390537_s_at	---	---	0.145	2.34	0.0216	1.00
merck2-U72763_at	TNFRSF25	NM_148965 NM_003790 NM_148966 N	0.176	2.34	0.0217	1.00
merck2-ENST00000379900_x_at	---	---	0.395	2.34	0.0217	1.00
merck2-NM_012412_at	H2AFV	NM_012412 NM_201436 NM_201516 N	-0.123	-2.34	0.0217	1.00
merck-NM_002731_at	PRKACB	NM_182948 NM_002731	0.201	2.34	0.0217	1.00
merck2-BU175285_at	PTGES3	NM_006601	0.202	2.34	0.0217	1.00
merck2-AA514227_at	---	CR616033	-0.159	-2.34	0.0217	1.00
merck2-BG115561_at	ST6GALNAC4	NM_175039	-0.141	-2.33	0.0217	1.00
merck-ENST00000377163_at	---	AF399496	-0.128	-2.33	0.0217	1.00
merck2-NM_000254_a_at	MTR	NM_000254	0.130	2.33	0.0218	1.00
merck-AL137603_at	ZFY	NM_003411 NM_001145275 NM_00114	-0.678	-2.33	0.0218	1.00
merck-ENST00000327506_at	HEATR7B1	XM_001721240 XM_291007 XM_93970	0.124	2.33	0.0218	1.00
merck-NM_005262_at	GFER	NM_005262	-0.167	-2.33	0.0218	1.00
merck-NM_019020_s_at	TBC1D16	NM_019020	-0.140	-2.33	0.0219	1.00
merck2-BQ925373_x_at	YWHAQ	NM_006826	0.157	2.33	0.0219	1.00
merck-NM_052902_at	STK11IP	NM_052902	-0.217	-2.33	0.0219	1.00
merck-NM_001832_at	CLPS	NM_001832	-0.114	-2.33	0.0219	1.00
merck-AK128185_at	LOC399715	AK128185	-0.194	-2.33	0.0220	1.00
merck2-ENST00000380631_a_at	SH3BGR	NM_007341 NM_001001713	0.173	2.33	0.0220	1.00
merck2-X59739_at	ZFX	NM_003410 NM_001178084 NM_00117	0.249	2.33	0.0220	1.00
merck-NM_019903_at	ADD3	NM_016824 NM_019903 NM_001121	0.218	2.33	0.0221	1.00
merck2-AK123210_at	LOC100505759	XR_110332 XR_111324 XR_114603	0.207	2.33	0.0221	1.00
merck2-NM_198480_at	ZNF615	NM_001199324 NM_198480	0.166	2.33	0.0222	1.00
merck-XM_936762_at	---	NG_021571	-0.133	-2.33	0.0222	1.00
merck-BX089627_at	---	---	0.230	2.33	0.0222	1.00
merck-AL080280_s_at	---	AL080281	-0.239	-2.32	0.0223	1.00
merck-AK022479_at	---	AK022479	0.216	2.32	0.0223	1.00
merck-CR608093_s_at	YWHAE	NM_006761 NR_024058	-0.0586	-2.32	0.0223	1.00
merck-NM_002463_at	MX2	NM_002463	-0.171	-2.32	0.0223	1.00
merck-NM_003400_at	XPO1	NM_003400	0.181	2.32	0.0223	1.00
merck2-NM_001042370_at	TROVE2	NM_004600 NM_001173524 NR_03339	-0.128	-2.32	0.0223	1.00
merck-ENST00000368578_at	LOC399829	XR_109033 XR_111134 XR_114445	-0.114	-2.32	0.0223	1.00
merck2-AL136587_at	AGPAT5	NM_018361	0.211	2.32	0.0224	1.00
merck-AK023967_at	---	AK023967	-0.216	-2.32	0.0224	1.00
merck-BX100544_at	---	AC009068	0.206	2.32	0.0224	1.00
merck2-NM_144702_at	LRRC71	NM_144702	-0.152	-2.32	0.0225	1.00
merck2-BC019655_at	SNX22	NM_024798	0.302	2.32	0.0225	1.00
merck-BG197227_at	---	NG_008727	-0.146	-2.32	0.0225	1.00
merck-ENST00000298211_at	---	NG_022167	-0.209	-2.32	0.0225	1.00
merck-NM_032566_at	SPINK7	NM_032566	-0.119	-2.32	0.0225	1.00
merck-AA897420_at	---	---	-0.187	-2.32	0.0226	1.00
merck2-BX385129_at	LARGE	NM_004737 NM_133642	0.144	2.32	0.0226	1.00
merck2-NM_145060_a_at	SKA1	NM_001039535 NM_145060	-0.154	-2.32	0.0227	1.00
merck2-BG503223_at	---	---	-0.0972	-2.32	0.0227	1.00
merck-R02328_s_at	---	---	0.315	2.32	0.0227	1.00
merck2-NM_001080851_s_at	---	---	0.214	2.32	0.0227	1.00
merck2-AL548534_a_at	C19orf12	NM_001031726 NM_031448	0.144	2.32	0.0227	1.00
merck-AW977000_at	---	NG_012834	0.354	2.32	0.0227	1.00
merck-NM_001716_at	CXCR5	NM_001716 NM_032966	0.338	2.32	0.0227	1.00
merck2-BG695979_at	---	AL034582	0.221	2.32	0.0227	1.00
merck-NM_005532_at	IFI27	NM_001130080 NM_005532	-0.780	-2.32	0.0227	1.00
merck-CR608275_at	CBLL1	NM_024814 NR_024199	0.175	2.32	0.0227	1.00
merck-BC029962_s_at	SYT5	NM_003180	-0.131	-2.32	0.0228	1.00
merck-ENST00000339047_at	KIAA0825	NM_001145678	-0.261	-2.32	0.0228	1.00
merck2-DA947637_at	MED29	NM_017592	-0.203	-2.32	0.0228	1.00
merck2-M92449_a_at	NAAA	NM_014435 NM_001042402	-0.208	-2.32	0.0228	1.00
merck-AI282569_at	---	AP000274	0.200	2.32	0.0228	1.00
merck-NM_001009925_a_at	C20orf30	NM_001009923 NM_001009924 NM_0	0.124	2.31	0.0228	1.00
merck-AA243882_at	---	AC006375	-0.238	-2.31	0.0229	1.00
merck2-BM541598_at	PHLDA1	NM_007350	0.160	2.31	0.0229	1.00
merck2-NM_021871_at	FGA	NM_021871	0.130	2.31	0.0229	1.00
merck2-NM_001004196_at	CD200	NM_005944 NM_001004196	0.417	2.31	0.0229	1.00
merck-NM_019045_at	WDR44	NM_019045 NM_001184965 NM_00118	0.169	2.31	0.0229	1.00
merck-NM_019106_at	SEPT3	NM_145733 NM_019106	-0.139	-2.31	0.0230	1.00
merck-AK021556_at	---	AK021556	0.173	2.31	0.0230	1.00
merck2-AF000992_a_at	KDM6A	NM_021140	0.299	2.31	0.0230	1.00
merck-XM_932757_at	---	---	-0.143	-2.31	0.0230	1.00
merck-NM_012290_at	TLK1	NM_012290 NM_001136554 NM_00113	0.182	2.31	0.0230	1.00
merck2-NM_001002275_at	FCGR2B	NM_004001 NM_001002273 NM_0010	0.289	2.31	0.0230	1.00
merck-NM_012069_at	ATP1B4	NM_001142447 NM_012069	0.339	2.31	0.0230	1.00
merck-NM_014294_at	TRAM1	NM_014294	0.172	2.31	0.0231	1.00
merck-NM_021643_at	TRIB2	NM_021643 NR_027303	0.165	2.31	0.0231	1.00
merck2-AL049423_at	---	AL049423	0.136	2.31	0.0231	1.00
merck-BC001459_s_at	RAD51	NM_002875 NM_133487 NM_00116427	-0.162	-2.31	0.0232	1.00
merck-NM_003943_at	STBD1	NM_003943	-0.190	-2.31	0.0232	1.00
merck2-CMV_UL89_at	---	---	-0.103	-2.31	0.0232	1.00
merck-NM_001040070_x_at	---	---	0.346	2.31	0.0232	1.00
merck-BX647350_at	ITGA9	BX647350	-0.265	-2.31	0.0232	1.00
merck-AF019617_at	PAR4	NR_022010	-0.158	-2.31	0.0233	1.00
merck2-AB006651_at	MED14	AF304448	0.188	2.31	0.0233	1.00
merck2-BC064028_at	---	---	-0.132	-2.31	0.0233	1.00
merck2-NM_199440_s_at	HSPD1	NM_002156 NM_199440	0.104	2.31	0.0233	1.00
merck2-NM_020406_at	CD177	NM_020406	-0.367	-2.31	0.0233	1.00
merck-NM_174937_at	TCERG1L	NM_174937	-0.117	-2.31	0.0233	1.00
merck-AW104394_at	---	AL359390	0.196	2.31	0.0234	1.00
merck-BC050658_a_at	ZC3H13	NM_015070	-0.164	-2.31	0.0234	1.00
merck-NM_174939_s_at	LINC00301	NR_026946	-0.140	-2.30	0.0234	1.00
merck-NM_018075_s_at	ANO10	NM_018075	-0.168	-2.30	0.0234	1.00
merck2-DB293898_a_at	BAZ2A	NM_013449	-0.292	-2.30	0.0235	1.00
merck-NM_138799_at	MBOAT2	NM_138799	-0.190	-2.30	0.0235	1.00
merck-NM_139266_at	STAT1	NM_139266	-0.246	-2.30	0.0235	1.00
merck-AK127065_a_at	ZBTB47	NM_145166	-0.222	-2.30	0.0235	1.00
merck-BC089428_a_at	PLEKHG1	NM_001029884	0.170	2.30	0.0236	1.00
merck-NM_080753_at	WFDC10A	NM_080753	0.139	2.30	0.0238	1.00
merck-NM_020726_a_at	NLN	NM_020726	-0.196	-2.30	0.0238	1.00
merck2-HSV1_ORF_UL50_at	---	---	-0.120	-2.30	0.0238	1.00
merck2-BG166466_at	PPP6C	NM_001123355 NM_002721 NM_00112	0.114	2.30	0.0239	1.00
merck-NM_000487_a_at	ARSA	NM_000487 NM_001085425 NM_00108	-0.227	-2.30	0.0239	1.00
merck2-NM_172236_at	POFUT1	NM_172236	0.124	2.30	0.0239	1.00
merck-NM_001005182_at	OR6C1	NM_001005182	-0.127	-2.30	0.0239	1.00
merck2-BG680395_at	---	---	0.114	2.30	0.0239	1.00
merck-NM_021047_s_at	ZNF253	NM_021047	0.246	2.30	0.0239	1.00
merck-NM_138346_s_at	KIAA2013	NM_138346	-0.137	-2.30	0.0239	1.00
merck-AL080200_at	C8orf71	NR_026772	-0.150	-2.30	0.0240	1.00
merck-BC041884_at	RFTN1	BC041884	-0.196	-2.30	0.0240	1.00
merck2-BM679668_at	---	---	0.225	2.30	0.0240	1.00
merck-NM_003495_at	HIST1H4I	NM_003495	-0.135	-2.29	0.0240	1.00
merck-CR613437_at	---	CR613437	0.225	2.29	0.0240	1.00
merck-BC037904_at	MGC42157	BC037904	-0.118	-2.29	0.0240	1.00
merck-AK098298_at	---	XR_109469 XR_111770 XR_115168	0.184	2.29	0.0241	1.00
merck-NM_178865_at	SERINC2	NM_178865 NM_018565 NM_0011990	-0.188	-2.29	0.0241	1.00
merck-ENST00000341231_at	---	AF399360	-0.173	-2.29	0.0242	1.00
merck-NM_153234_at	LIX1	NM_153234	0.253	2.29	0.0242	1.00
merck-NM_001346_at	DGKG	NM_001346 NM_001080744 NM_00108	-0.233	-2.29	0.0242	1.00
merck2-BX647250_at	BAZ2B	BX647250	-0.180	-2.29	0.0242	1.00
merck2-BI461234_s_at	APLP2	NM_001642 NM_001142276 NM_00114	-0.270	-2.29	0.0242	1.00

merck-NM_015441_at	OLFML2B	NM_015441	-0.161	-2.29	0.0242	1.00
merck-NM_174907_a_at	PPP4R2	NM_174907	-0.179	-2.29	0.0243	1.00
merck2-NM_001080538_x_at	AKR1B15	NM_001080538	-0.138	-2.29	0.0243	1.00
merck-AK027161_at	---	AK027161	0.162	2.29	0.0243	1.00
merck-BC010705_at	ZNF519	NM_145287	0.213	2.29	0.0243	1.00
merck-BC051743_a_at	ZFP91 ZFP91-CNTF	NM_053023 NM_001197051 NR_02409	-0.196	-2.29	0.0243	1.00
merck-AA399109_at	---	NG_013090	0.241	2.29	0.0243	1.00
merck-DB327110_at	---	AL121957	-0.0981	-2.29	0.0243	1.00
merck2-HSV1_ORF_UL22_at	---	---	-0.120	-2.29	0.0243	1.00
merck-BC042959_at	---	XR_109831 XR_112669 XR_113670 XF	-0.122	-2.29	0.0244	1.00
merck2-AK092450_s_at	---	AK092450	0.146	2.29	0.0244	1.00
merck-NM_001007249_at	OR8G2	NM_001007249	-0.0947	-2.29	0.0244	1.00
merck2-AL080179_at	FAM75C1 FAM75C2	NM_001145124 NM_001166137	-0.253	-2.29	0.0244	1.00
merck-BG482638_at	---	---	-0.0965	-2.29	0.0244	1.00
merck-NM_023034_at	WHSC1L1	NM_023034	-0.111	-2.29	0.0244	1.00
merck2-BC009851_at	IGHM	BC009851	0.356	2.29	0.0244	1.00
merck-NM_198337_at	INSIG1	NM_005542 NM_198336 NM_198337	0.146	2.29	0.0245	1.00
merck-hCT1822680_at	---	AC211782	-0.124	-2.29	0.0245	1.00
merck-NM_198576_s_at	AGRN	NM_198576	-0.277	-2.29	0.0245	1.00
merck2-DA833911_at	---	---	-0.226	-2.29	0.0245	1.00
merck-AK024684_at	---	AK024684	0.180	2.29	0.0245	1.00
merck-BX094453_a_at	LOC283143	NR_034148	-0.133	-2.29	0.0245	1.00
merck2-ENST00000331944_s_at	---	---	-0.165	-2.29	0.0246	1.00
merck-BM806633_at	---	---	-0.104	-2.28	0.0247	1.00
merck-NM_022368_at	PJA1	NM_145119 NM_001032396 NM_02236	0.154	2.28	0.0247	1.00
merck2-DQ535269_x_at	---	DQ535269	-0.162	-2.28	0.0247	1.00
merck-CB110908_x_at	MT2A	NM_005953	-0.263	-2.28	0.0247	1.00
merck2-BE144260_at	---	AY644470	-0.131	-2.28	0.0247	1.00
merck-NM_002417_at	MKI67	NM_002417 NM_001145966	-0.197	-2.28	0.0247	1.00
merck-NM_015589_s_at	SAMD4A	NM_015589 NM_001161576 NM_00116	-0.209	-2.28	0.0248	1.00
merck-NM_001006121_s_at	RBMY1A1 RBMY1B RBMY1D RBMY1E	NM_005058 NM_001006121 NM_0010(	-0.129	-2.28	0.0248	1.00
merck2-AA203536_at	CLN8	NM_018941	0.128	2.28	0.0248	1.00
merck-NM_003559_a_at	PIP4K2B	NM_003559	-0.161	-2.28	0.0248	1.00
merck2-DQ893675_x_at	RIN1	NM_004292	-0.190	-2.28	0.0248	1.00
merck-NM_022375_at	OCLM	NM_022375 NM_001199906	-0.182	-2.28	0.0248	1.00
merck-DB508953_a_at	ABCB11	NM_003742	0.133	2.28	0.0249	1.00
merck2-NM_020139_at	BDH2	NM_020139	0.229	2.28	0.0249	1.00
merck-AK125986_a_at	ATP11C	NM_173694 NM_001010986	0.206	2.28	0.0249	1.00
merck-BC111030_a_at	SPIRE2	NM_032451	-0.178	-2.28	0.0249	1.00
merck2-NM_198964_a_at	PTHLH	NM_002820 NM_198964	0.154	2.28	0.0249	1.00
merck-NM_006939_at	SOS2	NM_006939	-0.174	-2.28	0.0249	1.00
merck2-AK126360_at	---	AK126360	0.194	2.28	0.0250	1.00
merck-NM_016551_at	TM7SF3	NM_016551	-0.127	-2.28	0.0250	1.00
merck-AV690960_at	---	---	-0.120	-2.28	0.0250	1.00
merck-AK027787_a_at	SLC25A32	NM_030780	0.180	2.28	0.0250	1.00
merck-BM810405_at	---	---	-0.132	-2.28	0.0251	1.00
merck-BX111278_at	---	AL441885	-0.127	-2.28	0.0251	1.00
merck2-AI377384_at	---	---	-0.108	-2.28	0.0251	1.00
merck2-ENST00000355476_at	RBPJ	NM_005349 NM_015874 NM_203283 N	-0.0934	-2.28	0.0251	1.00
merck-NM_016446_a_at	TMEM8B	NM_001042590 NM_001042589 NM_0	-0.188	-2.28	0.0251	1.00
merck-NM_000880_at	IL7	NM_000880 NM_001199886 NM_0011	0.180	2.28	0.0251	1.00
merck2-NM_001036645_x_at	HHLA3	NM_001036645	0.112	2.28	0.0251	1.00
merck-NM_139214_s_at	TGIF2LX TGIF2LY	NM_138960 NM_139214	-0.0922	-2.28	0.0252	1.00
merck-BC034299_at	---	BC034299	-0.147	-2.28	0.0252	1.00
merck-BC029907_s_at	---	BC029907	0.234	2.28	0.0252	1.00
merck-AK091312_at	---	AK091312	0.189	2.27	0.0253	1.00
merck-NM_023940_at	RASL11B	NM_023940	0.210	2.27	0.0253	1.00
merck-AK056037_at	LOC100272217	NR_027440	-0.161	-2.27	0.0254	1.00
merck-XM_929289_at	---	AC214986	-0.136	-2.27	0.0254	1.00
merck2-BF667260_at	MTRR	NM_002454 NM_024010	0.219	2.27	0.0254	1.00
merck-NM_002015_at	FOXO1	NM_002015	0.142	2.27	0.0254	1.00
merck2-CB999623_at	MLLT3	NM_004529	0.175	2.27	0.0254	1.00
merck-BX647248_at	TKT	NM_001135055	-0.281	-2.27	0.0255	1.00
merck-BC064148_x_at	FAM95B1	NR_026759	-0.163	-2.27	0.0255	1.00
merck2-NM_194441_at	BTN3A1	NM_007048 NM_194441 NM_0011450(	-0.223	-2.27	0.0255	1.00
merck-NM_001037125_at	UNKL	NM_001037125	-0.185	-2.27	0.0256	1.00
merck-NM_001039876_at	C19orf46	NM_001039876	-0.153	-2.27	0.0256	1.00
merck-XM_929880_s_at	TLK2	NM_006852 NM_001112707 XR_11074:	-0.202	-2.27	0.0257	1.00
merck-NM_153446_at	B4GALNT2	NM_153446 NM_001159387 NM_0011	-0.154	-2.27	0.0257	1.00
merck-AA203478_at	---	---	-0.121	-2.27	0.0257	1.00
merck-NM_005101_at	ISG15	NM_005101	-0.426	-2.27	0.0258	1.00
merck2-Z22968_at	CD163	NM_004244 NM_203416	-0.193	-2.27	0.0258	1.00
merck-NM_133460_at	ZNF418	NM_133460	0.198	2.27	0.0258	1.00
merck-AK024263_at	SLC38A1	NM_030674 NM_001077484	0.232	2.26	0.0259	1.00
merck2-BU070752_a_at	OCIAD1	NM_017830 NM_001079839 NM_0010	0.125	2.26	0.0259	1.00
merck-BC032433_a_at	ATF7	NM_001130059 NM_006856 NM_0011	-0.177	-2.26	0.0259	1.00
merck-NM_015093_at	TAB2	NM_015093	0.168	2.26	0.0259	1.00
merck-R12375_s_at	---	AC016654	0.161	2.26	0.0259	1.00
merck-NM_002855_at	PVRL1	NM_002855	-0.181	-2.26	0.0259	1.00
merck2-CK823615_a_at	FBXL16	NM_153350	0.171	2.26	0.0260	1.00
merck2-ENST00000375401_at	KDM5C	NM_004187 NM_001146702	0.254	2.26	0.0261	1.00
merck2-BC028370_at	---	---	-0.288	-2.26	0.0261	1.00
merck-NM_199171_at	PMEPA1	NM_020182 NM_199169 NM_199170 N	0.256	2.26	0.0261	1.00
merck-NM_007052_at	NOX1	NM_007052 NM_013955	0.226	2.26	0.0261	1.00
merck-BC019906_s_at	EIF2S3	NM_001415	0.105	2.26	0.0261	1.00
merck-BQ575676_at	---	NG_005422	-0.164	-2.26	0.0261	1.00
merck-NM_153183_at	NUDT10	NM_153183	0.201	2.26	0.0262	1.00
merck2-NM_001076675_at	ZNF626	NM_001076675	0.201	2.26	0.0262	1.00
merck-AK128649_at	IGHD	AK090461	0.391	2.26	0.0262	1.00
merck-BM977434_a_at	ABLIM1	NM_002313 NM_001003407 NM_0010(	0.161	2.26	0.0263	1.00
merck-AK092628_a_at	CALML4	NM_033429 NM_001031733	-0.204	-2.26	0.0263	1.00
merck2-AL134313_x_at	---	---	-0.118	-2.26	0.0263	1.00
merck-BM151384_at	---	---	-0.157	-2.26	0.0264	1.00
merck2-NM_181335_s_at	---	---	-0.191	-2.26	0.0264	1.00
merck-XM_377529_at	---	NG_021764	-0.110	-2.26	0.0264	1.00
merck2-NM_001007526_s_at	---	---	-0.101	-2.26	0.0265	1.00
merck2-AK057887_at	---	AK057887	0.161	2.25	0.0266	1.00
merck-BF170817_at	---	---	0.147	2.25	0.0266	1.00
merck-NM_000983_at	RPL22	NM_000983	0.117	2.25	0.0266	1.00
merck-BF373138_at	---	---	-0.0933	-2.25	0.0266	1.00
merck-AK093292_at	LOC283501	AK093292	-0.130	-2.25	0.0266	1.00
merck-NM_139179_at	DAGLB	NM_139179 NM_001142936	-0.242	-2.25	0.0266	1.00
merck-BC035064_at	---	BC035064	-0.123	-2.25	0.0266	1.00
merck-NM_031297_s_at	RNF208	NM_031297	-0.144	-2.25	0.0266	1.00
merck2-DQ890613_a_at	CHN1	NM_001822 NM_001025201	0.202	2.25	0.0266	1.00
merck-ENST00000326638_at	NKAPP1	NR_027131	0.140	2.25	0.0267	1.00
merck-NM_004672_at	MAP3K6	NM_004672	-0.182	-2.25	0.0267	1.00
merck-NM_003538_at	HIST1H4A	NM_003538	-0.157	-2.25	0.0267	1.00
merck-BM982256_at	---	---	-0.133	-2.25	0.0267	1.00
merck-AI637893_at	---	AL139120	-0.152	-2.25	0.0267	1.00
merck-NM_001001732_at	CC2D2B	NM_001159747 NM_001001732	-0.136	-2.25	0.0268	1.00
merck-BC030635_at	LOC100291323	BC030635	0.405	2.25	0.0268	1.00
merck-DQ323013_x_at	---	---	0.120	2.25	0.0269	1.00
merck2-AK055020_at	NAP1L1	NM_139207 NM_004537	0.112	2.25	0.0269	1.00
merck2-CF131182_s_at	---	---	0.151	2.25	0.0269	1.00
merck-ENST00000327581_a_at	NT5DC4	BC041437	-0.181	-2.25	0.0270	1.00
merck-NM_003202_at	TCF7	NM_003202 NM_201632 NM_0011348	0.161	2.25	0.0270	1.00
merck-NM_005480_at	TROAP	NM_005480	-0.152	-2.25	0.0271	1.00
merck-BC110370_at	ABCB5	NM_001163993	-0.0966	-2.25	0.0271	1.00
merck-CR595881_at	---	CR608336	-0.136	-2.25	0.0271	1.00
merck-AF116672_at	C8orf39	NR_027259	-0.140	-2.25	0.0271	1.00
merck-BC020734_a_at	HPGDS	NM_014485	-0.194	-2.25	0.0271	1.00
merck2-AY358900_at	COCH	NM_001135058 NM_004086	0.327	2.24	0.0272	1.00
merck-NM_006595_at	API5	NM_001142930 NM_006595 NM_00114	0.137	2.24	0.0272	1.00
merck2-AY345857_x_at	WWP1	NM_007013	0.120	2.24	0.0272	1.00
merck2-CR936605_at	ZNF667	NM_022103 NR_030740	0.119	2.24	0.0272	1.00
merck-XM_497480_at	---	---	0.158	2.24	0.0272	1.00
merck2-AJ270695_at	BHLHE41	NM_030762	0.240	2.24	0.0273	1.00
merck-U43677_s_at	FCAR	NM_002000 NM_133269 NM_133271 N	-0.205	-2.24	0.0273	1.00

merck2-NM_001042780_a_at	TNNT3	NM_006757 NM_001042781 NM_00104	-0.180	-2.24	0.0273	1.00
merck2-AF379633_x_at	---	---	-0.111	-2.24	0.0273	1.00
merck-NM_000266_at	NDP	NM_000266	0.268	2.24	0.0275	1.00
merck2-AK057931_at	RSPH3	NM_031924	-0.230	-2.24	0.0275	1.00
merck-XM_938074_at	LOC390760	XM_001715760 XM_001717472 XM_00	0.170	2.24	0.0275	1.00
merck2-NM_001018008_at	TPM1	NM_001018008	-0.173	-2.24	0.0275	1.00
merck-AK097071_s_at	---	---	0.334	2.24	0.0275	1.00
merck-NM_021572_a_at	ENPP5	NM_021572	0.187	2.24	0.0276	1.00
merck-AF093744_at	LOC100288675	AF093744	0.226	2.24	0.0277	1.00
merck2-NM_022739_at	SMURF2	NM_022739	0.170	2.24	0.0277	1.00
merck-CB242128_s_at	---	AC006287	0.146	2.24	0.0277	1.00
merck-NM_003643_at	GCM1	NM_003643	-0.252	-2.24	0.0277	1.00
merck-ENST00000328798_at	OK/SW-CL.36	AB064670	-0.204	-2.24	0.0277	1.00
merck2-NM_001039535_a_at	SKA1	NM_001039535 NM_145060	-0.185	-2.24	0.0277	1.00
merck-AL834381_a_at	BAZ2B	NM_013450	-0.159	-2.24	0.0277	1.00
merck-BQ023373_at	---	AL110203	0.172	2.24	0.0277	1.00
merck-BM552168_at	---	---	-0.131	-2.24	0.0277	1.00
merck2-BM677789_x_at	---	---	-0.123	-2.24	0.0278	1.00
merck-AF109362_a_at	SCD	NM_005063	-0.187	-2.24	0.0278	1.00
merck-NM_005958_at	MTNR1A	NM_005958	-0.127	-2.24	0.0278	1.00
merck-ENST00000355337_x_at	CTAGE4 CTAGE9 CTAGE15P CTAGE6	NM_198495 NM_001145659 NM_00100	0.154	2.24	0.0278	1.00
merck-ENST00000379900_x_at	---	XM_003120441	0.137	2.24	0.0278	1.00
merck-ENST00000344523_at	---	AL354979	-0.212	-2.24	0.0278	1.00
merck-NM_000773_at	CYP2E1	NM_000773	0.193	2.23	0.0278	1.00
merck-NM_133625_at	SYN2	NM_133625	-0.199	-2.23	0.0279	1.00
merck-NM_014792_at	KIAA0125	NR_026800	0.386	2.23	0.0279	1.00
merck-BE748225_at	---	---	0.147	2.23	0.0279	1.00
merck-CX752796_at	LOC253842	BC027988	-0.196	-2.23	0.0279	1.00
merck-NM_198486_at	RPL7L1	NM_198486	-0.181	-2.23	0.0280	1.00
merck-NM_054108_s_at	HRASLS5	NM_054108 NM_001146729 NM_00114	-0.134	-2.23	0.0280	1.00
merck-NC_001357_ORF_1184_at	---	---	-0.120	-2.23	0.0280	1.00
merck-AI762342_at	---	AL953870	0.297	2.23	0.0280	1.00
merck-NM_001231_at	CASQ1	NM_001231	-0.139	-2.23	0.0280	1.00
merck-NM_001004355_a_at	---	XR_109778 XR_113319 XR_115608	0.226	2.23	0.0281	1.00
merck-CR613436_s_at	---	CR613436	0.410	2.23	0.0281	1.00
merck-BX648739_at	C19orf50	BX648739	-0.112	-2.23	0.0281	1.00
merck-NM_013438_at	UBQLN1	NM_013438 NM_053067	0.151	2.23	0.0281	1.00
merck-NM_024764_at	CATSPERB	NM_024764	0.233	2.23	0.0281	1.00
merck2-BX648938_at	---	BX648938	-0.152	-2.23	0.0281	1.00
merck-ENST00000248668_at	---	---	-0.172	-2.23	0.0281	1.00
merck-NM_012470_a_at	TNPO3	NM_012470 NM_001191028 NR_03405	-0.161	-2.23	0.0281	1.00
merck-NM_015296_at	DOCK9	NM_015296 NM_001130048	0.263	2.23	0.0281	1.00
merck-hCT2345774_at	---	AC138657	0.137	2.23	0.0282	1.00
merck2-AF054589_at	MDFIC	NM_001166345 NM_199072	0.263	2.23	0.0282	1.00
merck2-BX104084_x_at	---	AC018688	-0.0865	-2.23	0.0282	1.00
merck-AY078405_s_at	ABCC6 ABCC6P2	NM_001079528 NR_023387	-0.222	-2.23	0.0282	1.00
merck-NM_012286_at	MORF4L2	NM_001142418 NM_001142426 NM_00	0.132	2.23	0.0283	1.00
merck2-AL117392_at	CGGBP1	NM_001008390 NM_003663 NM_0011	0.189	2.23	0.0283	1.00
merck-NM_207644_s_at	C22orf36	NM_207644	-0.169	-2.23	0.0284	1.00
merck-NM_030775_at	WNT5B	NM_032642 NM_030775	-0.179	-2.23	0.0284	1.00
merck-ENST00000274705_at	PCDHB18	NR_001281	0.183	2.23	0.0284	1.00
merck-BI829805_at	---	AL512410	0.110	2.23	0.0284	1.00
merck-NM_006850_s_at	IL24	NM_006850 NM_001185156 NM_00118	0.197	2.23	0.0284	1.00
merck-DB122906_a_at	PLEKHB1	NM_021200 NM_001130034 NM_00113	0.166	2.23	0.0284	1.00
merck-NM_139204_s_at	EPS8L1	NM_133180 NM_017729	-0.177	-2.23	0.0284	1.00
merck-NM_058174_s_at	COL6A2	NM_058175 NM_058174	-0.129	-2.23	0.0285	1.00
merck-BC026185_a_at	SCCPDH	NM_016002	-0.192	-2.23	0.0285	1.00
merck-XM_939741_at	---	---	-0.124	-2.23	0.0285	1.00
merck2-AI924182_at	---	---	-0.122	-2.22	0.0285	1.00
merck-NM_003141_at	TRIM21	NM_003141	-0.163	-2.22	0.0286	1.00
merck2-AY358811_at	FGF18	NM_003862	-0.148	-2.22	0.0286	1.00
merck-NM_003011_at	SET	NM_001122821 NM_003011	0.0985	2.22	0.0287	1.00
merck2-AY099107_at	SPICE1	NM_144718	0.133	2.22	0.0287	1.00
merck2-BX640705_at	ZNF44	BX640705	0.174	2.22	0.0287	1.00
merck-AA032155_at	LOC158572	NR_026742	0.182	2.22	0.0287	1.00
merck2-BC062720_at	HEATR5A	BC062720	-0.270	-2.22	0.0289	1.00
merck2-CD703280_at	---	---	0.435	2.22	0.0289	1.00
merck2-BG740130_at	---	---	0.122	2.22	0.0289	1.00
merck-AV692789_at	---	AC096642	0.266	2.22	0.0289	1.00
merck2-BM668558_at	SART1	NM_005146	-0.150	-2.22	0.0289	1.00
merck-AF125530_a_at	ARL6IP5	NM_006407	0.114	2.22	0.0289	1.00
merck2-BC110817_at	TXLNG2P	BC110817	-0.512	-2.22	0.0290	1.00
merck2-H17596_at	CLEC2D	NM_013269 NM_001004419 NM_0011	0.219	2.22	0.0290	1.00
merck-ENST00000302098_x_at	NBPF11 NBPF24 LOC100506032 KIAA	NM_183372 NM_001101663 XM_00311	0.109	2.22	0.0290	1.00
merck-NM_004106_at	FCER1G	NM_004106	-0.0930	-2.22	0.0291	1.00
merck-AJ297964_a_at	---	AJ297964	-0.205	-2.22	0.0291	1.00
merck-BC064407_s_at	FAM107B	NM_031453	0.0994	2.22	0.0291	1.00
merck-NM_002462_at	MX1	NM_001144925 NM_002462 NM_00117	-0.409	-2.22	0.0292	1.00
merck2-NM_198881_at	TBC1D8B	NM_198881	0.360	2.22	0.0292	1.00
merck-BX103488_at	---	---	-0.144	-2.21	0.0292	1.00
merck2-CD709967_x_at	HSPA8	NM_006597	0.168	2.21	0.0292	1.00
merck-NM_001039661_s_at	TIRAP	NM_001039661	-0.165	-2.21	0.0292	1.00
merck-NM_005241_a_at	MECOM	NM_001105077 NM_005241 NM_00110	0.192	2.21	0.0292	1.00
merck2-AJ717664_at	BTLA	NM_181780 NM_001085357	0.215	2.21	0.0292	1.00
merck-NM_015130_s_at	TBC1D9	NM_015130	0.215	2.21	0.0293	1.00
merck-NM_018593_s_at	SLC16A10	NM_018593	0.266	2.21	0.0293	1.00
merck-XM_067021_at	---	---	-0.124	-2.21	0.0293	1.00
merck-CR611332_s_at	PURA	NM_005859	0.236	2.21	0.0293	1.00
merck-BX647997_a_at	MTMR2	NM_016156 NM_201278 NM_201281 N	0.137	2.21	0.0294	1.00
merck-NM_002935_at	RNASE3	NM_002935	-0.491	-2.21	0.0294	1.00
merck-CX757198_at	---	---	-0.142	-2.21	0.0294	1.00
merck-DR000578_a_at	AGPAT4	NM_020133	-0.149	-2.21	0.0294	1.00
merck2-CX865413_x_at	LDHB	NM_002300 NM_001174097	0.157	2.21	0.0294	1.00
merck-AK023472_at	CCDC149	NM_173463 NM_001130726	-0.221	-2.21	0.0294	1.00
merck-AK022171_at	---	AK022171	-0.163	-2.21	0.0294	1.00
merck-BF374857_x_at	---	NG_021754	-0.134	-2.21	0.0295	1.00
merck2-BM310611_at	---	---	-0.107	-2.21	0.0295	1.00
merck-AW243584_at	---	AC005570	-0.163	-2.21	0.0296	1.00
merck-NM_005094_s_at	SLC27A4	NM_005094	-0.155	-2.21	0.0296	1.00
merck-ENST00000377260_a_at	CTNNBIP1	NM_020248 NM_001012329	-0.102	-2.21	0.0296	1.00
merck-BC065527_a_at	---	XR_110885	-0.149	-2.21	0.0296	1.00
merck-AU142077_at	TTLL3 ARPC4-TTLL3	NM_001025930 NM_001198793 NR_03	-0.170	-2.21	0.0297	1.00
merck2-AA812559_at	WBP2NL	AF393575	-0.145	-2.21	0.0297	1.00
merck-NM_020897_at	HCN3	NM_020897	-0.188	-2.21	0.0297	1.00
merck2-BC071865_at	LOXL3	NM_032603	-0.214	-2.21	0.0297	1.00
merck-ENST00000360119_a_at	NLGN1	NM_014932	0.186	2.21	0.0297	1.00
merck-DB312486_s_at	NEK3	AL833951	-0.203	-2.21	0.0298	1.00
merck2-AF059195_a_at	MAFG	NM_002359 NM_032711	-0.125	-2.21	0.0298	1.00
merck-X96661_s_at	SNORD61	NR_002735	0.180	2.21	0.0298	1.00
merck2-NM_013289_x_at	KIR3DL1	NM_013289	-0.417	-2.21	0.0298	1.00
merck-NM_153218_a_at	LACC1	NM_001128303 NM_153218	-0.175	-2.21	0.0298	1.00
merck-G65685_at	---	BX323860	-0.134	-2.21	0.0298	1.00
merck2-AF026941_a_at	RSAD2	NM_080657	-0.523	-2.21	0.0299	1.00
merck-AL359566_at	---	AL359566	0.391	2.21	0.0299	1.00
merck2-AB011175_at	TBC1D4	NM_014832	0.288	2.21	0.0299	1.00
merck-AK027107_at	---	AK027107	-0.153	-2.21	0.0299	1.00
merck2-CV420128_at	---	---	-0.196	-2.20	0.0299	1.00
merck2-NM_004019_at	DMD	NM_004019	0.305	2.20	0.0300	1.00
merck-ENST00000324446_a_at	NCRNA00185	NR_001544	-0.223	-2.20	0.0300	1.00
merck-XM_936725_at	---	---	-0.145	-2.20	0.0300	1.00
merck-NM_001024210_at	S100A13	NM_001024210 NM_005979 NM_00102	-0.204	-2.20	0.0300	1.00
merck2-AK125843_x_at	SLC6A8 SLC6A10P	NM_005629 NM_001142805 NM_00114	-0.312	-2.20	0.0301	1.00
merck-NM_144689_at	ZNF420	NM_144689	0.232	2.20	0.0301	1.00
merck-AK024021_a_at	C12orf23	NM_152261	0.232	2.20	0.0301	1.00
merck2-BQ379815_at	---	AK092386	-0.142	-2.20	0.0301	1.00
merck-ENST00000325558_s_at	PGA3 PGA4 PGA5	NM_001079807 NM_001079808 NM_0	-0.361	-2.20	0.0301	1.00
merck2-AB209527_s_at	HNRNPC	NM_031314 NM_004500 NM_0010774	0.0700	2.20	0.0301	1.00
merck2-NM_002608_a_at	PDGFB	NM_002608 NM_033016	0.143	2.20	0.0302	1.00
merck-NM_003441_at	ZNF141	L15309	0.252	2.20	0.0302	1.00

merck2-BU166338_at	HNRNPA0	NM_006805	0.138	2.20	0.0302	1.00
merck2-VZV_OKA_O38_up_at	---	---	-0.0928	-2.20	0.0302	1.00
merck2-BU169961_at	NUMA1	NM_006185	-0.241	-2.20	0.0302	1.00
merck2-NM_080657_at	RSAD2	NM_080657	-0.643	-2.20	0.0302	1.00
merck-NM_031481_at	SLC25A18	NM_031481	-0.146	-2.20	0.0302	1.00
merck2-ENST00000390243_at	---	AF490937	0.277	2.20	0.0302	1.00
merck-ENST00000314053_at	---	AF116673	0.155	2.20	0.0302	1.00
merck-AF244577_a_at	SLC17A5	NM_012434	-0.154	-2.20	0.0303	1.00
merck-BP271655_s_at	APLP2	NM_001642 NM_001142276 NM_00114	-0.247	-2.20	0.0303	1.00
merck2-M35296_at	ABL2	NM_007314 NM_005158 NM_0011360(	-0.157	-2.20	0.0303	1.00
merck-NM_002961_s_at	S100A4	NM_002961 NM_019554	-0.0677	-2.20	0.0304	1.00
merck2-AY128643_at	PMEPA1	NM_020182 NM_199169 NM_199170 N	0.240	2.20	0.0304	1.00
merck-NM_145040_at	PRKCDBP	NM_145040	-0.156	-2.20	0.0304	1.00
merck2-BG674122_a_at	HLF	NM_002126	0.143	2.20	0.0304	1.00
merck-NM_000633_at	BCL2	NM_000633	0.155	2.20	0.0304	1.00
merck-BC013099_a_at	CABIN1	NM_001199281 NM_012295 NM_0012(	-0.156	-2.20	0.0305	1.00
merck2-NM_025170_at	PREX2	NM_025170	0.236	2.20	0.0305	1.00
merck2-NM_005782_at	THOC4	NM_005782	-0.138	-2.20	0.0305	1.00
merck-AI092890_a_at	ZNF704	NM_001033723	-0.124	-2.20	0.0305	1.00
merck2-BC013872_at	TP73-AS1	NR_033711 NR_033710 NR_033709 NI	0.112	2.20	0.0305	1.00
merck-NM_015129_at	SEPT6	NM_145799 NM_015129	0.200	2.20	0.0306	1.00
merck2-BC082765_at	TMEM8B	NM_001042590 NM_001042589	-0.124	-2.20	0.0307	1.00
merck2-NM_001935_at	DPP4	NM_001935	0.230	2.19	0.0307	1.00
merck-NM_017762_at	---	---	-0.176	-2.19	0.0307	1.00
merck-ENST00000372183_a_at	EIF2B3	NM_020365 NM_001166588	-0.112	-2.19	0.0307	1.00
merck-XM_926622_x_at	---	---	0.150	2.19	0.0307	1.00
merck2-AW516817_a_at	LOC146880	NR_027487	-0.150	-2.19	0.0308	1.00
merck2-AL533240_at	TMEM66	NM_016127	0.174	2.19	0.0308	1.00
merck2-NM_003465_at	CHIT1	NM_003465	-0.271	-2.19	0.0308	1.00
merck-XM_943972_at	---	AC074363	-0.0895	-2.19	0.0308	1.00
merck2-NM_022803_at	UCP3	NM_003356 NM_022803	-0.150	-2.19	0.0308	1.00
merck2-CMV_UL94_at	---	---	-0.142	-2.19	0.0308	1.00
merck2-AI399709_at	BAZ2B	NM_013450	-0.154	-2.19	0.0308	1.00
merck-ENST00000215935_x_at	---	---	-0.125	-2.19	0.0309	1.00
merck2-AK026568_x_at	---	---	0.0633	2.19	0.0309	1.00
merck-AW194700_a_at	NR2F2	NM_001145155	-0.138	-2.19	0.0309	1.00
merck-AC_000017_ORF_1085_at	---	---	-0.102	-2.19	0.0309	1.00
merck2-NM_139241_at	FGD4	NM_139241	-0.265	-2.19	0.0309	1.00
merck-NM_052956_s_at	ACSM1	NM_052956	0.128	2.19	0.0310	1.00
merck-NM_032140_at	C16orf48	NM_032140	-0.127	-2.19	0.0310	1.00
merck2-Y16708_x_at	---	Y16708	-0.141	-2.19	0.0310	1.00
merck-CD672190_a_at	PLCE1	NM_016341 NM_001165979	-0.142	-2.19	0.0310	1.00
merck2-CD709967_at	HSPA8	AK310467	0.199	2.19	0.0311	1.00
merck-NM_001005861_at	RYK	NM_001005861 NM_002958	0.149	2.19	0.0311	1.00
merck-BC034591_at	LOC284648	NR_036490	-0.145	-2.19	0.0311	1.00
merck-NM_021183_at	RAP2C	NM_021183	0.226	2.19	0.0311	1.00
merck-BC019877_at	---	BC019877	-0.126	-2.19	0.0311	1.00
merck-AK027399_s_at	ZFYVE1	NM_021260 NM_178441	-0.170	-2.19	0.0311	1.00
merck-NM_031889_at	ENAM	NM_031889	0.254	2.19	0.0311	1.00
merck2-CR595807_at	---	CR595807	0.308	2.19	0.0311	1.00
merck-AK130024_at	---	XM_003118742 XM_003119680 XM_00	0.152	2.19	0.0311	1.00
merck-AI417759_a_at	LOC100505987	XR_110833	0.240	2.19	0.0312	1.00
merck2-AK027738_at	FOXP1	BX647682	0.127	2.19	0.0312	1.00
merck-AK027821_a_at	TSEN2	NM_025265 NM_001145392 NM_00114	0.167	2.19	0.0312	1.00
merck-NM_004850_a_at	ROCK2	NM_004850	-0.121	-2.19	0.0313	1.00
merck-ENST00000309184_s_at	---	AJ404607	-0.262	-2.19	0.0314	1.00
merck-NM_004778_at	PTGDR2	NM_004778	-0.231	-2.18	0.0314	1.00
merck-NM_199227_s_at	METAP1D	NM_199227	0.143	2.18	0.0315	1.00
merck-BC014494_at	---	BC014494	-0.143	-2.18	0.0315	1.00
merck-AF130047_at	---	AF130047	-0.131	-2.18	0.0315	1.00
merck2-AK124553_at	EML6	NM_001039753	0.265	2.18	0.0315	1.00
merck-ENST00000376775_at	LOC729275	AK129559	0.284	2.18	0.0315	1.00
merck-BI905840_at	---	---	-0.117	-2.18	0.0315	1.00
merck2-BX641100_at	---	---	-0.124	-2.18	0.0315	1.00
merck-NM_080681_a_at	COL11A2	NM_080680 NM_080681 NM_080679	-0.151	-2.18	0.0315	1.00
merck-ENST00000375258_at	METTL8	NM_024770	0.208	2.18	0.0316	1.00
merck-AB209912_a_at	THBS1	NM_003246	-0.362	-2.18	0.0316	1.00
merck-BX647131_at	RBMX	NM_001164803	0.173	2.18	0.0316	1.00
merck-BM666105_a_at	CUX1	NM_181552 NM_001202543 XR_10885	-0.130	-2.18	0.0316	1.00
merck2-BC018124_at	HSPH1	NM_006644	0.122	2.18	0.0316	1.00
merck2-AI003348_at	NMNAT2	NM_015039 NM_170706	-0.113	-2.18	0.0316	1.00
merck-ENST00000263850_a_at	TPD52	NM_001025252 NM_001025253 NM_0(	0.297	2.18	0.0317	1.00
merck-AK131093_a_at	CHIT1	NM_003465	-0.269	-2.18	0.0317	1.00
merck2-AK128370_at	CD160	NM_007053	0.345	2.18	0.0317	1.00
merck-NM_001877_at	CR2	NM_001006658 NM_001877	0.258	2.18	0.0317	1.00
merck2-BE935035_at	---	---	0.320	2.18	0.0318	1.00
merck2-NM_001007545_at	HEATR1	BC062442	-0.135	-2.18	0.0318	1.00
merck-M80919_at	---	---	0.116	2.18	0.0319	1.00
merck-NM_013269_x_at	CLEC2D	NM_013269 NM_001004419 NM_0011§	0.133	2.18	0.0319	1.00
merck-ENST00000343315_s_at	TMPO	NM_001032283 NM_001032284	0.187	2.18	0.0319	1.00
merck2-BG119190_at	---	---	0.177	2.18	0.0319	1.00
merck-AL831948_at	---	AL831948	-0.158	-2.18	0.0320	1.00
merck-BF683837_at	---	---	-0.103	-2.18	0.0320	1.00
merck2-AL137325_at	---	AL137325	0.145	2.18	0.0320	1.00
merck-XM_945176_at	---	AL035078	-0.118	-2.18	0.0320	1.00
merck2-VZV_OKA_ORF32_at	---	NC_001348	-0.0885	-2.18	0.0320	1.00
merck2-VZV_OKA_ORF9_at	---	NC_001348	-0.132	-2.18	0.0320	1.00
merck-AB209742_at	PARP9	NM_001146106	-0.292	-2.18	0.0321	1.00
merck2-BX537545_a_at	---	BX537545	0.185	2.18	0.0321	1.00
merck-CA428475_at	---	AC018978	-0.102	-2.18	0.0321	1.00
merck-ENST00000359646_a_at	RBM12	NM_006047 NM_152838 NM_0011988(	0.208	2.18	0.0321	1.00
merck-NM_152133_at	TAGAP	NM_152133 NM_054114	0.115	2.18	0.0321	1.00
merck-U18914_a_at	TPD52	NM_001025252 NM_001025253 NM_0(	0.236	2.18	0.0322	1.00
merck-NM_152312_at	GYLTL1B	NM_152312	0.156	2.18	0.0322	1.00
merck-BX358637_a_at	MMP11	NM_005940	-0.149	-2.17	0.0322	1.00
merck-NM_001004316_at	LEKR1	NM_001004316	0.195	2.17	0.0322	1.00
merck2-BC017925_at	LMOD3	NM_198271	-0.125	-2.17	0.0323	1.00
merck2-AY254199_at	FAM129C	NM_173544	0.268	2.17	0.0323	1.00
merck-AF023476_s_at	ADAM12	NM_003474	-0.126	-2.17	0.0324	1.00
merck-AW134779_at	---	AC004009	-0.0980	-2.17	0.0325	1.00
merck2-NM_015463_at	CNRIP1	NM_015463	-0.171	-2.17	0.0325	1.00
merck-BC043286_at	---	---	-0.0934	-2.17	0.0325	1.00
merck-ENST00000302464_a_at	UBE2L3	NM_003347 NR_028436 NR_028437	-0.0985	-2.17	0.0325	1.00
merck-NM_002120_at	HLA-DOB	NM_002120	0.329	2.17	0.0325	1.00
merck-BG222187_x_at	HSPA8	AB034951	0.138	2.17	0.0326	1.00
merck-BC047864_a_at	APOL6	NM_030641	-0.219	-2.17	0.0326	1.00
merck2-AL157459_at	CBX2	AL157459	-0.171	-2.17	0.0326	1.00
merck-BC045769_at	LOC339192	BC045769	-0.246	-2.17	0.0326	1.00
merck-AF035036_x_at	---	---	0.271	2.17	0.0327	1.00
merck2-BC033795_s_at	---	---	0.159	2.17	0.0327	1.00
merck-NM_194277_s_at	FRMD7	NM_194277	-0.128	-2.17	0.0327	1.00
merck2-Rota_P_s9_at	---	---	-0.0871	-2.17	0.0327	1.00
merck-AJ845164_s_at	LOC100507616	XR_110328 XR_111328 XR_114669	0.209	2.17	0.0327	1.00
merck2-ENST00000390351_x_at	---	AF316870	0.364	2.17	0.0327	1.00
merck-NM_173562_a_at	KCTD20	NM_173562	-0.160	-2.17	0.0328	1.00
merck2-NM_001001802_s_at	---	---	0.245	2.17	0.0328	1.00
merck2-XM_091890_a_at	ZNF320	NM_207333	0.265	2.17	0.0328	1.00
merck-XM_932886_at	---	AC079059	-0.134	-2.17	0.0328	1.00
merck2-U52682_at	IRF4	NM_002460 NM_001195286 NR_03658	0.170	2.17	0.0329	1.00
merck-NM_019063_at	EML4	NM_019063 NM_001145076	0.151	2.17	0.0329	1.00
merck-NM_030920_at	ANP32E	NM_030920 NM_001136478 NM_00113	0.202	2.17	0.0329	1.00
merck-AL570146_s_at	---	---	0.180	2.17	0.0329	1.00
merck-NM_024613_at	PLEKHF2	NM_024613	0.285	2.17	0.0329	1.00
merck-NM_199189_a_at	MATR3	NM_199189 NM_018834 NM_0011949!	0.152	2.16	0.0330	1.00
merck2-AK093871_at	---	AK093871	-0.181	-2.16	0.0330	1.00
merck2-AI126618_at	KHDRBS1	NM_006559	0.109	2.16	0.0330	1.00
merck-BM687995_at	---	---	-0.129	-2.16	0.0330	1.00
merck-BU959629_at	---	---	-0.115	-2.16	0.0330	1.00
merck-hCT2293782_at	---	AC013437	-0.243	-2.16	0.0330	1.00

merck2-NM_030928_at	CDT1	NM_030928	-0.141	-2.16	0.0331	1.00
merck2-AV710073_at	---	---	-0.115	-2.16	0.0331	1.00
merck-BI552493_a_at	SCG3	NM_013243 NM_001165257	-0.129	-2.16	0.0331	1.00
merck-NM_002675_at	PML	NM_033249 NM_002675 NM_033246	-0.208	-2.16	0.0331	1.00
merck-AL049349_at	---	AL049349	-0.148	-2.16	0.0331	1.00
merck-BQ959720_at	EIF1AX	AL832930	0.201	2.16	0.0331	1.00
merck-CR936744_at	---	XR_110598 XR_111540 XR_114879	0.174	2.16	0.0331	1.00
merck-XM_936048_at	---	NG_006083	0.195	2.16	0.0332	1.00
merck-BC040587_at	LSAMP-AS4	XR_108471 XR_112529 XR_113582	-0.107	-2.16	0.0332	1.00
merck-NM_032561_at	C22orf23	NM_032561	-0.125	-2.16	0.0332	1.00
merck-NM_004677_s_at	XKRY XKRY2	NM_004677 NM_001002906	-0.202	-2.16	0.0332	1.00
merck2-BX475081_at	---	CR933606	0.251	2.16	0.0333	1.00
merck2-BX649160_a_at	BCLAF1	NM_014739 NM_001077440 NM_0010	0.205	2.16	0.0333	1.00
merck2-NM_004682_a_at	PSIP1	NM_033222 NM_001128217	0.271	2.16	0.0333	1.00
merck-BX647225_at	TLK2	NM_006852 NM_001112707	-0.103	-2.16	0.0333	1.00
merck2-BC029540_s_at	---	---	0.511	2.16	0.0334	1.00
merck-NM_003287_at	TPD52L1	NM_003287 NM_001003395 NM_0010	0.140	2.16	0.0334	1.00
merck-NM_001822_s_at	CHN1	NM_001822 NM_001025201	0.234	2.16	0.0334	1.00
merck-BC036382_at	SLC35G1	BC047102	0.169	2.16	0.0334	1.00
merck-R85439_at	---	---	-0.145	-2.16	0.0334	1.00
merck2-HSV1_ORF_UL24_at	---	---	-0.149	-2.16	0.0334	1.00
merck-NM_173078_at	SLITRK4	NM_001184749 NM_173078 NM_0011	0.260	2.16	0.0334	1.00
merck-BC038745_at	---	XR_109649 XR_112123 XR_115346	0.151	2.16	0.0335	1.00
merck-BC036240_a_at	SEPT6	NM_145799 NM_015129	0.118	2.16	0.0335	1.00
merck2-NM_001036646_a_at	HHLA3	NM_001031693 NM_001036645 NM_00	0.153	2.16	0.0335	1.00
merck2-BI754652_at	SLC25A4	NM_001151	0.153	2.16	0.0335	1.00
merck-BC065843_at	COL4A4	NM_000092	0.264	2.16	0.0336	1.00
merck-ENST00000374750_s_at	GLB1L3	NM_001080407	-0.136	-2.16	0.0336	1.00
merck-BC022040_at	---	---	0.241	2.16	0.0336	1.00
merck-NM_024684_at	C11orf67	NM_024684	-0.177	-2.16	0.0336	1.00
merck-DB308208_at	---	AC005748	0.274	2.16	0.0336	1.00
merck-NM_005660_at	SLC35A2	NM_005660	-0.218	-2.16	0.0336	1.00
merck2-hCT1850182.1_at	---	BC089413	0.146	2.16	0.0337	1.00
merck-AK057576_at	---	AK057576	-0.186	-2.16	0.0337	1.00
merck-ENST00000367962_a_at	FCGR2B	NM_004001 NM_001002273 NM_0010	0.314	2.16	0.0337	1.00
merck2-CR936789_at	FSD1L	CR936789	0.163	2.16	0.0337	1.00
merck-NM_003632_at	CNTNAP1	NM_003632	0.180	2.16	0.0337	1.00
merck2-BG230607_at	PSAP	NM_002778 NM_001042465 NM_0010	-0.0654	-2.16	0.0337	1.00
merck-BU685426_at	MUC20	NM_152673 NM_001098516	0.144	2.16	0.0337	1.00
merck-DB443631_at	---	---	0.127	2.15	0.0338	1.00
merck-NM_004568_s_at	SERPINB6	NM_004568 NM_001195291	-0.160	-2.15	0.0338	1.00
merck-NM_018304_s_at	PRR11	NM_018304	-0.147	-2.15	0.0338	1.00
merck-NM_144633_at	KCNH8	NM_144633	0.327	2.15	0.0339	1.00
merck-BF515552_at	---	AL158062	0.195	2.15	0.0339	1.00
merck-XM_497859_at	---	---	-0.123	-2.15	0.0339	1.00
merck-AK092172_at	LOC286109	AK092172	-0.157	-2.15	0.0339	1.00
merck-AK026933_a_at	EIF5	NM_001969 NM_183004	0.144	2.15	0.0339	1.00
merck-ENST00000354314_at	---	---	-0.128	-2.15	0.0340	1.00
merck2-BQ016861_at	---	AC240516	0.124	2.15	0.0340	1.00
merck2-AI051196_a_at	LRP1	NM_002332	-0.249	-2.15	0.0340	1.00
merck-BQ774745_at	---	---	-0.169	-2.15	0.0340	1.00
merck2-BC112087_at	KCNE2	NM_172201	0.157	2.15	0.0340	1.00
merck2-NM_152336_at	AGBL1	NM_152336	-0.121	-2.15	0.0340	1.00
merck2-AB064041_x_at	---	---	0.138	2.15	0.0340	1.00
merck-NM_052960_at	RBP7	NM_052960	-0.214	-2.15	0.0341	1.00
merck-NM_001012715_at	C9orf106	AK092588	-0.180	-2.15	0.0341	1.00
merck-NM_015055_at	SWAP70	NM_015055	0.246	2.15	0.0341	1.00
merck-NM_006626_at	ZBTB6	NM_006626	0.150	2.15	0.0341	1.00
merck-AW341489_a_at	AFF2	NM_002025 NM_001169122 NM_0011	-0.109	-2.15	0.0341	1.00
merck-NM_005292_at	GPR18	NM_005292 NM_001098200	0.175	2.15	0.0342	1.00
merck-NM_153612_a_at	HS3ST5	NM_153612	-0.123	-2.15	0.0343	1.00
merck2-BF195886_at	---	---	-0.138	-2.15	0.0343	1.00
merck2-AL538641_at	---	AL713980	0.356	2.15	0.0343	1.00
merck-NM_001953_s_at	TYMP	NM_001113755 NM_001953 NM_0011	-0.211	-2.15	0.0343	1.00
merck-NM_138450_at	ARL11	NM_138450	-0.187	-2.15	0.0344	1.00
merck2-NM_152440_at	C12orf66	NM_152440	0.153	2.15	0.0345	1.00
merck-NM_017567_at	NAGK	NM_017567	-0.170	-2.15	0.0345	1.00
merck-AF520746_a_at	SDC4P	NR_001580	-0.136	-2.15	0.0345	1.00
merck-BI091220_a_at	GM2A	NM_000405	-0.191	-2.15	0.0345	1.00
merck2-M27539_x_at	HLA-A LOC100507703	NM_002116 XM_003119226 XM_0031	-0.0725	-2.15	0.0345	1.00
merck-NM_020448_s_at	NIPAL3	NM_020448	0.125	2.15	0.0345	1.00
merck2-AB209563_at	SERPINB6	NM_004568 NM_001195291	-0.173	-2.15	0.0346	1.00
merck-NM_001005373_at	LRSAM1	NM_138361 NM_001005373 NM_0010	-0.134	-2.15	0.0346	1.00
merck2-U39114_s_at	---	---	0.195	2.15	0.0346	1.00
merck-AL711312_at	---	AC123786	-0.135	-2.15	0.0346	1.00
merck-hCT1834893.1_at	---	NG_009747	-0.125	-2.14	0.0346	1.00
merck-NM_021049_at	MAGEA5	NM_021049	0.191	2.14	0.0346	1.00
merck-ENST00000336387_a_at	CXorf41	NM_001169154 NM_173494	0.227	2.14	0.0346	1.00
merck2-BC002665_at	PLP1	NM_000533 NM_199478 NM_0011288	0.317	2.14	0.0346	1.00
merck-NM_175068_at	KRT73	NM_175068	-0.278	-2.14	0.0346	1.00
merck-ENST00000376124_at	GDPD1	NM_182569 NM_001165993	0.186	2.14	0.0347	1.00
merck-AL049270_at	SLC5A12	NM_178498	-0.0953	-2.14	0.0347	1.00
merck-AK091442_at	---	AK091442	0.124	2.14	0.0347	1.00
merck-BU688698_s_at	---	---	-0.129	-2.14	0.0347	1.00
merck2-AL832977_s_at	---	---	0.224	2.14	0.0347	1.00
merck-CR601230_a_at	CYBB	NM_000397	-0.143	-2.14	0.0347	1.00
merck2-AK096708_at	EBF1	NM_024007	0.230	2.14	0.0348	1.00
merck-AW207203_at	---	AL160398	0.145	2.14	0.0348	1.00
merck-AK055524_at	---	AK055524	-0.133	-2.14	0.0348	1.00
merck-NM_152456_at	IL34	NM_152456 NM_001172771 NM_0011	-0.100	-2.14	0.0349	1.00
merck-AK090920_at	LOC100130373	AK090920	-0.193	-2.14	0.0349	1.00
merck-XM_937466_at	---	---	0.155	2.14	0.0349	1.00
merck2-BC016691_at	RNF20	NM_019592	-0.208	-2.14	0.0349	1.00
merck2-BC018532_at	FAM129A	NM_052966	-0.311	-2.14	0.0350	1.00
merck2-BE881281_at	MOXD1	NM_015529	0.185	2.14	0.0350	1.00
AFFX-DapX-3_at	---	---	-0.0811	-2.14	0.0350	1.00
merck-XM_210737_at	---	NG_009950	-0.179	-2.14	0.0350	1.00
merck2-AA738241_at	LOC100506610	XR_110929 XR_113300 XR_115503	0.186	2.14	0.0350	1.00
merck2-XM_166279_at	RSPRY1	AL834402	0.125	2.14	0.0350	1.00
merck2-NM_005953_x_at	MT2A	NM_005953	-0.226	-2.14	0.0350	1.00
merck2-NM_138367_at	ZNF251	NM_138367	0.179	2.14	0.0351	1.00
merck-BM925199_at	---	---	0.168	2.14	0.0351	1.00
merck-AY888048_at	TAF4B	NM_005640	0.197	2.14	0.0351	1.00
merck2-NM_024318_at	LILRA6	NM_024318	-0.186	-2.14	0.0351	1.00
merck-NM_145176_at	SLC2A12	NM_145176	0.215	2.14	0.0351	1.00
merck-AK095773_at	---	AK095773	-0.212	-2.14	0.0352	1.00
merck-ENST00000250805_a_at	TTTY1 TTTY1B	NR_001538 NR_003589	-0.246	-2.14	0.0352	1.00
merck-NM_020415_at	RETN	NM_020415 NM_001193374	-0.236	-2.14	0.0352	1.00
merck-NM_199327_s_at	SPRY1	NM_005841 NM_199327	0.248	2.14	0.0352	1.00
merck-AK027795_at	---	AK027795	-0.186	-2.14	0.0352	1.00
merck2-NM_020827_at	KIAA1430	NM_020827	0.198	2.14	0.0352	1.00
merck2-AY515008_at	---	AY515008	-0.0658	-2.14	0.0353	1.00
merck2-AA888002_at	ARHGEF37	NM_001001669	-0.138	-2.14	0.0353	1.00
merck-BC040672_at	---	BC040672	0.117	2.14	0.0353	1.00
merck2-BF061740_x_at	---	BC059952	-0.141	-2.14	0.0353	1.00
merck-NM_021949_at	ATP2B3	NM_021949 NM_001001344	0.126	2.14	0.0353	1.00
merck-BX111225_s_at	ANKRD36 ANKRD36B LOC400986	NM_001164315 NM_025190 XM_0031	0.237	2.14	0.0353	1.00
merck-NM_001024455_s_at	RGAG4	NM_001024455	-0.179	-2.14	0.0354	1.00
merck2-BX647215_at	---	BX647215	0.153	2.14	0.0354	1.00
merck-NM_001987_s_at	ETV6	NM_001987	-0.100	-2.14	0.0354	1.00
merck-AF269286_at	---	AF269286	0.159	2.13	0.0354	1.00
merck-NM_002959_at	SORT1	NM_002959	-0.188	-2.13	0.0354	1.00
merck2-BX410084_a_at	TPD52	NM_001025252 NM_001025253 NM_00	0.345	2.13	0.0354	1.00
merck-ENST00000332111_x_at	OR7E37P MGC72080	NR_002163 NR_002822	-0.151	-2.13	0.0355	1.00
merck2-DQ473380_x_at	---	---	0.351	2.13	0.0355	1.00
merck2-NM_001018104_at	FAHD1	NM_001018104 NM_001142398	0.129	2.13	0.0355	1.00
merck2-BF434597_at	RORA	NM_134261	0.335	2.13	0.0355	1.00
merck-AI553879_a_at	TSEN15	NM_052965 NM_001127394 NR_02334	0.117	2.13	0.0355	1.00
merck2-BG751819_at	SLC20A2	NM_006749	-0.138	-2.13	0.0356	1.00

merck-AL832916_at	---	AL832916	0.520	2.13	0.0356	1.00
merck2-NM_080794_at	MRPL39	NM_080794	0.146	2.13	0.0356	1.00
merck2-NM_005332_a_at	HBZ	NM_005332	-0.428	-2.13	0.0357	1.00
merck-NM_005902_at	SMAD3	NM_005902 NM_001145102 NM_00114	0.132	2.13	0.0357	1.00
merck-BM547658_at	---	---	-0.129	-2.13	0.0357	1.00
merck-hCT1970787.1_at	---	---	-0.106	-2.13	0.0358	1.00
merck2-BI519527_at	IKZF1	NM_006060	0.0933	2.13	0.0358	1.00
merck-NM_001039463_at	NEXN-AS1	XR_110470 XR_112069 XR_113334	-0.127	-2.13	0.0358	1.00
merck2-U16261_at	IL24	NM_006850 NM_001185156 NM_00118	0.179	2.13	0.0358	1.00
merck-AK055172_a_at	DLG5	NM_004747	-0.173	-2.13	0.0358	1.00
merck-AL512683_s_at	SCRT1	NM_031309	-0.0851	-2.13	0.0358	1.00
merck-NM_020799_s_at	STAMBPL1	NM_020799	0.193	2.13	0.0359	1.00
merck-NM_032211_at	LOXL4	NM_032211	-0.144	-2.13	0.0359	1.00
merck2-BF511680_at	AMOTL1	NM_130847	0.130	2.13	0.0359	1.00
merck2-CA447088_at	VPS4B	NM_004869	0.153	2.13	0.0359	1.00
merck-NM_006805_at	HNRNPA0	NM_006805	0.142	2.13	0.0359	1.00
merck-AK055284_s_at	---	AK055284	-0.114	-2.13	0.0360	1.00
merck2-ENST00000298824_x_at	ARHGAP42	NM_152432	0.123	2.13	0.0360	1.00
merck-R96493_x_at	---	---	0.0947	2.13	0.0360	1.00
merck-ENST00000268533_at	NUDT7	NM_001105663	0.165	2.13	0.0360	1.00
merck-NM_018334_at	LRRN3	NM_001099660 NM_001099658 NM_0	0.440	2.13	0.0360	1.00
merck-AI929279_s_at	---	AK090408	0.125	2.13	0.0360	1.00
merck-AK002120_a_at	DCX	NM_000555 NM_178152 NM_178153 N	0.481	2.13	0.0361	1.00
merck2-BC009795_a_at	TNFSF10	NM_003810 NM_001190942 NR_03399	-0.130	-2.13	0.0361	1.00
merck-NM_005582_at	CD180	NM_005582	0.233	2.13	0.0361	1.00
merck-BC009467_at	LOC158960	BC009467	-0.137	-2.13	0.0361	1.00
merck-NM_001963_at	EGF	NM_001963 NM_001178130 NM_00117	-0.281	-2.13	0.0361	1.00
merck-DA633754_a_at	CTNNA1	NM_001903	-0.141	-2.13	0.0361	1.00
merck2-T64550_a_at	KHDRBS1	NM_006559	0.106	2.13	0.0362	1.00
merck-NM_032121_s_at	MAGT1	NM_032121	0.166	2.13	0.0362	1.00
merck-NM_005036_at	PPARA	NM_001001928 NM_005036	-0.204	-2.13	0.0362	1.00
merck-NM_014728_s_at	FRMPD4	NM_014728	0.151	2.13	0.0362	1.00
merck-AK055833_a_at	TRIM14	NM_014788	-0.169	-2.13	0.0362	1.00
merck2-CD653323_at	IGF1R	NM_000875	-0.131	-2.12	0.0363	1.00
merck2-HSV2_ORF_US11_s_at	---	---	-0.156	-2.12	0.0363	1.00
merck-XM_937660_at	---	---	-0.121	-2.12	0.0363	1.00
merck-ENST00000265121_at	ODZ3	NM_001080477	0.149	2.12	0.0364	1.00
merck-NM_173570_s_at	ZDHHC23	NM_173570	0.251	2.12	0.0364	1.00
merck-ENST00000276770_s_at	TTTY7 TTTY7B	NR_001534 NR_003592	-0.141	-2.12	0.0364	1.00
merck2-BX649155_a_at	PSIP1	NM_033222 NM_001128217	0.249	2.12	0.0364	1.00
merck-NM_002862_at	PYGB	NM_002862	-0.176	-2.12	0.0364	1.00
merck2-BU602759_at	FLJ39051	NR_033839	-0.243	-2.12	0.0365	1.00
merck2-BG744509_at	---	---	0.0986	2.12	0.0365	1.00
merck-CR744385_at	---	AC206362	-0.103	-2.12	0.0365	1.00
merck-NM_004060_at	CCNG1	NM_004060 NM_199246	0.190	2.12	0.0366	1.00
merck-AY190101_at	---	AY190101	0.111	2.12	0.0366	1.00
merck2-CR590554_at	---	CR594785	0.246	2.12	0.0366	1.00
merck-AK023738_at	CEP63	NM_001042384	-0.132	-2.12	0.0366	1.00
merck-AK123300_at	---	AK123300	-0.145	-2.12	0.0366	1.00
merck-NM_152904_s_at	SPECC1	NM_001033554 NM_001033555 NM_1	-0.158	-2.12	0.0367	1.00
merck-NM_007030_s_at	TPPP	NM_007030	-0.109	-2.12	0.0367	1.00
merck2-NM_002332_at	LRP1	NM_002332	-0.238	-2.12	0.0367	1.00
merck-NM_005375_at	MYB	NM_001130173 NM_005375 NM_00113	-0.155	-2.12	0.0367	1.00
merck2-NM_152585_s_at	---	---	-0.100	-2.12	0.0367	1.00
merck-XM_936332_x_at	---	U07977	0.190	2.12	0.0368	1.00
merck2-BI666677_at	---	---	-0.276	-2.12	0.0368	1.00
merck-ENST00000359488_x_at	---	---	0.359	2.12	0.0368	1.00
merck2-D45917_a_at	TIMP3	NM_000362	-0.128	-2.12	0.0368	1.00
merck-ENST00000271011_a_at	SERBP1	NM_001018067 NM_001018068 NM_0	0.129	2.12	0.0368	1.00
merck-XM_067107_a_at	---	---	-0.135	-2.12	0.0368	1.00
merck-ENST00000355100_a_at	ABHD2	NM_007011 NM_152924	-0.169	-2.12	0.0368	1.00
merck-AL535164_a_at	RING1	NM_002931	-0.133	-2.12	0.0369	1.00
merck2-BC007782_at	IGLC1	BC007782	0.335	2.12	0.0369	1.00
merck-XM_937489_at	---	---	0.153	2.12	0.0369	1.00
merck-AF150307_at	---	---	-0.171	-2.12	0.0369	1.00
merck-ENST00000371556_s_at	HSP90AB1 HSP90AB3P	NM_007355 NR_036694	0.143	2.12	0.0369	1.00
merck-AV648465_at	---	---	-0.120	-2.12	0.0369	1.00
merck-NM_153834_at	GPR112	NM_153834	0.338	2.12	0.0370	1.00
merck2-BF792881_at	---	---	-0.103	-2.12	0.0370	1.00
merck-AK057759_at	---	AK057759	0.175	2.12	0.0370	1.00
merck2-DB567116_at	TMEM35	NM_021637	0.0975	2.12	0.0370	1.00
merck-NM_014632_s_at	MICAL2	NM_014632	-0.290	-2.12	0.0370	1.00
merck-AW206100_a_at	SNAP23	NM_003825 NM_130798	-0.146	-2.12	0.0370	1.00
merck-NM_007156_s_at	ZXDA ZXDB	NM_007156 NM_007157	0.169	2.12	0.0370	1.00
merck-NM_025080_s_at	ASRGL1	NM_001083926 NM_025080	-0.224	-2.12	0.0371	1.00
merck2-BQ614044_at	---	---	-0.114	-2.12	0.0371	1.00
merck-BC056887_a_at	FAM13B	NM_016603 NM_001101800 NM_0011	0.0940	2.12	0.0371	1.00
merck-NM_007249_at	KLF12	NM_007249	0.189	2.12	0.0371	1.00
merck2-NM_020780_at	PTCHD2	NM_020780	-0.120	-2.12	0.0371	1.00
merck2-BU430449_a_at	FYTTD1	NM_032288 NM_001011537 NR_02784	-0.123	-2.12	0.0371	1.00
merck2-BE272644_s_at	---	---	0.131	2.11	0.0371	1.00
merck-NM_003440_at	ZNF140	NM_003440	0.197	2.11	0.0372	1.00
merck-H75698_at	---	NG_007095	-0.143	-2.11	0.0372	1.00
merck-NM_007105_at	SLC22A18AS	NM_007105	-0.180	-2.11	0.0372	1.00
merck-hCT1782279_at	---	AF196997	0.186	2.11	0.0373	1.00
merck2-ENST00000255741_at	GNA11	NM_002067	-0.121	-2.11	0.0373	1.00
merck-NM_001771_at	CD22	NM_001771 NM_001185099 NM_0011	0.298	2.11	0.0373	1.00
merck-XM_926941_s_at	---	---	0.224	2.11	0.0373	1.00
merck-NM_207467_at	C1orf220	NR_033186	0.152	2.11	0.0374	1.00
merck-AK024851_at	---	AK024851	-0.217	-2.11	0.0374	1.00
merck-AK091308_at	---	AK091308	0.152	2.11	0.0374	1.00
merck-NM_152426_at	---	---	-0.111	-2.11	0.0374	1.00
merck-NM_052817_at	MID2	NM_012216 NM_052817	0.151	2.11	0.0374	1.00
merck-hCT1832253.1_at	---	AL391419	0.257	2.11	0.0375	1.00
merck2-AI862445_at	TNFAIP2	NM_006291	-0.187	-2.11	0.0375	1.00
merck-NM_058182_at	FAM165B	AY081143	0.142	2.11	0.0375	1.00
merck2-BE551097_at	---	---	0.213	2.11	0.0375	1.00
merck-BF876969_at	---	---	-0.232	-2.11	0.0375	1.00
merck-NM_002163_at	IRF8	NM_002163	0.153	2.11	0.0375	1.00
merck-NM_014639_a_at	TTC37	NM_014639	0.197	2.11	0.0376	1.00
merck-AF015041_a_at	NUMBL	NM_004756	-0.164	-2.11	0.0376	1.00
merck-AB037837_a_at	CHD7	NM_017780	-0.168	-2.11	0.0376	1.00
merck-BC047305_a_at	COL4A1	NM_001845	-0.103	-2.11	0.0376	1.00
merck-XM_497647_at	---	---	-0.155	-2.11	0.0376	1.00
merck2-BQ013763_at	NBLA00301	EU000854	-0.120	-2.11	0.0376	1.00
merck-AK022257_at	---	AK022257	-0.177	-2.11	0.0376	1.00
merck2-NM_000391_at	TPP1	NM_000391	-0.135	-2.11	0.0376	1.00
merck2-AB078432_at	GCNT2	NM_145649	-0.156	-2.11	0.0377	1.00
merck-ENST00000340858_a_at	ALOX12P2	NR_002710	0.123	2.11	0.0378	1.00
merck-NM_130469_s_at	JDP2	NM_130469 NM_001135047 NM_00113	-0.176	-2.11	0.0378	1.00
merck2-AK091280_at	FAM49A	NM_030797	-0.0931	-2.11	0.0378	1.00
merck-NM_015271_at	TRIM2	NM_015271 NM_001130067	0.177	2.11	0.0378	1.00
merck-NM_001010855_a_at	PIK3R6	NM_001010855	-0.176	-2.11	0.0378	1.00
merck-NM_001009894_at	C12orf29	NM_001009894	0.228	2.11	0.0378	1.00
merck2-NM_018553_at	C17orf85	NM_001114118 NM_018553	-0.0908	-2.11	0.0379	1.00
merck-AK097996_at	---	AK097996	-0.152	-2.11	0.0379	1.00
merck2-AA077332_x_at	ATP5J2 ATP5J2-PTCD1	NM_004889 NM_001003713 NM_0010	-0.174	-2.11	0.0379	1.00
merck2-NM_207292_at	MBNL1	NM_021038 NM_207292 NM_207293 N	0.156	2.11	0.0379	1.00
merck2-BX648290_at	---	BX648290	-0.205	-2.11	0.0379	1.00
merck-AY007155_at	LOC439949	NR_036502 NR_036503	0.200	2.11	0.0379	1.00
merck-AK097037_at	ZNF525	NR_003699	0.162	2.11	0.0380	1.00
merck-ENST00000370551_s_at	HS2ST1	NM_001134492	-0.158	-2.10	0.0380	1.00
merck-NM_000137_at	FAH	NM_000137	-0.170	-2.10	0.0380	1.00
merck-X64982_s_at	OR9A1P	X64982	0.144	2.10	0.0381	1.00
merck-NM_001211_at	BUB1B	NM_001211	-0.146	-2.10	0.0381	1.00
merck-NM_016220_s_at	ZNF107	NM_016220 NM_001013746	0.395	2.10	0.0381	1.00
merck-AF036974_x_at	---	---	0.235	2.10	0.0381	1.00
merck-NM_003411_a_at	ZFY	NM_003411 NM_001145275 NM_00114	-0.592	-2.10	0.0382	1.00
merck-NM_152407_at	GRPEL2	NM_152407	0.170	2.10	0.0382	1.00

merck-BQ942635_a_at	RBPJ	NM_005349 NM_015874 NM_203283 N	-0.101	-2.10	0.0382	1.00
merck2-AW814840_at	---	---	0.0873	2.10	0.0382	1.00
merck-BC023251_a_at	KIAA1609	NM_020947	-0.133	-2.10	0.0382	1.00
merck-R64384_at	---	AC019181	0.220	2.10	0.0383	1.00
merck-CR624071_at	ATF1	NM_005171	0.259	2.10	0.0383	1.00
merck2-AK222757_at	ITIH3	NM_002217	-0.127	-2.10	0.0383	1.00
merck-ENST00000312019_s_at	FLJ32790	XR_109256 XR_111642 XR_114919	-0.188	-2.10	0.0384	1.00
merck-ENST00000255531_a_at	PCDH19	NM_001105243 NM_020766 NM_00118	0.278	2.10	0.0384	1.00
merck-AI022645_at	---	---	0.185	2.10	0.0384	1.00
merck2-BF439126_at	---	---	-0.101	-2.10	0.0384	1.00
merck-DA156101_a_at	TCERG1	NM_006706 NM_001040006	-0.124	-2.10	0.0385	1.00
merck2-CB853209_at	---	AK124776	0.143	2.10	0.0385	1.00
merck-NM_181337_at	KAAG1	NM_181337	-0.106	-2.10	0.0385	1.00
merck-CD687039_a_at	DNAH12	NM_178504 NM_198564	0.143	2.10	0.0385	1.00
merck-NM_001003408_at	ABLIM1	NM_002313 NM_001003407 NM_0010(	0.140	2.10	0.0386	1.00
merck-NM_020370_at	GPR84	NM_020370	-0.275	-2.10	0.0386	1.00
merck-NM_001005168_at	OR52E8	NM_001005168	-0.101	-2.10	0.0387	1.00
merck-AI218739_at	---	---	-0.209	-2.10	0.0387	1.00
merck-AK023131_at	XPR1	NM_004736 NM_001135669	-0.242	-2.10	0.0387	1.00
merck-NM_021637_at	TMEM35	NM_021637	0.141	2.10	0.0387	1.00
merck-NM_000039_a_at	APOA1	NM_000039	-0.122	-2.10	0.0387	1.00
merck2-NM_000677_at	ADORA3	NM_000677	-0.323	-2.10	0.0387	1.00
merck-AK056597_s_at	OTUD4	NM_001102653	-0.161	-2.10	0.0388	1.00
merck2-D79986_at	BCLAF1	NM_014739 NM_001077440 NM_00107	0.156	2.10	0.0388	1.00
merck-BF028446_a_at	PEG10	NM_001040152 NM_001184961 NM_0(	0.199	2.10	0.0389	1.00
merck-BC012184_s_at	SNX27	NM_030918	-0.133	-2.10	0.0389	1.00
merck2-NM_021049_at	MAGEA5	NM_021049	0.322	2.09	0.0389	1.00
merck-AK054953_at	LOC200830	AK054953	-0.133	-2.09	0.0389	1.00
merck-NM_006820_at	IFI44L	NM_006820	-0.547	-2.09	0.0390	1.00
merck-NM_173571_s_at	CT47A1 CT47A10 CT47A11 CT47A2 C'	NM_001080146 NM_001080137 NM_17	0.465	2.09	0.0390	1.00
merck-BC040270_at	---	BC040270	0.121	2.09	0.0390	1.00
merck-AF088050_x_at	---	AF088050	-0.182	-2.09	0.0391	1.00
merck-BX640620_x_at	---	---	0.286	2.09	0.0392	1.00
merck-XM_063329_at	---	---	-0.103	-2.09	0.0392	1.00
merck2-NM_001001668_at	ZNF470	NM_001001668	0.172	2.09	0.0392	1.00
merck2-BC022485_at	GNG4	NM_001098722 NM_001098721 NM_0(	-0.131	-2.09	0.0392	1.00
merck2-BQ775674_s_at	---	---	0.143	2.09	0.0392	1.00
merck-T92393_s_at	---	---	-0.300	-2.09	0.0393	1.00
merck-NM_001040647_at	CRCP	NM_014478 NM_001040647 NM_0010(	-0.136	-2.09	0.0393	1.00
merck-NM_144676_at	TMED6	NM_144676	-0.221	-2.09	0.0394	1.00
merck-U51706_at	---	---	-0.313	-2.09	0.0394	1.00
merck2-AA576624_at	TMEM66	NM_016127	0.101	2.09	0.0394	1.00
merck2-BU175285_s_at	PTGES3	NM_006601	0.149	2.09	0.0394	1.00
merck2-NM_030908_s_at	---	---	0.134	2.09	0.0394	1.00
merck2-BM669527_at	---	---	0.147	2.09	0.0394	1.00
merck-AK093152_s_at	CCDC91	NM_018318	0.185	2.09	0.0394	1.00
merck-BC034812_at	---	XR_110131 XR_111394 XR_114702	0.236	2.09	0.0395	1.00
merck-AF380424_at	DLEU2	NR_002612	-0.145	-2.09	0.0395	1.00
merck-AF086545_a_at	IFI16	NM_005531	-0.140	-2.09	0.0395	1.00
merck-G36642_at	---	---	-0.137	-2.09	0.0396	1.00
merck-NM_198213_at	OASL	NM_003733 NM_198213	-0.354	-2.09	0.0396	1.00
merck-BC031864_at	LOC100507330	XR_110379 XR_111304 XR_114660	-0.130	-2.09	0.0396	1.00
merck2-BX648643_at	KDM5D	BX648643	-0.509	-2.09	0.0396	1.00
merck-hCT1830086_at	---	---	-0.0746	-2.09	0.0396	1.00
merck2-NM_001098845_s_at	---	---	-0.209	-2.09	0.0396	1.00
merck-BF373267_at	---	AC008577	0.132	2.09	0.0397	1.00
merck-DB088616_s_at	LARP4B	NM_015155	-0.145	-2.09	0.0397	1.00
merck-CA445255_at	---	---	-0.232	-2.09	0.0397	1.00
merck2-BC093952_at	CWC22	NM_020943	0.172	2.09	0.0397	1.00
merck-BM675973_a_at	BAK1	NM_001188	-0.181	-2.09	0.0398	1.00
merck2-L42806_at	---	L42806	0.170	2.08	0.0398	1.00
merck2-NM_197954_at	CLEC7A	NM_197947 NM_022570 NM_197948 N	-0.232	-2.08	0.0398	1.00
merck-AF091236_x_at	SLC25A6	NM_001636	0.120	2.08	0.0399	1.00
merck-ENST00000355278_at	---	BC008359	0.183	2.08	0.0399	1.00
merck2-BG291729_at	---	---	0.0755	2.08	0.0399	1.00
merck2-DA413429_at	LOC100127891	XR_108812 XR_110748 XR_113053 XR	-0.133	-2.08	0.0399	1.00
merck2-NM_173041_at	ICK	BC035807	-0.102	-2.08	0.0399	1.00
merck2-CMV_UL85_at	---	---	0.128	2.08	0.0399	1.00
merck-NM_004521_s_at	KIF5B	NM_004521	-0.143	-2.08	0.0400	1.00
merck-NM_024897_at	PAQR6	NM_024897 NM_198406	-0.288	-2.08	0.0400	1.00
merck-XM_062614_at	---	BN000483	-0.123	-2.08	0.0400	1.00
merck2-NM_172108_at	KCNQ2	NM_172107 NM_172106 NM_004518 N	-0.141	-2.08	0.0400	1.00
merck-AK057018_at	---	AK057018	-0.135	-2.08	0.0401	1.00
merck-AK024332_at	---	AK024332	0.154	2.08	0.0401	1.00
merck2-NM_001079530_at	---	---	-0.0924	-2.08	0.0401	1.00
merck2-BM763807_a_at	EXOSC3	NM_016042 NM_001002269	-0.127	-2.08	0.0401	1.00
merck-hCT1970919_at	---	---	-0.154	-2.08	0.0401	1.00
merck-NM_016521_at	TFDP3	NM_016521	0.217	2.08	0.0402	1.00
merck2-AY739713_at	TXLNG	NM_018360 NM_001168683	0.219	2.08	0.0402	1.00
merck2-AY743663_at	C10orf46	NM_153810	-0.142	-2.08	0.0402	1.00
merck2-AA805639_at	CD72	NM_001782	0.219	2.08	0.0403	1.00
merck-AK095512_s_at	PALLD	NM_001166108 NM_016081 NM_0011(	-0.136	-2.08	0.0403	1.00
merck-NM_205858_at	NMB	NM_021077 NM_205858	-0.196	-2.08	0.0403	1.00
merck-AA161183_at	---	---	-0.108	-2.08	0.0403	1.00
merck-AF432220_a_at	PSIP1	NM_033222 NM_001128217	0.177	2.08	0.0403	1.00
merck2-BQ004375_at	VCAN	NM_004385 NM_001126336 NM_0011(	-0.101	-2.08	0.0403	1.00
merck2-BC009180_at	MKKS	NM_018848 NM_170784	-0.210	-2.08	0.0404	1.00
merck2-AA806102_at	TTC39C	NM_001135993 NM_153211	0.121	2.08	0.0404	1.00
merck-BF206970_at	---	---	-0.163	-2.08	0.0404	1.00
merck-NM_006979_a_at	SLC39A7	NM_006979 NM_001077516	-0.174	-2.08	0.0404	1.00
merck-XM_212634_at	---	NG_004693	-0.0952	-2.08	0.0404	1.00
merck-NM_183233_at	SLC22A18	NM_002555 NM_183233	-0.217	-2.08	0.0405	1.00
merck-ENST00000374949_s_at	HLA-DQA1	NM_002122	0.577	2.08	0.0405	1.00
merck-NM_007270_s_at	FKBP9 FKBP9L	NM_007270 NR_027339 NR_027340 N	-0.203	-2.08	0.0405	1.00
merck-CR624203_a_at	TLE4	NM_007005	-0.149	-2.08	0.0406	1.00
merck-BX648430_s_at	---	AF130085	-0.115	-2.08	0.0406	1.00
merck2-AK094618_at	ZNF529	NR_027239	0.205	2.08	0.0406	1.00
merck-CR608017_at	---	CR608017	0.287	2.08	0.0407	1.00
merck-AB209420_at	MLLT4	NM_001040000	-0.0914	-2.08	0.0407	1.00
merck-AK024928_at	---	AK024928	0.178	2.08	0.0407	1.00
merck-AK091285_at	---	---	0.161	2.08	0.0407	1.00
merck-NM_001039476_a_at	NPRL3	NM_001077350 NM_001039476	-0.295	-2.08	0.0407	1.00
merck-N79684_s_at	LOC100507493	XR_110882 XR_113132 XR_114149	0.187	2.07	0.0408	1.00
merck-VZV_OKA_ORF66_s_at	---	---	-0.0885	-2.07	0.0408	1.00
merck2-BG385872_at	KCNQ1	NM_000218 NM_181798	-0.119	-2.07	0.0408	1.00
merck-AI655831_a_at	PPFIA1	NM_177423 NM_003626	-0.108	-2.07	0.0409	1.00
merck-BU664767_at	---	AC087361	0.144	2.07	0.0409	1.00
merck-NM_001621_at	AHR	NM_001621	0.253	2.07	0.0409	1.00
merck-H50080_s_at	---	AL160262	0.334	2.07	0.0409	1.00
merck-BX648371_at	---	XR_108885 XR_113170 XR_114207	0.174	2.07	0.0409	1.00
merck-EB387580_s_at	---	AK092450	0.140	2.07	0.0409	1.00
merck-NM_002185_a_at	IL7R	NM_002185	0.151	2.07	0.0409	1.00
merck-NM_153201_s_at	HSPA8	NM_006597 NM_153201	0.125	2.07	0.0409	1.00
merck-NM_003309_at	TSPYL1	NM_003309	0.128	2.07	0.0409	1.00
merck2-BC040959_at	---	XR_109767 XR_115520	0.211	2.07	0.0409	1.00
merck-NM_017509_at	KLK15	NM_138563 NM_138564 NM_017509	-0.150	-2.07	0.0409	1.00
merck-G30809_at	---	---	-0.141	-2.07	0.0410	1.00
merck-NM_013447_at	EMR2	NM_013447 NM_152916 NM_152917 N	-0.189	-2.07	0.0410	1.00
merck-NM_000788_at	DCK	NM_000788	0.201	2.07	0.0410	1.00
merck-BU531790_at	---	---	-0.128	-2.07	0.0410	1.00
merck-NM_005642_at	TAF7	NM_005642	0.164	2.07	0.0410	1.00
merck2-EB388744_at	ADD3	NM_016824 NM_019903 NM_001121	0.145	2.07	0.0410	1.00
merck-NM_022719_at	DGCR14	NM_022719	-0.153	-2.07	0.0411	1.00
merck-NM_019117_at	KLHL4	NM_019117	0.152	2.07	0.0411	1.00
merck-DB294168_at	PPP6C	NM_001123355 NM_002721 NM_00112	0.0971	2.07	0.0411	1.00
merck-NM_005393_at	PLXNB3	NM_005393 NM_001163257	-0.133	-2.07	0.0411	1.00
merck2-BI869895_at	CMIP	NM_198390 NM_030629	-0.189	-2.07	0.0412	1.00
merck-CR626250_a_at	SMARCE1	NM_003079	0.0877	2.07	0.0412	1.00
merck-AK126280_at	IGHD	BC063384	0.449	2.07	0.0412	1.00

merck-M74777_a_at	DPP4	NM_001935	0.239	2.07	0.0412	1.00
merck-NM_003360_at	UGT8	NM_001128174 NM_003360	0.110	2.07	0.0412	1.00
merck-NM_052945_at	TNFRSF13C	NM_052945	0.149	2.07	0.0412	1.00
merck-AY005981_at	SEPT6	NM_145799	0.170	2.07	0.0413	1.00
merck-AF425244_at	RAET1K	NR_024045	-0.0828	-2.07	0.0413	1.00
merck2-ENST00000367942_at	ATF6	NM_007348	-0.166	-2.07	0.0413	1.00
merck-NM_014883_a_at	FAM13A	NM_014883 NM_001015045	0.268	2.07	0.0413	1.00
merck-NM_016651_at	DACT1	NM_016651 NM_001079520	0.416	2.07	0.0413	1.00
merck-ENST00000377964_at	RECK	BC060806	-0.184	-2.07	0.0414	1.00
merck2-BX648519_at	ERP44	NM_015051	0.122	2.07	0.0414	1.00
merck-XM_929383_at	---	AC009492	-0.117	-2.07	0.0414	1.00
merck-ENST00000369655_at	---	XR_110456 XR_111116 XR_114431	-0.150	-2.07	0.0414	1.00
merck-BF977217_at	---	---	-0.0993	-2.07	0.0415	1.00
merck-NM_001004759_at	OR51T1	NM_001004759	-0.122	-2.07	0.0415	1.00
merck-BC047780_at	LOC100329109	NR_033248	-0.157	-2.07	0.0415	1.00
merck2-AF062248_at	---	AF062248	-0.117	-2.07	0.0416	1.00
merck-NM_030776_at	ZBP1	NM_030776 NM_001160417 NM_00116	-0.205	-2.07	0.0416	1.00
merck-NM_012067_s_at	AKR7A3	NM_012067	-0.109	-2.07	0.0416	1.00
merck-DA036409_a_at	CYP2C8	NM_000770 NM_001198853 NM_00119	-0.131	-2.07	0.0416	1.00
merck2-BM799863_a_at	SEL1L3	NM_015187	0.188	2.07	0.0416	1.00
merck-XM_938742_at	---	---	0.174	2.07	0.0417	1.00
merck-AI129830_s_at	RPL22	NM_000983	0.147	2.07	0.0417	1.00
merck-BC015311_a_at	KIF9	NM_022342 NM_182902 NM_00113487	-0.119	-2.07	0.0417	1.00
merck-NM_152310_at	ELOVL3	NM_152310	-0.173	-2.06	0.0417	1.00
merck-AK128754_at	---	AK128754	0.119	2.06	0.0418	1.00
merck-BC060766_at	SLC2A14	AL832448	-0.104	-2.06	0.0418	1.00
merck-NM_001788_s_at	SEPT7	NM_001788 NM_001011553	0.112	2.06	0.0418	1.00
merck-NM_152751_s_at	BEND7	NM_152751	-0.134	-2.06	0.0419	1.00
merck2-NM_178153_at	DCX	NM_000555 NM_178152 NM_178153 N	0.345	2.06	0.0419	1.00
merck-ENST00000361066_a_at	GSTM3	NM_000849 NR_024537	0.243	2.06	0.0419	1.00
merck-BQ924201_at	---	---	-0.119	-2.06	0.0419	1.00
merck-T80060_s_at	---	NG_009055	-0.162	-2.06	0.0420	1.00
merck-NM_017681_s_at	NUP62CL	NM_017681 NR_033676	0.291	2.06	0.0420	1.00
merck-NM_021794_at	ADAM30	NM_021794	0.118	2.06	0.0420	1.00
merck2-BU147178_at	WDR6	NM_018031	0.150	2.06	0.0420	1.00
merck-ENST00000288425_at	BAHCC1	NM_001080519	-0.160	-2.06	0.0420	1.00
merck2-NM_000172_a_at	GNAT1	NM_144499 NM_000172	-0.0941	-2.06	0.0420	1.00
merck-NM_001037335_at	PRIC285	NM_001037335 NM_033405	-0.175	-2.06	0.0420	1.00
merck-NM_007139_at	ZNF92	NM_007139 NM_152626	0.342	2.06	0.0420	1.00
merck-BC039434_at	---	BC039434	0.289	2.06	0.0420	1.00
merck2-BE006861_x_at	---	---	-0.136	-2.06	0.0420	1.00
merck-NM_002128_s_at	HMGB1	NM_002128	0.187	2.06	0.0421	1.00
merck-NM_014572_a_at	LATS2	NM_014572	-0.122	-2.06	0.0421	1.00
merck-NM_001004349_a_at	HLA-L	NR_027822	0.133	2.06	0.0421	1.00
merck2-BG385797_at	VPS53	NM_001128159	-0.167	-2.06	0.0421	1.00
merck-NM_001154_at	ANXA5	NM_001154	-0.111	-2.06	0.0421	1.00
merck-AK025286_at	---	AK025286	-0.176	-2.06	0.0421	1.00
merck2-ENST00000304187_x_at	---	---	0.234	2.06	0.0421	1.00
merck-AK131312_a_at	RCOR3	NM_001136223 NM_001136225 NM_01	-0.262	-2.06	0.0422	1.00
merck-NM_000419_at	ITGA2B	NM_000419	-0.445	-2.06	0.0422	1.00
merck2-BF979936_s_at	HEATR8 HEATR8-TTC4	NM_001039464 NR_026782 NR_03764	-0.143	-2.06	0.0422	1.00
merck-NM_024549_s_at	TCTN1	NM_001082538 NM_001082537 NM_02	0.150	2.06	0.0423	1.00
merck-ENST00000372602_s_at	TMSB15B	NM_194324	0.219	2.06	0.0423	1.00
merck2-NM_001098173_a_at	PRDM7	NM_001098173 NM_052996	-0.141	-2.06	0.0424	1.00
merck2-NM_022893_at	BCL11A	NM_022893	0.198	2.06	0.0424	1.00
merck-NM_001039091_at	PRPS2	NM_001039091 NM_002765	0.222	2.06	0.0424	1.00
merck2-DQ786228_at	---	DQ786228	-0.131	-2.06	0.0425	1.00
merck2-BC051268_at	FUCA2	NM_032020	-0.168	-2.06	0.0425	1.00
merck-NM_001040167_s_at	LFNG	NM_001040167 NM_001166355 NM_00	-0.104	-2.06	0.0425	1.00
merck-NM_018275_s_at	C7orf43	NM_018275	-0.173	-2.06	0.0426	1.00
merck-NM_001010853_s_at	PM20D2	NM_001010853	0.229	2.06	0.0426	1.00
merck-NM_207005_at	USF1	NM_007122 NM_207005	-0.229	-2.06	0.0427	1.00
merck-BC012447_a_at	SP110	NM_004509 NM_004510 NM_080424 N	-0.119	-2.06	0.0427	1.00
merck-NM_005132_at	REC8	NM_005132 NM_001048205	-0.269	-2.06	0.0427	1.00
merck2-AB051826_at	RHOU	NM_021205	-0.160	-2.05	0.0428	1.00
merck2-BU603313_at	BCAS2	NM_005872	0.0930	2.05	0.0428	1.00
merck-NM_020417_at	TBX20	NM_001077653 NM_001166220	0.114	2.05	0.0428	1.00
merck-BQ957607_a_at	TNFAIP2	NM_006291	-0.255	-2.05	0.0428	1.00
merck2-hCT1640765.3_at	---	NG_010517	-0.134	-2.05	0.0428	1.00
merck-AF103907_a_at	PCA3	NR_015342	-0.121	-2.05	0.0428	1.00
merck2-BU196243_at	---	---	-0.116	-2.05	0.0429	1.00
merck2-BC127705_at	TMEM220	NM_001004313	0.124	2.05	0.0429	1.00
merck-NM_198989_a_at	DLEU7	AK126830	-0.177	-2.05	0.0429	1.00
merck2-S67637_x_at	---	---	0.348	2.05	0.0430	1.00
merck2-BG536877_at	S1PR1	NM_001400	0.207	2.05	0.0430	1.00
merck-ENST00000325589_at	RABGAP1L	CR627374	0.113	2.05	0.0430	1.00
merck2-NM_205843_at	NFIC	NM_005597 NM_205843	-0.113	-2.05	0.0430	1.00
merck-BG745188_at	---	---	-0.118	-2.05	0.0430	1.00
merck2-DA660473_at	---	DQ068066	-0.155	-2.05	0.0431	1.00
merck2-AL832013_at	ANAPC1	AL832013	0.117	2.05	0.0431	1.00
merck-XM_929995_at	---	---	-0.0970	-2.05	0.0431	1.00
merck-NM_152578_at	FMR1NB	NM_152578	0.115	2.05	0.0431	1.00
merck2-DQ786327_at	---	DQ786327	0.209	2.05	0.0431	1.00
merck-CB048235_at	---	---	0.133	2.05	0.0432	1.00
merck-ENST00000357931_a_at	---	---	-0.336	-2.05	0.0432	1.00
merck-ENST00000278622_at	---	AF132199	0.239	2.05	0.0432	1.00
merck-BG716493_s_at	APLP2	NM_001642 NM_001142276 NM_00114	-0.0736	-2.05	0.0432	1.00
merck-AF264622_at	---	AF264622	-0.298	-2.05	0.0432	1.00
merck-AK123047_a_at	NR3C2	NM_000901 NM_001166104	0.154	2.05	0.0433	1.00
merck-NM_001001888_s_at	VCX VCX3A VCX3B	NM_013452 NM_016379 NM_0010018	-0.0961	-2.05	0.0433	1.00
merck-AW976243_at	BACH2	AJ271878	0.204	2.05	0.0433	1.00
merck-AB062477_at	---	AB062477	-0.138	-2.05	0.0433	1.00
merck-BC038721_at	LOC255025	NR_015400	0.109	2.05	0.0434	1.00
merck2-NM_004054_at	C3AR1	NM_004054	-0.234	-2.05	0.0434	1.00
merck2-BX648513_at	POLA1	BX648513	0.479	2.05	0.0434	1.00
merck-NM_018982_s_at	YIPF1	NM_018982 NR_036639 NR_036640	-0.0915	-2.05	0.0434	1.00
merck-NM_016107_at	ZFR	NM_016107	0.162	2.05	0.0434	1.00
merck2-NM_013394_at	FGF1	NM_000800 NM_033136 NM_033137 N	-0.0988	-2.05	0.0434	1.00
merck-NM_004529_at	MLLT3	NM_004529	0.142	2.05	0.0434	1.00
merck-ENST00000323920_at	---	NG_000957	-0.118	-2.05	0.0435	1.00
merck-NM_033150_at	COL2A1	NM_001844 NM_033150	-0.130	-2.05	0.0435	1.00
merck2-AK056080_at	LOC387647	NR_003930	-0.130	-2.05	0.0435	1.00
merck-NM_001194_at	HCN2	NM_001194	-0.132	-2.05	0.0435	1.00
merck2-BM468535_at	ZEB2	NM_014795 NM_001171653	-0.197	-2.05	0.0435	1.00
merck-RSE_00000399804_at	---	NG_025418	-0.137	-2.05	0.0436	1.00
merck-ENST00000380536_s_at	GPM6B	NM_001001995	0.190	2.05	0.0436	1.00
merck-NM_006706_at	TCERG1	NM_006706 NM_001040006	0.156	2.05	0.0436	1.00
merck-BF057089_s_at	---	AK095264	-0.243	-2.05	0.0436	1.00
merck2-EC495211_x_at	---	---	0.0567	2.05	0.0436	1.00
merck-ENST00000358967_at	NHLRC2	BC013743	0.156	2.05	0.0436	1.00
merck-ENST00000367603_a_at	HIVEP2	NM_006734	0.163	2.05	0.0436	1.00
merck-AK001851_s_at	QRSL1	NM_018292	0.209	2.05	0.0437	1.00
merck-NM_001005464_s_at	HIST2H3A HIST2H3C HIST2H3D	NM_001005464 NM_021059 NM_00112	-0.149	-2.05	0.0437	1.00
merck2-BC017339_at	THEM4	NM_053055	0.234	2.04	0.0437	1.00
merck-BC108287_a_at	ZNF516	NM_014643	-0.283	-2.04	0.0437	1.00
merck2-BG230607_x_at	PSAP	NM_002778 NM_001042465 NM_00104	-0.0612	-2.04	0.0438	1.00
merck2-XM_098264_s_at	LOC100128651	D25271	-0.114	-2.04	0.0438	1.00
merck-AV715889_s_at	SKP1 LOC728622	NM_006930 NM_170679 NR_036619	0.113	2.04	0.0438	1.00
merck-ENST00000373790_at	TAF1	NM_004606 NM_138923	0.127	2.04	0.0438	1.00
merck-AI421220_at	---	AL161785	-0.175	-2.04	0.0438	1.00
merck-NM_015327_at	SMG5	NM_015327	-0.169	-2.04	0.0438	1.00
merck-BX537586_at	STK17A	BC023508	0.153	2.04	0.0438	1.00
merck-BC050552_s_at	NISCH	BC050552	-0.114	-2.04	0.0438	1.00
merck-NM_016217_at	HECA	NM_016217	0.0710	2.04	0.0439	1.00
merck-CR621898_at	---	CR621898	0.142	2.04	0.0439	1.00
merck-AW852694_at	---	---	-0.0931	-2.04	0.0439	1.00
merck-ENST00000330493_at	---	XR_108331 XR_114966	-0.136	-2.04	0.0439	1.00
merck2-AK172753_at	PPP1R21	NM_001135629 NM_152994 NM_00119	0.103	2.04	0.0439	1.00
merck-NM_014731_at	ProSAPiP1	NM_014731	-0.131	-2.04	0.0439	1.00

merck-NM_003415_at	ZNF268	NM_003415 NM_001165881 NM_15294	0.176	2.04	0.0439	1.00
merck-ENST00000313566_at	AP4S1	NM_001128126	0.139	2.04	0.0440	1.00
merck2-AW665829_at	---	D00596	0.161	2.04	0.0440	1.00
merck2-NM_199127_s_at	---	---	-0.231	-2.04	0.0440	1.00
merck2-NM_007110_at	TEP1	NM_007110	-0.123	-2.04	0.0440	1.00
merck2-NM_001100874_at	SLC9B1	NM_001100874	-0.127	-2.04	0.0440	1.00
merck-NM_015137_at	EFR3A	NM_015137	0.153	2.04	0.0440	1.00
merck-NM_004566_at	PFKFB3	NM_004566 NM_001145443	-0.167	-2.04	0.0441	1.00
merck2-BX647290_at	---	BX647290	0.120	2.04	0.0441	1.00
merck-ENST00000373701_s_at	OGT	NM_181672 NM_181673	0.204	2.04	0.0441	1.00
merck-AF088004_at	---	AF088004	-0.170	-2.04	0.0441	1.00
merck-NM_003639_s_at	IKBKG	NM_001099857 NM_001099856 NM_00	-0.119	-2.04	0.0441	1.00
merck-NM_001013723_s_at	ZNRF2P1	NR_003502	-0.111	-2.04	0.0442	1.00
merck2-NM_017979_at	UNC45A	NM_018671 NM_001039675	-0.149	-2.04	0.0442	1.00
merck2-NM_001415_at	EIF2S3	NM_001415	0.0804	2.04	0.0442	1.00
merck2-AY346375_at	ZNF562 ZNF812	NM_001130031 NM_001130032 NM_01	-0.128	-2.04	0.0442	1.00
merck-AA860629_at	---	AP002755	-0.128	-2.04	0.0443	1.00
merck-BC002682_a_at	DUSP3	NM_004090	-0.146	-2.04	0.0443	1.00
merck-NM_003064_at	SLPI	NM_003064	-0.302	-2.04	0.0443	1.00
merck-NM_004783_at	TAOK2	NM_004783	-0.174	-2.04	0.0443	1.00
merck2-CR742385_at	NUP93	NM_014669	-0.143	-2.04	0.0443	1.00
merck2-NM_022073_at	EGLN3	NM_022073	0.243	2.04	0.0444	1.00
merck-AF195821_s_at	TCL6	AF195821	0.216	2.04	0.0445	1.00
merck-AK091013_at	LOC401188	AK091013	-0.145	-2.04	0.0445	1.00
merck2-BX348702_at	TLK1	NM_012290 NM_001136554 NM_00113	0.131	2.04	0.0445	1.00
merck-AI765698_at	---	AC080106	0.334	2.04	0.0445	1.00
merck-AK056007_s_at	---	AK056007	0.169	2.04	0.0445	1.00
merck-CK002033_at	---	---	-0.0979	-2.04	0.0445	1.00
merck-BQ927793_s_at	LOC100506119	XR_108951 XR_108954 XR_108952 XF	-0.200	-2.04	0.0446	1.00
merck-G65655_x_at	---	U73627	-0.178	-2.04	0.0446	1.00
merck-NM_178834_at	LAYN	NM_178834	-0.123	-2.04	0.0446	1.00
merck-DA395744_a_at	PDK2	NM_001199898	0.124	2.04	0.0446	1.00
merck-RSE_00000067763_at	---	NG_000929	0.108	2.04	0.0446	1.00
merck-NM_173077_at	CPO	NM_173077	0.162	2.04	0.0446	1.00
merck-NM_012217_at	TPSD1	NM_012217	-0.138	-2.04	0.0447	1.00
merck-NM_024760_s_at	TLE6	NM_001143986 NM_024760	-0.137	-2.04	0.0447	1.00
merck-NM_005337_at	NCKAP1L	NM_005337 NM_001184976	-0.133	-2.04	0.0447	1.00
merck-BF508564_at	---	AL365273	-0.172	-2.04	0.0447	1.00
merck-NM_016049_at	FAM158A	NM_016049	-0.133	-2.04	0.0447	1.00
merck-NM_000245_at	MET	NM_001127500 NM_000245	-0.122	-2.03	0.0447	1.00
merck2-BI025212_a_at	AGTPBP1	NM_015239	-0.131	-2.03	0.0448	1.00
merck-NM_000694_at	ALDH3B1	NM_000694 NM_001030010 NM_00116	-0.197	-2.03	0.0448	1.00
merck2-AA169659_s_at	---	---	-0.155	-2.03	0.0448	1.00
merck-hCT1837774.1_at	---	AL672206	0.518	2.03	0.0448	1.00
merck-NM_005170_at	ASCL2	NM_005170	-0.175	-2.03	0.0448	1.00
merck-NM_015866_at	PRDM2	NM_015866 NM_001007257	0.108	2.03	0.0449	1.00
merck-NM_020856_at	TSHZ3	NM_020856	-0.186	-2.03	0.0449	1.00
merck-NM_052889_s_at	CARD16	NM_001017534 NM_052889	-0.274	-2.03	0.0450	1.00
merck-NM_080552_at	SLC32A1	NM_080552	-0.0991	-2.03	0.0450	1.00
merck2-AL543631_at	TSPAN13	NM_014399	0.225	2.03	0.0450	1.00
merck-NM_015690_at	STK36	NM_015690	-0.178	-2.03	0.0450	1.00
merck-AF330042_at	---	AF330042	0.130	2.03	0.0450	1.00
merck-XM_925901_s_at	---	AC217322	-0.134	-2.03	0.0450	1.00
merck-NM_000355_at	TCN2	NM_000355 NM_001184726	-0.250	-2.03	0.0451	1.00
merck-ENST00000368374_s_at	GBA GBAP1	NM_000157 NM_001005741 NM_0010(	-0.170	-2.03	0.0451	1.00
merck2-VZV_OKA_O15_up_at	---	---	-0.129	-2.03	0.0451	1.00
merck-XM_945132_at	---	---	-0.126	-2.03	0.0451	1.00
merck-BM713507_at	---	AC000117	-0.0858	-2.03	0.0451	1.00
merck-NM_000390_s_at	CHM	NM_000390	0.273	2.03	0.0451	1.00
merck-NM_001007_x_at	RPS4X	NM_001007	0.0557	2.03	0.0451	1.00
merck2-BI192949_at	RERE	NM_012102 NM_001042681 NM_00104	-0.121	-2.03	0.0452	1.00
merck2-NM_030780_at	SLC25A32	NM_030780	0.190	2.03	0.0452	1.00
merck-NM_000509_at	FGG	NM_000509	-0.162	-2.03	0.0452	1.00
merck-AX772926_at	SERPINE3	NM_001101320	0.108	2.03	0.0452	1.00
merck-NM_138712_s_at	PPARG	NM_138712 NM_015869 NM_138711 N	-0.163	-2.03	0.0453	1.00
merck-NM_207383_at	FLJ42289	NR_028139 NR_028140	-0.143	-2.03	0.0453	1.00
merck-NM_002769_s_at	PRSS1	NM_002769	0.0905	2.03	0.0453	1.00
merck2-NM_013289_at	KIR3DL1	NM_013289	-0.277	-2.03	0.0453	1.00
merck2-BX647795_at	MIPOL1	BX647795	0.0997	2.03	0.0453	1.00
merck-BC047016_a_at	FCHSD1	NM_033449	-0.162	-2.03	0.0453	1.00
merck-ENST00000314117_at	RELL1	NM_001085399	-0.137	-2.03	0.0453	1.00
merck2-AB209992_at	DSP	NM_004415 NM_001008844	0.382	2.03	0.0454	1.00
merck2-BP197851_x_at	---	---	0.132	2.03	0.0454	1.00
merck-NM_001004320_at	AGMO	NM_001004320	-0.144	-2.03	0.0454	1.00
merck-AK126693_at	---	AK126693	-0.129	-2.03	0.0454	1.00
merck-ENST00000327832_x_at	LOC283911	AL833480	-0.126	-2.03	0.0455	1.00
merck2-BX107168_at	---	AF321561	0.144	2.03	0.0455	1.00
merck-NM_003410_a_at	ZFX	NM_003410 NM_001178084 NM_00117	0.250	2.03	0.0456	1.00
merck-AF339767_at	---	---	-0.194	-2.03	0.0456	1.00
merck-M13994_a_at	BCL2	NM_000633	0.178	2.03	0.0456	1.00
merck-AW027928_at	---	NG_008295	-0.128	-2.03	0.0456	1.00
merck-AW105183_at	---	---	0.161	2.03	0.0456	1.00
merck-NM_001549_at	IFIT3	NM_001549 NM_001031683	-0.377	-2.03	0.0457	1.00
merck2-BF697947_at	---	---	-0.114	-2.03	0.0457	1.00
merck-XM_108467_at	---	AC024367	-0.130	-2.03	0.0457	1.00
merck-AK091929_a_at	ZFP106	NM_022473	-0.156	-2.03	0.0457	1.00
merck-hCT1951691.1_x_at	---	NG_010914	0.166	2.03	0.0457	1.00
merck-AK097116_at	---	AK097116	-0.128	-2.02	0.0458	1.00
merck-BC018008_s_at	---	BC018008	-0.102	-2.02	0.0458	1.00
merck-NM_021244_a_at	RRAGD	NM_021244	-0.137	-2.02	0.0458	1.00
merck-BC070228_a_at	C7orf13	NR_026865	-0.127	-2.02	0.0458	1.00
merck2-AI283000_at	NARS2	NM_024678 NR_027479	0.181	2.02	0.0458	1.00
merck2-BF680332_x_at	---	---	-0.125	-2.02	0.0459	1.00
merck-NM_173502_at	PRSS36	NM_173502	-0.121	-2.02	0.0460	1.00
merck-ENST00000378484_at	---	AF090884	-0.129	-2.02	0.0460	1.00
merck-XM_932008_at	---	---	0.137	2.02	0.0460	1.00
merck-NM_138362_at	FAM104B	NM_138362 NM_001166699 NR_03072	0.156	2.02	0.0461	1.00
merck-NM_025135_at	FHOD3	NM_025135	0.152	2.02	0.0461	1.00
merck-NM_152467_at	KLHL10	NM_152467	-0.168	-2.02	0.0461	1.00
merck2-M10051_at	INSR	NM_000208 NM_001079817	-0.150	-2.02	0.0461	1.00
merck-AK054661_a_at	PCDH9	NM_203487 NM_020403	0.304	2.02	0.0461	1.00
merck2-NM_006187_at	OAS3	NM_006187	-0.374	-2.02	0.0462	1.00
merck-NM_001062_at	TCN1	NM_001062	-0.304	-2.02	0.0462	1.00
merck2-AY219181_at	PKHD1L1	NM_177531	0.207	2.02	0.0462	1.00
merck-NM_101395_s_at	DYRK1A	NM_001396 NM_130436 NM_101395 N	0.110	2.02	0.0462	1.00
merck-NM_015087_at	SPG20	NM_015087 NM_001142296 NM_00114	0.228	2.02	0.0462	1.00
merck-BI094070_at	---	---	-0.0871	-2.02	0.0463	1.00
merck2-NM_018404_at	ADAP2	NM_018404	-0.199	-2.02	0.0463	1.00
merck-ENST00000375762_at	GTDC1	AK126774	0.114	2.02	0.0463	1.00
merck-NM_006766_at	KAT6A	NM_001099412 NM_001099413 NM_00	-0.0942	-2.02	0.0463	1.00
merck2-AB055660_at	SHROOM3	NM_020859	-0.112	-2.02	0.0463	1.00
merck2-DA738256_at	KTN1	NM_182926 NM_001079521 NM_00107	-0.128	-2.02	0.0463	1.00
merck2-NM_033254_at	---	---	0.147	2.02	0.0464	1.00
merck-NM_002844_at	PTPRK	NM_001135648 NM_002844	0.331	2.02	0.0464	1.00
merck2-BC033793_at	---	XR_109079 XR_109080 XR_109081 XF	0.132	2.02	0.0464	1.00
merck-AK094441_at	---	AK094441	0.179	2.02	0.0464	1.00
merck2-AW270829_a_at	RAD51C	NM_058216 NM_002876	-0.144	-2.02	0.0464	1.00
merck2-BX647755_at	GABPA	NM_002040 NM_001197297	0.206	2.02	0.0464	1.00
merck-AK021429_a_at	SH3RF1	NM_020870	-0.131	-2.02	0.0464	1.00
merck-AB209463_s_at	CSNK1D	NM_001893 NM_139062	-0.143	-2.02	0.0464	1.00
merck-BC017745_a_at	NUFIP1	NM_012345	0.128	2.02	0.0464	1.00
merck-AL834345_at	MOSPD2	AL834345	0.186	2.02	0.0465	1.00
merck2-AA825724_at	GPR113	NM_001145169 NM_153835	-0.0847	-2.02	0.0465	1.00
merck-NM_004614_s_at	TK2	NM_004614 NM_001172644 NM_00117	-0.120	-2.02	0.0465	1.00
merck-CR625009_at	---	CR625009	0.137	2.02	0.0465	1.00
merck-BC038580_at	---	BC038580	0.261	2.02	0.0465	1.00
merck2-BX436262_at	SULT4A1	NM_014351	-0.112	-2.02	0.0466	1.00
merck-AK097986_at	LOC283745	AK097986	-0.162	-2.02	0.0466	1.00
merck-NM_001831_at	CLU	NM_001831 NM_203339 NM_00117113	0.157	2.02	0.0466	1.00

merck-NM_004063_at	CDH17	NM_004063 NM_001144663	-0.198	-2.02	0.0466	1.00
merck2-AF080216_at	BCL11A	NM_018014 NM_138559	0.196	2.02	0.0466	1.00
merck-NM_145175_s_at	FAM84A	NM_145175	-0.129	-2.02	0.0466	1.00
merck2-NM_024874_at	KIAA0319L	NM_024874	-0.185	-2.02	0.0467	1.00
merck2-BC030787_at	OFD1	NM_003611	0.185	2.02	0.0467	1.00
merck-AV724728_at	---	AC009331	0.144	2.02	0.0467	1.00
merck-NC_001405_ORF_1036_s_at	---	---	-0.115	-2.02	0.0467	1.00
merck-NM_001662_at	ARF5	NM_001662	-0.162	-2.02	0.0468	1.00
merck2-CR609492_at	---	AF067136	-0.220	-2.02	0.0468	1.00
merck-ENST00000360812_a_at	TTTY2 TTTY2B	NR_001536 NR_003590	-0.145	-2.01	0.0468	1.00
merck-T98486_s_at	---	---	0.174	2.01	0.0468	1.00
merck-NM_005860_at	FSTL3	NM_005860	-0.135	-2.01	0.0469	1.00
merck2-NM_153231_at	ZNF550	BX538032	0.203	2.01	0.0469	1.00
merck2-BG704181_a_at	SEPT15	NM_004261 NM_203341	0.181	2.01	0.0469	1.00
merck-BC110332_a_at	ZNF507	NM_001136156 NM_014910	0.186	2.01	0.0469	1.00
merck-NM_018120_at	ARMC1	NM_018120	0.102	2.01	0.0469	1.00
merck-H00927_at	---	---	-0.0734	-2.01	0.0470	1.00
merck-AI698687_at	---	---	0.0949	2.01	0.0471	1.00
merck-NM_012143_s_at	TFIP11	NM_001008697 NM_012143	-0.162	-2.01	0.0471	1.00
merck2-AK075393_s_at	CTSB	NM_001908 NM_147780 NM_147781 N	-0.0860	-2.01	0.0471	1.00
merck2-NM_005456_s_at	---	---	-0.285	-2.01	0.0472	1.00
merck-NM_024007_a_at	EBF1	NM_024007	0.224	2.01	0.0472	1.00
merck-DV080308_at	DNAH12	NM_178504	-0.179	-2.01	0.0472	1.00
merck-ENST00000322446_a_at	EIF2S3	NM_001415	0.144	2.01	0.0472	1.00
merck2-BE222032_at	---	AJ336618	-0.181	-2.01	0.0472	1.00
merck-ENST00000356030_at	---	AF118064	0.212	2.01	0.0472	1.00
merck-BM977356_at	---	---	-0.123	-2.01	0.0472	1.00
merck2-BF965720_x_at	---	---	-0.225	-2.01	0.0473	1.00
merck-AK098783_at	FLJ25917	AK098783	0.220	2.01	0.0473	1.00
merck2-NM_005826_at	HNRNPR	NM_001102398 NM_005826 NM_0011C	0.0982	2.01	0.0473	1.00
merck-AL833235_at	SLC9A7P1	NR_033801	-0.160	-2.01	0.0473	1.00
merck-NM_080664_s_at	C14orf126	NM_080664	0.159	2.01	0.0474	1.00
merck-NM_006417_at	IFI44	NM_006417	-0.457	-2.01	0.0474	1.00
merck-NM_030824_at	ZNF442	NM_030824	0.170	2.01	0.0474	1.00
merck-NM_001039674_x_at	USP6	NM_004505	0.128	2.01	0.0474	1.00
merck2-ENST00000262033_at	---	---	0.117	2.01	0.0474	1.00
merck-NM_012121_at	CDC42EP4	NM_012121	-0.131	-2.01	0.0474	1.00
merck-NM_173572_at	C10orf93	NM_173572	-0.101	-2.01	0.0474	1.00
merck-NM_017857_at	SSH3	NM_017857	-0.179	-2.01	0.0475	1.00
merck2-AK057568_at	---	---	0.200	2.01	0.0476	1.00
merck-NM_145064_at	STAC3	NM_145064	-0.215	-2.01	0.0477	1.00
merck-BU521288_s_at	NUDT3 RPS10-NUDT3	NM_006703 NM_001202470	-0.137	-2.01	0.0477	1.00
merck-BC009808_at	NBR1	BC009808	-0.156	-2.01	0.0477	1.00
merck-AK026670_a_at	METTL4	NM_022840	0.202	2.01	0.0477	1.00
merck-BE737112_at	---	---	-0.173	-2.01	0.0477	1.00
merck2-NM_001103169_a_at	AADACL3	NM_001103170 NM_001103169	-0.145	-2.01	0.0477	1.00
merck-AK057104_s_at	LOC100294145	NR_037177 NR_037178 XR_110964 XF	0.203	2.01	0.0478	1.00
merck-NM_002300_s_at	LDHB	NM_002300 NM_001174097	0.133	2.01	0.0478	1.00
merck2-AK027825_at	SLC38A1	NM_030674 NM_001077484	0.160	2.01	0.0478	1.00
merck2-NM_018324_at	OLAH	NM_018324 NM_001039702	-0.159	-2.01	0.0478	1.00
merck-NM_032038_at	SPNS1	NM_032038 NM_001142448 NM_00114	-0.137	-2.01	0.0478	1.00
merck2-W02883_at	KPNB1	L38951	-0.111	-2.00	0.0479	1.00
merck-NM_203472_at	SELS	NM_203472	-0.230	-2.00	0.0479	1.00
merck2-AI247169_x_at	---	---	-0.139	-2.00	0.0479	1.00
merck2-AW382852_at	SF3B1	NM_012433	-0.107	-2.00	0.0480	1.00
merck2-BQ218842_at	---	---	-0.0809	-2.00	0.0480	1.00
merck2-BQ889662_at	IGFBP5	NM_000599	0.164	2.00	0.0480	1.00
merck-BC039828_a_at	SMC6	NM_001142286 NM_024624	0.156	2.00	0.0480	1.00
merck2-NM_004867_at	ITM2A	NM_004867 NM_001171581	0.211	2.00	0.0480	1.00
merck2-CD250161_at	---	---	0.107	2.00	0.0480	1.00
merck-AK090842_at	---	CR597563	-0.181	-2.00	0.0480	1.00
merck-NM_018101_at	CDCA8	NM_018101	-0.206	-2.00	0.0480	1.00
merck-XM_940076_at	---	---	-0.122	-2.00	0.0481	1.00
merck-NM_001010844_s_at	IRAK1BP1	NM_001010844	0.153	2.00	0.0481	1.00
merck-AW263638_a_at	PRKD1	NM_002742	-0.179	-2.00	0.0482	1.00
merck-BX093353_at	---	AL133167	0.155	2.00	0.0482	1.00
merck2-BP331530_a_at	RRBP1	NM_001042576 NM_004587	-0.140	-2.00	0.0482	1.00
merck-NM_032814_a_at	RNFT2	NM_001109903 NM_032814	0.183	2.00	0.0482	1.00
merck2-BC038724_at	REEP3	NM_001001330	-0.262	-2.00	0.0482	1.00
merck-BC040914_at	---	BC040914	0.288	2.00	0.0482	1.00
merck2-AF025304_at	EPHB2	NM_017449 NM_004442	-0.162	-2.00	0.0483	1.00
merck2-NM_024587_at	TMEM53	NM_024587	-0.132	-2.00	0.0483	1.00
merck-AB231705_at	LOC644277	XR_110541 XR_114547	-0.148	-2.00	0.0483	1.00
merck2-DQ891344_s_at	---	---	0.283	2.00	0.0483	1.00
merck-NM_015386_at	COG4	NM_015386 NM_001195139	-0.220	-2.00	0.0483	1.00
merck-AV715015_s_at	CNR2	EU517121	0.168	2.00	0.0484	1.00
merck2-AI654832_at	---	---	-0.170	-2.00	0.0484	1.00
merck-NM_005186_s_at	CAPN1	NM_001198868 NM_005186 NM_0011S	-0.169	-2.00	0.0484	1.00
merck-AF162278_a_at	POU2F3	NM_014352	0.167	2.00	0.0485	1.00
merck-NM_016310_at	POLR3K	NM_016310	-0.172	-2.00	0.0485	1.00
merck-NM_032622_at	---	---	0.101	2.00	0.0485	1.00
merck-AF355799_a_at	DEFT1P DEFT1P2	NR_036686 NR_036687	-0.148	-2.00	0.0486	1.00
merck-NM_032350_s_at	C7orf50	NM_032350 NM_001134395 NM_00113	-0.146	-2.00	0.0486	1.00
merck-NM_017539_at	DNAH3	NM_017539	0.136	2.00	0.0486	1.00
merck-BC063531_a_at	SIN3B	NM_015260	-0.192	-2.00	0.0486	1.00
merck-BX647868_at	PHC3	NM_024947	0.173	2.00	0.0486	1.00
merck2-BE811240_s_at	---	---	-0.0830	-2.00	0.0487	1.00
merck-XM_943067_at	---	AY952890	-0.0983	-2.00	0.0487	1.00
merck-BC005286_a_at	EPM2A	NM_005670	0.175	2.00	0.0487	1.00
merck2-BX537483_at	ITGA6	NM_001079818 NM_000210	0.157	2.00	0.0488	1.00
merck-ENST00000244221_a_at	PAIP2B	NM_020459	0.148	2.00	0.0488	1.00
merck-NM_152570_s_at	LINGO2	NM_152570	-0.172	-2.00	0.0488	1.00
merck2-AK097700_at	LOC146880	NR_027487	-0.198	-2.00	0.0488	1.00
merck-NM_001365_at	DLG4	NM_001365 NM_001128827	-0.145	-2.00	0.0488	1.00
merck-DB074303_at	---	---	-0.138	-2.00	0.0488	1.00
merck-NM_152773_at	TCTEX1D2	NM_152773	-0.111	-2.00	0.0488	1.00
merck-NM_005032_at	PLS3	NM_005032 NM_001136025 NM_00117	0.181	2.00	0.0489	1.00
merck-XM_496769_at	---	AK123308	-0.109	-2.00	0.0489	1.00
merck2-AY687925_s_at	EEF1G	NM_001404	0.113	2.00	0.0489	1.00
merck-NM_002424_at	MMP8	NM_002424	-0.408	-2.00	0.0489	1.00
merck-NM_182621_at	FAM159A	BC041608	0.218	2.00	0.0489	1.00
merck-ENST00000326189_at	LOC646976	AK096082	-0.149	-2.00	0.0489	1.00
merck2-AA001222_at	---	---	0.174	2.00	0.0489	1.00
merck-ENST00000379938_at	RREB1	NM_001003699 NM_001168344 NM_0C	-0.0840	-2.00	0.0490	1.00
merck-DA100265_a_at	PTPRZ1	NM_002851	-0.115	-2.00	0.0490	1.00
merck2-Rota_P_s3_s_at	---	---	-0.0744	-2.00	0.0490	1.00
merck-NR_002748_x_at	SNORD45B	NR_002748	0.333	1.99	0.0490	1.00
merck2-CB962172_x_at	---	---	-0.0866	-1.99	0.0490	1.00
merck-NM_018068_s_at	PIWIL2	NM_001135721 NM_018068	-0.199	-1.99	0.0490	1.00
merck-NM_004747_at	DLG5	NM_004747	0.171	1.99	0.0490	1.00
merck-ENST00000342371_x_at	OR7E37P MGC72080	NR_002163 NR_002822	-0.153	-1.99	0.0490	1.00
merck-AK023235_at	EXOG	NM_005107 NM_001145464	0.171	1.99	0.0490	1.00
merck-CR593570_a_at	GPR107	NM_001136557 NM_001136558 NM_02	-0.193	-1.99	0.0490	1.00
merck-NM_014368_at	LHX6	NM_014368 NM_199160	0.212	1.99	0.0491	1.00
merck-AF147360_at	---	AF147360	0.433	1.99	0.0491	1.00
merck-NM_145029_a_at	C6orf136	NM_001109938 NM_145029 NM_00116	-0.194	-1.99	0.0491	1.00
merck2-BX647602_at	C5orf42	NM_023073	-0.115	-1.99	0.0491	1.00
merck-AK026301_a_at	RPS6KA6	NM_014496	0.164	1.99	0.0491	1.00
merck-NM_000803_at	FOLR2	NM_000803 NM_001113534 NM_00111	-0.211	-1.99	0.0491	1.00
merck-BC008462_s_at	---	BC008462	-0.180	-1.99	0.0491	1.00
merck-AI341167_a_at	CD163	NM_004244 NM_203416	-0.283	-1.99	0.0491	1.00
merck2-NM_004895.2_at	NLRP3	NM_004895 NM_183395 NM_0010798z	-0.190	-1.99	0.0492	1.00
merck-NM_153742_at	CTH	NM_001902 NM_153742 NM_0011904€	0.123	1.99	0.0492	1.00
merck2-BU684534_at	---	---	-0.186	-1.99	0.0492	1.00
merck-BC045691_at	RALGPS2	NM_152663	0.232	1.99	0.0492	1.00
merck2-BC042674_at	PLB1	NM_153021 NM_001170585	-0.164	-1.99	0.0492	1.00
merck-AK056307_a_at	CUX1	NM_181552 NM_001202543 XR_10885	-0.145	-1.99	0.0492	1.00
merck2-BX648541_at	---	XR_108561 XR_112720 XR_113724	0.140	1.99	0.0492	1.00
merck-ENST00000333891_a_at	PCLO	NM_033026 NM_014510	-0.134	-1.99	0.0492	1.00

merck-NM_198570_at	VWC2	NM_198570	-0.0885	-1.99	0.0493	1.00
merck-CR610223_a_at	SCARB2	NM_005506 NM_001204255	-0.279	-1.99	0.0493	1.00
merck-NM_002595_s_at	CDK17	NM_002595 NM_001170464	0.182	1.99	0.0493	1.00
merck-AL117542_at	---	AL117542	-0.139	-1.99	0.0493	1.00
merck-AJ003145_at	OR1F2P	NR_002169	-0.146	-1.99	0.0493	1.00
merck-NM_003294_s_at	TPSAB1 TPSB2	NM_003294 NM_024164 XM_00311964	-0.185	-1.99	0.0494	1.00
merck-NM_002534_a_at	OAS1	NM_016816 NM_002534 NM_0010324(	-0.322	-1.99	0.0494	1.00
merck-BX404802_at	---	EF445017	-0.126	-1.99	0.0494	1.00
merck-NM_032974_at	CASP10	NM_032974	-0.121	-1.99	0.0494	1.00
merck2-AI147879_at	---	EU280320	-0.184	-1.99	0.0495	1.00
merck-BX640760_s_at	NBEA	NM_015678 NM_001204197	0.190	1.99	0.0495	1.00
merck2-BI765353_at	REEP5	NM_005669	0.147	1.99	0.0495	1.00
merck-AK125365_at	C19orf50	BX648739	-0.100	-1.99	0.0495	1.00
merck-XM_936352_at	---	---	-0.136	-1.99	0.0496	1.00
merck-ENST00000340005_x_at	---	---	0.0787	1.99	0.0496	1.00
merck-NM_207318_a_at	FAM199X	NM_207318	0.135	1.99	0.0496	1.00
merck-NM_054104_at	OR6C3	NM_054104	0.223	1.99	0.0496	1.00
merck-AK123850_at	CEACAM22P	NR_027754	-0.132	-1.99	0.0496	1.00
merck2-XM_937563_a_at	HAUS6	NM_017645	-0.145	-1.99	0.0496	1.00
merck-NM_001007255_at	KLHDC9	NM_152366 NM_001007255 NR_03338	-0.136	-1.99	0.0497	1.00
merck2-BX648405_s_at	DDX21	BX648405	0.178	1.99	0.0497	1.00
merck-NM_001005862_s_at	ERBB2	NM_004448 NM_001005862	-0.240	-1.99	0.0497	1.00
merck-AF075028_at	FXR1	NM_005087 NM_001013438 NM_0010	0.131	1.99	0.0498	1.00
merck-NM_005739_at	RASGRP1	NM_005739 NM_001128602	0.171	1.99	0.0498	1.00
merck2-BE614460_at	C11orf46	BX647617	0.224	1.99	0.0498	1.00
merck-NM_173473_s_at	ANAPC16	NM_173473	0.154	1.99	0.0498	1.00
merck-NM_001242_at	CD27	NM_001242	0.190	1.99	0.0498	1.00
merck-NM_004314_at	ART1	NM_004314	-0.151	-1.99	0.0499	1.00
merck2-AI654073_at	---	AL589842	0.221	1.99	0.0499	1.00
merck-NM_173506_at	LYPD4	NM_173506	-0.142	-1.99	0.0499	1.00
merck2-NM_001080495_x_at	TNRC18	NM_001080495	-0.128	-1.99	0.0499	1.00
merck-BU509154_at	PPP2R1B	NM_002716 NM_001177562 NM_00117	0.129	1.99	0.0499	1.00
merck-AA454219_at	---	EU332860	-0.141	-1.99	0.0499	1.00
merck-BU741143_at	---	---	-0.112	-1.99	0.0499	1.00
merck2-BQ441045_at	---	---	-0.116	-1.99	0.0500	1.00
merck2-ENST00000376242_at	PSORS1C3	NR_026816	-0.130	-1.99	0.0500	1.00

List of 2,227 transcript identified in the EM131 dataset as being differentially expressed between HBV vaccine responders and poor-responders (moderated t-test: nominal $p \leq 0.05$). 1,004 transcripts were induced in HBV vaccine responders (red) and 1,223 transcripts were repressed in HBV vaccine responders (blue) compared to HBV vaccine poor-responders. For each transcript, its probeset identifier, its HUGO gene symbol, RefSeq accession number, fold difference between HBV vaccine responders versus poor-responders and the moderated t-test p-values are given. The transcripts belonging to the 15-gene expression signature are highlighted in grey.

Supplementary Table 4b. Prediction of HBV vaccine response of the 15-gene signature on the EM131 test set

	predicted by the 15-gene signature	
HBV vaccine	responder	poor responder
responder	9	8
poor responder	9	19

The 15-gene signature has an accuracy of 62.2% on the EM131 test set

Supplementary Table 4c. Pathway enrichment analysis among genes differentially expressed between HBV vaccine responders and poor-responders

Pathway	Ratio	nominal p-value	adjusted p-value	Intersect
B-cell signaling				
B_CELL_DEVELOPMENT	0.406	1.70E-07	6.33E-05	IGHG1,CD79B,HLA-DQA1,CD40,IGHM,HLA-DOB,HLA-DQB1,HLA-DRB1,CD79A,I
ALTERED_T_CELL_AND_B_CELL_SIGNALING_IN_RHEUMATOID_AI	0.155	0.00868	0.231	IGHG1,PRTN3,CD79B,HLA-DQA1,CD40,IGHM,TNFSF13B,HLA-DOB,HLA-DQB1,H
RAS signaling				
VEGF_SIGNALING	0.176	0.00133	0.0991	FIGF,EIF1AY,EIF1AX,PIK3R3,RRAS2,VEGFC,ATM,YWHAE,SOS2,FOXO1,EIF2S3,
EIF2_SIGNALING	0.131	0.0461	0.409	EIF1AY,EIF1AX,PIK3R3,RRAS2,ATM,SOS2,EIF2S3,EIF2B3,EIF5,PIK3R6,INSR
Antigen presentation				
ALLOGRAFT_REJECTION_SIGNALING	0.175	0.00619	0.192	IGHG1,HLA-DRB4,HLA-DQA1,IGHG2,CD40,HLA-DOB,HLA-DQB1,HLA-DRB1,HLA
DENDRITIC_CELL_MATURATION	0.132	0.00687	0.196	IGHG1,MAPK14,HLA-DRB4,HLA-DQA1,IGHG2,PIK3R3,CD40,HLA-DOB,HLA-DQE
AUTOIMMUNE_THYROID_DISEASE_SIGNALING	0.170	0.0152	0.354	IGHG1,HLA-DQA1,IGHG2,CD40,HLA-DOB,HLA-DQB1,HLA-DRB1,FCER1G,HLA-A
OX40_SIGNALING_PATHWAY	0.156	0.0189	0.370	HLA-DRB4,TRAF5,HLA-DQA1,HLA-DOB,HLA-DQB1,HLA-DRB1,HLA-DRB3,FCER
CYTOTOXIC_T_LYMPHOCYTE-MEDIATED_APOPTOSIS_OF_TARGE	0.155	0.0264	0.377	HLA-DRB4,HLA-DQA1,HLA-DOB,HLA-DQB1,HLA-DRB1,HLA-DRB3,FCER1G,BCL
ANTIGEN_PRESENTATION_PATHWAY	0.159	0.0422	0.409	HLA-DRB4,HLA-DQA1,HLA-DOB,HLA-DRB1,HLA-DRB3,HLA-A,HLA-L
G-protein coupled receptor signaling				
G_PROTEIN_SIGNALING_MEDIATED_BY_TUBBY	0.174	0.0189	0.370	PLCB3,GNA14,GNG13,GNG7,GNA11,GNG4,GNAT1,INSR
CCR3_SIGNALING_IN_EOSINOPHILS	0.125	0.0275	0.377	MAPK14,PLCB3,GNA14,PIK3R3,RRAS2,GNG13,GNG7,PAK3,ATM,ITPR1,ROCK2,
P2Y_PURIGENIC_RECEPTOR_SIGNALING_PATHWAY	0.126	0.0301	0.386	PLCB3,ITGB3,P2RY2,PRKACB,PIK3R3,RRAS2,GNG13,GNG7,ADCY9,ATM,PLCE
FMLP_SIGNALING_IN_NEUTROPHILS	0.122	0.0389	0.409	PLCB3,GNA14,PIK3R3,RRAS2,GNG13,GNG7,ATM,ITPR1,NOX1,CYBB,GNA11,PI
IL-1_SIGNALING	0.126	0.0485	0.409	MAPK14,GNA14,MAP2K6,PRKACB,GNG13,GNG7,ADCY9,TAB2,GNA11,GNG4,GI
Others				
ROLE_OF_NFAT_IN_REGULATION_OF_THE_IMMUNE_RESPONSE	0.162	2.46E-05	0.00457	IGHG1,BLNK,KPNA6,PLCB3,GNA14,CD79B,HLA-DQA1,PIK3R3,RRAS2,IGHM,GN
PRIMARY_IMMUNODEFICIENCY_SIGNALING	0.255	0.000118	0.0147	IGHG1,BLNK,IGHG2,CD40,IGHM,DCLRE1C,CD79A,IGHD,IGLC1,IL7R,TNFRSF13
IL-8_SIGNALING	0.146	0.000499	0.0464	DEFA1,DEFA1B,DEFA3,FIGF,GNA14,AZU1,PIK3R3,RRAS2,GNG13,VEGFC,GNG7
IL-4_SIGNALING	0.186	0.00171	0.106	HLA-DQA1,PIK3R3,RRAS2,INPPL1,HLA-DOB,HLA-DQB1,ATM,FCER2,HLA-DRB1
PI3K_SIGNALING_IN_B_LYMPHOCYTES	0.148	0.00219	0.109	IGHG1,BLNK,PLCB3,CD79B,RRAS2,CD40,MALT1,IGHM,PIK3AP1,BLK,ITPR1,CD
INTERFERON_SIGNALING	0.242	0.00235	0.109	IRF9,MED14,STAT1,MX1,BCL2,BAK1,IFIT3,OAS1
DNA_DOUBLE-STRAND_BREAK_REPAIR_BY_HOMOLOGOUS_REC	0.333	0.00360	0.149	MRE11A,ATRX,ATM,RAD51,POLA1
SYSTEMIC_LUPUS_ERYTHEMATOSUS_SIGNALING	0.146	0.00409	0.151	IGHG1,CD22,CD79B,IGHG2,PIK3R3,RRAS2,CD40,IGHM,TNFSF13B,ATM,CD79A
RENAL_CELL_CARCINOMA_SIGNALING	0.174	0.00447	0.151	HGF,GAB1,PIK3R3,RRAS2,VHL,PAK3,ATM,SOS2,PDGFB,PIK3R6,EGLN3,MET
MACROPINOCYTOSIS_SIGNALING	0.159	0.0123	0.304	HGF,ITGB3,PIK3R3,RRAS2,CD14,ATM,PDGFB,EGF,PIK3R6,MET,PRKD1
CD40_SIGNALING	0.156	0.0189	0.370	MAPK14,TRAF5,MAP2K6,PIK3R3,CD40,ATM,FCER2,PIK3R6,ATF1,IKBKG
FGF_SIGNALING	0.143	0.0209	0.377	HGF,MAPK14,GAB1,MAP2K6,PIK3R3,ATM,ITPR1,SOS2,FGF18,PIK3R6,FGF1,ME
B_CELL_RECEPTOR_SIGNALING	0.123	0.0228	0.377	BLNK,CD22,MAPK14,CD79B,GAB1,MAP2K6,PIK3R3,RRAS2,MALT1,PIK3AP1,INF
ROLE_OF_TISSUE_FACTOR_IN_CANCER	0.132	0.0248	0.377	MAPK14,ITGB3,GNA14,PIK3R3,RRAS2,VEGFC,BLK,ATM,FGA,GNA11,PIK3R6,FC
MYC_MEDIATED_APOPTOSIS_SIGNALING	0.155	0.0264	0.377	PIK3R3,RRAS2,ATM,YWHAQ,YWHAE,SOS2,BCL2,IGF1R,PIK3R6
RAN_SIGNALING	0.250	0.0270	0.377	KPNA6,TNPO1,XPO1,KPNB1
GLUCOCORTICOID_RECEPTOR_SIGNALING	0.107	0.0277	0.377	HSPA1B,POMC,MAPK14,PRKACB,PIK3R3,RRAS2,AR,ATM,PTGES3,MED14,STA
PI3K_AKT_SIGNALING	0.122	0.0284	0.377	ITGB3,GAB1,PIK3R3,RRAS2,INPPL1,YWHAQ,YWHAE,ITGA9,SOS2,FOXO1,BCL
FCGAMMARIIB_SIGNALING_IN_B_LYMPHOCYTES	0.139	0.0312	0.387	IGHG1,BLNK,CD79B,PIK3R3,RRAS2,IGHM,ATM,ITPR1,CD79A,FCGR2B,PIK3R6
COLORECTAL_CANCER_METASTASIS_SIGNALING	0.108	0.0324	0.388	FIGF,GNA14,PRKACB,PIK3R3,RRAS2,GNG13,VEGFC,GNG7,ADCY9,ATM,MMP1
HIF1ALPHA_SIGNALING	0.129	0.0360	0.409	MAPK14,FIGF,PIK3R3,RRAS2,VEGFC,VHL,ATM,MMP19,LDHB,MMP11,PIK3R6,E
ENDOMETRIAL_CANCER_SIGNALING	0.154	0.0369	0.409	PIK3R3,RRAS2,ATM,LEF1,SOS2,CTNNA1,PIK3R6,ERBB2
P70S6K_SIGNALING	0.122	0.0389	0.409	IGHG1,PLCB3,CD79B,PIK3R3,RRAS2,IGHM,ATM,CD79A,YWHAQ,YWHAE,SOS2
CELL_CYCLE:_G2_M_DNA_DAMAGE_CHECKPOINT_REGULATION	0.156	0.0469	0.409	ATM,CCNB2,TOP2A,YWHAQ,YWHAE,SKP1,LOC728622
PPARALPHA_RXRALPHA_ACTIVATION	0.113	0.0478	0.409	MAPK14,PLCB3,GPD2,MAP2K6,PRKACB,RRAS2,ADCY9,SOS2,PLCE1,SMAD3,F

List of 40 pathways from the Ingenuity canonical pathway database significantly enriched among genes differentially expressed between HBV vaccine responders and poor-responders (Fisher's exact test: $p \leq 0.05$). The list of differentially expressed genes overlapping each pathway is given. Pathways that overlap in terms of genes (Jaccard index ≥ 0.25) were regrouped and can be thought as implicated in a common biological function. Genes induced in HBV vaccine responders are indicated in red and genes repressed in HBV vaccine responders are indicated in blue.

Legend
- (red) Genes induced in HBV vaccine responders compared to poor-responders
- (blue) Genes repressed in HBV vaccine responders compared to poor-responders

Supplementary Table 5a. List of FCM markers measured on the EM131 cohort

Panel Name	Column Label	Cell Surface Marker
T cell	CT CD3	CD3+
T cell	CT CD4	CD3+, CD4+
T cell	CT CD8	CD3+, CD8+
T cell	CT DP	CD3+, CD4+, CD8+
T cell	CT DN	CD3+, CD4-, CD8-
T cell	CT DR+ B	CD3-, CD19+, HLADR+
T cell	CT DR- B	CD3-, CD19+, HLADR-
T cell	CT Mono	CD3-, CD4-, CD19-, CD14+
T cell	CT pDC	CD3-, CD4+, CD19-, CD14-, CD16-, CD56-, CD11c-, HLADR+, CD303+
T cell	CT mDC1	CD3-, CD4+, CD19-, CD14-, CD16-, CD56-, CD11c+, HLADR+, CD1c+, CD303-
T cell	CT mDC2	CD3-, CD4+, CD19-, CD14-, CD16-, CD56-, CD11c+, HLADR+, CD141+
T cell	CT ALL NK	CD3-, CD4-, CD19-, CD14-
T cell	CT 56-16+ NK	CD3-, CD4-, CD19-, CD14-, CD16+, CD56-
T cell	CT NK 56bright16-	CD3-, CD4-, CD19-, CD14-, CD16-, CD56++
T cell	CT NK 56low16-	CD56, CD16
T cell	CT NK 56low16+	CD56, CD16
T cell	CT NK 56bright16low	CD56, CD16
T-cell	PCT CD4 in CD3	CD3+, CD4+
T-cell	PCT CD8 in CD3	CD3+, CD8+
T-cell	PCT CD28- in CD8	CD3+, CD28-
T-cell	PCT CD62L+ in CD8	CD3+, CD8+, CD62L+
T-cell	PCT HLADR+CD38+ in CD4	CD3+, CD4+, CD38+, HLADR+
T-cell	PCT HLADR+CD38+ in CD8	CD3+, CD8+, CD38+, HLADR+
T-cell	PCT PD1+ in CD4	CD3+, CD4+, PD1+
T-cell	PCT PD1+ in CD8	CD3+, CD8+, PD1+
T-cell	PCT CD57+ in CD4	CD3+, CD4+, CD57+
T-cell	PCT CD57+ in CD8	CD3+, CD8+, CD57+
T-cell	PCT Naive in CD4	CD3+, CD4+, CD45RA+, CD27+, CCR7+
T-cell	PCT CM in CD4	CD3+, CD4+, CD45RA-, CD27+, CCR7+
T-cell	PCT EM in CD4	CD3+, CD4+, CD45RA-, CD27-, CCR7-
T-cell	PCT TEM1 in CD4	CD3+, CD4+, CD45RA-, CD27-, CCR7-, CD28+
T-cell	PCT TEM2 in CD4	CD3+, CD4+, CD45RA-, CD27-, CCR7-, CD28-
T-cell	PCT ILD in CD4	CD3+, CD4+, CD45RA+, CD27-, CCR7+
T-cell	PCT LD in CD4	CD3+, CD4+, CD45RA+, CD27-, CCR7-
T-cell	PCT Naive in CD8	CD3+, CD8+, CD45RA+, CD27+, CCR7+
T-cell	PCT CM in CD8	CD3+, CD8+, CD45RA-, CD27+, CCR7+
T-cell	PCT EM in CD8	CD3+, CD8+, CD45RA-, CD27-, CCR7-
T-cell	PCT TEM1 in CD8	CD3+, CD8+, CD45RA-, CD27-, CCR7-, CD28+
T-cell	PCT TEM2 in CD8	CD3+, CD8+, CD45RA-, CD27-, CCR7-, CD28-
T-cell	PCT ILD in CD8	CD3+, CD8+, CD45RA+, CD27-, CCR7+
T-cell	PCT LD in CD8	CD3+, CD8+, CD45RA+, CD27-, CCR7-
T-cell	PCT CD28+CD62L+Naive in CD4	CD3+, CD4+, CD45RA+, CD27+, CCR7+, CD28+, CD62L+
T-cell	PCT CD28+CD62L+Naive in CD8	CD3+, CD8+, CD45RA+, CD27+, CCR7+, CD28+, CD62L+
T-cell	PCT CD28+CD62L+CM in CD4	CD3+, CD4+, CD45RA-, CD27+, CCR7+, CD28+, CD62L+
T-cell	PCT CD28+CD62L+CM in CD8	CD3+, CD8+, CD45RA-, CD27+, CCR7+, CD28+, CD62L+
T-cell	PCT CD45RA- in CD4	CD3+, CD4+, CD45RA-
T-cell	PCT CD45RA- in CD8	CD3+, CD8+, CD45RA-
T-cell	RATIO CD4 to CD8	ratio of respective categories above
T-cell	RATIO CD28+CD62L+Naïve to CD45RA- in CD4	ratio of respective categories above
T-cell	RATIO CD28+CD62L+Naïve to CD45RA- in CD8	ratio of respective categories above
T-cell	PCT RATIO CD28+CD62L+CM to CD45RA-andILDandLD in CD4	ratio of respective categories above
T-cell	PCT RATIO CD28+CD62L+CM to CD45RA-andILDandLD in CD8	ratio of respective categories above
B-cell	PCT CD3+ T cells in Lymphocytes	CD3+
B-cell	PCT DR+ B cells in Lymphocytes	CD3-, CD19+, HLADR+
B-cell	PCT DR- B cells in Lymphocytes	CD3-, CD19+, HLADR-
B-cell	PCT switched IgG+ memory B cells	CD3-, CD19+, HLADR+, CD27+, IgG+
B-cell	PCT switched IgA+ MK memory B cells	CD3-, CD19+, HLADR+, CD27+, IgA+
B-cell	PCT unswitched IgM+ MK memory B cells	CD3-, CD19+, HLADR+, CD27+, IgM+
B-cell	PCT MZ like B cells	CD3-, CD19+, HLADR+, CD27+,B220+
B-cell	PCT immature B cells	CD3-, CD19+, HLADR+, CD27-, CD10+, IgM+, IgD-
B-cell	PCT IgM-IgD+ naive B cells	CD3-, CD19+, HLADR+, CD27-, CD10-, CD20-, IgM-, IgD+
B-cell	PCT IgM+IgD+ naive B cells	CD3-, CD19+, HLADR+, CD27-, CD10-, CD20-, IgM+, IgD+
B-cell	PCT IgM+IgD- naive B cells	CD3-, CD19+, HLADR+, CD27-, CD10-, CD20-, IgM+, IgD-
B-cell	PCT IgA+ memory B cells	CD3-, CD19+, HLADR+, CD27+, CD10-, CD20+, IgA+
B-cell	PCT IgG+ memory B cells	CD3-, CD19+, HLADR+, CD27+, CD10-, CD20+, IgG+
B-cell	PCT IgM+IgD+ memory B cells	CD3-, CD19+, HLADR+, CD27+, CD10-, CD20+, IgM+, IgD+
B-cell	PCT IgM+IgD- memory B cells	CD3-, CD19+, HLADR+, CD27+, CD10-, CD20+, IgM+, IgD-
B-cell	PCT IgM-IgD+ memory B cells	CD3-, CD19+, HLADR+, CD27+, CD10-, CD20+, IgM-, IgD+
B-cell	PCT plasma B cells	CD3-, CD19+, HLADR+, CD27+, CD10-, CD20-
B-cell	PCT IgA+ plasma B cells	CD3-, CD19+, HLADR+, CD27+, CD10-, CD20-, IgA+
B-cell	PCT IgD+ plasma B cells	CD3-, CD19+, HLADR+, CD27+, CD10-, CD20-, IgD+
B-cell	PCT IgG+ plasma B cells	CD3-, CD19+, HLADR+, CD27+, CD10-, CD20-, IgG+

B-cell	PCT IgM+ plasma B cells	CD3-, CD19+, HLADR+, CD27+, CD10-, CD20-, IgM+
Innate	PCT CD4 in WBC	CD3+, CD4+
Innate	PCT Mono in WBC	CD3-, CD4-, CD19-, CD14+
Innate	PCT CD16+ in Mono	CD3-, CD4-, CD19-, CD14+, CD16+
Innate	PCT NK 56-16+ in WBC	CD3-, CD4-, CD19-, CD14-, CD16+, CD56-
Innate	PCT NK 56bright16- in WBC	CD3-, CD4-, CD19-, CD14-, CD16-, CD56+
Innate	PCT NK 56low16- in WBC	CD3-, CD4-, CD19-, CD14-, CD16-, CD56lo
Innate	PCT NK 56low16+ in WBC	CD3-, CD4-, CD19-, CD14-, CD16+, CD56lo
Innate	PCT NK 56bright16low in WBC	CD3-, CD4-, CD19-, CD14-, CD16lo, CD56++
Innate	PCT pDC in WBC	CD3-,CD4+CD19-,CD14-CD16-,CD56-CD11c-,HLADR+,CD303+
Innate	PCT mDC1 in WBC	CD3-,CD4+CD19-,CD14-CD16-,CD56-CD11c+,HLADR+,CD1c+,CD303-
Innate	PCT mDC2 in WBC	CD3-,CD4+CD19-,CD14-CD16-,CD56-CD11c+,HLADR+,CD141+
Innate	PCT DRhi40+ in pDC	HLADR, CD40
Innate	PCT DRhi40+ in mDC1	HLADR, CD40
Innate	PCT DRhi40+ in mDC2	HLADR, CD40
Innate	MdFI DR in pDC	HLADR
Innate	MdFI CD4 in pDC	CD4
Innate	MdFI CD40 in pDC	CD40
Innate	MdFI DR in mDC1	HLADR
Innate	MdFI CD4 in mDC1	CD4
Innate	MdFI CD40 in mDC1	CD40
Innate	MdFI DR in mDC2	HLADR
Innate	MdFI CD4 in mDC2	CD4
Innate	MdFI CD40 in mDC2	CD40

FCM panel are colored differently; blue was used for T-cells, orange for B-cells and green for innate cells.

Supplementary Table 5b. List of chemokines and cytokines measured on the EM131 cohort

Name
Alpha-1-Antitrypsin (AAT)
Alpha-2-Macroglobulin (A2Macro)
Beta-2-Microglobulin (B2M)
Brain-Derived Neurotrophic Factor (BDNF)
C-Reactive Protein (CRP)
Complement C3 (C3)
Eotaxin-1
Factor VII
Ferritin (FRTN)
Fibrinogen
Granulocyte-Macrophage Colony-Stimulating Factor (GM-CSF)
Haptoglobin
Intercellular Adhesion Molecule 1 (ICAM-1)
Interferon gamma (IFN-gamma)
Interleukin-1 alpha (IL-1 alpha)
Interleukin-1 beta (IL-1 beta)
Interleukin-1 receptor antagonist (IL-1ra)
Interleukin-10 (IL-10)
Interleukin-12 Subunit p40 (IL-12p40)
Interleukin-12 Subunit p70 (IL-12p70)
Interleukin-15 (IL-15)
Interleukin-17 (IL-17)
Interleukin-18 (IL-18)
Interleukin-2 (IL-2)
Interleukin-23 (IL-23)
Interleukin-3 (IL-3)
Interleukin-4 (IL-4)
Interleukin-5 (IL-5)
Interleukin-6 (IL-6)
Interleukin-7 (IL-7)
Interleukin-8 (IL-8)
Macrophage Inflammatory Protein-1 alpha (MIP-1 alpha)
Macrophage Inflammatory Protein-1 beta (MIP-1 beta)
Matrix Metalloproteinase-2 (MMP-2)
Matrix Metalloproteinase-3 (MMP-3)
Matrix Metalloproteinase-9 (MMP-9)
Monocyte Chemotactic Protein 1 (MCP-1)
Stem Cell Factor (SCF)
T-Cell-Specific Protein RANTES (RANTES)
Tissue Inhibitor of Metalloproteinases 1 (TIMP-1)
Tumor Necrosis Factor alpha (TNF-alpha)
Tumor Necrosis Factor beta (TNF-beta)
Tumor Necrosis Factor Receptor 2 (TNFR2)
Vascular Cell Adhesion Molecule-1 (VCAM-1)
Vascular Endothelial Growth Factor (VEGF)
Vitamin D-Binding Protein (VDBP)
von Willebrand Factor (vWF)

Supplementary Table 5c. Identification of cytokines/chemokines markers associated with response to HBV vaccine on the EM131 training set

Cytokine marker	Univariate logistic regression			Multivariate logistic regression		
	OR	95% CI	*p*-value	OR	95% CI	*p*-value
Beta-2-Microglobulin (B2M)	1.56	[1.04, 2.46]	0.0397	0.938	[0.486, 1.79]	0.845
Interleukin-15 (IL-15)	2.15	[0.986, 5.27]	0.0702	1.14	[0.361, 3.56]	0.824
Interleukin-1 receptor antagonist (IL-1ra)	2.10	[0.925, 5.43]	0.0979	2.09	[0.652, 8.06]	0.244
Stem Cell Factor (SCF)	1.79	[1.01, 3.30]	0.0517	1.28	[0.570, 2.91]	0.548
Tumor Necrosis Factor Receptor 2 (TNFR2)	1.61	[1.07, 2.60]	0.0362	1.34	[0.653, 2.91]	0.440
Vascular Cell Adhesion Molecule-1 (VCAM-1)	1.47	[0.976, 2.36]	0.0839	1.07	[0.551, 2.16]	0.841
Vascular Endothelial Growth Factor (VEGF)	1.60	[1.01, 2.63]	0.0521	1.12	[0.636, 2.00]	0.697

Univariate and multivariate logistic regression between cytokines/chemokines marker levels pre-vaccination (Visit 2) and response to HBV vaccine in the EM131 training set. P-values of a normality test (z-test) testing the statistical significance of the association (null hypothesis: OR=1) are given in the table. Markers selected using the forward selection method based on AIC criteria (in grey) were combined in multivariate models.
